# A taxonomic monograph of Nearctic *Scolytus* Geoffroy (Coleoptera, Curculionidae, Scolytinae)

**DOI:** 10.3897/zookeys.450.7452

**Published:** 2014-10-29

**Authors:** Sarah M. Smith, Anthony I. Cognato

**Affiliations:** 1 Department of Entomology, Michigan State University, Natural Science Building, room 243, 288 Farm Lane, East Lansing, MI 48824, United States of America

**Keywords:** Scolytidae, bark beetle, fir engraver, banded elm bark beetle, lesser elm bark beetle, Douglas-fir engraver, hickory bark beetle, shot hole borer, large shot hole borer, hackberry engraver, taxonomy, revision

## Abstract

The Nearctic bark beetle genus *Scolytus* Geoffroy was revised based in part on a molecular and morphological phylogeny. Monophyly of the native species was tested using mitochondrial (COI) and nuclear (28S, CAD, ArgK) genes and 43 morphological characters in parsimony and Bayesian phylogenetic analyses. Parsimony analyses of molecular and combined datasets provided mixed results while Bayesian analysis recovered most nodes with posterior probabilities >90%. Native hardwood- and conifer-feeding *Scolytus* species were recovered as paraphyletic. Native Nearctic species were recovered as paraphyletic with hardwood-feeding species sister to Palearctic hardwood-feeding species rather than to native conifer-feeding species. The Nearctic conifer-feeding species were monophyletic. Twenty-five species were recognized. Four new synonyms were discovered: *Scolytus praeceps* LeConte, 1868 (= *Scolytus abietis* Blackman, 1934; = *Scolytus opacus* Blackman, 1934), *Scolytus reflexus* Blackman, 1934 (= *Scolytus virgatus* Bright, 1972; = *Scolytus wickhami* Blackman, 1934). Two species were reinstated: *Scolytus fiskei* Blackman, 1934 and *Scolytus silvaticus* Bright, 1972. A diagnosis, description, distribution, host records and images were provided for each species and a key is presented to all species.

## Introduction

This monograph presents a revision of the Nearctic *Scolytus* Geoffroy, 1762 species (Curculionidae: Scolytinae). The Scolytinae is comprised of approximately 6,000 species in 225 genera and 26 tribes (Alonso-Zarazaga and Lyal 2009). Termed ‘bark and ambrosia beetles’ this weevil subfamily is ubiquitous in forests worldwide. Many species contribute to the decomposition of dead vegetation while others are capable of causing substantial host mortality. True bark beetles, including *Scolytus*, feed exclusively on phloem and cambium of living, declining, or dead trees and some species specialize on different plant parts from root to fruit and pith (Wood 1982). Beetle feeding on phloem and xylem hastens the decomposition of trees and the introduction of other xylophagous organisms (Stokland 2012). Some bark beetle species kill live trees by passively introducing fungi into otherwise healthy hosts as a by-product of phloem feeding and the galleries may girdle the host causing mortality (Wood 1982). At high density these species cause widespread destruction of economically valuable tree species, giving bark beetles their nefarious reputation as ecologically and economically destructive forest pests (Furniss and Carolin 1977). Other economically important bark beetle genera such as *Dendroctonus* Erichson, 1836 and *Ips* DeGeer, 1775 have received considerable attention and therefore have a reasonably solid understanding of the taxonomy (Wood and Bright 1992). *Scolytus* is also important but has received considerably less attention.

## Natural history

*Scolytus* are distributed in the Holarctic, Oriental (Himalayan) and Neotropical regions and some Palearctic *Scolytus* species have been introduced around the world to the Nearctic, South Africa, Australia, New Zealand and temperate South America (Rosel and French 1975; Bain 1990; Wood and Bright 1992; Six et al. 2005; Wood 2007; Smith and Cognato 2013). In the Nearctic region, *Scolytus* species occur from the Atlantic to Pacific oceans and from the boundary of the Neotropical region to the northern limits of tree growth. In the Nearctic, native hardwood-feeders are generally found from the Atlantic coast to Texas and west to the foothills of the Rocky Mountains. With the exception of *Scolytus piceae* (Swaine, 1910), conifer-feeding *Scolytus* are restricted to the occurrence of host trees in western mountain ranges including the Rocky Mountains. *Scolytus piceae* has an expansive range from the east and west coasts of North America and from northern California and Colorado north to beyond the Arctic Circle. Invasive *Scolytus* species are found throughout the US, northern Mexico and southern Canada (*Scolytus mali* (Bechstein, 1805), *Scolytus multistriatus* (Marsham, 1802), *Scolytus rugulosus* (Müller, 1818), and *Scolytus schevyrewi* Semenov, 1902).

In North America, *Scolytus* species occur either in conifer or hardwood hosts (Table 1). Members of the *Scolytus* conifer-feeding clade feed exclusively on Pinaceae genera, including *Abies* Mill., *Larix* Mill., *Picea* D. Don. ex Loudon, *Pseudotsuga* Carrière, and *Tsuga* Carrière, with the notable exception of *Pinus* L. (Wood and Bright 1992) (Table 2). Members of the hardwood-feeding clade, *Scolytus muticus* Say, 1824, *Scolytus fagi* Walsh, 1867, *Scolytus quadrispinosus* Say, 1824 feed on the families Cannabaceae (*Celtis* L.), Fagaceae (*Fagus* L., *Quercus* L.) and Juglandaceae (*Carya* Nutt.), respectively (Table 1).

**Table 1.** Host plant families for all Nearctic *Scolytus*.

Species	Cannabaceae	Fagaceae	Juglandaceae	Pinaceae	Rosaceae	Ulmaceae
*Scolytus aztecus*				■		
*Scolytus dentatus*				■		
*Scolytus fagi*		■				
*Scolytus fiskei*				■		
*Scolytus hermosus*				■		
*Scolytus laricis*				■		
*Scolytus mali*					■	■
*Scolytus monticolae*				■		
*Scolytus multistriatus*						■
*Scolytus mundus*				■		
*Scolytus muticus*	■					
*Scolytus obelus*				■		
*Scolytus oregoni*				■		
*Scolytus piceae*				■		
*Scolytus praeceps*				■		
*Scolytus quadrispinosus*			■			
*Scolytus reflexus*				■		
*Scolytus robustus*				■		
*Scolytus rugulosus*					■	
*Scolytus schevyrewi*						■
*Scolytus silvaticus*				■		
*Scolytus subscaber*				■		
*Scolytus tsugae*				■		
*Scolytus unispinosus*				■		
*Scolytus ventralis*				■		

**Table 2.** Pinaceae host genus for conifer-feeding Nearctic *Scolytus*.

Species	*Abies*	*Larix*	*Picea*	*Pseudotsuga*	*Tsuga*
*Scolytus aztecus*	■				
*Scolytus dentatus*	■				
*Scolytus fiskei*				■	
*Scolytus hermosus*	■				
*Scolytus laricis*		■			
*Scolytus monticolae*				■	
*Scolytus mundus*	■				
*Scolytus obelus*	■				
*Scolytus oregoni*				■	
*Scolytus piceae*			■		
*Scolytus praeceps*	■				
*Scolytus reflexus*				■	
*Scolytus robustus*	■				
*Scolytus silvaticus*				■	
*Scolytus subscaber*	■				
*Scolytus tsugae*					■
*Scolytus unispinosus*				■	
*Scolytus ventralis*	■				

In the Nearctic region, six *Scolytus* species have the potential to kill host trees and cause significant mortality of either conifers (Cibrián Tovar et al. 1995) or *Carya* spp. (Furniss and Carolin 1977). Two of these are the exotic *Scolytus multistriatus* and *Scolytus schevyrewi*, both are key vectors of the Dutch elm disease fungi, pathogens that have killed millions of *Ulmus* L. spp. trees in forest and urban areas across much of the US (Furniss and Carolin 1977). Mortality caused by various *Scolytus* species is often sporadic and short-term, although some outbreaks locally affect thousands of acres a year (Furniss and Carolin 1977). Damage is most severe in times of environmental stress, which is mostly associated with drought and other insect infestations. For example, *Scolytus ventralis* LeConte, 1876 killed approximately 72,843 hectares of *Abies* spp. in California alone in a year (Furniss and Carolin 1977).

All Nearctic *Scolytus* species are monogamous. Females select brood material and begin galley construction. Males walk across the host material in search of females. Mating occurs with the female in the entrance tunnel and the male on the bark. However, mating in twig crotches has been reported for *Scolytus multistriatus* (Svihra and Clark 1980). The female creates an entrance tunnel at a 45° angle, boring through the bark to the cambium. From the entrance tunnel, she excavates a nuptial chamber and, depending on the species, one or two egg galleries in either direction from the entrance tunnel. The nuptial chamber and galleries are excavated in the cambium but also etch the sapwood. Females excavate egg niches on both sides of the egg galleries and a single egg is deposited in each niche and covered with boring dust. Adult males assist in removing frass and generally stay with the female until egg gallery construction is complete. The male then leaves the gallery and the female dies in the entrance tunnel with her abdomen projecting onto the bark surface. Larval galleries radiate away from the egg tunnels as they feed on phloem, also etching the sapwood. Once larvae mature, the prepupae burrow into the outer sapwood and pupate. The brood overwinters as pupae and adults emerge in the spring (Edson 1967). Upon emergence, *Scolytus mali*, *Scolytus multistriatus*, *Scolytus quadrispinosus*, *Scolytus rugulosus*, and *Scolytus schevyrewi* engage in maturation feeding at twig crotches and/or leaf petioles (Hoffman 1942; Baker 1972; Negrón et al. 2005). Feeding may also occur within small twigs in *Scolytus fiskei* Blackman, 1934 (reported as *Scolytus unispinosus* LeConte, 1876 in McMullen and Atkins 1962).

Gallery shape is directly related to the resin system of the host genus. *Larix*, *Picea* and *Pseudotsuga* possess an elaborate system of resin ducts with vertical and radial ducts that are connected to each other (Lieutier 2004). *Scolytus* galleries in these genera are consistently parallel to the grain of the wood. The vertical ducts are abundant in these tree genera and when *Scolytus* constructs a vertical egg gallery, both ducts are severed, and as the gallery is elongated, only vertical canals are severed. This minimizes exposure of *Scolytus* to host resins (Lieutier 2004). *Abies* and *Tsuga* lack resin canals and instead possess resin blisters. Galleries in these genera are quite variable and may be transverse, “V” or (“**ε**”) shaped depending on the species because there are no resin canals to avoid (Lieutier 2004).

Conifer-feeding *Scolytus* species exhibit primary attraction to host volatiles rather than to pheromones produced by conspecifics (Macías-Sámano et al. 1998a). Attraction of hardwood-feeding species is not well understood but primary attraction seems probable for native *Scolytus quadrispinosus* and the invasive species *Scolytus rugulosus* and *Scolytus schevyrewi* (Goeden and Norris 1964a; Kovach and Gorsuch 1985; Lee et al. 2010). *Scolytus multistriatus* exhibits secondary attraction and to 4-methyl-3-heptanol and multistriatin in combination with alpha-cubenene (Lanier et al. 1977). At endemic population levels, *Scolytus* infest over mature, unthrifty or weakened standing trees, shaded out branches, recent logging slash, windthrown trees and fallen branches. During outbreaks, vigorous trees may be colonized by more aggressive species, including *Scolytus quadrispinosus*, *Scolytus mundus* Wood, 1968 and *Scolytus ventralis* LeConte, 1868 (Edson 1967; Furniss and Carolin 1977), and the secondary species *Scolytus monticolae* (Swaine, 1917), *Scolytus reflexus* Blackman, 1934 [reported as *Scolytus monticolae*] and *Scolytus unispinosus* LeConte, 1876 (McMullen and Atkins 1959; USDA 2004). While *Scolytus* species are generally common in the forest, they are rarely abundant and seldom encountered by collectors. *Scolytus* species prefer fresh and moist host material. Sun-baked material is not preferred, but *Scolytus* galleries may be present in cooler, moister areas of the bark that are heavily shaded or on the underside of fallen trees and branches. Infested conifer branches and trees typically bear green needles while infested hardwoods have yellowing leaves (Smith, pers. obs.). The entrance can be identified by peeling off the bark flakes with a knife and searching for white to tan-colored boring dust or by close examination of the lateral and ventral sides of trunks and branches of smooth barked hosts. In *Pseudotsuga*, the entrance tunnels of *Pseudohylesinus* Swaine, 1917 can be easily confused with those of *Scolytus*, but *Scolytus* entrance tunnels are at a steeper angle and the boring dust is reddish rather than white. Some *Scolytus* species, including *Scolytus piceae*, *Scolytus monticolae* and *Scolytus subscaber* LeConte, 1876 typically attack shaded out branches on standing trees. Yellowing needles are indicative of an infested tree. *Scolytus* females typically conceal their entrance tunnels under bark flakes (common in *Picea*, *Pseudotsuga*, *Tsuga*) or in rough patches of bark (*Abies*, *Larix*).

## Sexual dimorphism

**Figure 1.**
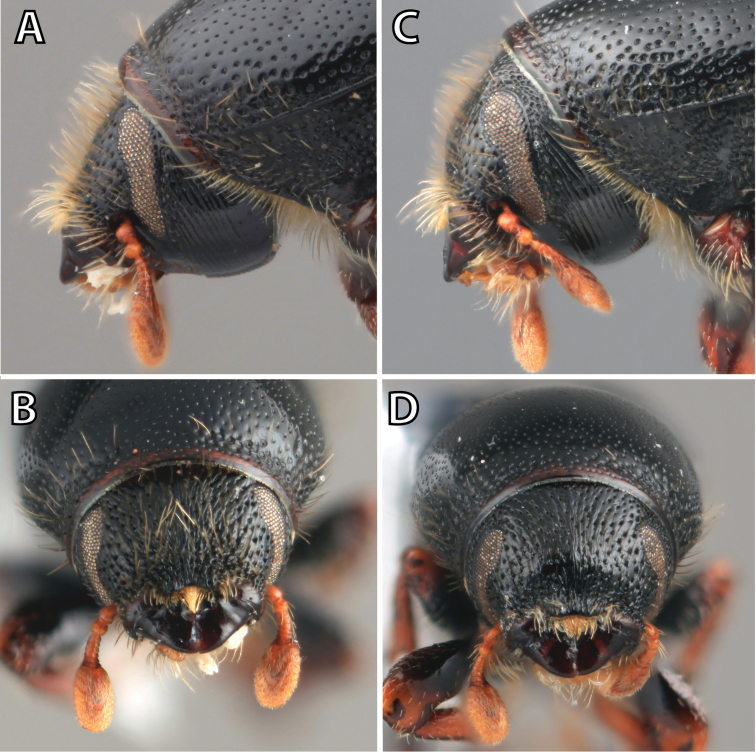
*Scolytus* typical sexual dimorphism of the frons and epistoma as exhibited by *Scolytus reflexus*. **A** male frons, frontal **B** male frontal oblique **C** female frons, frontal **D** female frontal oblique.

*Scolytus* species are sexually dimorphic. Sexually dimorphic structures are typically on the frons and epistoma (Fig. 1) and the abdominal ventrites and vary by clade (see clade discussions in results). When viewed laterally, males typically have a flattened, impressed frons and the female frons is always more strongly convex. The male frons is more strongly and coarsely longitudinally aciculate than the female and covered with longer, more abundant and dense erect setae. The epistomal process (when present) is more strongly developed in the male and less developed in the female. With the exception of *Scolytus piceae*, the spines, tubercles, denticles, carinae and tumescence on the venter are more pronounced in males.

## Systematics

The Scolytini currently contains 213 species and six genera including *Camptocerus* Dejean, 1821, *Ceratolepis* Chapuis, 1869, *Cnemonyx* Eichhoff, 1868, *Loganius* Chapuis, 1869, *Scolytopsis* Blandford, 1896 and *Scolytus* (Smith and Cognato 2013). With the exception of *Camptocerus*, the tribe consists of cambium-feeding bark beetles. *Scolytus* species that feed exclusively on either hardwoods or conifers (Wood 1986). Twenty Nearctic and three Palearctic species feed on conifers. All other Scolytini are restricted to angiosperm hosts (Wood and Bright 1992; Smith and Cognato 2010b; Smith and Cognato 2013).

A single apical, unarmed spine-like process that curves toward and extends beyond the process of the inner apical angle of the protibia and by a seven-segmented funicle readily distinguishes the tribe. All display a strongly sexually dimorphic head bearing hair-like setae, with the male frons variously excavated and female frons typically flat to convex. The eye is entire and the posterior area of head is subtruncate. The pronotum is unarmed with a costate lateral margin. In addition, the metapleural suture descends subventrally to the groove, receiving the groove on the costal margin of the elytra, then turns abruptly and parallels the groove near the metacoxal process (Wood 1972, 1978, 1982, 1986). *Scolytus* is perhaps one of the easiest bark beetle genera to recognize with its distinctive ascendant second abdominal ventrite, the slightly if at all declivous elytra and the depressed basal area of the elytra around the triangular scutellum (Wood 1982).

## Taxonomic history

There are currently about 127 recognized *Scolytus* species in the world (Wood and Bright 1992; Wood 2007; Knížek 2011; Petrov 2013; Smith and Cognato 2013). Twenty-five species occur in the Nearctic region, including four invasive Palearctic species. Several Nearctic *Scolytus* species were described as the North American continent was explored. General entomologists including Say (1824), Riley (1867) and Walsh (1867) first described the eastern hardwood-feeders. LeConte (1868, 1876) described the most common California species and species native to the interior mountain ranges. The prominent scolytine taxonomists Swaine (1910, 1917), Blackman (1934), Wood (1962, 1967, 1968) and Bright (1964, 1972) also described several *Scolytus* species. *Scolytus californicus* LeConte (1868) was described from “California” but Blackman (1934) deemed this species a synonym of *Scolytus scolytus* (Fabricius, 1775). Blackman also suggested that the specimen given to LeConte might have been incorrectly labeled with the ‘California’ locality. This species has been intercepted to the United States on numerous occasions but has never become established (Blackman 1934; Wood 1982). *Scolytus scolytus* was therefore excluded from this monograph. Blackman (1934) and China (1962, 1963) provide a detailed account of the taxonomic history of *Scolytus* and outline the *Scolytus*/*Eccoptogaster* Herbst, 1793 controversy, which occurred from the late 1800’s to the early 1900’s that resulted from intense debate on whether Geoffroy’s (1762) description was valid. China (1962, 1963) outlines the International Commission on Zoological Nomenclature ruling that preserved the name *Scolytus* over *Eccoptogaster*.

Historically, there has been a great deal of uncertainty regarding the status of several *Scolytus* species, particularly: *Scolytus monticolae* and *Scolytus tsugae* (Swaine, 1917); *Scolytus abietis* Blackman, 1934 and *Scolytus opacus* Blackman, 1934; *Scolytus reflexus* and *Scolytus wickhami* Blackman, 1934; *Scolytus fiskei* Blackman, 1934 and *Scolytus unispinosus*. Blackman (1934) and Wood (1982) formally revised the Nearctic *Scolytus*. A third revision by Edson (1967) was a M.S. thesis but was never published. Wood (1982) synonymized several species described by Blackman including *Scolytus abietis*, *Scolytus wickhami* and *Scolytus fiskei*. However, Edson (1967) recognized these species based on morphological, ecological and geographical differences. One of these species, *Scolytus abietis* Blackman, was recently removed from synonymy because of such differences (Equihua-Martínez and Furniss 2009). *Scolytus* species are generally recognized based on differences in male morphology, specifically shape of the venter concavity or lack thereof, ascending angle and protrusion of the basal margin of the second abdominal ventrite, the placement and shape of the spine or lack thereof on the second ventrite, the presence or absence of a spine on the margin of ventrite 2 and 3 and frons aciculation and vestiture. Differences among species are generally subtle and substantial knowledge of the intraspecific variation is needed for accurate identifications. In addition, there is a paucity of diagnostic morphological characters for females; to date diagnostic characters are known only for females of hardwood-feeding species (Smith and Cognato 2010a). Association with a male or comparison to previously identified specimens is needed to determine the species of female specimens of conifer-feeders. Previous keys (LeConte 1876; Swaine 1918; Blackman 1934; Chamberlin 1939, 1958; Edson 1967; Bright and Stark 1973; Bright 1976; Wood 1982; Furniss and Johnson 2002) did not address the above deficiencies in species identification. In addition, previous authors were not explicit about their species concepts, raising doubts about species boundaries.

Unlike previous investigations (Blackman 1934; Edson 1967; Bright 1976; Wood 1982), this study incorporates molecular and morphological data in phylogenetic analyses, which provides a basis for assessing species boundaries. This is the first modern taxonomic treatment of any group of *Scolytus* species and will serve as the basis from which the rest of the genus will be revised. This regional monograph of the Nearctic *Scolytus* species provides a review of taxonomic characters, an assessment of their phylogenetic utility, an evaluation of intraspecific variation for each species, a key to both sexes and fully illustrates each species and diagnostic characters. We recognize 25 Nearctic *Scolytus* species.

## Materials

### Morphology

This revision is based upon examination of 4,996 adult ingroup specimens and 447 outgroup specimens. Specimens were obtained from field collecting events, reared from host material, or borrowed from North American and European collections. Palearctic species *Scolytus intricatus* (Ratzeburg, 1837), *Scolytus laevis* Chapuis, 1869, *Scolytus mali*, *Scolytus multistriatus*, *Scolytus pygmaeus* (Fabricius, 1787), *Scolytus ratzeburgii* E.W. Janson, 1856, *Scolytus rugulosus*, *Scolytus schevyrewi*, *Scolytus scolytus*, *Scolytus sinopiceus* Tsai, 1962 and *Scolytus sulcifrons* Rey, 1892 were selected as outgroups. *Scolytus propinquus* Blandford, 1896, a Neotropical species, was selected as the root taxon. These outgroup taxa were chosen based on the results of a large Scolytini phylogenetic analysis (Smith et al. in prep.) which found Neotropical *Scolytus* sister to Holarctic *Scolytus*. The following entomological collection abbreviations (most following Arnett et al. 1993) are referenced in the text. Names of the curators that prepared loans are listed in parentheses.

ANSP Academy of Natural Sciences, Philadelphia, Pennsylvania;

AMNH American Museum of Natural History, New York, New York (Lee Herman and Aaron Smith);

CASC California Academy of Sciences, San Francisco, California (David Kavanaugh);

CSCA California State Collection of Arthropods, Sacramento, California (Andrew Cline and Jacqueline Kishmirian);

CNCI Canadian National Collection of Insects, Ottawa, Ontario, Canada (Hume Douglas, and Patrice Bouchard);

CSUC C. P. Gillette Museum of Arthropod Biodiversity, Colorado State University, Fort Collins, Colorado (Boris Kondratieff);

CUIC Cornell University Insect Collection, Cornell University, Ithaca, New York (James Liebherr);

DEBC Donald E. Bright, Jr. Collection, Fort Collins, Colorado (Donald E. Bright, Jr.), to be housed at the CNCI;

EMEC Essig Museum of Entomology, University of California Berkeley, Berkeley, California (Cheryl Barr);

FMNH Field Museum of Natural History, Chicago, Illinois (James Boone);

FSCA Florida State Collection of Arthropods, Gainesville, Florida (Paul Skelley);

ISNB Institut Royal des Sciences Naturelles de Belgique, Brussels, Belgium (Pol Limbourg);

MCZC Museum of Comparative Zoology, Harvard University, Cambridge, Massachusetts (Philip Perkins);

MSUC Albert J. Cook Arthropod Research Collection, Michigan State University, East Lansing, Michigan (Gary Parsons);

NHMW Naturhistorisches Museum Wien, Vienna, Austria (Harald Schillhammer);

OSAC Oregon State Arthropod Collection, Oregon State University, Corvallis, Oregon (Christopher Marshall);

RJRC Robert J. Rabaglia Collection, Annapolis, Maryland (Robert J. Rabaglia);

SBMN Santa Barbara Museum of Nature, Santa Barbara, California (Michael Caterino);

SMEC Snow Entomological Museum, Lawrence, Kansas (Zachary Falin);

THAC Thomas H. Atkinson Collection, Austin, Texas (Thomas Atkinson);

WFBM William F. Barr Entomological Collection, University of Idaho, Moscow, Idaho (Frank Merickel);

UMMZ Museum of Zoology, University of Michigan, Ann Arbor, Michigan (Mark O’Brien);

USNM National Museum of Natural History, Smithsonian Institution, Washington, DC (Including Stephen L. Wood Collection) (Natalia Vandenberg);

ZIFH Zoologische Institut der Forsliche Hochschule, Eberswald, Germany;

ZMBN University Museum of Bergen, The Natural History Collections, Bergen, Norway (Bjarte Jordal).

Additional distribution records were compiled from the following publications: Blackman 1922, 1934; Doane et al. 1936; Edson 1967; Bright and Stark 1973; Bright 1976; Furniss and Carolin 1977; Furniss and Johnson 1987; Gast et al. 1989; Wood and Bright 1992; Cibrián Tovar et al. 1995; Furniss and Johnson 2002; Furniss et al. 2002; Lee et al. 2006; Majka et al. 2007a,b; Humble et al. 2010; Smith and Cognato 2010a; Furniss and Kegley 2011; Lee et al. 2011). Plant nomenclature was verified using the Missouri Botanical Garden’s Tropicos database (www.tropicos.org).

Specimens were examined using either a Leica (Wetzlar, Germany) MZ125 or MZ16 compound microscope and illuminated with a SCHOTT (Mainz, Germany) 150W halogen light source (model ACE®1). Images were taken with a Visionary Digital Passport II system (Palmyra, VA) using a Canon EOS 5D Mark II, 58.0 mm Canon Macro photo lens, Canon Speedlite transmitter ST-E2, two Canon Speedlite 4303X II flashes and a Stack Shot (Cognisys, Inc, Kingsley, MI). Montage images were assembled using Helicon Focus Mac Pro 4.2.8 (Helicon Soft, Kharkov, Ukraine). Measurements were made using an ocular micrometer on the same microscope and light source as listed above and calibrated with ROK (Shenzhen, China) 150.0 mm digital calipers (model DC-122A) following the protocol of Smith and Cognato (2010b). Measurements were taken from the specimen’s dorsal surface. Length was measured from the pronotum apex to the elytral apex. Width was measured at the widest part of the pronotum. Proportions are given as the ratio of length to width. A maximum of 50 specimens selected to encompass the known distribution were measured for each species. If important locality data such as state or county was missing from specimen labels, the information was inserted between square brackets in the material examined. Holotypes of *Scolytus sulcatus* and *Scolytus californicus* LeConte, 1868 were not physically examined. Type images were examined from the MCZC type database (http://insects.oeb.harvard.edu/mcz/) and the synonymy of these species was confirmed. *Scolytus californicus* was not included in this revision because it is a synonym of *Scolytus scolytus*, a species not established in the New World.

### Taxonomic characters

External anatomical terminology followed Hopkins (1909) subsequently used by Schedl (1931), Kaston (1936), Edson (1967) and Wood (1982, 1986, 2007). Sculpture terminology followed Torre-Bueno (1989). Several highly informative recently described Scolytini characters were also scored (Smith and Cognato 2010b). Provisional morphological homology was assessed by similarity and relative positions of characters.

Characters were scored from both sexes unless otherwise noted. *Scolytus* exhibits strong sexual dimorphism of the frons and abdominal venter; males display a wide array of morphological features, particularly on the venter, whereas females are more morphologically conserved. Consequently, characters were predominately male based. Male genitalia was found to be autapomorphic or extremely conserved in structure and thus characters were not scored. Characters and character state numbers correspond to data coded in the morphological data matrix for each taxon. The character matrix (Table 3) was constructed and edited using the online database MX (Yoder et al. 2006). Character transformations were evaluated using MacClade 4.0 PPC (Maddison and Maddison 2000) and homology of characters and definitions of characters states were re-examined and modified if necessary.

**Table 3.** Morphological character matrix of 43 characters for 37 species. Characters are described in Methods.

	Characters																																										
Species	1	2	3	4	5	6	7	8	9	10	11	12	13	14	15	16	17	18	19	20	21	22	23	24	25	26	27	28	29	30	31	32	33	34	35	36	37	38	39	40	41	42	43
*Scolytus abietis*	0	0	0	1	2	1	1	2	1	1	1	1	1	0	0	2	1	0	1	1	0	2	1	0	0	0	3	5	1	0	0	0	0	0	0	1	2	0	0	0	0	1	2
*Scolytus aztecus*	2	1	1	1	2	1	1	2	1	0	0	1	1	2	1	2	1	2	1	2	1	2	1	2	0	0	1	5	0	1	0	0	0	0	0	1	0	0	0	0	0	1	2
*Scolytus dentatus*	1	1	1	1	2	1	1	2	1	1	1	1	0	0	0	2	1	1	1	0	0	2	1	0	0	0	4	0	1	0	0	0	0	0	1	1	2	0	0	0	0	0	2
*Scolytus fagi*	0	0	1	1	2	2	1	2	1	0	1	1	1	1	0	2	1	2	0	0	0	1	0	0	0	0	1	0	3	0	0	0	0	0	0	0	0	1	1	0	0	?	8
*Scolytus fiskei*	0	1	1	1	2	1	1	2	1	1	1	0	1	1	0	1	1	1	1	2	0	1	0	1	1	0	0	3	0	0	0	0	0	0	0	3	1	0	0	0	0	0	1
*Scolytus hermosus*	0	0	0	1	2	1	1	2	1	0	1	1	1	0	0	2	1	1	1	2	0	2	1	1	0	0	3	0	1	0	0	0	0	0	0	1	0	0	0	0	0	1	2
*Scolytus laricis*	0	0	1	1	2	0	1	2	1	1	1	0	1	1	0	1	1	1	1	2	0	2	0	1	1	0	0	3	0	0	0	0	0	0	0	3	1	0	0	0	0	0	4
*Scolytus mali*	0	0	0	3	1	1	0	2	1	0	1	2	1	0	1	2	1	1	0	1	0	1	0	1	0	0	5	0	0	0	0	0	0	0	0	0	2	0	1	0	0	0	7
*Scolytus monticolae*	0	0	1	1	2	1	1	2	1	1	1	0	0	0	0	1	1	0	1	2	0	2	0	1	0	0	1	0	3	0	0	0	0	0	0	1	2	0	0	0	0	0	1
*Scolytus multistriatus*	0	0	1	1	2	0	1	2	0	0	1	2	1	1	1	2	1	1	1	0	0	1	1	1	2	0	5	1	3	1	0	1	1	1	0	1	0	0	0	0	0	0	6
*Scolytus mundus*	0	0	1	1	2	0	0	2	1	1	0	1	1	2	1	2	0	1	1	1	0	2	0	2	1	0	1	3	3	0	0	0	0	0	0	1	0	0	0	0	0	0	2
*Scolytus muticus*	1	0	1	2	2	0	2	0	0	0	1	1	1	2	1	0	0	2	1	0	0	1	0	2	0	0	5	0	3	0	0	0	0	0	0	0	0	1	1	1	0	1	9
*Scolytus obelus*	0	0	0	1	0	0	1	2	0	0	1	1	1	0	0	2	1	0	1	1	1	2	1	0	0	0	3	3	1	0	0	0	0	0	0	1	2	0	0	0	0	1	2
*Scolytus opacus*	0	0	0	1	2	1	1	2	1	1	1	1	1	0	0	2	1	0	1	1	0	2	1	0	0	0	3	5	1	0	0	0	0	0	0	1	2	0	0	0	0	1	2
*Scolytus oregoni*	0	0	1	1	2	0	1	2	1	1	1	0	1	0	0	2	1	1	1	1	0	2	1	0	0	0	2	0	1	0	0	0	0	0	0	1	2	0	0	0	0	0	1
*Scolytus piceae*	0	0	1	1	2	1	1	2	1	1	1	1	1	0	1	2	1	1	1	1	0	2	1	1	3	0	0	2	0	0	0	0	0	0	0	1	1	0	0	0	0	0	3
*Scolytus praeceps*	0	0	0	1	2	1	1	2	1	1	1	1	1	0	0	2	1	0	1	1	0	2	1	0	0	0	3	3	1	0	0	0	0	0	0	1	2	0	0	0	0	1	2
*Scolytus quadrispinosus*	0	1	2	2	2	0	2	0	0	0	1	1	1	1	1	1	1	2	0	0	1	2	1	0	0	0	4	5	2	0	0	1	1	1	1	2	1	0	0	0	0	0	A
*Scolytus reflexus*	1	1	1	1	2	1	1	2	1	0	1	1	1	0	1	2	1	1	1	2	0	1	0	2	0	0	0	0	0	1	0	0	0	0	0	2	1	0	0	0	0	0	1
*Scolytus robustus*	1	1	1	1	2	1	1	2	0	0	1	1	1	0	0	2	1	1	1	2	1	2	0	3	0	0	4	0	3	1	0	0	0	0	0	1	2	0	0	0	0	1	2
*Scolytus rugulosus*	0	0	0	1	2	1	0	2	0	0	1	0	0	2	0	0	0	0	0	3	1	2	1	2	0	0	0	0	0	1	0	0	0	0	0	1	0	0	1	0	0	0	7
*Scolytus schevyrewi*	0	0	2	2	2	1	0	0	0	0	1	1	0	1	0	0	0	1	0	1	0	1	1	1	2	0	5	1	0	0	0	0	0	0	0	1	0	0	0	0	0	0	6
*Scolytus silvaticus*	1	0	1	1	2	0	0	2	1	1	0	1	1	2	0	2	1	1	1	1	1	2	0	1	0	0	3	0	3	0	0	0	0	0	1	1	2	0	0	0	0	?	1
*Scolytus subscaber*	1	1	1	1	2	1	1	2	1	1	1	1	1	1	0	2	1	1	1	2	0	1	1	3	0	0	4	3	1	0	0	0	0	0	0	1	0	0	0	0	0	2	2
*Scolytus tsugae*	0	0	0	1	2	1	1	2	1	0	1	0	0	0	0	2	1	1	1	2	0	2	1	1	0	0	1	0	3	0	0	0	0	0	0	1	0	0	0	0	0	1	5
*Scolytus unispinosus*	0	0	1	1	2	1	0	2	1	0	1	0	1	1	0	1	1	1	1	2	0	1	1	1	1	0	0	3	0	0	0	0	0	0	0	1	2	0	0	0	0	0	1
*Scolytus ventralis*	0	0	0	1	2	1	1	2	1	1	1	1	1	0	0	2	1	1	1	2	0	1	1	3	0	0	1	3	3	0	0	0	0	0	0	1	1	0	0	0	0	1	2
*Scolytus virgatus*	0	1	1	1	2	1	1	2	1	0	1	1	1	0	1	2	1	1	1	2	0	1	0	2	0	0	0	0	0	1	0	0	0	0	0	2	1	0	0	0	0	?	1
*Scolytus wickhami*	0	1	1	1	2	1	1	2	1	0	1	1	1	0	1	2	1	1	1	2	0	1	0	2	0	0	0	0	0	1	0	0	0	0	0	2	1	0	0	0	0	0	1
*Scolytus intricatus*	1	0	1	1	1	1	0	2	0	0	1	1	0	2	0	1	0	0	0	0	0	2	1	1	0	0	5	0	0	0	0	0	0	0	0	1	0	0	0	0	0	1	8
*Scolytus laevis*	0	0	1	3	2	1	0	2	1	0	1	1	1	1	0	1	1	1	0	0	0	1	0	3	0	1	5	0	0	0	0	0	0	0	1	0	2	0	0	0	0	0	6
*Scolytus propinquus*	1	0	1	2	2	3	0	1	1	0	1	2	1	1	1	2	1	1	1	0	0	1	1	1	0	1	1	1	3	1	1	0	0	0	0	1	0	0	0	1	1	3	0
*Scolytus pygmaeus*	0	0	1	1	2	1	0	2	0	0	1	1	1	1	1	2	1	1	1	0	0	1	0	2	0	0	5	0	3	0	0	0	0	0	1	1	0	0	0	0	0	0	6
*Scolytus ratzeburgii*	1	0	1	1	2	0	0	2	1	0	1	1	1	0	0	2	1	1	0	1	0	0	0	1	0	0	5	0	3	0	0	0	0	0	1	0	2	1	1	0	0	0	B
*Scolytus scolytus*	0	0	1	1	0	2	0	2	2	1	1	1	1	0	0	2	1	1	0	0	0	0	1	2	0	0	5	0	0	0	0	0	0	0	1	1	1	1	0	0	0	0	6
*Scolytus sinopiceus*	2	0	2	2	2	1	0	2	1	0	1	0	0	2	0	1	1	0	0	3	1	0	0	2	0	0	0	0	0	1	0	0	0	0	0	1	2	0	0	0	0	0	3
*Scolytus sulcifrons*	0	0	0	?	1	2	0	2	2	?	1	0	1	0	1	2	1	1	1	0	0	1	0	3	0	0	5	0	0	0	0	0	0	0	1	1	1	1	0	0	0	0	6

Ecological characters including gallery pattern and host were scored. Character states were assigned based on a comprehensive literature review (e.g. Butovitsch 1929; Chamberlin 1939; Schedl 1948; Balachowsky 1949; Stark 1952; Chamberlin 1958; Edson 1967; Bright and Stark 1973; Michalski 1973; Bright 1976; Furniss and Carolin 1977; Wood 1982; Atkinson and Equihua-Martínez 1986; Wood and Bright 1992; Pfeffer 1994; Cibrián Tovar et al. 1995; Bright and Skidmore 1997, 2002; Furniss and Johnson 2002), and with field notes, and label data on pinned museum specimens.

**Figure 2.**
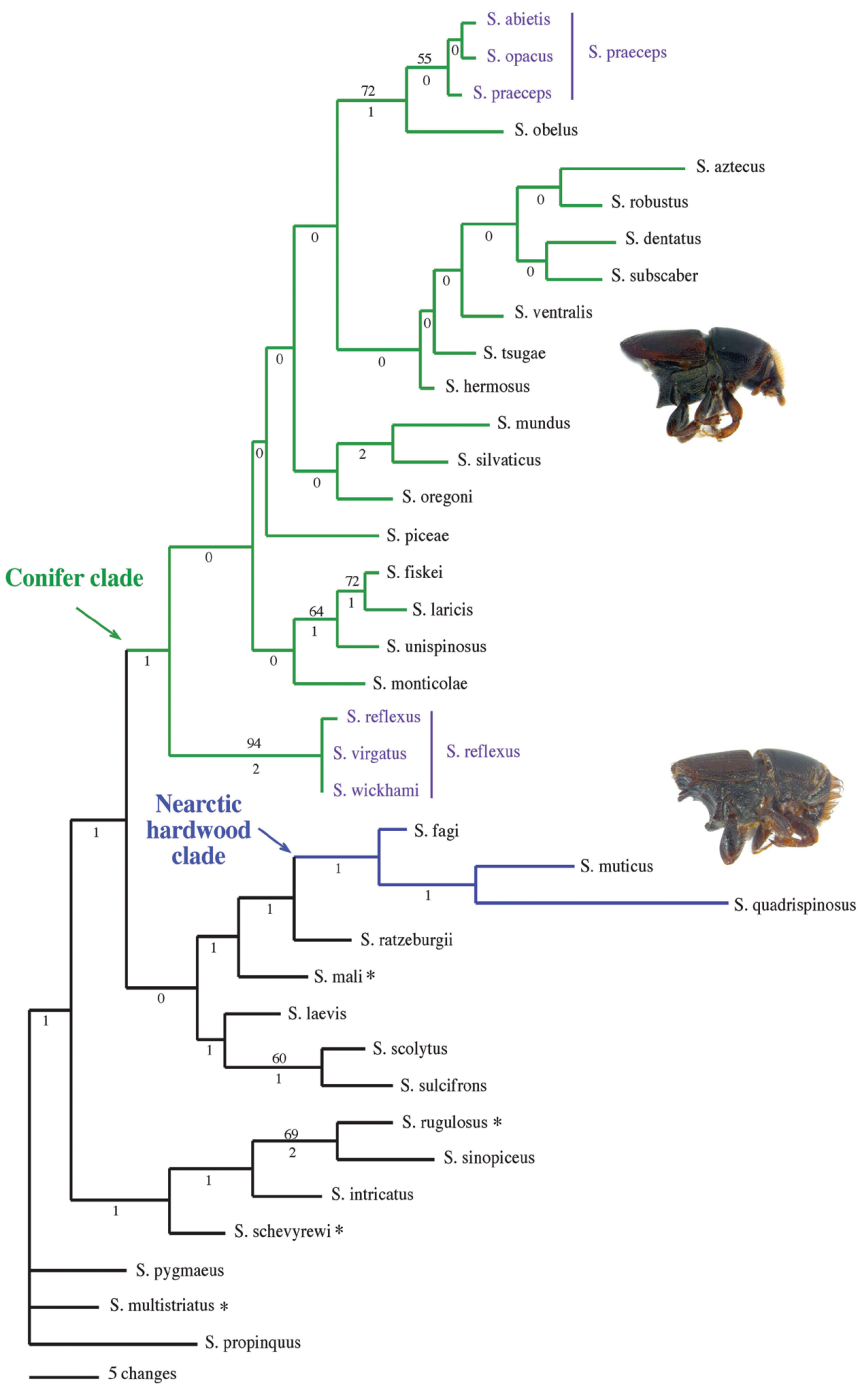
Morphological phylogeny of Nearctic *Scolytus*. Phylogram of one of 1016 most parsimonious trees (L = 262, CI = 0.332, RI = 0.539) generated for 37 taxa with 43 characters from a heuristic search of 1000 stepwise random additions with TBR in PAUP*. Numbers above the node indicate bootstrap values (>50). Bremer support values are listed below the node. The Nearctic hardwood and conifer clades are indicated in blue and green respectively. Non-monophyletic species are indicated in purple. * denotes species exotic to the Nearctic.

Forty-three characters were used in this study (19 binary and 24 multistate). Ten morphological characters were coded from the head, two from the thorax, ten from the elytra, one from the metepimeron, and 17 from the venter. Three ecological characters were also coded. Consistency and retention index values from the morphological phylogeny (Fig. 2) and generated from MacClade are listed next to each character. Characters and states were scored as follows:

1. *Epistomal emargination* (ci = 0.22; ri = 0.22): (0) weak; (1) moderate; (2) strong.
2. *Male epistomal process* (ci = 0.25; ri = 0.63): (0) absent; (1) present.
3. *Male frons shape* (ci = 0.25; ri = 0.45): (0) convex; (1) flat; (2) impressed.
4. *Male frons vestiture distribution* (ci = 0.33; ri = 0.20): (0) glabrous; (1) uniform; (2) predominately on margins; (3) epistomal region.
5. *Male frons vestiture length* (ci = 0.4; ri = 0.0): (0) less than width of eye; (1) equal to width of eye; (2) greater than width of eye.
6. *Male frons sculpturing* (ci = 0.38; ri = 0.50): (0) coarsely aciculate; (1) weakly aciculate; (2) rugose-reticulate; (3) smooth.
7. *Male frons punctures* (ci = 0.33; ri = 0.71): (0) small, fine; (1) small, coarse; (2) impunctate.
8. *Female frons shape* (ci = 0.67; ri = 0.50): (0) flat; (1) impressed; (2) convex; (3) excavated.
9. *Female frons sculpturing* (ci = 0.29; ri = 0.44): (0) coarsely aciculate; (1) finely aciculate; (2) reticulate.
10. *Female frons punctures* (ci = 0.14; ri = 0.54): (0) small, fine; (1) small, coarse.
11. *Pronotum length to width* (ci = 0.50; ri = 0.50): (0) as long as wide (0.95–1.05); (1) wider than long.
12. *Metepimeron length* (ci = 0.29; ri = 0.50): (0) less than half-length of metanepisternum; (1) half length of metanepisternum; (2) greater than half-length of metanepisternum.
13. *Elytral sides sub-parallel* (Michalski 1973) (ci = 0.25; ri = 0.50); (0) on basal half only; (1) on apical half only.
14. *Interstrial setae* (ci = 0.22; ri = 0.56); (0) glabrous; (1) sparse; (2) moderate.
15. *Interstrial impression* (ci = 0.13; ri = 0.42): (0) not impressed; (1) faintly impressed.
16. *Interstrial width* (ci = 0.29; ri = 0.44): (0) equal to striae; (1) twice width of striae; (2) more than twice the width of striae.
17. *Relative size of interstrial punctures* (ci = 0.25; ri = 0.25): (0) equal to strial; (1) smaller than strial.
18. *Strial impression* (ci = 0.40; ri = 0.70): (0) not impressed; (1) weakly impressed; (2) moderately impressed.
19. *Male elytral apex shape* (ci = 0.25; ri = 0.67): (0) rounded; (1) subquadrate.
20. *Elytral apex emargination* (ci = 0.38; ri = 0.76): (0) absent; (1) weak; (2) moderate; (3) strong.
21. *Apical margin of elytra serrate* (ci = 0.20; ri = 0.33): (0) absent; (1) present.
22. *Punctures on elytral apical margin* (ci = 0.20; ri = 0.53): (0) impunctate; (1) small, fine; (2) large, coarse.
23. *Venter appearance* (Blackman 1934) (ci = 0.11; ri = 0.50): (0) smooth, shining; (1) shagreened, dull.
24. *Venter setae length* (ci = 0.23; ri = 0.50): (0) less than 1 diameter of a puncture; (1) less than length of segment 3; (2) greater than length of segment 3; (3) glabrous.
25. *Female second ventrite* (ci = 0.60; ri = 0.50): (0) unarmed; (1) apical; (2) basal; (3) medial.
26. *Suture between first and second ventrites* (Wood 1982) (ci = 0.50; ri = 0.0): (0) clearly visible; (1) obsolete.
27. *Male first ventrite apical margin* (ci = 0.42; ri = 0.68): (0) rounded, on vertical surface; (1) weakly elevated; (2) lip; (3) weakly produced; (4) strongly produced; (5) flush, not on vertical surface.
28. *Male second ventrite armature* (ci = 0.36; ri = 0.42): (0) unarmed; (1) basal spine; (2) medial spine; (3) apical spine; (4) carina on basal half; (5) carina on apical half.
29. *Male second ventrite surface* (ci = 0.33; ri = 0.67): (0) convex; (1) weakly concave; (2) strongly concave; (3) flat.
30. *Second ventrite punctures* (ci = 0.25; ri = 0.63): (0) small, fine; (1) small, coarse.
31. *Male second ventrite setal tuft* (ci = 1.00; ri = 0.0): (0) absent; (1) present.
32. *Second ventrite lateral spines* (ci = 0.50; ri = 0.0): (0) absent; (1) present.
33. *Third ventrite lateral spines* (ci = 0.50; ri = 0.0): (0) absent; (1) present.
34. *Fourth ventrite lateral spines* (ci = 0.50; ri = 0.0): (0) absent; (1) present.
35. *Male fourth ventrite armed medially* (ci = 0.17; ri = 0.29): (0) absent; (1) present.
36. *Male fifth ventrite* (ci = 0.60; ri = 0.75): (0) unarmed – lacking carina; (1) midpoint of carina closer to apex; (2) midpoint of carina closer to base; (3) midpoint of carina equidistant from base and apex.
37. *Relative length of male fifth ventrite compared to third and fourth* (ci = 0.15; ri = 0.48): (0) 5 larger; (1) 3+4 longer; (2) equal.
38. *Male fifth ventrite setal patch* (ci = 0.33; ri = 0.50): (0) absent; (1) present.
39. *Male fifth ventrite with median depression* (ci = 0.33; ri = 0.50): (0) absent; (1) present.
40. *Male metatibial setae* (ci = 0.50; ri = 0.0): (0) not conspicuously longer than those of other tibiae; (1) much longer and more abundant than those of other tibiae.
41. *Mating system* (ci = 1.00; ri = 0.0): (0) monogamy; (1) polygamy.
42. *Gallery type* (ci = 0.50; ri = 0.70): (0) vertical; (1) transverse; (2) epsilon; (3) multi-branched.
43. *Host* (ci = 0.73; ri = 0.73): (0) Fabaceae; (1) *Pseudotsuga*; (2) *Abies*; (3) *Picea*; (4) *Larix*; (5) *Tsuga*; (6) Ulmaceae; (7) Rosaceae; (8) Fagaceae; (9) Cannabaceae; (A) Juglandaceae; (B) Betulaceae.

### Taxa, DNA sequences and alignment

We included 83 specimens (Table 4) representing 32 *Scolytus* species to reconstruct phylogenies using DNA sequences from mitochondrial and nuclear genes and morphology. The same Palearctic outgroup species used in the morphological analyses were included in the molecular dataset. As many Nearctic species as possible were included in the analysis. We were unable to collect fresh material for *Scolytus dentatus*, *Scolytus hermosus*, *Scolytus mundus* and *Scolytus silvaticus*, which were therefore excluded from the molecular analyses. Sequences of *Scolytus intricatus* and *Scolytus scolytus* were obtained from GenBank and included in the analyses. To assess mitochondrial cytochrome oxidase I (COI) intraspecific variation, we included 89 specimens (Table 4) representing 30 *Scolytus* species from as many populations as possible. Specimens were included in the four-gene phylogeny only if sequences were available for at least two genes.

**Table 4.** Table of *Scolytus* specimens sequenced, the collection locality, collector, host and GenBank accession numbers.

Species	Collection locality, date, collector and host	Matrix name	Extraction name	COI	28S	CAD	ArgK
*Scolytus abietis*	USA: California: Siskiyou Co.: Klamath National Forest, FR 41N16, N41°14.822', W122°53.562', 508ft, 28.VII.2010, S.M. Smith coll., Ex. *Abies concolor*	Sco abi 14	SMS 14	KJ909668	KJ923438	KJ923574	KJ923513
*Scolytus abietis*	USA: Idaho: Latah Co.: St. Joe National Forest, Moscow Mountain, N46°48.252', W116°49.811', 3834ft, S.M. Smith, A.R. Gillogly, M.M. Furniss coll., Ex. *Abies grandis*	Sco abi 15	SMS 15	KJ909669	-	-	-
*Scolytus abietis*	USA: Idaho: Bonner Co.: Kaniksu National Forest, FR 232, N48°20.609', W116°20.507', 2717ft, S.M. Smith, A.R. Gillogly coll., Ex. *Abies grandis*	Sco abi 16	SMS 16	KJ909670	KJ923439	KJ923575	KJ923514
*Scolytus abietis*	USA: Oregon: Deschutes Co.: Deschutes National Forest, Black Butte Rd, Black Butte, N40°24.924', W121°38.323', 4212ft, 1.VIII.2010, S.M. Smith coll., Ex. *Pseudotsuga menziesii*	Sco abi 17	SMS 17	KJ909671	-	-	-
*Scolytus aztecus*	Mexico: Nuevo Leon: San Antonio de las Alazanas: Santa Catarina, G. Cuellar coll., Ex. *Pseudotsuga menziesii*	Sco aze 161	SMS 161	-	KJ923440	KJ923576	KJ923515
*Scolytus fagi*	USA: Pennsylvania: Cumberland Co.: Roadway Dr @ Schenider Dr., 29.V.2009, L.R. Donovall coll., Ex. Lindgren funnel trap	Sco fagi 1	Sco fagi 1	KJ909672	KJ923441	-	-
*Scolytus fagi*	USA: Pennsylvania: Cumberland Co.: Roadway Dr @ Schenider Dr., 29.V.2009, L.R. Donovall coll., Ex. Lindgren funnel trap	Sco fagi 2	Sco fagi 2	KJ909673	KJ923442	KJ923577	KJ923516
*Scolytus fiskei*	USA: Colorado: Mesa Co.: Grand Mesa overlook, 8.VIII.2008, D.E. Bright, B.A. Barr coll., Ex. *Pseudotsuga menziesii* branches	Sco uni 1	Sco uni 1	KJ909736	KJ923496	KJ923625	KJ923561
*Scolytus fiskei*	USA: Colorado: Eastern Slope Rocky Mountains, 6.V.2010, D.E. Bright, B.A Barr, S.M. Smith coll., Ex. *Pseudotsuga menziesii*	Sco fis 36	SMS 36	KJ909737	-	-	-
*Scolytus fiskei*	USA: Colorado: Larimer Co.: Roosevelt National Forest, 9 km E Estes Park, Hwy 34, Big Thompson Canyon, N40°24.456', W105°24.565', 2143m, 5.V.2010, S.M. Smith, D.E. Bright, B.A. Barr coll., Ex. *Pseudotsuga menziesii*	Sco fis 37	SMS 37	KJ909738	-	-	-
*Scolytus fiskei*	USA: Idaho: Boise Co.: Boise National Forest, Bogus Basin, NF275, N43°44.347', W116°07.099', 6042ft, 8.VIII.2010, S.M. Smith, A.R. Gillogly coll., Ex. *Pseudotsuga menziesii*	Sco fisi 38	SMS 38	KJ909739	KJ923498	KJ923627	KJ923563
*Scolytus fiskei*	USA: Idaho: Latah Co.: Univeristy of Idaho experimental forest, N46°51.764', W116°43.786', 2855ft, 10.VIII.2010, S.M. Smith, A.R. Gillogly, M.M. Furniss coll., Ex. *Pseudotsuga menziesii*	Sco fis 41	SMS 41	KJ909742	KJ923501	KJ923630	KJ923566
*Scolytus intricatus*	Sweden: Smaaland: Oskarshamn [GenBank]	Sco int BJ	N/A	HQ883677	HQ883589	HQ883820	HQ883909
*Scolytus intricatus*	Sweden: Smaaland: Karlsburg, 15.VII.2005, B.H. Jordal coll., Ex. *Quercus* sp.	Sco int 64	SMS 64	-	KJ923444	KJ923579	KJ923518
*Scolytus laevis*	Denmark: Rødby Havn., 24.VIII.2005, J. Pedersen coll.	Sco laevis	Sco laevis	-	KJ923445	KJ923580	KJ923519
*Scolytus laricis*	USA: Idaho: Boundary Co.: Kaniksu National Forest, Robinson Lake campground, N48°58.385', W116°13.351', 2748ft, 11.VIII.2010, S.M. Smith, A.R. Gillogly coll., Ex. *Larix occidentalis*	Sco lar 2	SMS 2	KJ909676	-	-	-
*Scolytus laricis*	USA: Idaho: Boundary Co.: Kaniksu National Forest, Robinson Lake campground, N48°58.197', W116°13.068', 2748ft, 11.VIII.2010, S.M. Smith, A.R. Gillogly coll., Ex. *Larix occidentalis*	Sco lar 12	SMS 12	KJ909677	KJ923446	KJ923581	KJ923520
*Scolytus laricis*	USA: Idaho: Boundary Co.: Kaniksu National Forest, N48°39.185', W116°32.662', 5311ft, 12.VIII.2010, S.M. Smith, A.R. Gillogly coll., Ex. *Larix occidentalis*	Sco lar 13	SMS 13	KJ909678	-	-	-
*Scolytus mali*	USA: Pennsylvania: Lebanon Co.: Mt. Gretna, N40.242501 W76.462406, IV-VIII.2009, S.E. Spichiger coll., Ex. Lindgren funnel trap	Sco mal 1	Sco mal 1	-	KJ923447	KJ923582	KJ923521
*Scolytus mali*	Czech Republic: South Bohemia: Jindřichův Hradec env. Číměř; 23.VII.2011, M.Knížek lgt., Ex. *Prunus* sp.	Sco mal 78	SMS 78	KJ909679	KJ923448	KJ923583	KJ923522
*Scolytus mali*	USA: Michigan: Kalamazoo Co.: Gourdneck Lake State Game Area, 6.VIII.2011, S.M. Smith, A.I. Cognato coll., Ex. *Prunus* sp.	Sco mal 81	SMS 81	KJ909680	KJ923449	KJ923584	KJ923523
*Scolytus monticolae*	USA: Idaho: Boundary Co.: Kaniksu National Forest, Robinson Lake campground, N48°58.385', W116°13.351', 2748ft, 11.VIII.2010, S.M. Smith, A.R. Gillogly coll., Ex. *Pseudotsuga menziesii*	Sco mon 33	SMS 33	KJ909681	-	-	-
*Scolytus monticolae*	USA: Idaho: Soshone Co.: Coeur D’Alene National Forest, Placer Creek Rd N47°25.746', W115°53.601', 3547ft, 15.VIII.2010, S.M. Smith, A.R. Gillogly coll., Ex. *Pseudotsuga menziesii*	Sco mon 34	SMS 34	KJ909682	KJ923450	-	-
*Scolytus monticolae*	USA: Idaho: Boise Co.: Boise Natioanl Forest, Bogus Basin, NF275, N43°44.347', W116°07.099', 6042ft, 8.VIII.2010, S.M. Smith, A.R. Gillogly coll., Ex. *Pseudotsuga menziesii*	Sco mon 35	SMS 35	KJ909683	KJ923451		KJ923524
*Scolytus multistriatus*	Denmark: Storstrøm: Rødbyhavn, 24.VIII.2005, J. Pedersen coll.	Sco mul 65	SMS 65	KJ909685	KJ923453	KJ923586	KJ923525
*Scolytus multistriatus*	USA: Michigan: Kalamazoo Co.: Gourdneck Lake State Game Area, 19.VI.2011, A.I. Cognato coll., Ex. *Ulmus* sp.	Sco mul 82	SMS 82	KJ909686	KJ923454	KJ923587	-
*Scolytus multistriatus*	Russia: Moscow Oblast: Dolgoprudnly, N55°58.266', E37°30.191’ 570m, 28.VII.2011, A.V. Petrov, Ex. *Ulmus laevis*	Sco mul 147	SMS 147	KJ909687	KJ923455	KJ923588	-
*Scolytus muticus*	USA: Pennsylvania: Cumberland Co.: Roadway Dr @ Schenider Dr., 29.V.2009, L.R. Donovall coll., Ex. Lindgren funnel trap	Sco mut 1	Sco mut 1	KJ909688	KJ923456	KJ923589	KJ923526
*Scolytus muticus*	USA: Michigan: Wayne Co.: Grosse Ile, N42.17060 W83.14496, 7-20.VI.2007, R. Mech coll., Ex. Lindgren funnel trap	Sco mut 2	Sco mut 2	KJ909689	KJ923457	-	-
*Scolytus muticus*	USA: Pennsylvania: Dauphin Co.: Harrisburg, N40.316325 W76.888783, IV-VIII.2009, S.E. Spichiger coll., Ex. Lindgren Funnel trap	Sco mut 4	Sco mut 4	KJ909690	-	-	-
*Scolytus muticus*	USA: South Carolina: Columbia, 26.X.2011, W. Jones coll., Ex. *Celtis* sp.	Sco mut 169	SMS 169	KJ909691	KJ923458	KJ923590	KJ923527
*Scolytus obelus*	USA: Arizona: Cochise Co.: Coronado National Forest, Chiricahua Mountains, N31°55.987', W109°16.331', 7022ft, 22.V.2010, S.M. Smith coll. Ex. *Abies concolor*	Sco obe 18	SMS 18	KJ909692	KJ923459	KJ923591	KJ923528
*Scolytus obelus*	USA: Arizona: Graham Co.: Coronado National Forest, Pinaleno Mountains, N32°37.702', W109°49.472', 7896ft, 23.V.2010, S.M. Smith coll. Ex. *Abies concolor*	Sco obe 19	SMS 19	KJ909693	-	-	-
*Scolytus obelus*	USA: Arizona: Coconino Co.: Arizona Snowbowl, N35°19.593', W111°42.681', 9032ft, 27.V.2010 S.M. Smith coll., Ex. *Abies lasiocarpa*	Sco obe 20	SMS 20	KJ909694	-	-	-
*Scolytus obelus*	USA: New Mexico: Torrance Co.: Cibola National Forest, W. Manzano, N34°37.226', W106°24.752', 7999ft, 11.V.2010, S.M. Smith, A.I. Cognato coll., Ex. *Abies concolor*	Sco obe 44	SMS 44	KJ909695	KJ923460	KJ923592	KJ923529
*Scolytus opacus*	USA: Idaho: Latah Co.: Univeristy of Idaho experimental forest, N46°51.372', W116°44.038', 2857ft, 10.VIII.2010, S.M. Smith, A.R. Gillogly, M.M. Furniss, Ex. *Abies lasiocarpa*	Sco opa 21	SMS 21	KJ909696	KJ923461	KJ923593	KJ923530
*Scolytus opacus*	USA: Idaho: Boundary Co.: Kaniksu National Forest, Robinson Lake campground, N48°58.200', W116°13.067', 2696ft 13.VIII.2010, S.M. Smith, A.R. Gillogly, Ex. *Abies lasiocarpa*	Sco opa 68	SMS 68	KJ909697	KJ923462	-	-
*Scolytus oregoni*	USA: Oregon: Jackson Co.: Rogue River National Forest, Rogue River gorge viewpoint, Hwy 62 1/4 mi N Union Creek Rd, N42°54.540', W122°26.733', 3489ft, 21.VIII.2010, S.M. Smith coll. Ex. *Pseudotsuga menziesii*	Sco ore 5	SMS 5	-	KJ923463	KJ923594	KJ923531
*Scolytus oregoni*	USA: Oregon: Jackson Co.: Rogue River National Forest, Rogue River gorge viewpoint, Hwy 62 1/4 mi N Union Creek Rd, N42°54.540', W122°26.733', 3489ft, 21.VIII.2010, S.M. Smith coll. Ex. *Pseudotsuga menziesii*	Sco ore 71	SMS 71	KJ909698	KJ923464	KJ923595	KJ923532
*Scolytus piceae*	USA: Michigan: Livingston Co.: Howell, 431 Bishop Rd, N42.5076 W83.85698, 25.VI.2009, R. Mech coll., Ex. Lindgren funnel trap	Sco pic 1	Sco mul 1	KJ909700	-	-	-
*Scolytus piceae*	USA: Montana: Jefferson Co.: highway 15 N. of Butte, N46.2075 W112.3360, 6013ft, 24.VII.2004, K.P. Dole coll., Ex. *Picea* sp.	Sco pic	Sco pic 1	KJ909699	KJ923465	KJ923596	
*Scolytus piceae*	USA: Idaho: Latah Co.: University of Idaho Experimental Forest, N46°51.164', W116°44.838', 2939ft, S.M. Smith, A.R. Gillogly, M.M. Furniss coll., Ex. *Picea engelmanni*	Sco pic 7	SMS 7	KJ909701	KJ923466	KJ923597	KJ923533
*Scolytus piceae*	USA: Idaho: Valley Co.: Boise National Forest, Hwy 55, S. of Donnelly, N44°20.117', W116°02.698', 4816ft, 17.VIII.2010, S.M. Smith, A.R. Gillogly, Ex. *Picea engelmannii*	Sco pic 8	SMS 8	KJ909702	-	-	-
*Scolytus piceae*	USA: South Dakota: Lawrence Co.: Brownsville Rd near Leads, N44.2922 W103.7828, 27.VII.2004, 5650ft, K.P. Dole coll., Ex. *Picea glauca*	Sco pic 73	SMS 73	KJ909703	KJ923467	KJ923598	KJ923534
*Scolytus piceae*	USA: Wyoming: Carbon Co.: Medicine Bow National Forest, Snowy Mountains, WY130, Lake Marie, N41°19.965', W106°19.516’ 3208m, 26.VII.2011, S.M. Smith, D.E. Bright, B.A Barr coll., Emerged 1-5.IX.2011, Ex. *Picea engelmannii* branches	Sco pic 83	SMS 83	KJ909704	KJ923468	KJ923599	KJ923535
*Scolytus praeceps*	USA: California: El Dorado Co.: El Dorado National Forest, Ise house resevoir, N38.50 W 120.22, 1653m, 17.VI.2003, A.I. Cognato coll., Ex. *Abies* sp.	Sco pra	Sco pra	KJ909709	-	-	-
*Scolytus praeceps*	USA: California: Alpine Co.: Toiyabe National Forest, 11.6 miles E of Markleeville, N38°39.906', W119°38.540', 24.VII.2010, S.M. Smith coll., Ex. *Abies magnifica*	Sco pra 28	SMS 28	KJ909705	-	-	-
*Scolytus praeceps*	USA: California: El Dorado Co.: El Dorado National Forest, nr. Ice House Resevoir N38°50.002', W120°21.160', 5413ft, 25.VII.2010, S.M. Smith coll., Ex. *Abies concolor*	Sco pra 29	SMS 29	KJ909706	KJ923469	KJ923600	KJ923536
*Scolytus praeceps*	USA: California: Siskiyou Co.: Shasta Trinity National Forest, Mt. Shasta, N41°20.844', W122°16.691', 4892ft, S.M. Smith coll., Ex. *Abies concolor*	Sco pra 30	SMS 30	KJ909707	KJ923470	KJ923601	KJ923537
*Scolytus praeceps*	USA: California: Siskiyou Co.: Klamath National Forest, FR 41N16, N41°14.822', W122°53.562', 5081ft, 28.VII.2010, S.M. Smith coll., Ex. *Abies concolor*	Sco pra 31	SMS 31	KJ909708	-	-	-
*Scolytus praeceps*	USA: Wyoming: Albany Co.: Snowy Mountains, Medicine Bow National Forest, Spruce campground, 6.IX.2010, D.E. Bright, B.A. Barr coll., Ex. *Abies concolor*	Sco pra 91	SMS 91	KJ909710	KJ923471	KJ923602	KJ923538
*Scolytus propinquus*	Mexico: Oaxaca: Huatulco, 15.76234, -96.12885, 41m, 23.VI.2009, T.H. Atkinson coll., THA 874, Ex. legume tree	Sco pro 1	Sco pro 1	KJ909711	KJ923472	KJ923603	KJ923539
*Scolytus propinquus*	Mexico: Oaxaca: Huatulco, 15.76234, -96.12885, 41m, 23.VI.2009, T.H. Atkinson coll., THA 874, Ex. legume tree	Sco pro 2	Sco pro 2	KJ909712		KJ923604	KJ923540
*Scolytus pygmaeus*	Czech Republic: Moravia: Břeclav Kamci obora, 2004, K. Novakova coll.	Sco pyg 62	SMS 62	KJ909713	KJ923473	KJ923605	KJ923541
*Scolytus pygmaeus*	Denmark: Storstrøm: Rødbyhavn, 24.VIII.2005, J. Pedersen coll.	Sco pyg 67	SMS 67	KJ909714	KJ923474	KJ923606	KJ923542
*Scolytus quadrispinosus*	USA: Pennsylvania: Cumberland Co.: Roadway Dr @ Schenider Dr., 24.VII.2009, L.R. Donovall coll., Ex. Lindgren funnel trap	Sco qua 1	Sco qua 1	KJ909715	KJ923475	KJ923607	KJ923543
*Scolytus quadrispinosus*	USA: Pennsylvania: Cumberland Co.: Roadway Dr @ Schenider Dr., 24.VII.2009, L.R. Donovall coll., Ex. Lindgren funnel trap	Sco qua 2	Sco qua 2	KJ909716	-	-	-
*Scolytus quadrispinosus*	USA: Pennsylvania: Lebanon Co.: Mt. Gretna, N40.242501 W76.462406, IV-VIII.2009, S.E. Spichiger coll., Ex. Lindgren funnel trap	Sco qua 3	Sco qua 3	KJ909717	-	-	-
*Scolytus quadrispinosus*	USA: Maryland: Ann Aruridel Co.: Annapolis, 26.V.2012, R.J. Rabaglia coll., Ex. Lindgren funnel trap	Sco qua 170	SMS 170	KJ909718	KJ923476	KJ923608	KJ923544
*Scolytus ratzeburgii*	Russia: Primorsky: Anisimovka, 12.VII.2008, B.H. Jordal coll.	Sco ratz 60	SMS 60	KJ909719	KJ923477	KJ923609	KJ923545
*Scolytus reflexus*	USA: Arizona: Pima Co.: Coronado National Forest, Santa Catalina Mountains, N32°24.529', W110°42.678', 7869ft, 22.V.2010, S.M. Smith coll. Ex. *Pseudotsuga menziesii*	Sco ref 26	SMS 26	KJ909720	KJ923478	KJ923610	-
*Scolytus reflexus*	USA: Arizona: Cochise Co.: Coronado National Forest, Chiricahua Mountains, N31°54.915', W109°16.040', 8196ft, 20.V.2010, S.M. Smith coll. Ex. *Pseudotsuga menziesii*	Sco ref 27	SMS 27	KJ909721	KJ923479	KJ923611	-
*Scolytus reflexus*	USA: Colorado: Larimer Co.: Roosevelt National Forest, 9 km E Estes Park, Hwy 34, Big Thompson Canyon, N40°24.456', W105°24.565', 2143m, 5.V.2010, S.M. Smith, D.E. Bright, B.A. Barr coll. Ex. *Pseudotsuga menziesii*	Sco ref 32	SMS 32	KJ909722	KJ923480	KJ923612	KJ923546
*Scolytus reflexus*	USA: Colorado: Boulder Co.: Roosevelt National Forest, St. Vrain canyon, N40°10.072', W105°23.623', 2127m, 24.VII.2011, S.M. Smith, D.E. Bright, B.A. Barr coll., Ex. *Pseudotsuga menziesii*	Sco ref 85	SMS 85	KJ909684	KJ923452	KJ923585	-
*Scolytus robustus*	USA: Arizona: Graham Co.: Coronado National Forest, Pinaleno Mountains, N32°37.702', W109°49.472', 7896ft, 23.V.2010, S.M. Smith coll. Ex. *Abies concolor*	Sco rob 3	SMS 3	KJ909723	KJ923481	KJ923613	KJ923547
*Scolytus robustus*	USA: New Mexico: Taos Co.: Carson National Forest, Agua Piedra campground, Hwy 75, N36°07.960', W105°31.828’ 8477 ft, 13.V.2010, S.M. Smith, A.I. Cognato coll., Ex. *Abies concolor*	Sco rob 43	SMS 43	KJ909724	KJ923482	KJ923614	KJ923548
*Scolytus robustus*	USA: Arizona: Cochise Co.: Coronado National Forest, Chiricahua Mountains, N31°54.665', W109°16.336', 2445m, 5.VIII.2012, S.M. Smith, A.I. Cognato coll., Ex. *Abies concolor*	Sco rob 178	SMS 178	KJ909725	KJ923483	KJ923615	KJ923549
*Scolytus rugulosus*	Hungary: Győr-Moson-Sopron: Sopron, 2003, F. Lakatos coll.	Sco rug 66	SMS 66	-	KJ923484		KJ923550
*Scolytus rugulosus*	Morocco: 30 km S. Asni, Tizi’n’Test, 18.IV.2002, B.H. Jordal coll,. Ex. *Prunus dulcis*	Sco rug 72	SMS 72	-	KJ923485	KJ923616	KJ923551
*Scolytus rugulosus*	Czech Republic: South Bohemia, Jindřichův Hradec env. Číměř; 23.VII.2011, M.Knížek coll., Ex. *Prunus* sp.	Sco rug 79	SMS 79	KJ909726	KJ923486	KJ923617	KJ923552
*Scolytus rugulosus*	Iran: Guilan: Khoshkestalkh, N37°27'01” E49°42'06", 17.VII.2011, S. Amini coll., Ex. *Malus* sp.	Sco rug 165	SMS 165	-	KJ923487	KJ923618	KJ923553
*Scolytus schevyrewi*	USA: Missouri: Saint Louis Co.: Maryland Heights, 22.V.2008, Ex. Lindgren funnel trap	Sco sch 2	Sco sch 2	-	KJ923488	KJ923619	KJ923554
*Scolytus scolytus*	Denmark: NEJ, Tofte Skov [GenBank]	Sco sco BJ	N/A	HQ883678	HQ883590	HQ883821	HQ883910
*Scolytus sinopiceus*	China: Qinghai: Mai Xiu Forest Preserve, N35°16.288', E101°55.904', 2927m, 21.V.2008, A.I. Cognato coll., Ex. Lindgren funnel trap	Sco sin 1	Sco sin 1	KJ909728	KJ923489	KJ923620	KJ923555
*Scolytus sinopiceus*	China: Sichuan: Highway 213 near Zhangla, 9.VII.2004, A.I. Cognato coll., Ex. *Picea purpurea*	Sco sin 70	SMS 70	KJ909729	KJ923490	-	KJ923556
*Scolytus subscaber*	USA: California: Alpine Co.: Toiyabe National Forest, 11.6 miles E of Markleeville, N38°39.906', W119°38.540', 24.VII.2010, S.M. Smith coll., Ex. *Abies magnifica*	Sco sub 6	SMS 6	KJ909730	KJ923491	KJ923621	KJ923557
*Scolytus subscaber*	USA: Idaho: Boundary Co.: Kaniksu National Forest, Robinson Lake campground, N48°58.197', W116°13.068', 2748ft 13.VIII.2010, S.M. Smith, A.R. Gillogly, Ex. *Abies lasiocarpa*	Sco sub 51	SMS 51	KJ909731	KJ923492	-	KJ923558
*Scolytus sulcifrons*	Russia: Moscow Oblast: Dolgoprudnly, N55°58.266', E37°30.191', 570m, 28.VII.2011, A.V. Petrov coll., Ex. *Ulmus laevis*	Sco sul 146	SMS 146	KJ909732	KJ923493	KJ923622	KJ923559
*Scolytus tsugae*	USA: Oregon: Hood River Co.: Mount Hood National Forest, Sherwood Campground, Hwy 35, N45°19.278', W121°37.104', 4293ft, 2.VIII.2010, S.M. Smith coll., Ex. *Tsuga heterophylla*	Sco tsu 9	SMS 9	KJ909733	-	-	-
*Scolytus tsugae*	USA: Idaho: Soshone Co.: St. Joe National Forest, N47°00.790', W116°12.359', 3192 ft, 15.VIII.2010, S.M. Smith, A.R. Gillogly coll., Ex. *Tsuga heterophylla*	Sco tsu 10	SMS 10	KJ909734	KJ923494	KJ923623	-
*Scolytus tsugae*	USA: Idaho: Boundary Co.: Kaniksu National Forest, N48°40.911', W116°34.345', 4353ft, 12.VIII.2010, S.M. Smith, A.R. Gillogly coll., Ex. *Tsuga heterophylla*	Sco tsu 11	SMS 11	KJ909735	KJ923495	KJ923624	KJ923560
*Scolytus unispinosus*	USA: Oregon: Deschutes Co.: Deschutes National Forest, Black Butte Rd, Black Butte, N40°24.924', W121°38.323', 4212ft, 1.VIII.2010 S.M. Smith coll., Ex. *Pseudotsuga menziesii*	Sco fis 1	SMS 1	KJ909674	KJ923443	KJ923578	KJ923517
*Scolytus unispinosus*	USA: Oregon: Hood River Co.: Mount Hood National Forest, Sherwood Campground, Hwy 35, N45°19.278', W121°37.104', 4293ft, 2.VIII.2010, S.M. Smith coll. Ex. *Pseudotsuga menziesii*	Sco uni 39	SMS 39	KJ909740	KJ923499	KJ923628	KJ923564
*Scolytus unispinosus*	USA: Oregon: Jackson Co.: Rogue River National Forest, NF 60, N42°53.926', W122°19.177', 4547ft, 30.VII.2010, S.M. Smith coll. Ex. *Pseudotsuga menziesii*	Sco uni 40	SMS 40	KJ909741	KJ923500	KJ923629	KJ923565
*Scolytus unispinosus*	USA: Oregon: Klamath Co.: Deschutes National Forest, NF 4672, N43°30.474', W121°52.147', 4923ft, 31.VII.2010, S.M. Smith coll. Ex. *Pseudotsuga menziesii*	Sco uni 42	SMS 42	KJ909743	KJ923502	KJ923631	KJ923567
*Scolytus unispinosus*	USA: Oregon: Marion Co.: Williamette National Forest,Breitenbush Rd, N44°47.200', W121°56.557', 2645ft, 1.VIII.2010, S.M. Smith coll., Ex. *Pseudotsuga menziesii*	Sco uni 151	SMS 151	KJ909744	KJ923503	KJ923632	KJ923568
*Scolytus ventralis*	USA: Arizona: Cochise Co.: Coronado National Forest, Chiricahua Mountains, 14.VII.2009, J. Hulcr coll., Ex. *Pseudotsuga menziesii*	Sco ven 1	Sco sp 1	KJ909745	-	-	-
*Scolytus ventralis*	USA: Idaho: Boundary Co.: Kaniksu National Forest, Robinson Lake campground, N48°58.385', W116°13.351', 2748ft, 11.VIII.2010, S.M. Smith, A.R. Gillogly coll. Ex. *Abies lasiocarpa*	Sco ven 22	SMS 22	KJ909746	KJ923504	KJ923633	KJ923569
*Scolytus ventralis*	USA: Idaho: Adams Co.: Payette National Forest N45°00.225', W116°09.651', 6025ft, 17.VIII.2010, S.M. Smith, A.R. Gillogly coll., Ex. *Abies grandis*	Sco ven 45	SMS 45	KJ909747	-	-	-
*Scolytus ventralis*	USA: Idaho: Benewah Co.: McCroskey State Park, N47°04.801', W116°54.960', 3744ft,11.VIII.2010, S.M. Smith, A.R. Gillogly coll., Ex. *Abies grandis*	Sco ven 46	SMS 46	KJ909748	KJ923505	-	-
*Scolytus ventralis*	USA: Idaho: Boundary Co.: Kaniksu National Forest, Robinson Lake campground, N48°58.200', W116°13.067', 2696ft 13.VIII.2010, S.M. Smith, A.R. Gillogly, Ex. *Abies grandis*	Sco ven 47	SMS 47	KJ909749	-	-	-
*Scolytus ventralis*	USA: New Mexico: Torrance Co.: Cibola National Forest, W. Manzano, N34°37.325', W106°24.642', 8026ft, 11.V.2010, S.M. Smith, A.I. Cognato coll., Ex. *Abies concolor*	Sco ven 48	SMS 48	KJ909750	KJ923506	KJ923634	KJ923570
*Scolytus ventralis*	USA: California: El Dorado Co.: El Dorado National Forest, nr. Ice House Resevoir N38°50.002', W120°21.160', 5413ft, 25.VII.2010, S.M. Smith coll., Ex. *Abies concolor*	Sco ven 49	SMS 49	KJ909751	KJ923507	KJ923635	KJ923571
*Scolytus ventralis*	USA: Arizona: Coconino Co.: Arizona Snowbowl, N36°24.381', W112°05.619', 8923ft, 30.V.2010 S.M. Smith, K. Bush coll., Ex. *Abies lasiocarpa*	Sco ven 50	SMS 50	KJ909752	KJ923508	KJ923636	-
*Scolytus virgatus*	Mexico: Nuevo Leon: San Antonio de las Alazanas: Santa Catarina, G. Cuellar coll., Ex. *Pseudotsuga menziesii*	Sco vir 162	SMS 162	-	KJ923509	KJ923637	-
*Scolytus wickhami*	USA: Arizona: Cochise Co.: Coronado National Forest, Chiricahua Mountains, N31°55.360', W109°15.702', 7882ft, 20.V.2010, S.M. Smith coll. Ex. *Pseudotsuga menziesii*	Sco wick 24	SMS 24	KJ909753	KJ923510	KJ923638	-
*Scolytus wickhami*	USA: New Mexico: Otero Co.: Lincoln National Forest, Apache Point observatory, N32°47.046', W105°48.841', 9116ft, 16.V.2010, S.M. Smith coll., Ex. *Pseudotsuga menziesii*	Sco wick 25	SMS 25	KJ909754	KJ923511	KJ923639	KJ923572
*Scolytus wickhami*	USA: New Mexico: Colfax Co.: Highway 64 near Ute Park, N36°33'9.26” W105°07'6.74", 2242m, 24.VI.2004, A.I. Cognato, S.A. Stephens coll., Ex. *Pinus ponderosa*	Sco wick 74	SMS 74	KJ909755	KJ923512	KJ923640	KJ923573

DNA was extracted from freshly collected specimens preserved in 200 proof ethanol and from pinned specimens killed in sawdust impregnated with ethyl acetate and immediately pinned. Specimens were dissected by removing the head and thorax from the abdomen. DNA extractions were performed on the head and thorax using a Qiagen DNEasy blood and tissue kit (Hilden, Germany) following manufacturer protocols except for the DNA elution procedure, which consisted of a single elution of 100–200 µl of buffer AE depending on the size of the specimen, with specimens measuring less than 5.0 mm in length receiving 100 µl and those greater than 5.0 mm in length receiving 200 µl. After the extraction process was completed, the body parts were rinsed in 70% ethanol, glued onto a mounting board, pinned, labeled and were vouchered in the MSUC. The resulting purified DNA was used to amplify partial gene regions of COI, D2 region of nuclear ribosomal 28S, CAD, and Argenine Kinase (ArgK) using the PCR primers listed in Table 5. The COI barcoding primers LCO 1490 and HCO 2198 did not consistently amplify scolytine COI sequences, which necessitated the construction of the degenerate scolytine specific primers, 1495b and rev750. These primers worked best for *Scolytus* specimens and have proven to be effective on a broad array of scolytine taxa (Cognato et al. unpublished). The 1495b and rev750 primers were used to create the *Scolytus* specific F215 and Rev453 primers, respectively for 5–20 years or older pinned specimens. All PCR cocktails consisted of a total volume of 25 µl and included 14.25–17.25 µl ddH_2_O, 2.5 µl 10X PCR buffer (Qiagen), 1.0 µl 25 mM MgCl_2_ (Qiagen), 0.5µl dNTP mix (Qiagen), 2–5 µl DNA temfig, 0.25 µl HotStar Taq DNA polymerase (Qiagen). PCR reactions were performed on a thermal cycler (PTC-2000, MJ Research, Waltham, MA, USA or MyCylcer Thermocycler, BioRad, Hercules, CA, USA) under the following conditions: one cycle for 15 min at 95 °C, 40 cycles for 30 (COI)–45 (28S, CAD, ArgK) sec at 94 °C, 45 sec at 50–58 °C (see Table 5 for specific annealing temperatures), 1 min at 72 °C and a final elongation cycle of 5 min at 72 °C. PCR products were cleaned using ExoSAP-IT (USB Corp., Cleveland, OH, USA) and following the manufacturer protocols. Cleaned PCR products were then prepared for sequencing. Each reaction contained 3.5 µl of cleaned PCR product, 1 µl 33 pM/µl sequencing primers (identical to those used in PCR), and 7.5 µl of ddH_2_O. Samples were sequenced in the Michigan State University Research Technology Support Facility using a BigDye Terminator v 1.1 (Applied Biosystems, Foster City, CA, USA) and visualized using an ABI 3730 Genetic Analyzer (Applied Biosystems).

**Table 5.** Table of PCR primers and the annealing temperatures used for the amplification of gene sequences.

Gene	Primer	Primer sequence	Annealing Temp °C	BP analyzed	First Cited
COI	LCO 1490	5’-GGTCAACAAATCATAAAGATATTGG-3’	50	615	Hebert et al. 2003
	HCO 2198	5’-TAAACTTCAGGGTGACCAAAAAATCA-3’	50	615	Hebert et al. 2003
	1495b	5’-AACAAATCATAAAGATATTGGRAC-3’	50	615	This study
	rev 750	5’-GAAATTATNCCAATTCCTGG-3’	50	615	This study
	ScoCOI F215	5’-CCCCCGACATAGCTTTCCC-3’	50	271	This study
	ScoCOI Rev453	5’-TATTTGATCGAACTTTATTCC-3’	50	271	This study
28S	3665	5’-AGACAGAGTTCAAGAGTACGTG-3’	55	402–496	Belshaw and Quicke 1997
	4068	5’-TTGGTCCGTGTTTCAAGACGGG-3’	55	402–496	Belshaw and Quicke 1997
CAD	apCADforB2	5’-TGGAARGARGTBGARTACGARGTGGYCG-3’	56 or 58	690	Dole et al. 2010
	apCADfor4	5’-TGGAARGARGTBGARTACGARGTGGTYCG-3’	56 or 58	472	Danforth et al. 2006
	apCADrev1mod	5’-GCCATYRCTCBCCTACRCTYTTCAT-3’	56 or 58	472 or 690	Danforth et al. 2006
ArgK	ArgKforB2	5’-GAYTCCGGWATYGGWATCTAYGCTCC-3’	56 or 58	692	Dole et al. 2010
	ArgKrevB2	5’-GTATGYTCMCCRCGRGTACCACG-3’	56 or 58	692	Dole et al. 2010

Sequences were received as chromatograms and the sense and antisense strands were compiled using Sequencher (Ann Arbor, MI) to trim sequences, examine for ambiguities and create consensus sequences. Sequences were blasted in GenBank to examine for paralogous copies and other potential problems including contamination and pseudogenes. Sequences for COI, CAD and ArgK were aligned using SE-AL v.2.0a11 Carbon (http://tree.bio.ed.ac.uk/software/seal/). Sequence length variation was only observed in 28S. Sequences of 28S were manually aligned in SE-AL using a scolytine-specific secondary structure model (Jordal et al. 2008). Nexus files are available at http://www.scolytid.msu.edu.

## Methods

### Species concept

Species are hypotheses of unique evolutionary entities, tested by monophyly (*sensu* Wheeler and Platnick 2000). These monophyletic groups of individuals were named if they were diagnosable by synapomorphies or a unique combination of homoplastic characters. If diagnostic characters were not found, the clade retained the original species name or was synonymized with the sister clade. Therefore, given our species level morphological and molecular phylogenies, species revisions were based on monophyly of multiple individuals from disjunct populations.

### Morphology

*Parsimony phylogenetic analysis.* A phylogeny was reconstructed using the criterion of parsimony implemented in PAUP*4.0 b10 PPC (Swofford 2002). A heuristic search with 1,000 stepwise random additions with tree bisection-reconnection (TBR) for 37 taxa (25 ingroup, 12 outgroup) was performed. Characters were unordered and equally weighted. Bootstrap values were calculated by performing 1,000 pseudoreplicates with simple additions in PAUP*. Bremer support values were calculated by creating a constraint tree in TreeRot v.2 (Sorenson 1999) and analyzed in PAUP* using a heuristic search with 100 addition-sequence replicates.

### Molecular

*Likelihood phylogenetic analyses.* We analyzed the molecular and morphological datasets using Bayesian estimation of phylogeny with MrBayes 3.2.2 (Ronquist et al. 2012) on the CIPRES Science Gateway (Schwartz 2010). The Bayesian analysis consisted of a combined molecular and morphological dataset (82 taxa). The dataset was divided into 11 partitions by gene and codon position for COI, CAD and ArgK, and one partition each for 28s and morphology. We selected the best model for each data partition using MrModeltest (Nylander 2004). The GTR+I+Γ (general time reversible with a proportion of invariant sites and a gamma-shaped distribution of rate variation across sites) model selected by AIC was found to have the optimal fit for the gene partitions and Γ (gamma) was chosen for the morphology partition. Taxa that were unable to be sequenced were included in the morphology partition of the combined dataset.

Four Metropolis-Coupled Markov Chain Monte Carlo searches (3 heated, 1 cold) were performed for 10 million generations with sampling as described for the molecular dataset. *Scolytus dentatus*, *Scolytus hermosus*, *Scolytus mundus*, and *Scolytus silvaticus* were included in this analysis with only morphological characters. All parameters reached stability at 500,000 generations and the standard deviation of split frequencies between runs was 0.006983. Bayesian posterior probabilities of clades were calculated by a majority-rule consensus of those trees after the burn-in (75,000 trees).

*Parsimony phylogenetic analysis.* Intraspecific (Table 6) and interspecific (Table 7) differences for 83 taxa were generated by computing pairwise distances for each gene in PAUP*. A single gene phylogeny was reconstructed using COI using a heuristic search with 100 stepwise random additions with TBR for 89 taxa (79 ingroup, 10 outgroup). Bootstrap values were calculated by performing 1,000 pseudoreplicates with simple additions in PAUP*. Bremer support values were calculated by creating a constraint tree in TreeRot v.2 and analyzed in PAUP* using a heuristic search with 100 addition-sequence replicates.

**Table 6.** Intraspecific differences among genes expressed as the proportion of sites differing between sequences.

	**Gene**							
	**COI**		**28S**		**CAD**		**ArgK**	
**Species**	**Range**	**Average**	**Range**	**Average**	**Range**	**Average**	**Range**	**Average**
*Scolytus aztecus*	N/A	N/A	N/A	N/A	N/A	N/A	N/A	N/A
*Scolytus fagi*	0	0	0	0	N/A	N/A	N/A	N/A
*Scolytus fiskei*	0–0.0231	0.0183	0	0	0	0	0–0.0058	0.0034
*Scolytus intricatus*	N/A	N/A	N/A	N/A	N/A	N/A	N/A	N/A
*Scolytus laricis*	0–0.0043	0.0020	0	0	0	0	0.0043	0.0043
*Scolytus mali*	0.0433	0.0433	0	0	0	0	0–0.0030	0.0020
*Scolytus monticolae*	0–0.0017	0.0011	0	0	N/A	N/A	N/A	N/A
*Scolytus multistriatus*	0–0.0016	0.0033	0–0.0027	0.0009	0–0.0127	0.0078	N/A	N/A
*Scolytus muticus*	0.0034–0.0105	0.0079	0–0.0074	0.0049	0	0	0	0
*Scolytus obelus*	0.0081–0.0195	0.0135	0	0	0.0021	0.0021	0.0014	0.0014
*Scolytus oregoni*	N/A	N/A	0	0	0.0085	0.0085	0	0
*Scolytus piceae*	0–0.0163	0.0100	0–0.0025	0.0008	0–0.00850	0.0050	0	0
*Scolytus praeceps*	0–0.0148	0.0077	0	0	0	0	0–0.0044	0.0024
*Scolytus propinquus*	0	0	N/A	N/A	0	0	0.0063	0.0063
*Scolytus pygmaeus*	0	0	0	0.0000	0	0	0	0
*Scolytus quadrispinosus*	0.0016–0.0049	0.0032	0	0.0000	0	0	0.0014	0.0014
*Scolytus ratzeburgii*	N/A	N/A	N/A	N/A	N/A	N/A	N/A	N/A
*Scolytus reflexus*	0–0.0130	0.0070	0–0.0049	0.0012	0–0.0023	0.0003	0–0.0043	0.0029
*Scolytus robustus*	0.0033–0.0537	0.0358	0–0.0025	0.0017	0–0.0043	0.0029	0.0015–0.00612	0.0038
*Scolytus rugulosus*	N/A	N/A	0–0.0074	0.0050	0.0065–0.0192	0.0128	0–0.0274	0.0150
*Scolytus schevyrewi*	N/A	N/A	N/A	N/A	N/A	N/A	N/A	N/A
*Scolytus scolytus*	N/A	N/A	N/A	N/A	N/A	N/A	N/A	N/A
*Scolytus sinopiceus*	0.0157	0.0157	0.0123	0.0123	0	0	0	0
*Scolytus subscaber*	0.0158	0.0158	0.0049	0.0049	N/A	N/A	0.0101	0.0101
*Scolytus sulcifrons*	N/A	N/A	N/A	N/A	N/A	N/A	N/A	N/A
*Scolytus tsugae*	0.0016–0.0147	0.0103	0	0	0	0	N/A	N/A
*Scolytus unispinosus*	0.0016–0.0282	0.0121	0	0	0–0.0023	0.0015	0–0.0029	0.0007
*Scolytus ventralis*	0–0.0181	0.0089	0–0.0025	0.0010	0–0.0043	0.0022	0–0.0043	0.0022

**Table 7.** Interspecific differences among genes expressed as the proportion of sites differing among sister taxa.

	Gene							
	COI		28S		CAD		ArgK	
Species	Range	Average	Range	Average	Range	Average	Range	Average
*Scolytus aztecus* vs. *Scolytus ventralis*	N/A	N/A	0–0.0025	0.0020	0.0047–0.0088	0.0067	0.0119–0.0133	0.0128
*Scolytus fagi* vs. *Scolytus muticus*	0.1317–0.1382	0.1361	0.0176–0.0251	0.0201	0.0171–0.208	0.0190	0.0267	0.0267
*Scolytus fagi* vs. *Scolytus quadrispinosus*	0.1789–0.1821	0.1801	0.0528	0.0528	0.0531–0.0533	0.0532	0.0533–0.0549	0.0541
*Scolytus fiskei* vs. *Scolytus laricis*	0–0.0297	0.0143	0	0	0–0.0128	0.0051	0.0029–0.0072	0.0051
*Scolytus fiskei* vs. *Scolytus piceae*	0–0.0.028	0.0109	0.0025–0.0049	0.0031	0.0065–0.0234	0.0096	0.0130–0.0159	0.0145
*Scolytus fiskei* vs. *Scolytus unispinosus*	0.0331–0.0520	0.043	0	0	0–0.0149	0.0074	0–0.0072	0.0043
*Scolytus laricis* vs. *Scolytus piceae*	0–0.0179	0.0151	0.0025–0.0052	0.0032	0.0043–0.0149	0.0138	0.013–0.0145	0.01375
*Scolytus laricis* vs. *Scolytus unispinosus*	0.0397–0.0520	0.046	0	0	0.0043–0.0093	0.007	0–0.0043	0.0024
*Scolytus monticolae* vs. *Scolytus oregoni*	0.0390–0.0396	0.0393	0.0025	0.0025	N/A	N/A	0.0043–0.0046	0.0045
*Scolytus monticolae* vs. *Scolytus reflexus*	0.0765–0.0846	0.0798	0.0025–0.0049	0.0031	N/A	N/A	0.0072–0.0087	0.0077
*Scolytus monticolae* vs. *Scolytus tsugae*	0.0380–0.0413	0.0398	0.0025	0.0025	N/A	N/A	0.0185	0.0185
*Scolytus muticus* vs. *Scolytus quadrispinosus*	0.1626–0.1691	0.1659	0.0500–0.0575	0.0525	0.0488–0.0532	0.0510	0.0522–0.0552	0.0537
*Scolytus obelus* vs. *Scolytus praeceps*	0.0934–0.1089	0.1036	0.0025	0.0025	0.0021	0.0021	0.0052–0.0103	0.0089
*Scolytus oregoni* vs. *Scolytus tsugae*	0.0098–0.0179	0.0131	0.0000	0.0000	0.0064–0.0142	0.0103	0.0155–0.0169	0.0162
*Scolytus robustus* vs. *Scolytus subscaber*	0.0791–0.1041	0.0902	0.0025–0.0099	0.0066	0.0150–0.0194	0.0172	0.0089–0.01231	0.0107
*Scolytus rugulosus* vs. *Scolytus sinopiceus*	0.1260–0.1366	0.1320	0.0099–0.0149	0.0120	0.0336–0.0414	0.0370	0.0135–0.0245	0.0204

### Distribution mapping

Distribution maps were created in ArcMap (RockWare; Golden, Colorado, USA) by Thomas Atkinson (University of Texas) and are a combination of specimens examined as part of this study (black) and reported material examined by Atkinson and records gleaned from literature (white). A database of Atkinson’s localities is available at www.barkbeetles.info.

### Terminology

There are some traditionally used terms and characters in *Scolytus* literature that require clarification. The term ‘venter’ is regularly utilized. In general, the term refers to the entire ventral surface of an organism. However in *Scolytus* it only pertains to the abdominal ventral concavity, specifically ventrites 2–5. The length of ventrite 5 compared to ventrite 3 and 4 is another common misleading character in *Scolytus* literature. Rather than measuring the entire length of segment 5, only the distance from the basal margin to the subapical carinate ridge is measured and compared to the combined lengths of ventrites 3 and 4. The following *Glossary* (modified from Hopkins 1909; Torre-Bueno 1989; Edson 1967; Harris 1979) summarizes the meanings ascribed to some terms used in this monograph:

***General***

*Apical*: referring to a point at or close to the apex or tip.

*Basal*: referring to a point at or closest to the main body.

*Impressed*: a depression in a surface, typically referring the elytral striae or frons.

*Concave*: appearing hollowed out.

*Convex*: appearing rounded.

*Vestiture*: the surface covering composed of setae. In *Scolytus* the setae are either long or short.

***Head*** (Fig. 3)

**Figure 3.**
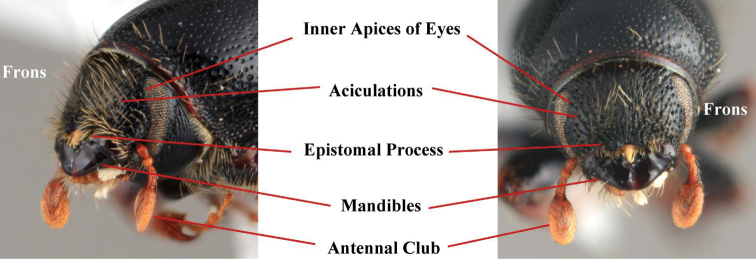
Terminology of the head (*Scolytus reflexus* male).

*Aciculate*: referring to longitudinal groves or scratches on the frons that can appear coarse as if made by a knife or fine as if lightly scratched with a needle.

*Epistomal process*: a raised, sinuate process composed of a median and two lateral sections apically fringed with thick, long bristles that cover the median epistomal area just above the mandibles.

*Frons*: region of the head from just above the epistoma to a point that is just dorsal to the inner apices of the eyes.

*Inner apices of eyes*: the innermost mesal margins of the eyes as viewed frontally.

*Strigate*: having narrow, transverse lines in the cuticle.

*Vertex*: the top of the head, above the eyes.

**Figure 4.**
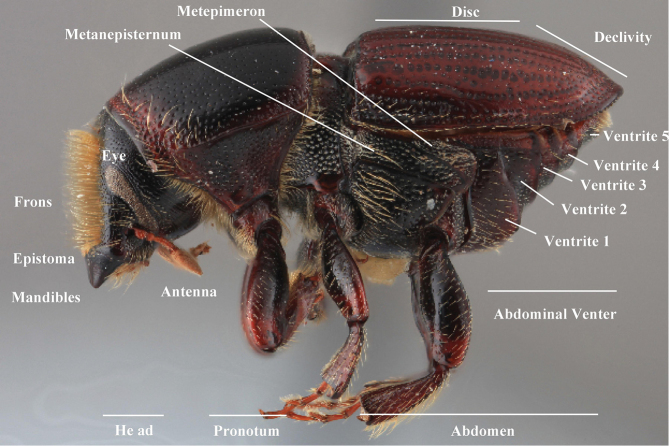
Habitus terminology (*Scolytus fagi* male), lateral.

**Figure 5.**
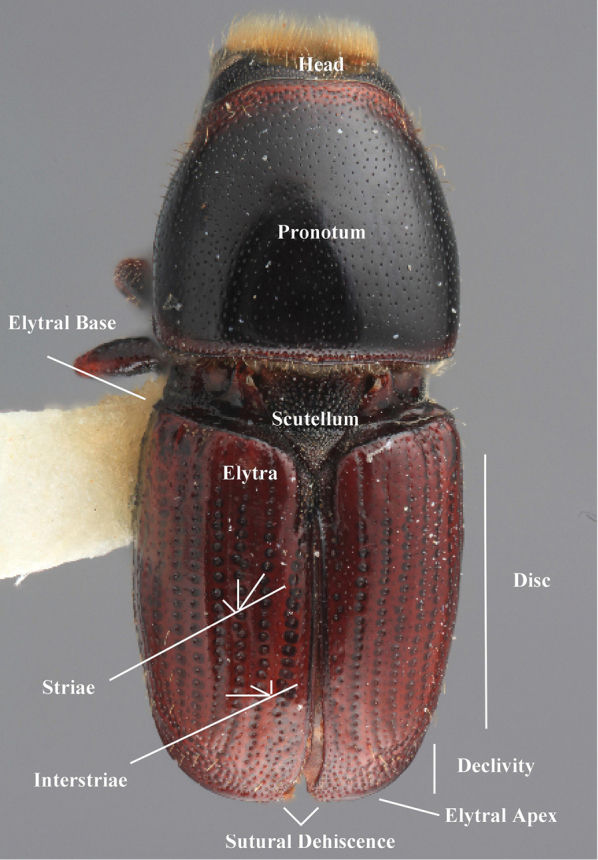
Habitus terminology (*Scolytus fagi* male), dorsal.

***Elytra*** (Figs 4–5)

*Apex*: end of a structure that is distal to the base.

*Disc*: the central upper surface of the elytra between the elytral bases and the sloped declivity.

*Striae*: punctures in rows, which may or may not be impressed to make grooves.

*Interstriae*: longitudinal spaces along the elytra between the striae, which is not as impressed and bear smaller punctures.

*Corrugated*: with alternate ridges and channels, referring to the appearance of the elytral striae and interstriae.

*Sutural dehiscence*: the central notch at the apical margin of the elytra.

**Figure 6.**
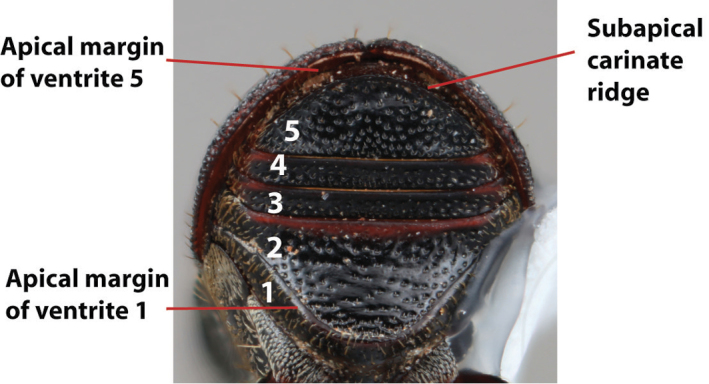
Abdominal venter terminology (*Scolytus robustus* female).

***Abdomen*** (Fig. 6)

*Carina*: a ridge-like or keel-shaped projection of the exoskeleton.

*Cusp*: a slight projection or elevation along a margin; refers to the apical margin of ventrite 1 in a few species.

*Denticle*: a small tooth.

*Opaque*: appearing dull in luster; referring to a surface that reflects little light.

*Produced*: refers to a part of the exoskeleton that is extended, lengthened, or elevated.

*Punctate*: set with fine impressed points appearing as pinpricks.

*Punctulate*: minute punctures.

*Rugose*: appearing wrinkled.

*Shagreened*: covered with a closely set roughness and appearing similar to sharkskin.

*Shiny*: appearing glossy or bright in luster; refers to a surface that appears polished and reflects light well.

*Spine*: an elongate projection of the exoskeleton that is longer than its basal width.

*Ventrite*: In *Scolytus* there are five visible ventrites. They are numbered from anterior to posterior with ventrite 1 closest to the head and ventrite 5 closest to the elytral apex.

*Subapical carinate ridge*: a carinate ridge on ventrite 5 occurring just before the apical margin.

*Tubercle*: a small knob-like or rounded protuberance of the exoskeleton.

*Tumescence*: a swelling of the exoskeleton.

*Venter*: the undersurface of the abdomen, in *Scolytus* this pertains to the five visible ventrites.

**Figure 7.**
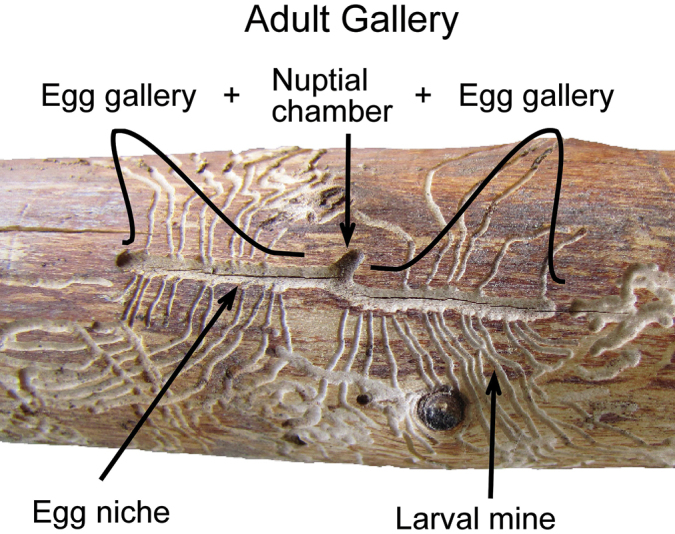
Gallery pattern terminology (*Scolytus monticolae*).

***Gallery pattern*** (Fig. 7)

*Adult gallery*: the composite tunnel produced by the adult female includes 1–2 egg galleries, the egg niches and the nuptial chamber (if present).

*Egg gallery*: a single extension of the adult gallery from the nuptial chamber (if present) along which eggs are deposited in niches.

*Egg niche*: notches along the sides of the egg gallery excavated by females in which a single egg is deposited.

*Larval mine*: the excavation tunnel produced by a larva as it feeds.

*Nuptial chamber*: an enlarged area or short extension of the adult gallery at the base of the entrance tunnel that may be used for mating or as a turning niche.

*Pupation chamber*: an ovoid or circular excavation at the end of a larval mine in which pupation occurs.

## Phylogenetic results

### Morphology

The morphological phylogeny was poorly resolved and few synapomorphic characters were found (Fig. 2). This reflects the morphologically similarity of many taxa. The morphological phylogeny recovered two clades: native hardwood species (*Scolytus fagi*, *Scolytus muticus* and *Scolytus quadrispinosus*) and conifer species (*Scolytus aztecus*, *Scolytus dentatus*, *Scolytus fiskei*, *Scolytus hermosus*, *Scolytus laricis*, *Scolytus monticolae*, *Scolytus mundus*, *Scolytus obelus*, *Scolytus oregoni*, *Scolytus piceae*, *Scolytus praeceps*, *Scolytus reflexus*, *Scolytus robustus*, *Scolytus silvaticus*, *Scolytus subscaber*, *Scolytus tsugae*, *Scolytus unispinosus*, and *Scolytus ventralis*). The outgroup taxa were poorly resolved except for *Scolytus rugulosus* and *Scolytus sinopiceus*, which were recovered as sister taxa. *Scolytus fiskei* was recovered as a distinct lineage separate from *Scolytus unispinosus*, of which it was considered a synonym. The relationship among *Scolytus obelus*, *Scolytus praeceps*, *Scolytus abietis* and *Scolytus opacus* was unresolved and *Scolytus reflexus*, *Scolytus virgatus* and *Scolytus wickhami* were strongly supported as monophyletic.

### Mitochondrial COI

**Figure 8.**
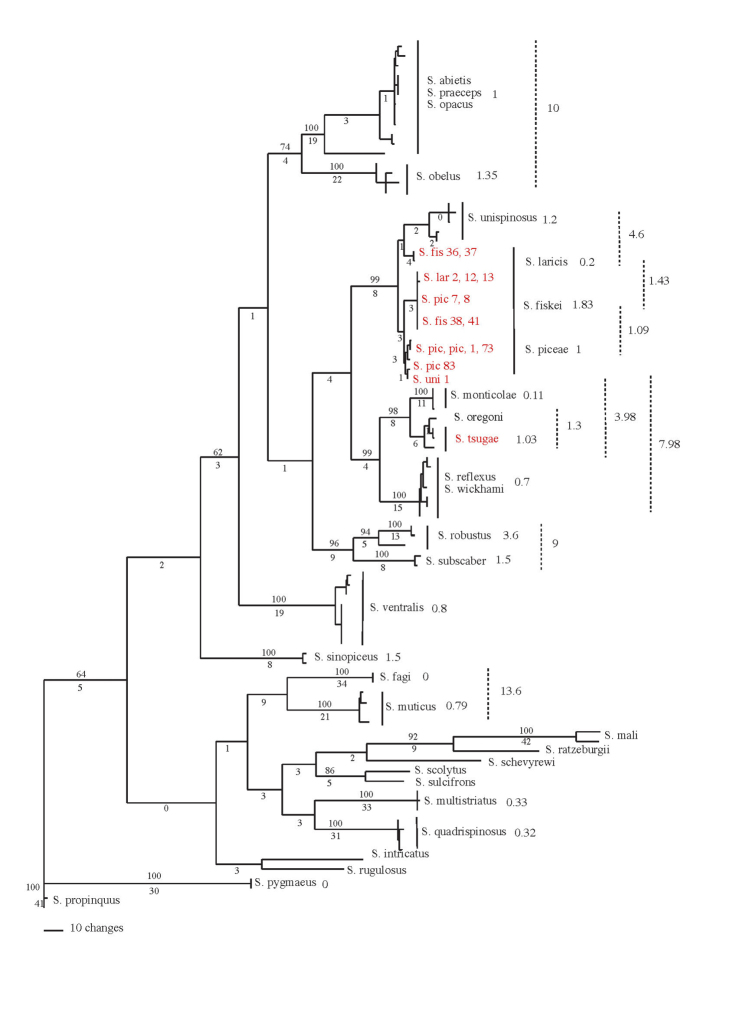
Mitochondrial COI phylogeny of Nearctic *Scolytus*. Phylogram of one of 2958 most parsimonious trees (L = 1431, CI = 0.356, RI = 0.809) generated from a heurtistic search of 1000 stepwise random additions with TBR in PAUP*. Numbers above the node indicate bootstrap values (>50). Bremer support values are listed below the node. Non-monophyletic species are indicated in red. Mean intraspecific nucleotide differences (listed next to each taxon) and mean interspecific nucleotide differences (right of solid line) are given. Taxa highlighted in red were not recovered as monophyletic.

The parsimony analysis of mitochondrial COI produced 2,958 most parsimonious trees with a length of 1,434 steps and 606 of 616 characters were parsimony informative (Fig. 8). There was a high degree of synapomorphy and low amount of homoplasy in the phylogeny (CI = 0.356, RI = 0.809).

### Combined analyses

**Figure 9.**
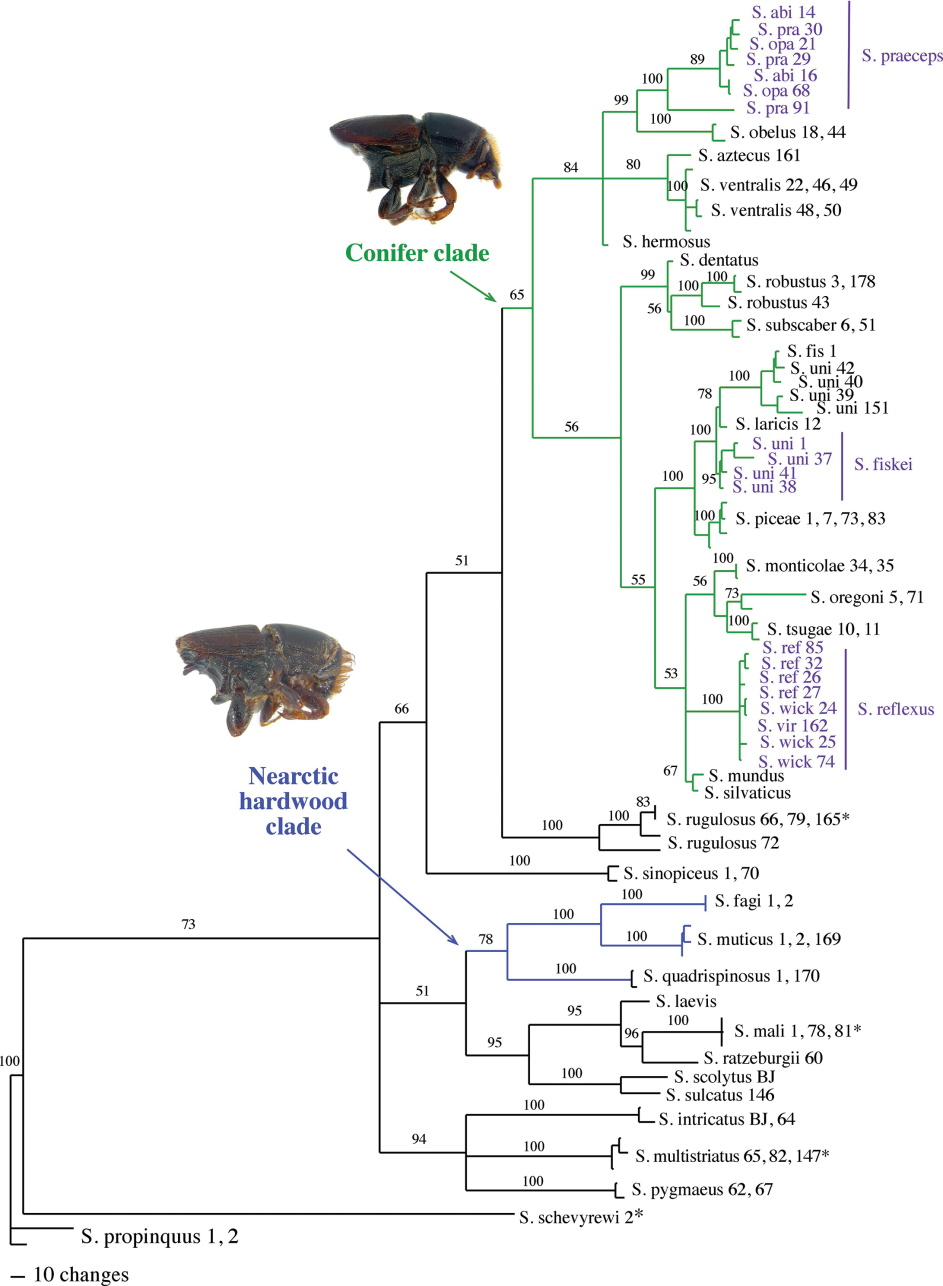
Bayesian tree found by analysis of data using combined molecular and morphological datasets. St. dev. = 0.006983. Numbers above the nodes are Bayesian posterior probabilities. Taxa in purple were not recovered as monophyletic. *Scolytus fiskei* was a synonym of *Scolytus unispinosus*. * denotes species exotic to the Nearctic.

Bayesian analysis of the combined dataset recovered the native Nearctic *Scolytus* as paraphyletic with native species found in two clades: native hardwood and conifer feeders (Fig. 9). Bayesian analysis of the combined dataset (Fig. 9) recovered the monophyletic groups observed in the morphological phylogeny and the native species were recovered in two clades: native hardwood-feeders (*Scolytus fagi*, *Scolytus muticus* and *Scolytus quadrispinosus*) and a second clade of conifer-feeders (*Scolytus aztecus*, *Scolytus dentatus*, *Scolytus fiskei*, *Scolytus hermosus*, *Scolytus laricis*, *Scolytus monticolae*, *Scolytus mundus*, *Scolytus obelus*, *Scolytus oregoni*, *Scolytus piceae*, *Scolytus praeceps*, *Scolytus reflexus*, *Scolytus robustus*, *Scolytus silvaticus*, *Scolytus subscaber*, *Scolytus tsugae*, *Scolytus unispinosus*, and *Scolytus ventralis*) sister to *Scolytus rugulosus*.

## Taxonomic treatment

Based on the results of our phylogenetic analyses, 25 species occur in the Nearctic. Several species were not recovered as monophyletic and had less than 1% difference in all genes sequences (Table 6). These species are thus placed in synonymy with the oldest described species. The following synonyms were discovered: *Scolytus praeceps* (= *Scolytus abietis*; = *Scolytus opacus*), *Scolytus reflexus* (= *Scolytus virgatus*; = *Scolytus wickhami*). *Scolytus fiskei* was recovered as monophyletic and is thus removed from synonymy with *Scolytus unispinosus*. Further detail regarding taxonomic changes is found in the remarks section for each species.

### A checklist of the Nearctic *Scolytus* species

*Scolytus aztecus* Wood, 1967

*Scolytus dentatus* Bright, 1964

*Scolytus fagi* Walsh, 1867

*Scolytus fiskei* Blackman, 1934, valid species

*Scolytus hermosus* Wood, 1968

*Scolytus laricis* Blackman, 1934

*Scolytus mali* (Bechstein, 1805) – Introduced

= *Scolytus sulcatus* LeConte, 1868

*Scolytus monticolae* (Swaine, 1917)

*Scolytus multistriatus* (Marsham, 1802) – Introduced

*Scolytus mundus* Wood, 1968

*Scolytus muticus* Say, 1824

*Scolytus obelus* Wood, 1962

*Scolytus oregoni* Blackman, 1934

*Scolytus piceae* (Swaine, 1910)

*Scolytus praeceps* LeConte, 1876

= *Scolytus abietis* Blackman, 1934, syn. n.

= *Scolytus opacus* Blackman, 1934, syn. n.

*Scolytus quadrispinosus* Say, 1824

= *Scolytus carya* Riley, 1867

= *Scolytus caryae* Walsh, 1867

*Scolytus reflexus* Blackman, 1934

= *Scolytus virgatus* Bright, 1972, syn. n.

= *Scolytus wickhami* Blackman, 1934, syn. n.

*Scolytus robustus* Blackman, 1934

*Scolytus rugulosus* (Müller, 1818) – Introduced

*Scolytus schevyrewi* Semenov, 1902 – Introduced

*Scolytus silvaticus* Bright, 1972, valid species

*Scolytus subscaber* LeConte, 1876

*Scolytus tsugae* (Swaine, 1917)

*Scolytus unispinosus* LeConte, 1876

= *Scolytus sobrinus* Blackman, 1934

*Scolytus ventralis* LeConte, 1868

### Scolytus

Taxon classificationAnimaliaColeopteraCurculionidae

Geoffroy, 1762

Scolytus Geoffroy, 1762: 309

#### Type species.

*Bostrichus scolytus* Fabricius, 1775.

See Alonso-Zarazaga and Lyal (2009) for complete taxonomic history.

#### Diagnostic characters.

*Scolytus* is easily distinguished from all other Nearctic scolytines by the unarmed protibia with a single curved uncus at the outer apical angle, by the flattened antennal club with 0–1 septate procurved sutures, by the seven-segmented funicle, by the slightly declivous elytra, the depressed scutellar notch and scutellum and by the abruptly ascending abdominal ventrites 2–5 (except *Scolytus rugulosus*).

#### Description

**(modified from Wood 1982).** Length 1.7–6.0 mm, 1.7–2.9 times as long as wide. Color variable, red-brown to black. Teneral adults are often light brown.

Head visible from above. Frons flattened to convex, sexually dimorphic (discussed below for each species group or clade). Eye elongate, sinuate to shallowly emarginated, finely faceted. Antennal scape club shaped, shorter than four funicle segments; funicle seven-segmented; club larger than funicle, flattened, oval to obovate, minutely pubescent and with 3 strongly procurved sutures, suture 1 partially to completely septate and with or without a surface groove. Pronotum large, wider than long; lateral and basal margins marked by a fine elevated line; disc finely punctate. Scutellum triangular, deeply depressed below elytral surface. Elytra depressed around scutellum and along basal one-fifth to half-length of elytral suture. Elytra wider than pronotum, flattened; striae punctate; interstriate punctate, with or without setae. Venter either gradually ascending from apical margin of ventrite 1 to elytral apex, or ventrite 2 abruptly ascending, sexually dimorphic, with or without tubercules and/or carinae; remaing ventrite usually unarmed, may have various armature or elevated margins, especially in males. Procoxae narrowly separated. Protibia rectangular; sides straight, nearly parallel, without denticles; outer distal angle produced into a curved uncus.

#### Species descriptions.

Species are redescribed and treated alphabetically by clade or group beginning with the introduced group, native hardwood clade and conifer clade. Common names listed are the official common names of the Entomological Society of America and Canada.

#### Introduced species group.

The introduced species group (1.9–4.2 mm long) (*Scolytus mali*, *Scolytus multistriatus*, *Scolytus rugulosus* and *Scolytus schevyrewi*) is not monophyletic and consists of a morphologically diverse group of Palearctic species that encompasses three formerly recognized subgenera (Butovitsch 1929; Balachowsky 1949). This diversity is reflected in the sexual dimorphism exhibited by the group. *Scolytus multistriatus* and *Scolytus schevyrewi* exhibit the typical frons dimorphism as discussed above. The venter lateral teeth are identical in both sexes of *Scolytus multistriatus* and the spine on ventrite 2 is smaller in the female compared to the male. Sexual dimorphism of *Scolytus mali* and *Scolytus rugulosus* is much more subtle and differentiating the sexes can be challenging. *Scolytus mali* males have the medial area of the frons and epistomal regions impressed and the lateral margins of the epistoma are lightly covered with more abundant erect setae than the female. *Scolytus rugulosus* males have the medial area of the frons and epistoma slightly more impressed and the frons bears longer and more abundant, erect setae.

### Scolytus mali

Taxon classificationAnimaliaColeopteraCurculionidae

(Bechstein, 1805)

Fig. 10

Bostrichus mali Bechstein, 1805: 882.
Scolytus mali (Bechstein, 1805): Eichhoff 1881: 41.
Scolytus sulcatus LeConte, 1868: 167. Brown 1950: 203.
Scolytus mali For complete taxonomic history see Wood and Bright (1992).

#### Diagnosis.

Both sexes of *Scolytus mali* are distinguished by having ventrite 2 at an oblique angle to ventrite 1, by the aciculate frons, with most setae found just above the epistoma and on the lateral epistomal margins, by the smooth, shining appearance of the pronotum and elytra, and by the weakly rounded and smooth elytral apex.

#### Description (male).

3.2–4.1 mm long (mean = 3.7 mm; n = 10); 2.1–2.5 times as long as wide. Head, pronotum and abdominal venter dark red-brown, legs and antennae light brown, elytra brown to red-brown. Pronotum typically darker than elytra.

*Head*. Epistoma weakly, broadly emarginate; epistomal process absent; median area above mandibles bearing dense patch of long, yellow, hair-like setae. Frons appearing convex when viewed laterally, moderately transversely impressed just above epistoma, longitudinally impressed near median line; densely, finely longitudinally aciculate-punctate; aciculations converging at epistoma; punctures small, coarse; sparsely covered by long, fine, erect, yellow hair-like setae, these as long as width of midpoint of eye, more abundant along epistoma. Antennal scape short, elongate; club flattened, irregularly ovoid, setose with partial septum, two arcuate sutures visible.

*Pronotum* wider than long; apical margin broadly rounded, median area between eyes lined with scales; sides distinctly arcuate, strongly constricted near apex, forming a weak transverse impression near apical margin; surface smooth, shining, punctures on disc fine, shallow, moderately abundant, larger and more abundant laterally and on apical constriction; apical and anterolateral margins bearing sparse, erect, yellow hair-like setae; base weakly bisinuate.

*Elytra* with sides sub-parallel on apical half, narrowing to weakly rounded, smooth apex; apex weakly emarginate at suture. Margin of apical edge bearing small, fine punctures. Disc smooth, shining; interstriae weakly impressed, more than twice width of striae, interstrial punctures uniseriate, smaller than those of striae, bearing sparse, long, semi-erect yellow hair-like setae (may be abraded); striae weakly impressed. Declivity bearing sparse, short, erect yellow setae. Metepimeron greater than half-length of metanepisternum.

*Venter.* Apical margin of ventrite 1 weakly elevated above base of ventrite 2. Ventrite 2 nearly at an oblique angle to ventrite 1; surface smooth, shining, finely punctate; punctures small, fine, shallow; surface convex, unarmed; setae erect, short, about half of ventrite 3 length; lateral margins of ventrites 2–3 and ventrite 4 unarmed. Ventrite 5 unarmed; length of ventrite 5 equal to combined lengths of ventrites 3 and 4; setal patch absent; median depression present.

**Figure 10.**
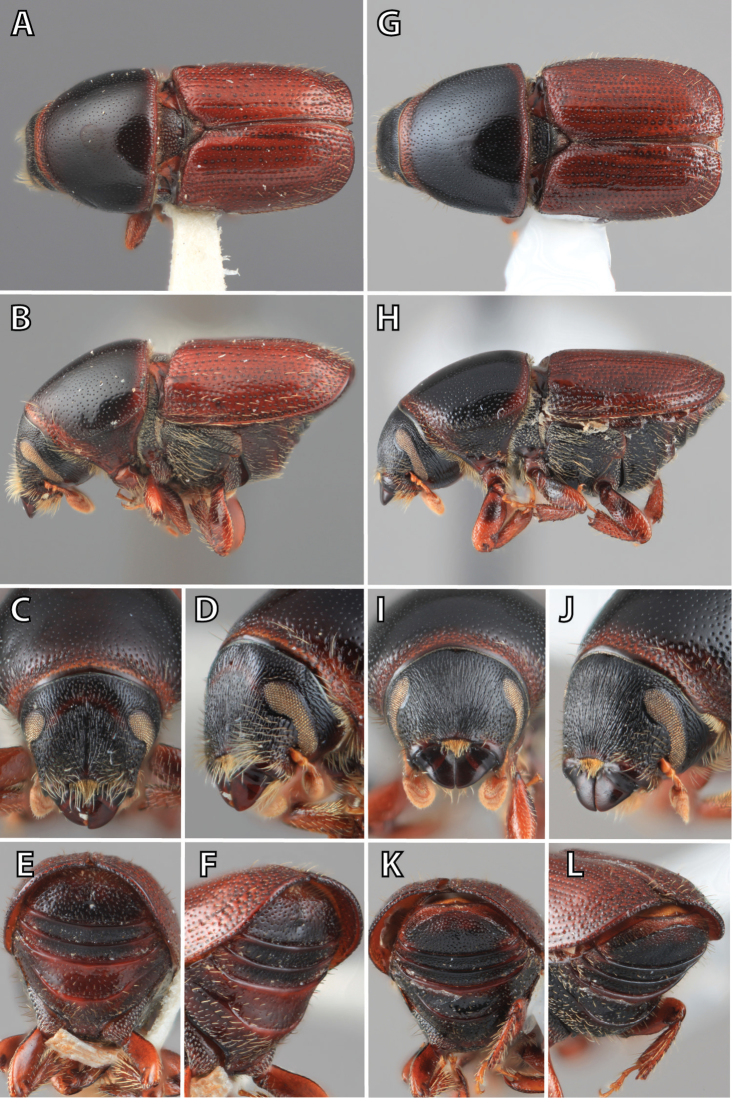
*Scolytus mali* **A** dorsal male habitus **B** lateral male habitus **C** male frons **D** male frons oblique **E** male venter **F** male venter oblique **G** dorsal female habitus **H** lateral female habitus **I** female frons **J** female frons oblique **K** female venter **L** female venter oblique.

#### Female.

3.0–4.2 mm long (mean = 3.7 mm; n = 10); 2.0–2.5 times as long as wide. Similar to male except epistoma feebly emarginate, frons more strongly convex when viewed laterally, weakly aciculate, setae sparser, shorter, less than width of eye.

#### Specimens examined.

111.

*Type material.* Syntypes *Bostrichus mali* Bechstein (location unknown). Holotype *Scolytus sulcatus* LeConte: male, labeled “[pink disc = Middle States (Md., Del., N.Y., N.J., Pa., Conn.?, R.I.?)]; type 969" (MCZC).

*Non-type material.* **CANADA:** ***ONTARIO*:** Hamilton, 14–21.VI.1981, M. Sanborne (CNCI-2), 23.VII.1980, ex. malaise trap (CNCI-1). Owen Sound, 27.V.[19]65, K.E. Stewart, ex. elm [= *Ulmus* sp.] (CNCI-1). Vineland Station, 8.VI.1949, W.L. Putnam, ex. apple [= *Malus* sp.] (CNCI-2). ***QUEBEC*:** Cantic, 11.VIII.1945, W.J. Brown, ex. apple [= *Malus* sp.] (CNCI-2). Cap-Saint-Ignace, VII.1970 (CNCI-1). Dunham, 2.IX.1998, Vignoble, L’Orpailleur, ECORC/CRDHAg-Cord (DEBC-1). Napierville, 2 mi N.E., 13.VIII.1945, W.J. Brown (CNCI-1). Noyan, 0.75 mi W., 15.VIII.1945, W.J. Brown (CNCI-1). **UNITED STATES:** ***CONNECTICUT*:** [*Fairfield Co.*]: Greenwich, 22.VII.1933, F.J. Dillaway, ex. in plum [= *Prunus* sp.] (USNM-2). *New Haven Co.*: New Haven, 25.VI.1956, C.W. O’Brien (EMEC-1). ***MAINE*:** [*Androscoggin Co.*]: Livermore Falls, 5.VIII.1975, ex. plum [= *Prunus* sp.] (USNM-1). ***MARYLAND*:** *Montgomery Co.*: Ashton, 4 mi S.W., 31.V.1986, G.F. & J.F. Hevel, ex. malaise trap (USNM-1). ***MASSACHUSETTS*:** *Worchester Co.*: 16.II.[19]53, ex. indoors (USNM-2). ***MICHIGAN*:** *Allegan Co.*: Fennville area, 30.VI.2003, P. McGhee, ex. apple trees [= *Malus* sp.] (MSUC-6). *Genesee Co.*: Richfield County Park, N43°100610, W-83°55810, 16.VI.2008, R. Mech, PI Anthony Cognato (MSUC-1). *Ingham Co.*: Lansing, 2 mi N., E. State Rd, N42.7842°, W84.5362°, 261 m, 2-18.VI.2007, PI Anthony Cognato, ex. Lindgren trap with ipslure (MSUC-1). *Kalamazoo Co.*: Gourdneck Lake State Game Area, 16.VII.2011, S.M. Smith, A.I. Cognato, ex. *Prunus* sp. (MSUC-2). *Oakland Co.*: Farmington Hills, N42°27.668', W83°25.579', 2.VII.2004, B. Sullivan, ex. Lindgren funnel with multistriatus lure (MSUC-2). *Saginaw Co.*: St. Charles, 25.VI.1968, J.G. Truchan, ex. rotary trap (MSUC-1), 25.VI.1969 (MSUC-1). *Wayne Co.*: 20.VI.1960, G. Steyskai (USNM-2). ***NEW JERSEY*:** [*Essex Co.*]: Maplewood, 7.VI.[19]34, D. Fivaz, ex. on elm [= *Ulmus* sp.] (USNM-12). [*Morris Co.*]: Chatham, 25.III.[19]34, W.D. Buchanan (USNM-1). ***NEW YORK*:** *Albany Co.*: near Rensselaerville, Huyck Preserve, 3-10.VII.1967, R. & J. Matthews, ex. window pane trap (CNCI-1). [*Suffolk Co.*]: Cutchogue, [19]45, Tuthill, ex. in apple [= *Malus* sp.] (USNM-26). [*Tompkins Co.*]: Groton, 24.V.1942, N.M. Downie (FMNH-1), 23.VI.1946 (FMNH-2). [*Westchester Co.*]: Armonk, 5.VI.[19]35, H. Dietrich, ex. apple [= *Malus* sp.] (CNCI-2). ***OHIO*:** *Medina Co.*: 15.VI.[19]62, C.L. Griswold (DEBC-4). ***PENNSYLVANIA*:** *Luzerne Co.*: Nanticoke, 20.IX.[19]60, ex. *Malus* sp. (CNCI-1). Nuangola, 12.V-23.VIII.1983, S. & J. Peck, ex. forest intercept (CNCI-1). ***VERMONT*:** *Rutland Co.*: Castleon, 18-19.VI.1989, H.V. Weems (FSCA-1). ***WASHINGTON, D.C.*:** 5.IV.1983, E.R. Hodges, ex. on sweater of collector (USNM-1). **Additional specimens:** *Czech Republic* (MSUC-21) and *Italy* (MSUC-1).

#### Distribution.

**Figure 11.**
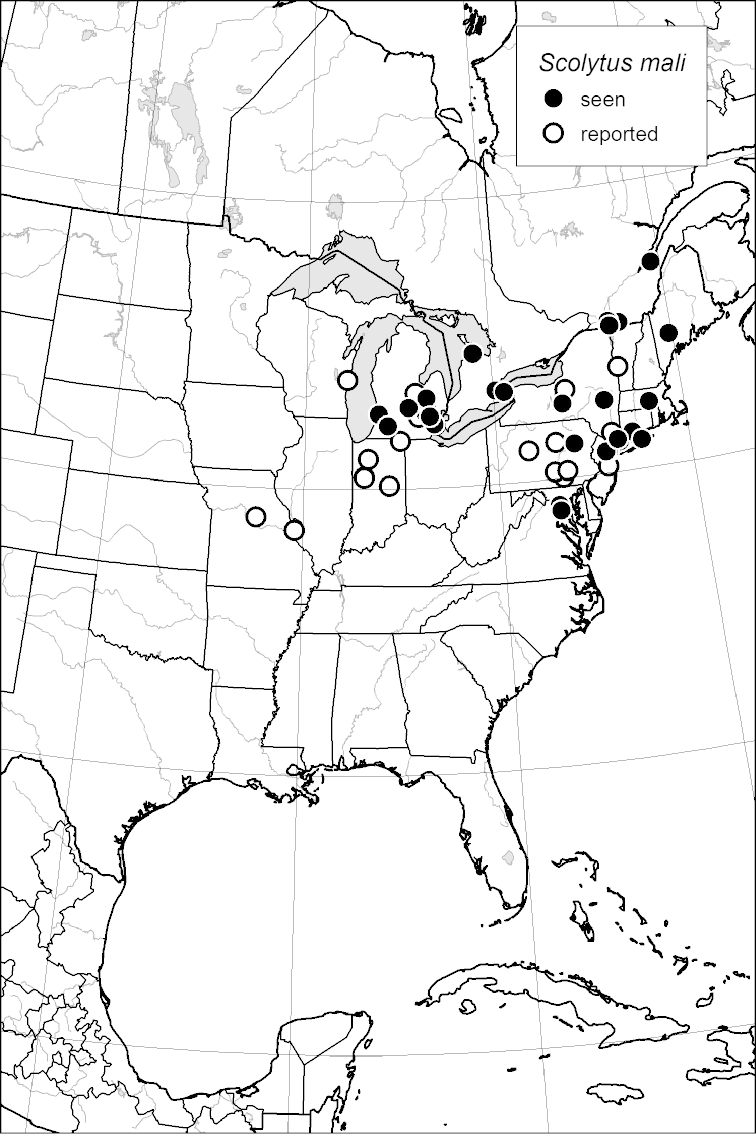
*Scolytus mali* distribution map.

CANADA: Ontario, Quebec. UNITED STATES: Connecticut, Maine, Maryland, Massachusetts, Michigan, New Jersey, New York, Ohio, Pennsylvania, Vermont, Washington, D.C., Wisconsin (Fig. 11).

#### Hosts.

*Malus* spp. (apple), *Prunus* spp. (cherry), *Ulmus* spp. (elm), *Pyrus* spp. (pear) and *Sorbus* spp. (mountain ash).

#### Common name.

Large shothole borer.

#### Biology.

This species colonizes dying and weakened limbs of its host as well as fresh slash. Typical host material is 15.0–38.0 cm in diameter but branches as small as 8.0 cm are colonized (Pechuman 1938).

Adult galleries are somewhat variable and are either parallel or at a slight oblique angle to the grain of the wood and consisting of a nuptial chamber and a single egg gallery. The adult gallery strongly scores the sapwood and ranges in size from 3.5–6.0 cm in length. Egg niches are constructed along the gallery and score the sapwood. Six to 100 eggs may be laid along the egg galleries with the typical gallery having 40 eggs. Larval mines lightly score the sapwood and radiate perpendicular to the egg gallery. Larval galleries later meander often at an oblique angle to the grain of wood, forming a fan shaped pattern. Pupation occurs within the sapwood and broods overwinter as larvae or adults. The following year, adults emerge and feed at twig crotches before selecting host material (Pechuman 1938; Balachowsky 1949; Baker 1972; Wood 1982). In New York, *Scolytus mali* has one generation per year although there are two generations per year in Europe (Pechuman 1938).

#### Collection notes.

The senior author collected this species from an 8.0 cm diameter *Prunus* sp. branch that had broken during a recent windstorm. Females constructed gallery entrances beneath large flakes of bark on the sides and bottom surfaces of the branch. Specimens were infesting the same limbs as *Phloeotribus liminaris* (Harris, 1852).

#### Remarks.

This species is native to the Palearctic region and was first detected in New York in 1868 when it was described as *Scolytus sulcatus* LeConte. LeConte (1868) noted in his description that the species most strongly resembled the Palearctic species *Scolytus rugulosus* and did not mention the collection date of his specimens. Interestingly, no other specimens were collected until 1933, 65 years after the initial discovery from southeastern New York, northern New Jersey and western Connecticut. It is likely that like LeConte’s *Scolytus californicus* (= *Scolytus scolytus*), *Scolytus mali* was collected in 1868 but populations never became established. The later collections may be the result of multiple introduction events (Pechuman 1938).

Brown (1950) recognized that LeConte’s species was morphologically and behaviorally identical to that of *Scolytus mali* and placed *Scolytus sulcatus* into synonymy. The native range of *Scolytus mali* is Europe, Central Asia, Siberia, the Russian Far East, North Africa (Michalski 1973; Knížek 2011).

### Scolytus multistriatus

Taxon classificationAnimaliaColeopteraCurculionidae

(Marsham, 1802)

Fig. 12

Ips multistriatus Marsham, 1802: 54.
Scolytus multistriatus (Marsham, 1802). Chapuis and Candeze 1853: 577.
Scolytus javanus Chapuis, 1869: 56. Schedl 1954: 137.
Scolytus multistriatus For complete taxonomic history see Wood and Bright (1992).

#### Diagnosis.

Both sexes of the species are distinguished by the presence of lateral teeth on the apical margins of ventrites 2–4 and by a median conical spine on the basal margin of ventrite 2.

#### Description (male).

2.2–3.9 mm long (mean = 2.81 mm; n = 20); 2.0–2.6 times as long as wide. Head, pronotum, and abdominal venter dark red-brown, legs light brown, antennae yellow-brown, elytra usually dark red-brown but may be brown. Color not uniform and pronotal and elytral surfaces frequently contain patches of red-brown mixed with dark red-brown. Pronotum typically darker than elytra.

*Head.* Epistoma weakly, broadly emarginate; epistomal process absent; median area above mandibles bearing dense patch of long, yellow, hair-like setae. Frons appearing flattened when viewed laterally; moderately, coarsely, longitudinally aciculate-punctate; aciculations converging at epistoma; punctures small, coarse; smoderately, uniformly covered by long, fine, yellow erect hair-like setae, these longer than width of midpoint of eye. Antennal scape short, elongate; club flattened, thinner on apical half, irregularly ovoid, setose with partial septum, two sharply arcuate sutures visible.

*Pronotum* wider than long; apical margin broadly rounded, median area between eyes lined with scales; sides distinctly arcuate, strongly constricted near apex, forming a weak transverse impression near apical margin; surface smooth, shining, punctures on disc fine, shallow, moderately abundant, larger and more abundant laterally and on apical constriction; apical and anterolateral margins bearing sparse, erect, yellow, hair-like setae; base weakly bisinuate.

*Elytra* with sides sub-parallel on apical half, narrowing to subquadrate, smooth apex; apex entire at suture. Margin of apical edge bearing small, fine punctures. Disc smooth, shining; interstriae weakly impressed, more than twice width of striae, interstrial punctures uniseriate, smaller than those of striae, bearing sparse, long, semi-erect yellow hair-like setae (may be abraded); striae weakly impressed. Declivity bearing sparse, short, erect yellow setae. Metepimeron greater than half-length of metanepisternum.

*Venter.* Apical margin of ventrite 1 weakly elevated above base of ventrite 2. Ventrite 2 nearly perpendicular to ventrite 1; surface smooth, shining, finely punctate, punctures small, coarse; surface flattened; basal margin armed with a long, smooth, conical spine with it’s base extending from basal margin to half length of segment; lateral margins of ventrites 2–4 armed with lateral tooth. Ventrite 5 carinate ridge closer to apical margin of segment; length of ventrite 5 greater than combined lengths of ventrites 3 and 4; setal patch and median depression absent.

**Figure 12.**
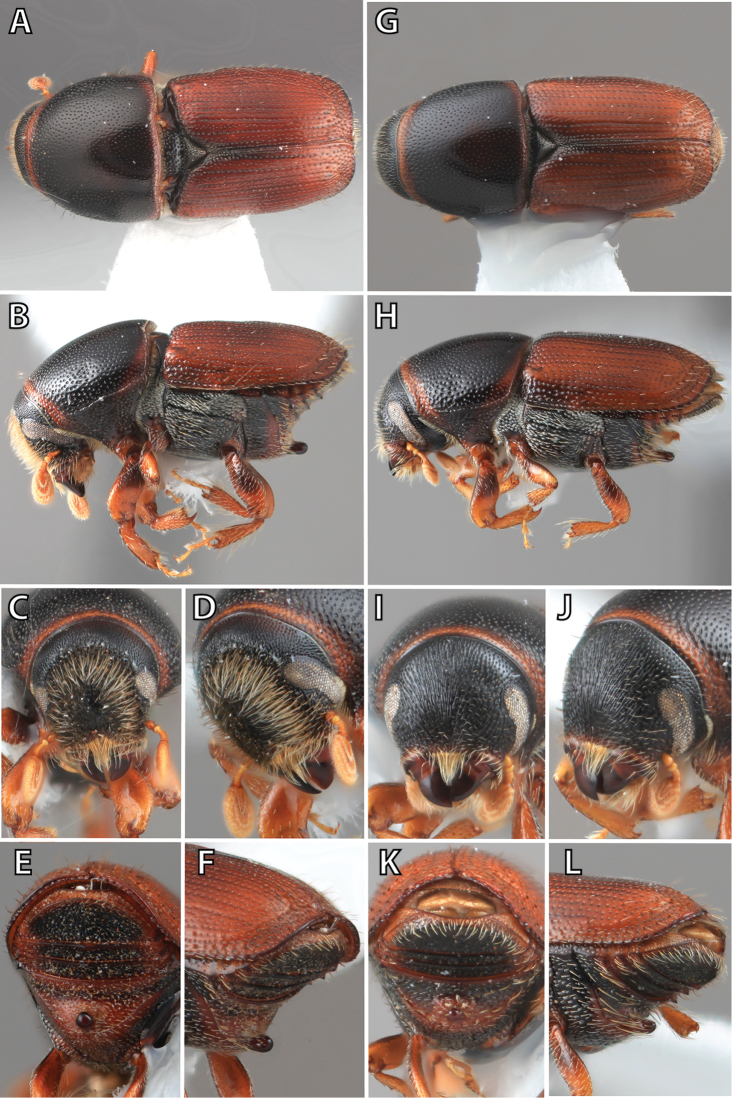
*Scolytus multistriatus* **A** dorsal male habitus **B** lateral male habitus **C** male frons **D** male frons oblique **E** male venter **F** male venter oblique **G** dorsal female habitus **H** lateral female habitus **I** female frons **J** female frons oblique **K** female venter **L** female venter oblique.

#### Female.

2.4–3.3 mm long (mean = 2.95 mm; n = 20); 2.2–2.7 times as long as wide. Similar to male except epistoma feebly entire, frons strongly convex when viewed laterally, weakly aciculate, setae sparser, shorter, less than the width of eye.

#### Specimens examined.

165.

#### Type material.

Holotype *Scolytus javanus* Chapuis: male, labeled “Java, Solier, Dejean” (ISNB).

#### Non-type material.

**CANADA:** ***ALBERTA*:** Calgary, 19.VII.[19]94, T. Reichardt, ex. pheromone trap no. EBB XI (CNCI-2). ***ONTARIO*:** St. Catherines, 22.VI.1961, Kelton, Brumpton (CNCI-1). Toronto, 1970, ex. reared on elm [= *Ulmus* sp.] (CNCI-1). Queenston, 26.VI.[19]50, ex. elm [= *Ulmus* sp.] (CNCI-1). ***QUEBEC*:** Montreal, 14.VII.1977, E.J. Kiteley (CNCI-1); Île de Montreal, Île de Sainte Hélène, 12.VIII.1985, L. Lariviere (CNCI-1). **MEXICO:** ***Chihuahua*:** Cuidad Juarez, II.1987, I.C. Rodriguez (CNCI-2). **UNITED STATES:** ***CALIFORNIA*:** [*Alameda Co.*]: Berkeley, 23.V.1962, ex. elm [= *Ulmus* sp.] (EMEC-2). *Contra Costa Co.*: Antioch [Dunes] National Wildlife Refuge, 10.X.[19]91, J.A. Powell (EMEC-1). *Fresno Co.*: 11.I.1950, N.J. Smith (SBMN-2). *Inyo Co.*: Big Pine, 4000 ft, V.1971, D. Guiliani, ex. black light (CASC-1), VII.1971 (CASC-1). ***COLORADO*:** *Mesa Co.*: Grand Junction, X.1979, D. Leatherman, ex. European ash [= *Fraxinus* sp.] (CSUC-1). *Weed Co.*: Briggsdale, 1.IX.198[sic!], D. Leatherman (CSUC-1). ***GEORGIA*:** *Clarke Co.*: Whitehall forest, 17-24.IX.1976, R. Turnbow, ex. window trap F-8 (CNCI-1). ***ILLINOIS*:** *Alexander Co.*: Horseshoe Lake, 28.IX.1968, T.E. Brooks (CNCI-4). ***KENTUCKY*:** *Green Co.*: 9.VII.1941, C. Cook (MSUC-1). ***MASSACHUSETTS*:** [*Hampshire Co.*]: Northampton, 10.VIII.1974, E.J. Kiteley (MSUC-1). [*Suffolk Co.*]: Forest Hills [= Boston], 3.VI.[?], W.M. Mann (USNM-1). ***MICHIGAN*:** *Antrim Co.*: Eastport, ca. 3 mi N., N45°08'30", W85°22'50", 3–5.VII.2003, F.W. Stehr, ex. UV light (MSUC-1). *Cass Co.*: Cassopolis, 2.VI.2007, A.D. Smith (MSUC-3). *Genesee Co.*: Richfield County Park, N43.100610°, W-83.55810°, ex. Lindgren with EtOH (MSUC-1). *Ingham Co.*: Lansing, 2 mi N., E. State Rd, N42.7842°, W84.5361°, 261 m, 16-30.VII.2007, PI A.I. Cognato (MSUC-1); G.L. Parsons, ex. UV light. & white lights (MSUC-1). East Lansing, 1.X.1957, R.C. Fox (MSUC-1), 2.X.1957 (MSUC-5), 3.X.1957 (MSUC-7), 4.X.1957 (MSUC-1), 5.X.1957 (MSUC-3), 6.X.1957 (MSUC-4), 10.X.1957 (MSUC-1), 15.X.1957 (MSUC-5); 25.VI.1981, R. Fischer (MSUC-1); N42°11.320', W84°27.867', 258 m, 16.IX.2011, S.M. Smith, A.I. Cognato, I.A. Cognato, ex. *Ulmus* sp. (MSUC-7); Michigan State University campus, 17.VI.2011, D.G. McCullough (MSUC-3). *Kalamazoo Co.*: Gourdneck Lake State Game Area, 19.VI.2011, A.I. Cognato, ex. *Ulmus* sp. (MSUC-21). *Kalkaska Co.*: T27N R7W S18, 28.XII.1986, P. Waclawski, ex. basement wood (MSUC-1), 20.XII.1987 (MSUC-1), 3.I.1987 (MSUC-5). *Lapeer Co.*: Potter Lake, 19.VI.1967, Brivio (MSUC-2). *Macomb Co.*: East of Memphis, 3.V.1964, C. Brivio (MSUC-1), 17.VI.1965 (MSUC-1), 21.VIII.[19]66 (MSUC-2), 23.VIII.1969 (MSUC-1), 30.VIII.1969 (MSUC-1). *Saginaw Co.*: St. Charles, 30.V.1969, J.H. Truchan, ex. rotary trap at 6 ft height level (MSUC-2), 11.VI.1969, ex. rotary trap at 12 ft height level (MSUC-1), 16.VI.1969 (MSUC-3). *Sanilac Co.*: Port Sanilac, 20.VI.1986, Brivio (MSUC-1). ***MONTANA*:** *Choteau Co.*: Great Falls, VII-VIII.1977, S. Kohler, ex. caught in flight, sticky trap, multilure bait (CNCI-1). *Gallatin Co.*: Bozeman, VII-VIII.1977, S. Kohler, ex. caught in flight, sticky trap, multilure bait (CNCI-1). ***NEW YORK*:** [*Westchester Co.*]: Yonkers, VIII.1935, H. Dietrich, ex. elm [= *Ulmus* sp.] (CNCI-3). ***OKLAHOMA*:** [*Oklahoma Co.*]: Jones, 13.VI.1957, D. Alexander (USNM-1). ***TEXAS*:** [*El Paso Co.*]: El Paso, 045483, 17.VII.[19]94, lot 94 07975, ex. Mexico–in log of *Prunus* sp. (USNM-10). ***WYOMING*:** *Carbon Co.*: Medicine Bow National Forest, VI-VIII.1999, ex. *Polyphorus volvatus* [= *Cryptoporus volvatus* (Peck) Shear] (CSUC-1). **Additional specimens:** *Czech Republic* (MSUC-2), *Italy* (MSUC-8) and *Russia* (MSUC-22).

#### Distribution.

**Figure 13.**
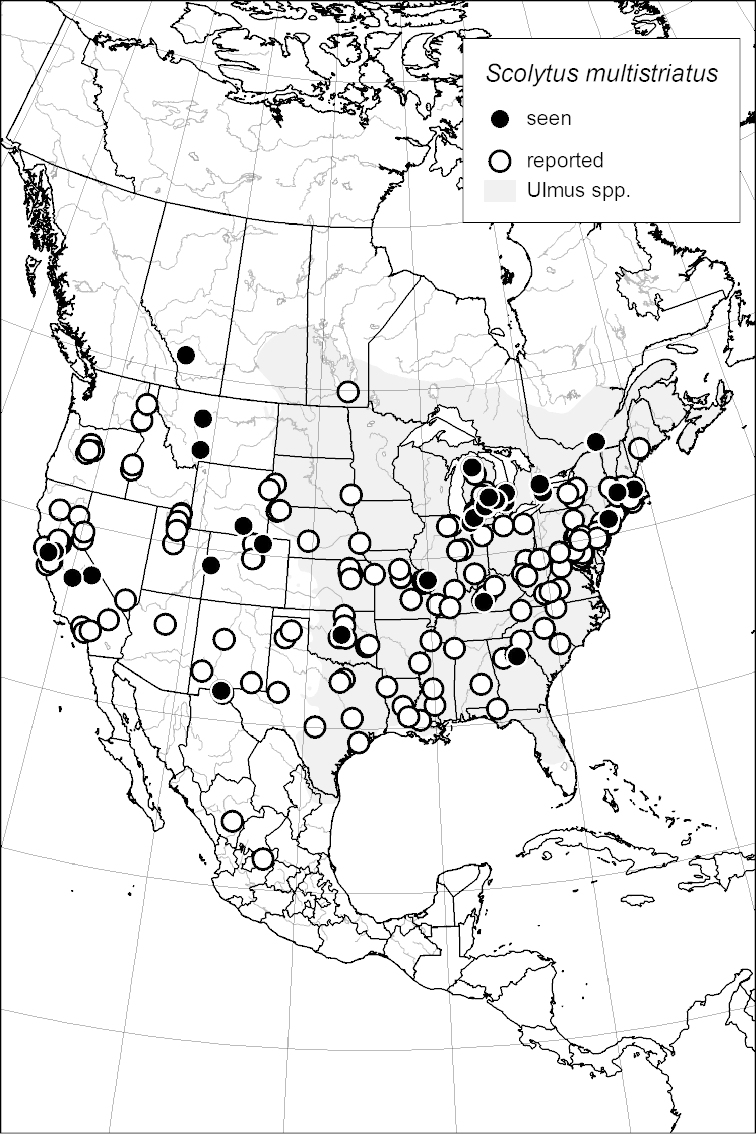
*Scolytus multistriatus* distribution map.

CANADA: Alberta, Manitoba, New Brunswick, Nova Scotia, Ontario, Quebec, Saskatchewan. MEXICO: Aguascalientes, Chihuahua. UNITED STATES: Alabama, Arizona, Arkansas, California, Colorado, Connecticut, Florida, Georgia, Idaho, Illinois, Indiana, Kansas, Kentucky, Louisiana, Maine, Maryland, Massachusetts, Michigan, Missouri, Montana, Nebraska, Nevada, New Hampshire, New Jersey, New Mexico, New York, North Carolina, North Dakota, Ohio, Oklahoma, Oregon, Pennsylvania, Rhode Island, South Carolina, South Dakota, Tennessee, Texas, Utah, Vermont, Virginia, Washington, D.C., West Virginia, Wisconsin, Wyoming (Fig. 13).

#### Hosts.

All native and introduced *Ulmus* spp. including *Ulmus americana* L. (American elm) and *Zelkova serrata* (Thunb.) Makino.

#### Common names.

Smaller European elm bark beetle and the European elm bark beetle.

#### Biology.

*Scolytus multistriatus* colonizes cut, stressed, weakened and diseased elm trees (*Ulmus* spp.) (Wood 1982). It seldom attacks healthy and vigorous trees (Bright 1976). *Scolytus multistriatus* females produce an aggregation pheromone to aggregate conspecifics to host trees. The pheromone bouquet is composed of three components: (-)-4-methyl-3-heptanol, (-)-2,4-dimethyl-5-ethyl-6,8-dioxabicylo[3.2.1]octane (α)-multistriatin and (-)-α-cubebene (Pearce et al. 1975).

Mating can occur either as the female is undergoing maturation feeding in twig crotches or within the gallery as described for the genus (Svihra and Clark 1980). The adult gallery is excavated parallel to the grain of the wood and consists of a single egg gallery; a nuptial chamber is not constructed. The adult gallery ranges in size from 2.5–5.0 cm in length. Egg niches are constructed along the gallery and score the sapwood. Twenty-four to 96 eggs may be laid singly along the egg gallery. Larval mines lightly score the sapwood and radiate perpendicular to the egg gallery. Larval galleries later meander often at an oblique angle to the grain of wood, forming a fan shaped pattern. Larvae construct pupal chambers in the bark (Bright 1976).

There are one to one-half generations per year in Canada and three in the southern US (Furniss and Johnson 2002). In Canada, adults emerge in June and July and feed at twig crotches of healthy trees for 7–10 days before selecting host material (Chamberlin 1958; Baker 1972; Svihra and Clark 1980). The brood from these adults either emerges in August or September or overwinters as larvae.

*Scolytus multistriatus* is the principal vector of the Dutch elm disease fungus *Ophiostoma ulmi* (Buisman) Melin & Nannf in North America. This beetle vectored disease killed 50–75% of the elms population in northeastern North America prior to the 1930s (Bloomfield 1979). Adults become covered in fungal spores upon emergence from brood material. Adults inoculate elms with the Dutch elm disease fungus as they feed in twig crotches (Svihra and Clark 1980). This feeding activity leaves wounds in the bark that allow spores to be transferred from the beetle’s cuticle to the tree tissues (Bright 1976).

#### Remarks.

This species is native to the Palearctic region and is primarily distributed throughout Europe but also occurs in Iran and Algeria (Knížek 2011). *Scolytus multistriatus* was first detected in North America in 1909 from elm trees on the Harvard University campus in Massachusetts (Chapman 1910).

### Scolytus rugulosus

Taxon classificationAnimaliaColeopteraCurculionidae

(Müller, 1818)

Fig. 14

Bostrichus rugulosus Müller, 1818: 247.
Scolytus rugulosus (Müller, 1818): Eichhoff 1881: 41.
Scolytus rugulosus For complete taxonomic history see Wood and Bright (1992).

#### Diagnosis.

Both sexes of *Scolytus rugulosus* are distinguished by the rounded elytral apices, by the serrate and deeply emarginated elytral apex, and by the oblique ventrite 2.

#### Description (male).

1.9–2.6 mm long (mean = 2.4 mm; n = 10); 2.1–2.7 times as long as wide. Color red-brown to dark red brown, antenna yellow-brown. Pronotum typically darker than elytra.

*Head.* Epistoma weakly, broadly emarginate; epistomal process absent; median area above mandibles bearing dense patch of long, yellow, hair-like setae. Frons appearing convex when viewed laterally, slightly transversely impressed just above epistoma; moderately, finely, longitudinally aciculate-punctate; aciculations converging at epistoma; punctures small, coarse; moderately, uniformly covered by long, fine, yellow erect hair-like setae, these longer than width of midpoint of eye. Antennal scape short, elongate; club flattened, irregularly ovoid, setose with partial septum, two arcuate sutures visible.

*Pronotum* wider than long; apical margin broadly rounded, median area between eyes lined with scales; sides distinctly arcuate, strongly constricted near apex, forming a weak transverse impression near apical margin; surface smooth, shining, punctures on disc fine, shallow, moderately abundant, larger and more abundant laterally and on apical constriction; apical and anterolateral margins bearing sparse, erect, yellow setae; base weakly bisinuate.

*Elytra* with sides sub-parallel on basal half, narrowing to strongly rounded, moderately serrate apex; apex strongly emarginated at suture. Margin of apical edge bearing large, coarse punctures. Disc glabrous, smooth, shining; interstriae not impressed and equal in width to striae, interstrial punctures uniseriate, equal in size to those of striae, bearing moderately abundant short, semi-erect yellow-brown hair-like setae; striae not impressed. Declivity bearing sparse, short, erect yellow setae. Metepimeron less than half-length of metanepisternum.

*Venter.* Apical margin of ventrite 1 rounded, flush with base of ventrite 2. Ventrite 2 nearly at an oblique angle to ventrite 1; surface shagreened, dull finely punctate; punctures small, coarse, shallow; surface flattened, unarmed; setae abundant, erect, long, greater than length of segment 3; lateral margins of ventrites 2–3 and ventrite 4 unarmed. Ventrite 5 carinate ridge closer to apical margin of segment; length of ventrite 5 greater than combined lengths of ventrites 3 and 4; setal patch absent, median depression present.

**Figure 14.**
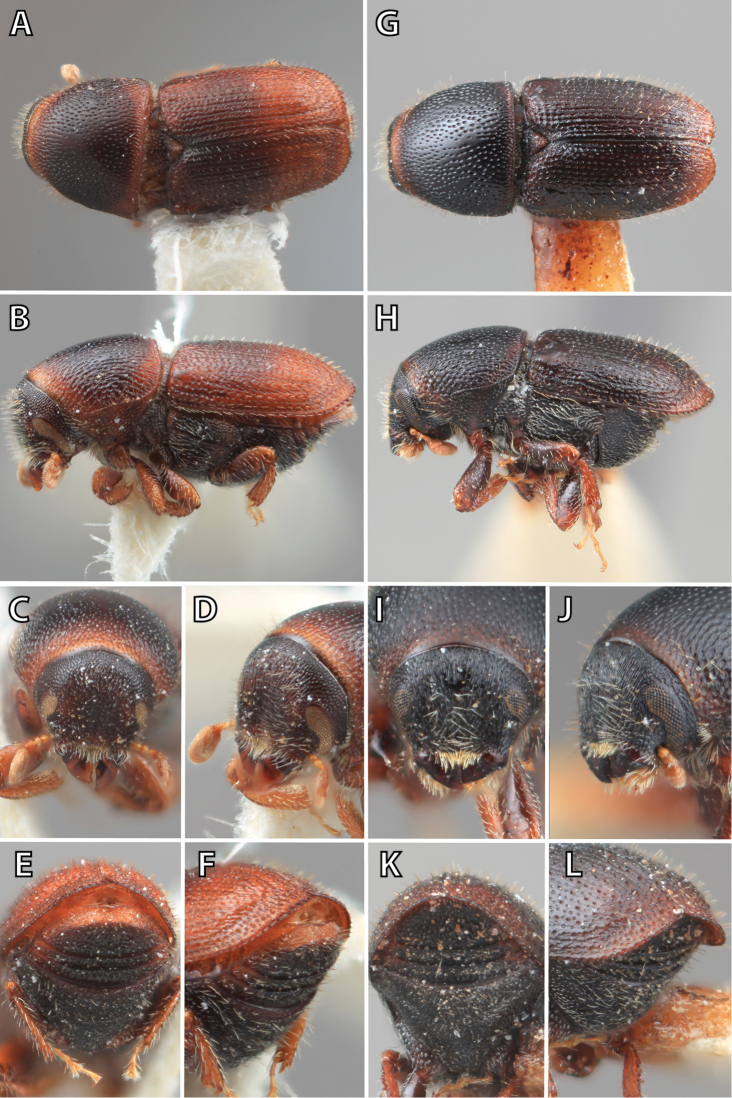
*Scolytus rugulosus* **A** dorsal male habitus **B** lateral male habitus **C** male frons **D** male frons oblique **E** male venter **F** male venter oblique **G** dorsal female habitus **H** lateral female habitus **I** female frons **J** female frons oblique **K** female venter **L** female venter oblique.

#### Female.

2.0–3.1 mm long (mean = 2.5 mm; n = 10); 2.1–2.8 times as long as wide. Similar to male except frontal setae sparser, shorter, less than width of eye.

#### Specimens examined.

102.

#### Type material.

Syntypes: (ISNB, ZIFH). None examined.

#### Non-type material.

**CANADA:** ***BRITISH COLUMBIA*:** Vancouver, 5.VII.1975, N.M. Downie (FMNH-2). ***NOVA SCOTIA*:** Middleton, 11.VI.1938 (CNCI-2). ***ONTARIO*:** Brimley, 17.VII.[19]21 (CNCI-1), 30.VII.[19]22 (CNCI-1). Ottawa, 21.VIII.1999, A.T. Howden, ex. wild grape [= *Vitis* sp.] (CNCI-1). Simcoe, 3.IX.1931, J.A. Hall (CNCI-2). Smith Falls, V.1940, H.S. Fleming (CNCI-1). **UNITED STATES:** ***ARIZONA*:** [*Cochise Co.*]: Green Canyon, 26.IX.[19]98, Sequeira and Jordal, ex. *Fraxinus* sp. (ZMBN-1). ***CALIFORNIA*:** [*Los Angeles Co.*]: San Marino, 25.VI.[19]42, G.P. Mackenzie (FMNH-1). [*Orange Co.*]: Santa Ana, 1.IV.1943, L.R. Gillogly, ex. bait traps (USNM-1). [*Placer Co.*]: Penryn, 1.X.1929, A.T. McClay (FMNH-2). ***DELAWARE*:** [*Kent Co.*]: Camdem, XII.1940, J.M. Amos (FMNH-1). ***INDIANA*:** *Madison Co.*: 10.VIII.1937, ex. apricot [= *Prunus armeniaca*] (FMNH-2). *Porter Co.*: Jackson Township, Maple Knoll Farm, 6.IV.[19]60, C.C. Gregg (FMNH-10). *Tippecanoe Co.*: 7.VI.1956, N.M. Downie (FMNH-2), 1.XI.1978 (FMNH-1). ***MICHIGAN*:** *Allegan Co.*: Fennville area, 30.VI.2003, P. McGhee, ex. on apple trees [= *Malus* sp.] (MSUC-1). [*Cass Co.*]: Edwardsburg, 30.VIII.1928 (MSUC-6). [*Ingham Co.*]: [East Lansing], Agriculture College [= Michigan State University campus], 1.I.1917 (MSUC-3). *Isabella Co.*: 16.IX.[19]55, R.R. Dreisbach (MSUC-1). [*Kent Co.*]: Grand Rapids, 17.X.[19]11 (MSUC-6). [*Lenawee Co.*]: Adrian, 20.VII.1900 (MSUC-7). *Livingston Co.*: Howell, 214 Inverness St, N42.61678°, W84.92810°, 23.V-7.VI.2007, R. Mech, ex. Lindgren trap with EtOH + alpha (MSUC-1). *Midland Co.*: 24.VII.[19]46, R.R. Dreisbach (MSUC-1), 14.IX.[19]56 (MSUC-1). *Oakland Co.*: A.W. Andrews (MSUC-1); 28.VI.[19]47, B. Summerville (MSUC-1). [*Washtenaw Co.*]: Manchester, 21.V.1913 (MSUC-4), 31.V.1913 (MSUC-7). ***NEW YORK*:** *Onondaga Co.*: Syracuse, X.1987, R.J. Rabaglia, ex. mountain ash [= *Sorbus* sp.] (RJRC-1). [*Tompkins Co.*]: Ithaca, ex. peach [= *Prunus* sp.] (CASC-1). [*Ulster Co.*]: Oliverea, 20.VI.[19]18 (USNM-1). *Wayne Co.*: 26.VI.1950, Shumaker (USNM-1), 8.VIII.[19]51 (USNM-1). [*Unspecified County*]: (CASC-2). ***PENNSYLVANIA*:** *Allegheny Co.*: Upper St. Clair Township, 28.VIII.[19]59 (EMEC-1). [*Philadelphia Co.*]: Philadelphia, 20.V.[18]98 (USNM-1). ***UTAH*:** [*Utah Co.*]: Vineyard, 6.IX.[19]23, T. Spalding (USNM-1). ***WASHINGTON*:** [*Whitman Co.*]: Steptoe Butte State Park, 9.VII.1971, N.M. Downie (FMNH-1). ***WASHINGTON, D.C.*:** 26.VII.1943, L.J. Bottimer, ex. on *Prunus* sp. (CNCI-2). **ADDITIONAL SPECIMENS:** *Brazil* (MSUC-21), *Czech Republic* (MSUC-1), *Hungary* (MSUC-1), *Morocco* (MSUC-1) and *Italy* (MSUC-2).

#### Distribution.

**Figure 15.**
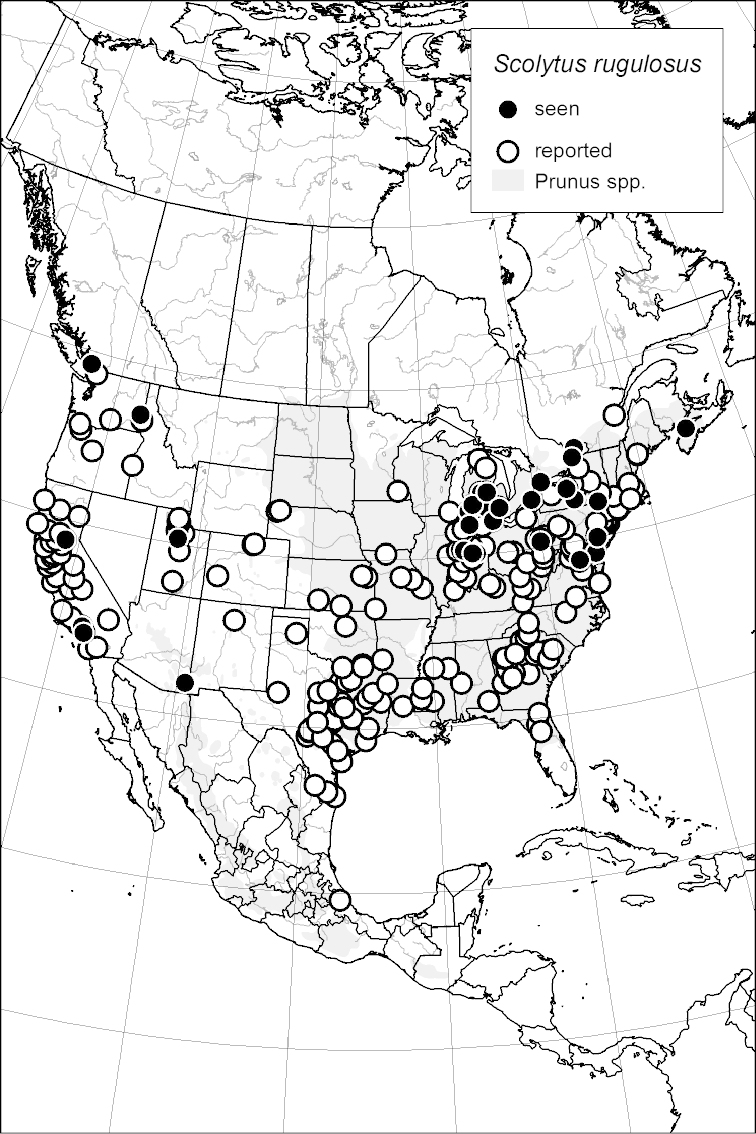
*Scolytus rugulosus* distribution map.

CANADA: British Columbia, New Brunswick, Nova Scotia, Ontario, Prince Edward Island. Greenland. MEXICO: Durango. UNITED STATES: Arizona, Arkansas, California, Colorado, Connecticut, Delaware, Florida, Georgia, Idaho, Illinois, Indiana, Iowa, Kansas, Kentucky, Louisiana, Maryland, Massachusetts, Michigan, Mississippi, Missouri, Montana, New Jersey, New York, North Carolina, Ohio, Oregon, Pennsylvania, South Carolina, Texas, Utah, Virginia, Washington, Washington D.C, West Virginia, Wisconsin (Fig. 15).

#### Hosts.

Stone and pome fruit trees including *Malus* spp. (apple), *Pyrus* spp. (pear), and *Prunus* spp. (cherry) and is less common in *Crataegus* (hawthorn), *Sorbus* (mountain ash) and *Ulmus* spp. (elm).

#### Common name.

Shothole borer.

#### Biology.

*Scolytus rugulosus* primarily attacks and kills small twigs and branches but may kill whole trees through the process of excavating adult galleries and larval feeding. Twig injury is the result of feeding activity at the base of the bud. Attacks can begin near an injury or on a healthy tree (Chamberlin 1939; Bright and Stark 1973).

The adult gallery is parallel to the grain of the wood and solely consists of an egg gallery; a nuptial chamber is not constructed. The adult gallery strongly scores the cambium, lightly scores the sapwood and ranges in size from 1.0–5.0 cm in length (Bright and Stark 1976; Furniss and Johnson 2002). Eggs are laid singly in larval niches on both sides of the egg gallery. Larval mines first radiate perpendicular to the egg gallery against the grain of the wood and later turn parallel to the grain (Bright and Stark 1973). The larval stage typically lasts one month. Pupation occurs in the bark for summer emergence. If the brood is unable to complete development during the warmer months, larvae will burrow 1.0–2.0 cm deep within the sapwood to overwinter. The following year, adults emerge and feed at twig crotches before selecting host material (Baker 1972). There are one to four generations per year depending on locality, with more generations occurring in warmer climates (Chittenden 1898; Baker 1972; Wood 1982).

#### Remarks.

This species is native to the Palearctic region and was first detected in North America in 1877 from New York (Chittenden 1898). The native range of *Scolytus rugulosus* encompasses Europe, North Africa, Asia Minor and middle Asia to Zabaikalye (Russia) (Michalski 1973; Knížek 2011).

### Scolytus schevyrewi

Taxon classificationAnimaliaColeopteraCurculionidae

Semenov, 1902

Fig. 16

Scolytus schevyrewi Semenov, 1902: 265.
Scolytus schevyrewi For complete taxonomic history see Wood and Bright (1992).

#### Diagnosis.

The *Scolytus schevyrewi* male most strongly resembles that of *Scolytus piceae* because in both species the spine on the second ventrite never attains the apical margin. Both sexes can be distinguished from *Scolytus piceae* by the subapical carina on ventrite 5 located just before end of segment. Males are further differentiated from those of *Scolytus piceae* by the laterally compressed spine with a bulbous apex, which frequently has a longitudinal groove and by the banded appearance of the elytra. The female is further differentiated from that of *Scolytus piceae* by the banded pattern of the elytra and by the low median carina (variable and may be absent) that does not touch either margin on the second ventrite.

#### Description (male).

2.8–3.5 mm long (mean = 3.2 mm; n = 10); 2.0–2.2 times as long as wide. Color red-brown to dark red brown. Elytra of most specimens appears banded with a characteristic dark brown band on reddish colored elytra, but may also be solely dark brown or red-brown without a band. Antennae yellow-brown. Pronotum typically darker than elytra.

*Head.* Epistoma weakly, broadly emarginated; epistomal process absent; median area above mandibles bearing dense patch of long, yellow, hair-like setae. Frons appearing impressed when viewed laterally; moderately, coarsely reticulate punctate to weakly longitudinally aciculate; punctures dense, small, coarse; moderately covered by long, fine, yellow, erect, hair-like setae, these longer than width midpoint of eye, setae on lateral and dorsal margins longer, thicker, incurved. Antennal scape short, elongate; club flattened, thinner on apical half, irregularly ovoid, setose with partial septum and two arcuate sutures visible.

*Pronotum* wider than long; apical margin broadly rounded, median area between eyes lined with scales; sides distinctly arcuate, strongly constricted near apex, forming a weak transverse impression near apical margin; surface smooth, shining, punctures on disc fine, shallow, moderately abundant, larger, coarser, deeper and more abundant laterally and on apical constriction; apical and anterolateral margins bearing sparse, erect, yellow, hair-like setae; base weakly bisinuate.

*Elytra* with sides sub-parallel on basal half, narrowing to moderately rounded, smooth apex; apex weakly emarginated at suture. Margin of apical edge bearing small, fine punctures. Scutellar notch densely covered in recumbent white setae (may be abraded). Disc glabrous, smooth, shining; interstriae not impressed, equal in width to striae; interstrial punctures uniseriate, equal in size to those of striae, bearing sparse short, semi-erect yellow hair-like setae; striae weakly impressed. Declivity bearing sparse, short, erect yellow setae. Metepimeron half-length of metanepisternum.

*Venter.* Apical margin of ventrite 1 flush with base of ventrite 2. Ventrite 2 nearly perpendicular to ventrite 1; surface shagreened, dull, finely punctate; punctures small, fine, shallow; surface convex; densely covered with semi-recumbent, long setae, as long as length of ventrite 3 or less; surface armed with median laterally compressed spine (rarely absent) that has its base close to basal margin but does not touch it; lateral margins of ventrites 2–3 and ventrite 4 unarmed. Ventrite 5 carinate ridge closer to apical margin of segment; length of ventrite 5 greater than combined lengths of ventrites 3 and 4; setal patch and median depression absent.

**Figure 16.**
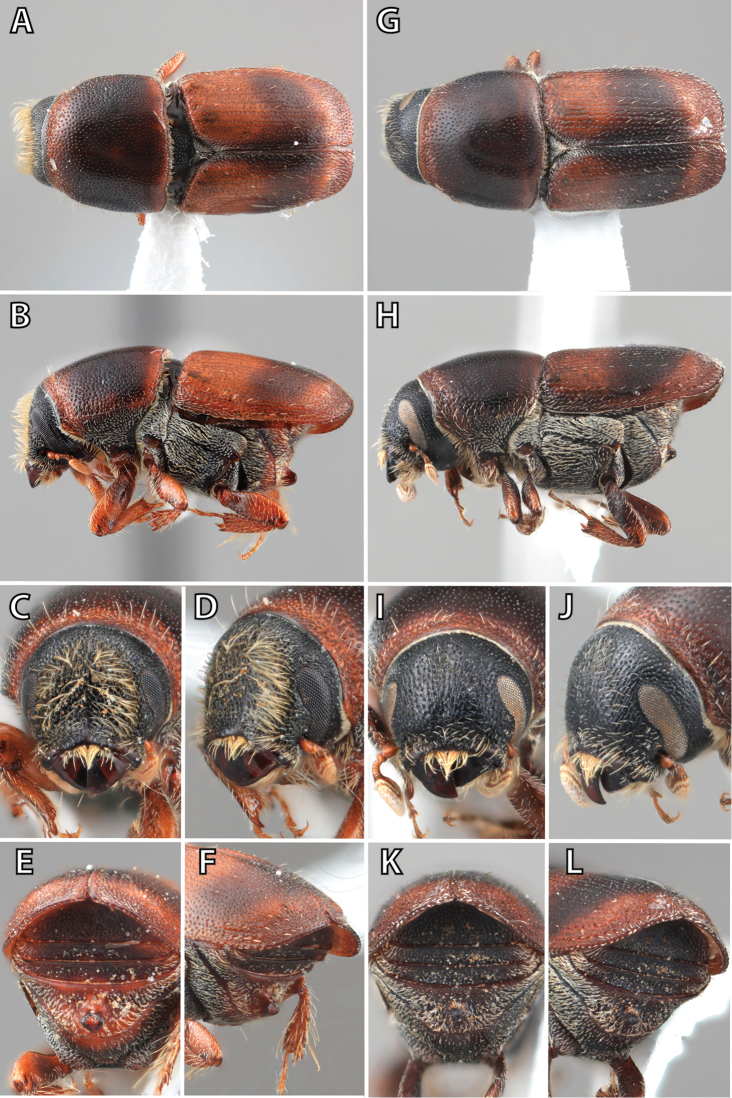
*Scolytus schevyrewi* **A** dorsal male habitus **B** lateral male habitus **C** male frons **D** male frons oblique **E** male venter **F** male venter oblique **G** dorsal female habitus **H** lateral female habitus **I** female frons **J** female frons oblique **K** female venter **L** female venter oblique.

#### Female.

2.3–3.6 mm long (mean = 3.2 mm; n = 10); 1.7–2.4 times as long as wide. Similar to male except epistoma feebly emarginated, frons flattened when viewed laterally, weakly longitudinally aciculate, setae sparser, shorter, less than width of eye; weakly transversely impressed just above epistoma and longitudinally impressed in median area. Apical margin of ventrite 1 weakly elevated above base of ventrite 2. Second ventrite armed with low median carina (variable and may be absent) that does not touch either margin.

#### Specimens examined.

24.

#### Type material.

Holotype, male (location unknown).

#### Non-type material.

**UNITED STATES:** ***CALIFORNIA*:** *Los Angeles Co.*: Whittier, Whittier Fertilizer, 7.IX.2006, A. Sanchez, ex. Lindgren funnel + ETOH & alpha pinene (DEBC-1). ***COLORADO*:** *Adams Co.*: Aurora, wood recycler, N39°807', W104°994', 9.VII.2003, P. McPherren, ex. funnel trap with Ips lure (MSUC-11), 15.IV.2003 (MSUC-1), 26.IV.2003 (MSUC-1), 2.V.2003 (MSUC-1), 29.V.2003 (MSUC-1). ***MICHIGAN*:** *Ingham Co.*: 42.736501 -84.464670, 258 m, 16.IX.2011, S.M. Smith, A.I. Cognato, I.A. Cognato, ex. *Ulmus* sp. (MSUC-1). *Wayne Co.*: Trenton-Woodhaven, 26.VII.2004, T. Dutton, ex. Lindgren funnel alpha-pinene Trap WY4 (MSUC-1). ***UTAH*:** *Weber Co.*: Ogden, Ogden Nature Center, 13.VII.2003, ex. funnel trap with alpha pinene & ethanol lure (MSUC-6).

#### Distribution.

**Figure 17.**
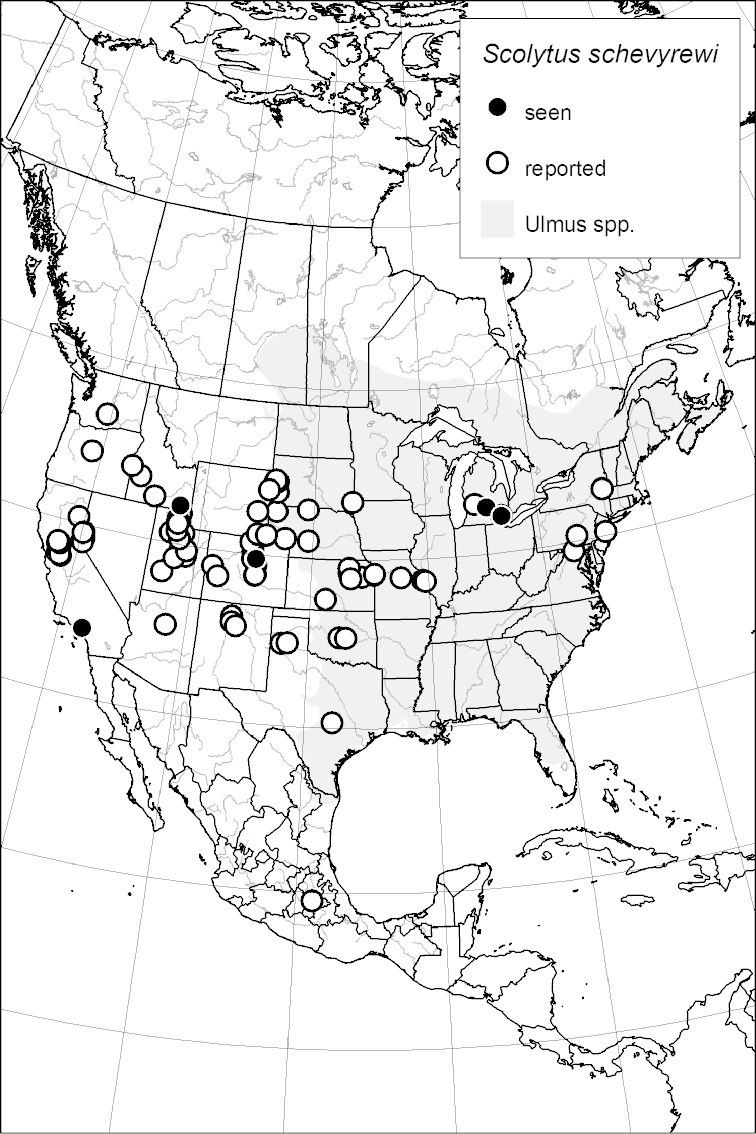
*Scolytus schevyrewi* distribution map.

CANADA: Alberta, British Columbia, Manitoba, Ontario, Saskatchewan. UNITED STATES: Arizona, California, Colorado, Connecticut, Delaware, Idaho, Illinois, Indiana, Kansas, Maryland, Michigan, Minnesota, Missouri, Montana, Nebraska, Nevada, New Jersey, New Mexico, Ohio, Oklahoma, Oregon, Pennsylvania, South Dakota, Texas, Utah, Virginia, Washington, Wyoming (Fig. 17).

#### Hosts.

*Ulmus* spp. including *Ulmus americana* L. (American elm), *Ulmus pumila* L. (Siberian elm), *Ulmus thomasii* Sarg. (rock elm) and *Ulmus procera* Salisb. (English elm).

#### Common name.

Banded elm bark beetle.

#### Biology.

This species prefers to attack drought stressed elms (Negrón et al. 2005). *Scolytus schevyrewi* locate a suitable host via host tree volatiles (Lee et al. 2010).

The adult gallery consists solely of a single egg gallery parallel with grain of the wood; a nuptial chamber is not constructed (Lee et al. 2006). The adult gallery strongly scores the sapwood. Egg niches are constructed along the gallery and score the sapwood. Twenty to 120 eggs are laid along the egg galleries (Lee et al. 2010). Larval mines lightly score the sapwood and radiate perpendicular to the egg gallery. Larval galleries later meander often at an oblique angle to the grain of wood, forming a fan shaped pattern. Pupation occurs in the outer bark and broods overwinter as mature larvae or pupae (Lee et al. 2006). In California, flight occurs from April-September or October. In Nevada, Utah, Wyoming, Colorado, Kansas and Utah, flight occurs from May to September (Lee et al. 2011). There are two to three generations per year (Lee et al. 2011). Development from egg to adult takes 30–45 days (Negrón et al. 2005). Upon emergence, adults feed at twig crotches prior to selecting host material (Negrón et al. 2005). *Scolytus schevyrewi* is a less effective vector of the Dutch elm disease fungus *Ophiostoma ulmi* in North America, especially in Rocky Mountain states (Jacobi et al. 2013).

#### Remarks.

This species is native to the Palearctic where it occurs from western Russia and Uzbekistan and east to China, Mongolia and Korea (Michalski 1973). *Scolytus schevyrewi* was first detected in North America in 2003. The first specimens were collected in Colorado and Utah. By 2005, it was recorded from 21 states suggesting that it had been present for many years before its initial detection (Negrón et al. 2005; LaBonte 2010). Subsequent review of bark beetle survey collections revealed the earlier occurrence of this species in Denver, Colorado in 1994 and Clovis, New Mexico in 1998 (Lee et al. 2006).

In areas where populations of *Scolytus schevyrewi* and *Scolytus multistriatus* are both present, the abundance of *Scolytus multistriatus* is decreasing to the point where this once common species is difficult to find (Negrón et al. 2005; Lee et al. 2010). This competitive displacement of *Scolytus multistriatus* by *Scolytus schevyrewi* is likely the result of differences in fecundity, generation time, and emergence. *Scolytus schevyrewi* produces larger broods that may overwinter as pupae, have a quicker development period, have an earlier flight, and exhibit rapid, strong aggregation to host kairomones as compared to *Scolytus multistriatus* (Lee et al. 2010).

*Scolytus schevyrewi* is a highly morphologically variable species. LaBonte (2010) provides an excellent discussion regarding intraspecific variation exhibited within *Scolytus schevyrewi*. There is considerable variation observed in the shape and appearance of spine on the second ventrite. The male second ventrite spine is typically well developed with a blunt apex that is broader than the base and appears triangular when laterally viewed. In females the spine is variously reduced and may even be absent. In most males, the spine is closest to the apical margin of the second ventrite. In some males the base of the spine is closer to basal margin. Most individuals exhibit the characteristic dark brown band on reddish colored elytra, which is the derivative of the species’.

#### Common name.

Occasionally individuals have been encountered with unicolorous dark brown or reddish elytra. Additional variation was also observed in the coloration of the pronotum. The pronotum can vary from almost entirely dark brown with reddish coloration along the margins (common form) to the entire dorsal surface being reddish with dark brown margins (uncommon form) (LaBonte 2010).

### Native Hardwood clade

The native hardwood clade (2.2–5.6 mm long) (*Scolytus fagi*, *Scolytus muticus*, and *Scolytus quadrispinosus*) is monophyletic. The sexual dimorphism of the native hardwood clade differs from the introduced group and the conifer clade as both the males and females have an impressed frons that is strongly longitudinally aciculate, except *Scolytus fagi* in which the males have a faintly longitudinally aciculate frons and the female frons is granulate punctate. This group arguably has the strongest degree of sexual dimorphism with *Scolytus quadrispinosus* and *Scolytus muticus* each possessing a strongly excavated second ventrite numerous spines and teeth or a large setal patch on ventrite 5 respectively. The abdominal venter of *Scolytus fagi* is identical in both sexes.

#### Scolytus fagi

Taxon classificationAnimaliaColeopteraCurculionidae

Walsh, 1867

Fig. 18

Scolytus fagi Walsh, 1867: 58.

##### Diagnosis.

Both sexes of *Scolytus fagi* are distinguished from *Scolytus mali* and *Scolytus muticus* by the rugose-reticulate frons that is covered by uniformly distributed setae.

##### Description (male).

3.3–5.5 mm long (mean = 4.45 mm; n = 20); 1.96–2.75 times as long as wide. Color dark red-brown to black, antenna light brown. Pronotum typically same color as elytra.

*Head.* Epistoma weakly, broadly emarginated; epistomal process absent; median area above mandibles bearing dense patch of long, yellow, hair-like setae. Frons appearing flattened when viewed laterally, slightly transversely impressed just above epistoma and along median line; rugose-reticulate, strongly punctate; punctures small, coarse; moderately, uniformly covered by long, fine, yellow erect hair-like setae, these longer than width of midpoint of eye. Antennal scape short, elongate; club flattened, thinner on apical half, irregularly ovoid, setose with partial septum, two arcuate sutures visible.

*Pronotum* wider than long; apical margin broadly rounded, median area between eyes lined with scales; sides distinctly arcuate, strongly constricted near apex, forming a weak transverse impression near apical margin; surface smooth, shining, punctulate, punctures moderately abundant, larger, coarse and more abundant laterally and on apical constriction; apical and anterolateral margins bearing sparse, erect, yellow hair-like setae; base weakly bisinuate.

*Elytra* with sides sub-parallel on apical half, narrowing to weakly rounded, smooth apex; apex weakly emarginated at suture. Margin of apical edge bearing small, fine punctures. Disc smooth, shining; interstriae not impressed, more than twice width of striae, punctures uniseriate, smaller than those of striae, punctures bearing short recumbent yellow setae slightly longer than size of a puncture (may be abraded); striae moderately impressed. Declivity bearing sparse, short, erect yellow setae. Metepimeron half-length of metanepisternum.

*Venter.* Apical margin of ventrite 1 weakly elevated above base of ventrite 2. Ventrite 2 nearly perpendicular to ventrite 1; surface smooth, shining, finely punctate; punctures large, fine, shallow; surface flattened; setae semi-recumbent, short, about half length of segment 3 or less; apical margin unarmed; lateral margins of ventrites 2–3 and ventrite 4 unarmed. Ventrite 5 unarmed; length of ventrite 5 greater than combined lengths of ventrites 3 and 4; setal patch absent, median depression present.

**Figure 18.**
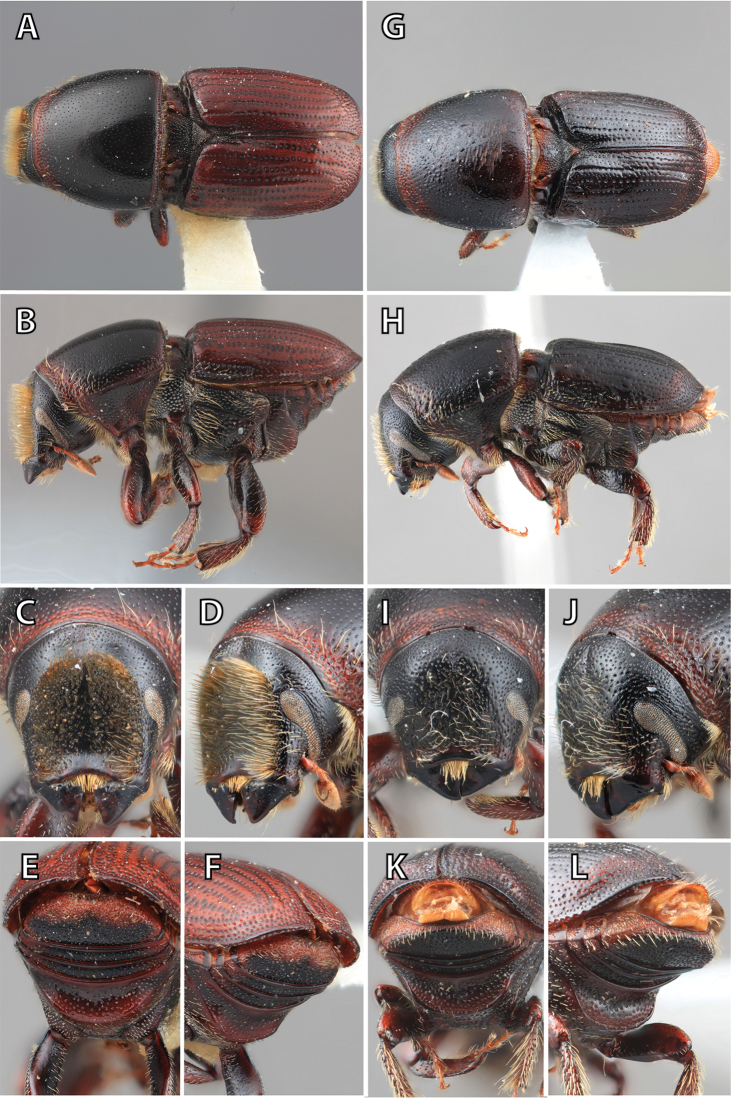
*Scolytus fagi* **A** dorsal male habitus **B** lateral male habitus **C** male frons **D** male frons oblique **E** male venter **F** male venter oblique **G** dorsal female habitus **H** lateral female habitus **I** female frons **J** female frons oblique **K** female venter **L** female venter oblique.

##### Female.

3.9–5.6 mm long (mean = 4.65 mm; n = 20); 1.9–2.8 times as long as wide. Similar to male except epistoma feebly emarginated, epistomal process absent, frons convex when viewed laterally, weakly longitudinally strigate-punctate, setae shorter, less than width of eye and sparse; weakly transversely impressed just above epistoma and between inner apices of eyes. Second ventrite unarmed.

##### Specimens examined.

74.

##### Type material.

Neotype *Scolytus fagi* Walsh: male, labeled “Galesburg, Illinois, Liebeck Collection” (MCZC). Neotype designated Smith and Cognato 2010b: 36.

##### Non-type material.

**CANADA:** ***ONTARIO*:** Point Pelee National Park, 27.IX.1989, K. Dunster, ex. *Celtis tenuifolia* (CNCI-9). **UNITED STATES:** ***MISSISSIPPI*:** [*Sharkey Co.*]: Rolling Fork, VIII.1976, J.D. Solomon, ex. Nuttall oak [= *Quercus* sp.] (USNM-1). ***PENNSYLVANIA*:** *Cumberland Co.*: Roadway Dr @ Schneider Dr, 40.229030°N, 77.111580°W, 26.VI.2009, L.R. Donovall, (MSUC-25), 29.V.2009, ex. Lindgren-alpha pinene/EtOH (MSUC-4), 29.V.2009, ex. Lindgren-EtOH (MSUC-1). *Dauphin Co.*: Wildwood on Industrial Rd, 40.316325°N, 76.888783°W, 6.VIII.2009, S.-E. Spichiger, ex. Lindgren-EtoH (MSUC-2). *Lancaster Co.*: 7031 Elizabethtown Rd, 40.182583°N, 76.498783°W, 23.VII.2009, ex. Lindgren-BEBB/EtOH (MSUC-1). *York Co.*: 400 Mundis Rd, 40.030170°N, 76.705330°W, 10.VI.2009, S. Rebert, ex. Lindgren-alpha/EtOH (MSUC-4). ***TEXAS*:** [*Colorado Co.*]: Columbus, [18]88 (MSUC-4, USNM-15). [*Unspecified county*]: (USNM-3). Fort Worth, 31.VII.[19]12, ex. bred from hackberry [= *Celtis* sp.] (USNM-4).

##### Distribution.

**Figure 19.**
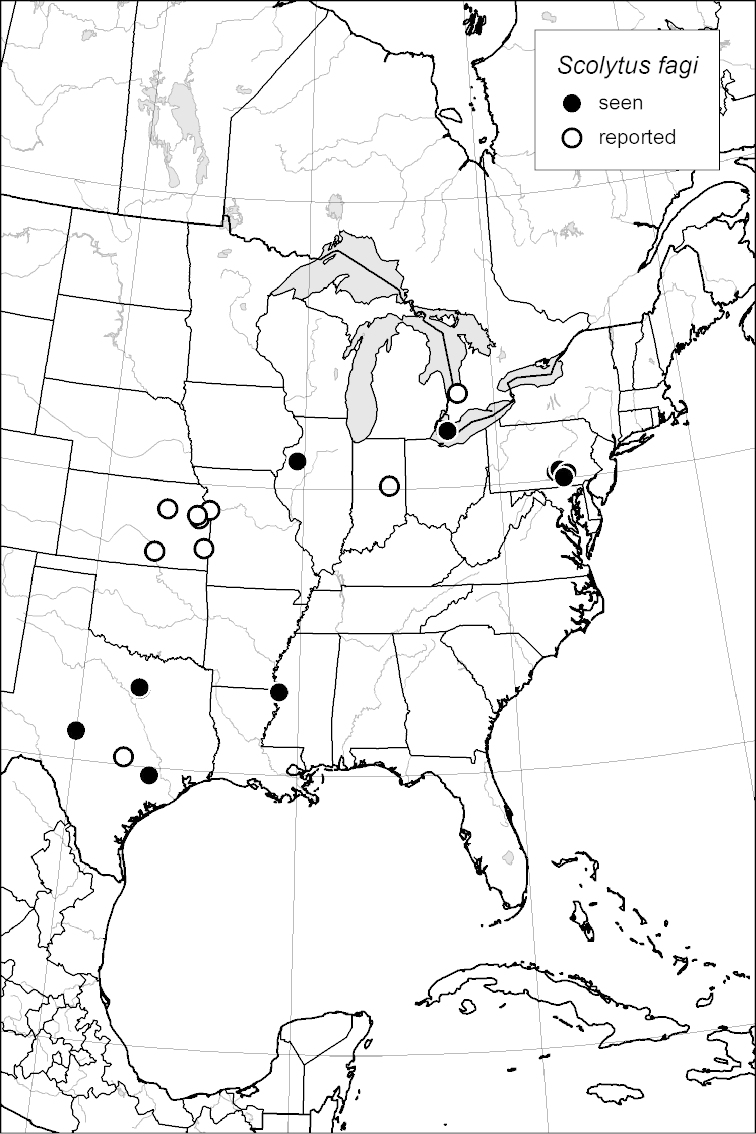
*Scolytus fagi* distribution map.

CANADA: Ontario. UNITED STATES: Illinois, Kansas, Mississippi, Ohio, Pennsylvania, Texas (Fig. 19).

##### Hosts.

*Celtis occidentalis* L. (common hackberry), *Celtis tenuifolia* Nutt. (dwarf hackberry), *Celtis laevigata* Willd. var. *texana* Sarg. (Texan sugarberry), *Fagus grandifolia* Ehrh. (American beech), and *Quercus* spp. (oak).

##### Biology.

The gallery has been reported as very confused and undecipherable. Larvae were reported boring in the wood. In addition, the species did not appear to colonize healthy trees (Packard 1890). Smith and Cognato (2010a) summarized all information known about this species.

##### Remarks.

This species is very rarely encountered. Most museum collections occurred in the early 1900’s and most recent collections have been from Lindgren funnel traps. There are many gaps in the known distribution of *Scolytus fagi* but the species likely occurs throughout the eastern US associated with its host trees.

#### Scolytus muticus

Taxon classificationAnimaliaColeopteraCurculionidae

Say, 1824

Fig. 20

Scolytus muticus Say, 1824: 182.

##### Diagnosis.

*Scolytus muticus* are differentiated from other *Scolytus* species by the presence of long, erect hair-like setae on the elytral interstriae and on the abdominal venter. The male is further distinguished by a pair of strongly elevated areas on the basal two-thirds of ventrite 5, each densely covered with abundant fine, long hair-like setae.

##### Description (male).

2.2–5.3 mm long (mean = 3.65 mm; n = 20); 1.75–2.6 times as long as wide. Head, pronotum, legs and abdominal venter dark red-brown, antennae yellow-brown, elytra usually dark red-brown but occasionally red-brown. Pronotum typically darker than elytra.

*Head.* Epistoma moderately, broadly emarginated; epistomal process absent; median area above mandibles bearing dense patch of long, yellow, hair-like setae. Frons appearing strongly flattened when viewed laterally; densely, finely, longitudinally aciculate; aciculations converging at epistoma; impunctate; setae on lateral and dorsal margins covered by long, thick, incurved, yellow erect hair-like setae, these longer than width of midpoint of eye, median areas covered with sparse, shorter and thinner setae. Antennal scape short, elongate; club flattened, irregularly ovoid, setose with partial septum, three very sharply arcuate sutures visible.

*Pronotum* wider than long; apical margin broadly rounded, median area between eyes lined with scales; sides distinctly arcuate, strongly constricted near apex, forming a weak transverse impression near apical margin; smooth, shining, punctures on disc fine, shallow, moderately abundant, larger, coarser and dense laterally and on apical constriction; apical and anterolateral margins bearing sparse, erect, yellow hair-like setae; base weakly bisinuate.

*Elytra* with sides sub-parallel on apical half, narrowing to subquadrate, smooth apex; apex entire at suture. Margin of apical edge bearing small, fine punctures. Disc reticulate, shining; interstriae faintly impressed, equal to width of striae, interstrial punctures large, uniseriate, equal in size to those of striae, bearing moderately abundant, long, semi-erect, yellow hair-like setae (may be abraded); striae moderately impressed. Declivity bearing sparse, short, erect yellow setae. Metepimeron half-length of metanepisternum.

*Venter.* Apical margin of ventrite 1 weakly elevated above base of ventrite 2. Ventrite 2 nearly perpendicular to ventrite 1; surface reticulate, shining, densely, coarsely punctured; punctures large, coarse; surface flattened; setae moderately abundant, longer than length of ventrite 3; apical margin unarmed; lateral margins of ventrites 2–3 and ventrite 4 unarmed. Ventrite 5 unarmed; length of ventrite 5 greater than combined lengths of ventrites 3 and 4; median depression absent; pair of strongly elevated areas on basal two-thirds, each densely covered with abundant fine, long hair-like setae; apical third strongly impressed.

**Figure 20.**
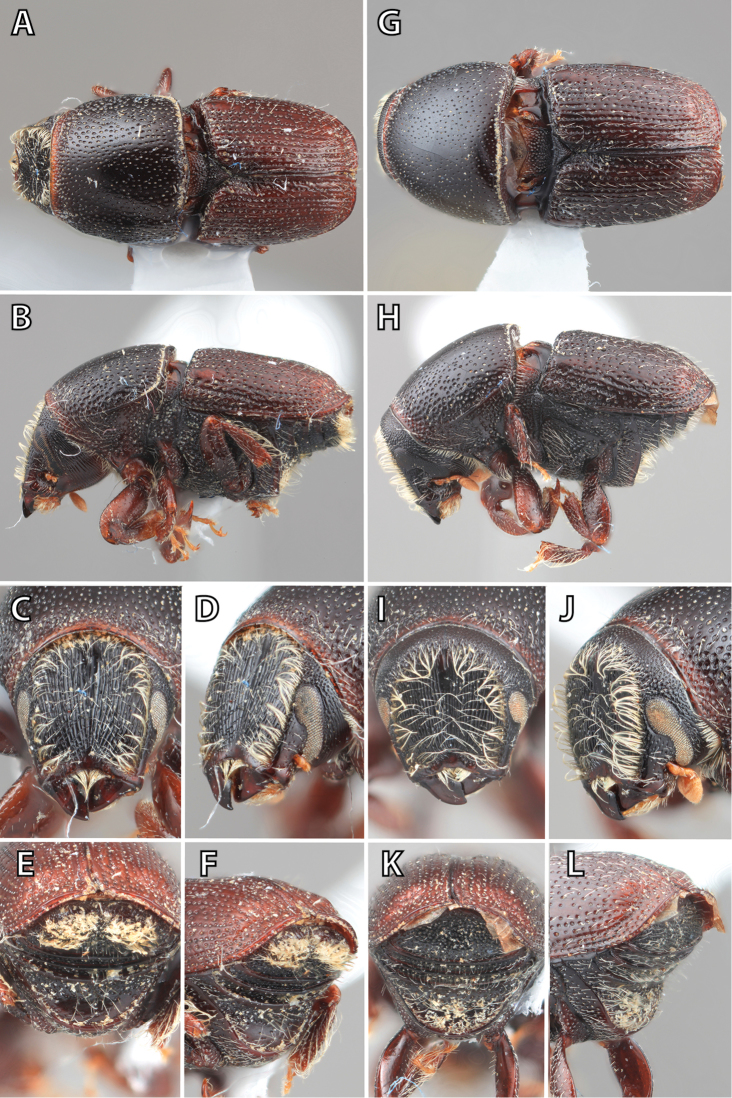
*Scolytus muticus* **A** dorsal male habitus **B** lateral male habitus **C** male frons **D** male frons oblique **E** male venter **F** male venter oblique **G** dorsal female habitus **H** lateral female habitus **I** female frons **J** female frons oblique **K** female venter **L** female venter oblique.

##### Female.

3.1–5.1 mm long (mean = 4.0 mm; n = 20); 1.96–2.45 times as long as wide. Similar to male except epistoma feebly emarginated, epistomal process absent, frons less strongly flattened when viewed laterally, finely, narrowly longitudinally aciculate, setae shorter, less than width of eye and less abundant; weakly transversely impressed between inner apices of eyes. Second ventrite unarmed. Ventrite 5 without a pair of strongly elevated areas on basal two-thirds or setal patches.

##### Specimens examined.

214.

##### Type material.

Holotype: male, Missouri (ANSP, lost).

##### Non-type material.

**CANADA:** ***ONTARIO*:** Pelee Island, 29.VI.1940, W.J. Brown (CNCI-1). Point Pelee National Park, 23.VI.[19]31, W.J. Brown (CNCI-1); 27.IX.1989, K. Dunster, ex. *Celtis tenuifolia* (CNCI-1), 2.VIII.1990 (CNCI-1). **UNITED STATES:** ***COLORADO*:** *Jefferson Co.*: Evergreen, Upper Bear Creek Rd, 11.IX.1980, D. Leatherman, ex. Douglas fir [= *Pseudotsuga menziesii*] (CSUC-1). ***ILLINOIS*:** *Alexander Co.*: Pleasant Valley, 15.VI.1979, B.C. Weber, ex. Trap 6, Ht. 2 (USNM-1), 22.VI.1979, ex. Trap 14, Ht. 4 (USNM-1). *Champaign Co.*: 28.X.1960, ex. hackberry [= *Celtis* sp.] (EMEC-4). *Sangamon Co.*: Springfield, 29.V.2003, C. Helm (CUIC-1). *Stephenson Co.*: Freeport, 4.VII.[19]17 (USNM-1). ***INDIANA*:** *Tippecanoe Co.*: 6.VI.1971, N.M. Downie, ex. *Celtis occidentalis* (USNM-6). ***IOWA*:** [*Story Co.*]: Ames, 18.VIII.1926, H.H. Harris (DEBC-2), 1.IV.1936 (DEBC-1), 22.V.1939, E. Polderboer (DEBC-1); 22.V.1939, C. Vocom (USNM-2); E. Snead (USNM-1). ***KANSAS*:** *Douglas Co.*: Lawrence, 5 mi N.E., Kansas University Natural History Research Station, 9.VII.1982, D.H. Wahl (CNCI-1). Lawrence, 10.VI.[19]20, M.W. Blackman (USNM-8); 5.IX.1950, S.L. Wood (USNM-4). *Riley Co.*: G.A. Dean (DEBC-1); Popenoe (DEBC-1). [*Unspecified County*]: Manhattan, 1.VI.[19]29, F. Kruger (DEBC-1); 5.VI.[19]29, T.N. Winburn (DEBC-1); 8.V.1968, G. Hevel (USNM-1). ***KENTUCKY*:** *Christian Co.*: 15.VI.1960, J.M. Campbell (CNCI-1). ***LOUISANA*:** *Jefferson Parish*: New Orleans, 4.XII.1975, emerged II.1976, S.G. Wellso, ex. *Celtis laevigata* wood (MSUC-13). *Saint Bernard Parish*: J.N. Knull, ex. reared from *Celtis mississippiensis* [= *Celtis tenuifolia*] (USNM-3). ***MARYLAND*:** [*Cecil Co.*]: Port Deposit, 26.VI.1977, D. Jump (USNM-1), 13.VII.1977 (USNM-1), 9.VI.1979 (USNM-2), 3.VII.1979 (USNM-1). *Montgomery Co.*: Bethesda, 25.V.1981, W.E. Steiner (USNM-1). Plummers Island, II-III.1912, E.A. Schwarz, ex. branch of *Celtis* sp. (USNM-30). ***MICHIGAN*:** *Ingham Co.*: East Lansing, Agriculture College [Michigan State University] (MSUC-8); S21 T4N R1W, VII.1970, S.G. Wellso (MSUC-12). East Lansing, IV.1972, D.K. Young (MSUC-4); 15.VI.1970, S.G. Wellso, ex. emerged from *Celtis occidentalis* (MSUC-7). Okemos, 12.VIII.1969, S.G. Wellso (MSUC-1), 5.VI.1976 (MSUC-1); 28.V.1970, S.G. Wellso, ex. *Celtis occidentalis* (MSUC-2), 10.VIII.1969 (MSUC-1). *Kalamazoo Co.*: Gourdneck Lake State Game Area, 19.VI.2011, A.I. Cognato, ex. funnel trap with EtOH (MSUC-1). *Wayne Co.*: Grosse Ile, Pke [sic! = Parke] lane, N42.17060°, W84.14496°, 23.V–7.VI.2007, R. Mech, ex. Lindgren trap with ipslure (MSUC-1), ex. Lindgren trap with EtOH + alpha (MSUC-1), 20.VI-6.VII.2007, ex. Lindgren trap with ipslure (MSUC-1), 7-20.VI.2007 (MSUC-5). ***MISSISSIPPI*:** [*Unspecified County*]: 19.V.1920, M.W. Blackman (CNCI-1). ***MISSOURI*:** [*Boone Co.*]: Columbia, 24.IV.1954, P.J. Spangler (USNM-1). ***NEBRASKA*:** *Dixon Co.*: Ponca, Ponca State Park, 42.607161°N, -96.73223°W, 23.VIII.2007, T.P. Miller, ex. funnel trap with PSB alpha-pinene (MSUC-1). *Knox Co.*: Crofton, Lewis and Clarke Lake, 42.8321983°N -97.575555°W, 5.IX.2007, T.P. Miller, ex. funnel trap with Sirex (MSUC-1). [*Lancaster Co.*]: Lincoln, 29.VIII.[19]53, R. Roselle, ex. elm [= *Ulmus* sp.] (USNM-1). *Sarpy Co.*: Fontenelle Forest, 41.171478°N -95.9068166°W, 30.V.2006, N. Haxton, ex. funnel trap with ethanol lure (MSUC-1), 7.VII.2006 (MSUC-1). ***NEW JERSEY*:** [*Burlington Co.*]: Riverton, 12.II.1934, Wadley, ex. on Hackberry [= *Celtis* sp.] (USNM-1). [*Essex Co.*]: Newark (USNM-1). [*Somerset Co.*]: North Branch, 9.III.1937, C.H. Hoffman, ex. from gallery made in honey locust [= *Gleditsia triacanthos*] (USNM-1). ***NEW YORK*:** [*Tompkins Co.*]: Ithaca, 18.VI.[19]59, ex. in *Celtis occidentalis* (CUIC-11). ***NORTH CAROLINA*:** *Mecklenburg Co.*: Charlotte, 15-20.VI.2006, J.F. Cornell, ex. FIT in vacant lot (MSUC-1). ***NORTH DAKOTA*:** [*Ransom Co.*]: Mcleod, 5 mi N.W., 4.VII.1968, H.F. Howden (CNCI-1). ***OHIO*:** [*Franklin Co.*]: Columbus (USNM-1); 11.V.[19]14 (DEBC-2). [*Hamilton Co.*]: Cincinnati, 6.VI.[?] (USNM-1), 24.VI.[?] (USNM-1), 9.VII.[?] (USNM-1). ***OKLAHOMA*:** *Latimer Co.*: V.1982, K. Stephan (USNM-2). ***PENNSYLVANIA*:** [*Allegheny Co.*]: Allegheny [= Pittsburgh], 7.VIII.[18]95 (CUIC-12). [*Dauphin Co.*]: Harrisburg, T.H. Hubbell (CUIC-1). Hummelstown, 26.VI.[19]37, J.N. Knull (CUIC-6). ***TEXAS*:** *Brazos Co.*: College Station, 13.IV.1964, S.G. Wellso (MSUC-1), 26.IV.1964 (MSUC-1). [*Unspecified County*]: Dallas, Hopk. U.S. 9929-X, F.C. Bishop, ex. hackberry [= *Celtis* sp.] (USNM-5). ***VIRGINIA*:** *Clarke Co.*: Boyne, 2 mi S., U[niversity of] Virginia Blandy Experimental Farm, 8-18.VI.1990, D.R. Smith, ex. malaise trap (USNM-1), 19-30.VI.1990 (USNM-1), 1-12.VII.1990 (USNM-1). *Essex Co.*: Dunnsville, 1 mi S.E., 37°52’N, 76°48’W, 29.V-9.VI.1993, D.R. Smith, ex. malaise trap (USNM-1); 24.VI-9.VII.1992 (USNM-1). *Fairfax Co.*: Mount Vernon, 27.VI.1915, ex. *Cornus stricta* (USNM-1). [*Unspecified County*]: Falls Church, 4.III.1921, E.A. Chapin (USNM-1). ***WASHINGTON, D.C.*:** 21.V.1908 (MSUC-5). ***WEST VIRGINIA*:** *Morgan Co.*: near Great Cacapon, 25.V.1985, G.F. & J.F. Hevel (USNM-2). *Wood Co.*: Hopk. U.S. 6675, ex. *Celtis* (USNM-1).

##### Distribution.

**Figure 21.**
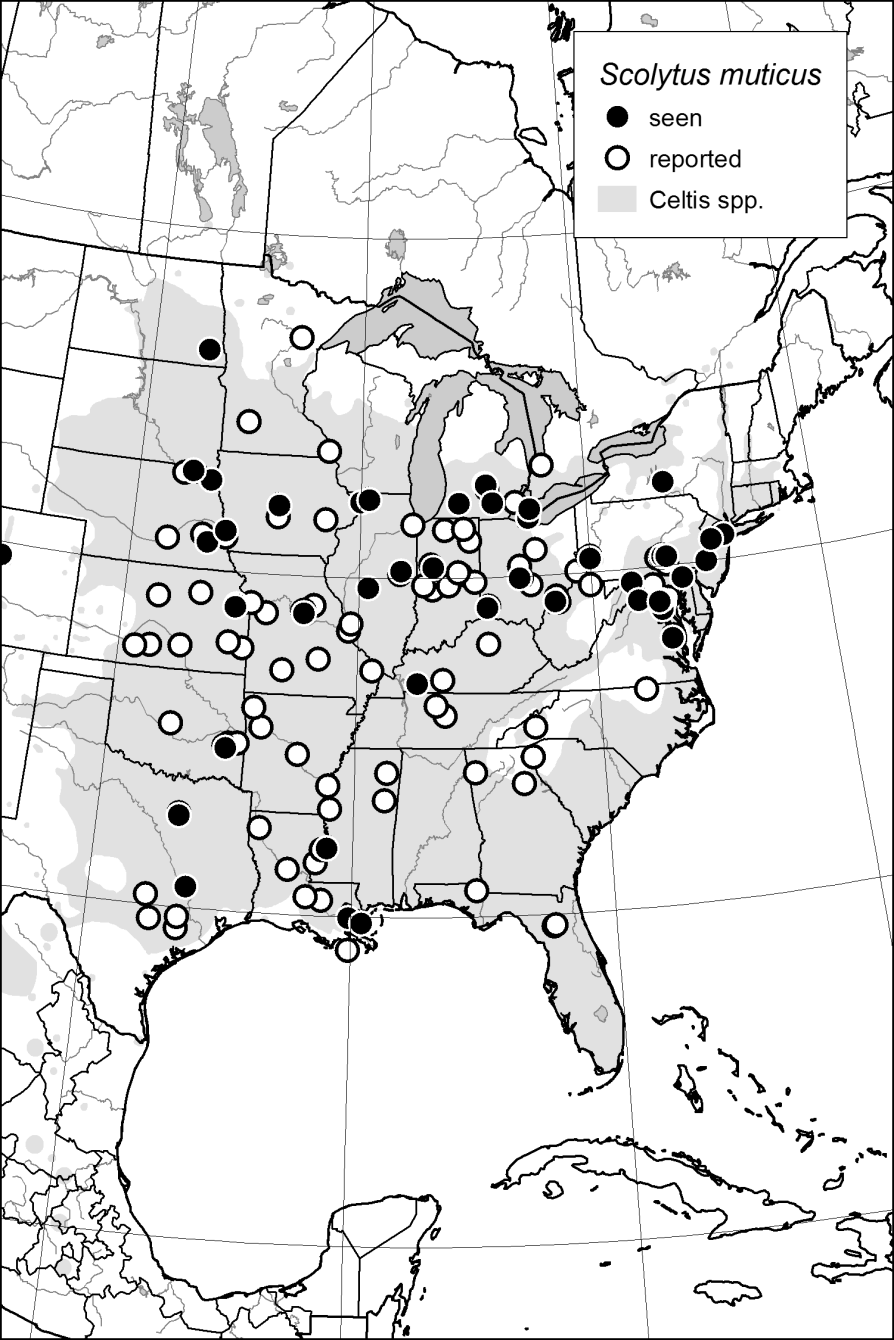
*Scolytus muticus* distribution map.

CANADA: Ontario. UNITED STATES: Colorado, Florida, Iowa, Illinois, Indiana, Kansas, Kentucky, Louisiana, Maryland, Michigan, Mississippi, Missouri, Nebraska, New Jersey, New York, North Carolina, North Dakota, Ohio, Oklahoma, Pennsylvania, South Carolina, Tennessee, Texas, Virginia, Washington, D.C., West Virginia (Fig. 21).

##### Hosts.

*Celtis occidentalis* L. (common hackberry), *Celtis tenuifolia* Nutt. (dwarf hackberry), *Celtis laevigata* Willd. (sugar hackberry) and *Gleditsia triacanthos* L. (honeylocust).

##### Common name.

Hackberry engraver.

##### Biology.

*Scolytus muticus* prefers to colonize dead, dying or felled trees and the broken, dead and dying branches of live hackberry (Blackman 1922; Doane et al. 1936; Bright 1976).

The biology of this species is poorly understood. Adult galleries are parallel to the grain of the wood and consist of a single egg gallery; a nuptial chamber is not constructed. The adult gallery strongly scores the sapwood and ranges in size from 2.5–5.0 cm in length (Blackman 1922). Larval mines first radiate perpendicular to the egg gallery and later meander, giving the galleries a tortuous appearance (Blackman 1922; Bright 1976). Larvae construct pupal chambers in burrows 2.0–3.0 cm deep within the sapwood (Bright 1976; Smith and Cognato, pers. obs.). There are two generations per year in the southeastern US and one generation per year in the northeastern US (Blackman 1922; Baker 1972).

##### Remarks.

The holotype of *Scolytus muticus* has been lost (Wood 1982), however, Say’s (1824) description is unambiguous as to the characteristics of this species.

Wood (1982) reports *Scolytus muticus* as having a transverse gallery that is perpendicular to the grain of the wood. Other authors, including Blackman (1922), Baker (1972), and Bright (1976) report this species as having a longitudinal gallery that is parallel to the grain of the wood. After examining the literature and photographs provided by T.H. Atkinson, it appears that Wood erred in his description and the gallery is parallel to the grain of the wood.

#### Scolytus quadrispinosus

Taxon classificationAnimaliaColeopteraCurculionidae

Say, 1824

Fig. 22

Scolytus quadrispinosus Say, 1824: 182.
Scolytus carya Riley, 1867: 68. LeConte 1876: 371.
Scolytus caryae Walsh, 1867: 58. LeConte 1876: 371.

##### Diagnosis.

The *Scolytus quadrispinosus* male is easily distinguished by the autapomorphic features of the abdominal venter which include: the apical margin of ventrite 3 armed by three acute spines (two lateral and one medial), the apical margin of ventrite 4 armed by one median tooth, ventrite 1 apically descending, ventrite 2 deeply concave, with the basal margin produced and bearing a median tubercle. The female is distinguished by the flattened and moderately, finely longitudinally aciculate frons, bearing long, fine, incurved setae on the lateral and dorsal margins.

##### Description (male).

2.8–4.8 mm long (mean = 4.0 mm; n = 20); 1.8–2.25 times as long as wide. Color red-brown to dark red-brown. Pronotum typically darker than elytra.

*Head.* Epistoma weakly emarginated; epistomal process strongly produced, moderately elevated, smooth, shining; median area above mandibles bearing a dense patch of long, yellow, hair-like setae. Frons appearing strongly flattened when viewed laterally; strongly, densely, coarsely, aciculate-punctate; aciculations converging at epistoma; punctures small, coarse; surface moderately covered by long, fine, yellow, erect hair-like setae, these longer than width of midpoint of eye, setae on lateral and dorsal margins longer, thicker, incurved. Antennal scape short, elongate; club flattened, irregularly ovoid, setose with partial septum, two arcuate sutures visible.

*Pronotum* wider than long; apical margin broadly rounded, median area between eyes lined with scales; sides distinctly arcuate, strongly constricted near apex, forming a weak transverse impression near apical margin; surface smooth, shining, punctures on disc fine, shallow, moderately abundant, larger and more abundant laterally and on apical constriction; apical and anterolateral margins bearing sparse, erect, golden setae; base weakly bisinuate.

*Elytra* with sides sub-parallel on apical half, narrowing to weakly rounded, serrate apex; apex entire at suture. Margin of apical edge bearing large, coarse punctures.. Disc smooth, shining; interstriae weakly impressed, twice width of striae, punctures large, uniseriate, smaller than those of striae, interstrial punctures bearing sparse, long, semi-erect yellow hair-like setae (may be abraded); striae moderately impressed. Declivity bearing sparse, short, erect yellow setae. Metepimeron half-length of metanepisternum.

*Venter.* Apical margin of ventrite 1 descending, strongly, acutely produced; ventrite 2 deeply concave, basal margin produced, bearing median tubercle. Ventrite 2 nearly perpendicular to ventrite 1; surface shagreened, dull, finely, obscurely punctate with small, fine, shallow punctures; apical margin armed with broad median denticle, occasionally absent. Apical margin of ventrite 3 armed by three acute spines (two lateral and one medial); apical margin of ventrite 4 armed by one median tooth. Ventrite 5 carinate ridge closer to basal margin of segment; length of ventrite 5 less than combined lengths of ventrites 3 and 4; median depression absent, apical half of segment pubescent.

**Figure 22.**
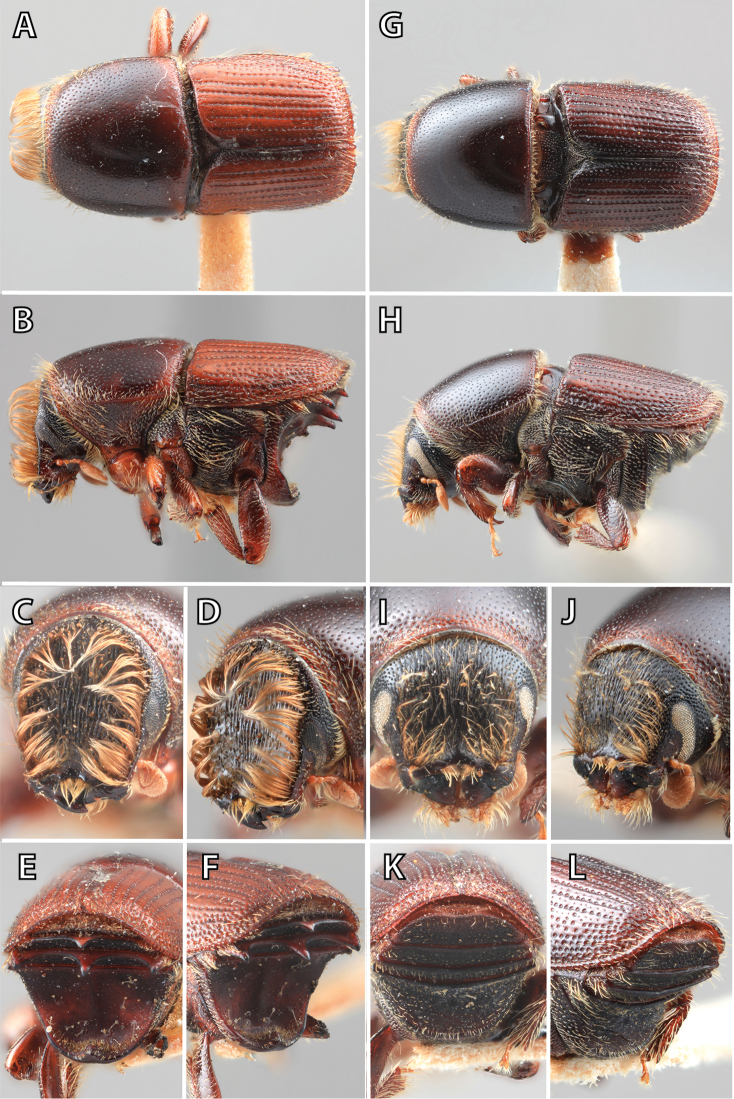
*Scolytus quadrispinosus* **A** dorsal male habitus **B** lateral male habitus **C** male frons **D** male frons oblique **E** male venter **F** male venter oblique **G** dorsal female habitus **H** lateral female habitus **I** female frons **J** female frons oblique **K** female venter **L** female venter oblique.

##### Female.

2.9–5.0 mm long (mean = 3.9 mm; n = 20); 1.8–2.4 times as long as wide. Similar to male except epistoma feebly emarginated, frons less strongly flattened when viewed laterally, moderately and finely aciculate, setae shorter, less than width of eye and uniformly distributed. Apical margin of ventrite 1 weakly elevated above base of ventrite 2. Ventrite 2 surface flattened, nearly perpendicular to ventrite 1; setae erect, short, about half length of segment 3. Venter unarmed.

##### Specimens examined.

143.

##### Type material.

Holotype *Scolytus quadrispinosus* Say: male, Missouri (ANSP, lost). Holotype *Scolytus carya* Riley, location unknown. Holotype *Scolytus caryae* Walsh, location unknown.

##### Non-type material.

**CANADA:** ***ONTARIO*:** Queenston, 15.VII.1938, D.F. Patterson, ex. *Carya ovata* (CNCI-2); Z17 E464 N4820, 20.VII.2004, Seaforth, ex. handpicked from eastern white pine [= *Pinus strobus*], 04-5-0265 (CNCI-1). **UNITED STATES:** ***GEORGIA*:** 1.IX.[19]45 (CASC-4). ***ILLINOIS*:** [*Cook Co.*]: Edgebrook (CNCI-2). [*Unspecified County*]: Willow Springs, 21.VIII.[19]04 (FMNH-1), 13.V.[19]05, A.B Wolcott (FMNH-4). ***KANSAS*:** [*Shawnee Co.*]: Topeka, 16.VI.[?], Popenoe (USNM-1). ***MASSACHUSSETS*:** [*Unspecified County*]: (CASC-1). ***MICHIGAN*:** [*Ingham Co.*]: East Lansing, 15.VII.1932, (MSUC-4). *Wayne Co.*: Detroit, 18.VIII.1902 (MSUC-3). [*Unspecified County*]: Lansing, 9.IX.1929 (MSUC-36). ***MISSOURI*:** *Dent Co.*: 28.VIII.1973, M.P. Rolling (USNM-1), 31.VIII.1973 (USNM-3). [*Unspecified County*]: (FMNH-1). ***MINNESOTA*:** [*Sherburne Co.*]: Elk River, 8.VII.1959, E.J. Kingsley (CNCI-2). ***Mississippi*:** [*Madison Co.*]: Canton, 16.VI.[19]04 (CUIC-1). [*Oktibbeha Co.*]: [Starkville], Agriculture College of Mississippi [= Mississippi State University], 15.IV.1922, F.M. Hull (CUIC-1). ***New jersey*:** [*Middlesex Co.*]: Dunellen (CUIC-1). ***NEW YORK*:** *Onondaga Co.*: 10.VI.1942, N.M. Downie (FMNH-2), 14.VII.1946 (FMNH-1). Syracuse, C.J. Drake, ex. *Hickoria glabra* [= *Carya glabra*] (USNM-1). [*Orange Co.*]: Highland Falls, 20.VI.1920, F. Schott (CUIC-1). Middletown (CUIC-1). [*Queens Co.*]: Long Island Aqueduct, 14.VII.1912 (MSUC-4). [*Tompkins Co.*]: Groton, 7.VII.1946, N.M. Downie (FMNH-1). Ithaca, 4.VIII.1928, P.P. Babiy (CUIC-1). [*Westchester Co.*]: Mount Vernon, VII.1913, ex. from hickory [= *Carya* sp.] (CASC-14). Yonkers, 28.V.1935, P.A. Readio, H. Dietrich, ex. taken on air trap (CUIC-1). [*Unspecified County*]: New York City, 15.V.1912, (CNCI-1), 13.V.1912 (CUIC-1). ***north carolina*:** [*Buncombe Co.*]: Asheville, Bent Creek, 17.VI.[19]29 (FMNH-1). ***Pennsylvania*:** [*Allegheny Co.*]: Allegheny [= Pittsburgh] (FMNH-3), 24.VI.[18]93 (CUIC-1). [*Armstrong Co.*]: Ford City, 28.VIII.[19]11 (USNM-1). [*Blair Co.*]: Tyrone, VII.[19]12, lot 367, ex. *Fraxinus alba* [= *Fraxinus americana*] (CASC- 7). *Cumberland Co.*: Roadway Dr @ Schneider Dr, 40.229030°N, -77.111580°W, 26.VI.2009, L.R. Donovall (MSUC-1). [*Dauphin Co.*]: Harrisburg, 19.IV.1911 (CASC-1), VI.1911 (CASC-3); 1.III.[19]11, emerged 7.IV.[19]11, Champlain (CASC-12); Hopk. U.S. 10935-E, 13-14.VII.[?], W.S. Fisher, ex. *Hicoria* [= *Carya* sp.] (CUIC-1). Hummelstown, 20.VI.[19]15 (CUIC-1). Linglestown, 8.VI.1912, W.S. Fisher (CNCI-1, CUIC-2). [*Philadelphia Co.*]: Angora [= Philadelphia], IX.[19]15, H.A. Kaeber, ex. Hickory bark [= *Carya* sp.] (USNM-7). [*Westmoreland Co.*]: Jeannette, H.G. Klages (CASC-2). ***TENNESSEE*:** [*Hamilton Co.*]: Chattanooga, 2.VI.[19]19, Leach (FMNH-1).

##### Distribution.

**Figure 23.**
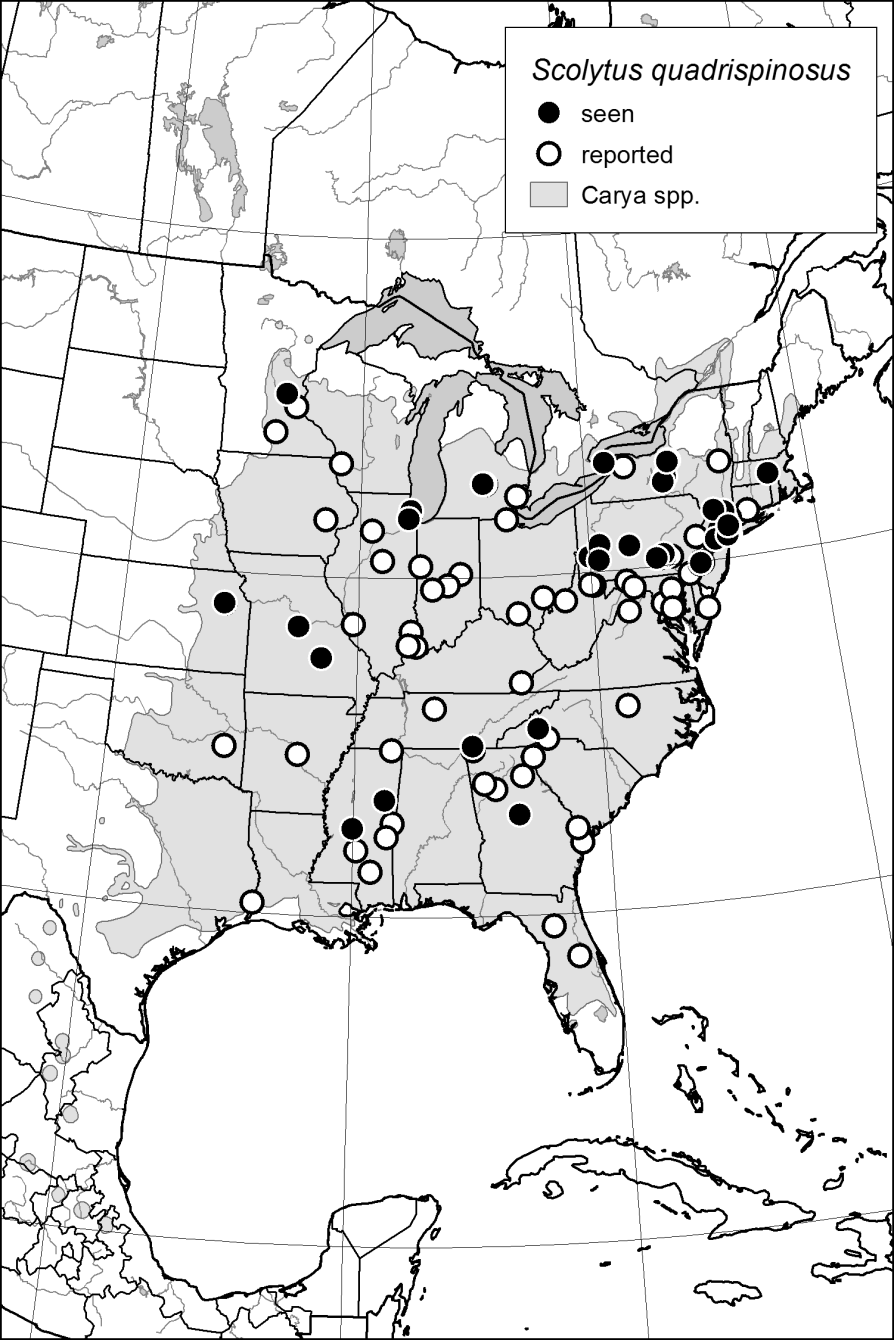
*Scolytus quadrispinosus* distribution map.

CANADA: Ontario, Quebec. UNITED STATES: Alabama, Connecticut, Delaware, Florida, Georgia, Illinois, Indiana, Iowa, Kansas, Kentucky, Louisiana, Maryland, Massachussets, Michigan, Minnesota, Mississippi, Missouri, New Jersey, New York, North Carolina, Ohio, Pennsylvania, South Carolina, Ohio, Pennsylvania, South Carolina, Tennessee, Texas, Virginia, West Virginia, Washington, D.C., Wisconsin (Fig. 23).

##### Hosts.

Primary hosts: *Carya* spp. (hickory) including *Carya illinoinensis* (Wangenh.) K. Koch (pecan). Incidental hosts: *Juglans cinerea* L. (butternut).

##### Common name.

Hickory bark beetle.

##### Biology.

*Scolytus quadrispinosus* is one of the most destructive pests of hardwoods in the US and the most important pest of hickory (Doane et al. 1936; Baker 1972). The species generally attacks and kills single trees, or solely treetops. However, outbreaks can develop during periods of drought, killing large tracts of hickory. *Scolytus quadrispinosus* kills its host by mass attack in which a multitude of broods develop in the phloem and cambium, effectively girdling the host tree (Blackman 1922).

Adult galleries are parallel to the grain of the wood and deeply score the sapwood; a nuptial chamber is not constructed. The adult gallery is short (2.5–5.0 cm), and consists of a single egg gallery (Blackman 1922). Eggs are deposited singly in niches on each side of the egg gallery with 20–60 niches per gallery (Blackman 1922). After the eggs have been laid, the female constructs a postovipositional feeding tunnel parallel to the egg gallery (Goeden and Norris 1965b). Larval mines are excavated in the cambium. From the egg gallery, the larval mines are first perpendicular to the grain of the wood and then gradually turn and diverge creating a fan shaped appearance. The larvae bore into the inner bark to overwinter. Larvae pupate the following spring and emerge as adults the following summer (Blackman 1922). Upon emergence, adults feed at twig crotches and leaf petioles before selecting a host (Baker 1972; Goeden and Norris 1964b). There is one generation per year in the north with larvae completing their development in March and April and emergence in May. There may be two generations per year in the south with the brood overwintering as larvae (Doane et al. 1936). See Goeden and Norris (1964a,b, 1965a,b) for more information regarding the biology of this species.

##### Remarks.

The holotype of *Scolytus quadrispinosus* is lost (Wood 1982), however Say’s (1824) description is unambiguous as to the characteristics of this species. The holotype of *Scolytus caryae* Walsh was likely deposited in Walsh’s type collection, which was housed in the Chicago Academy of Science Museum. This collection burned in the Great Chicago Fire and the holotypes were lost (Sheppard 2004; J. Colby, pers. comm.).

### Conifer clade

**Figure 24.**
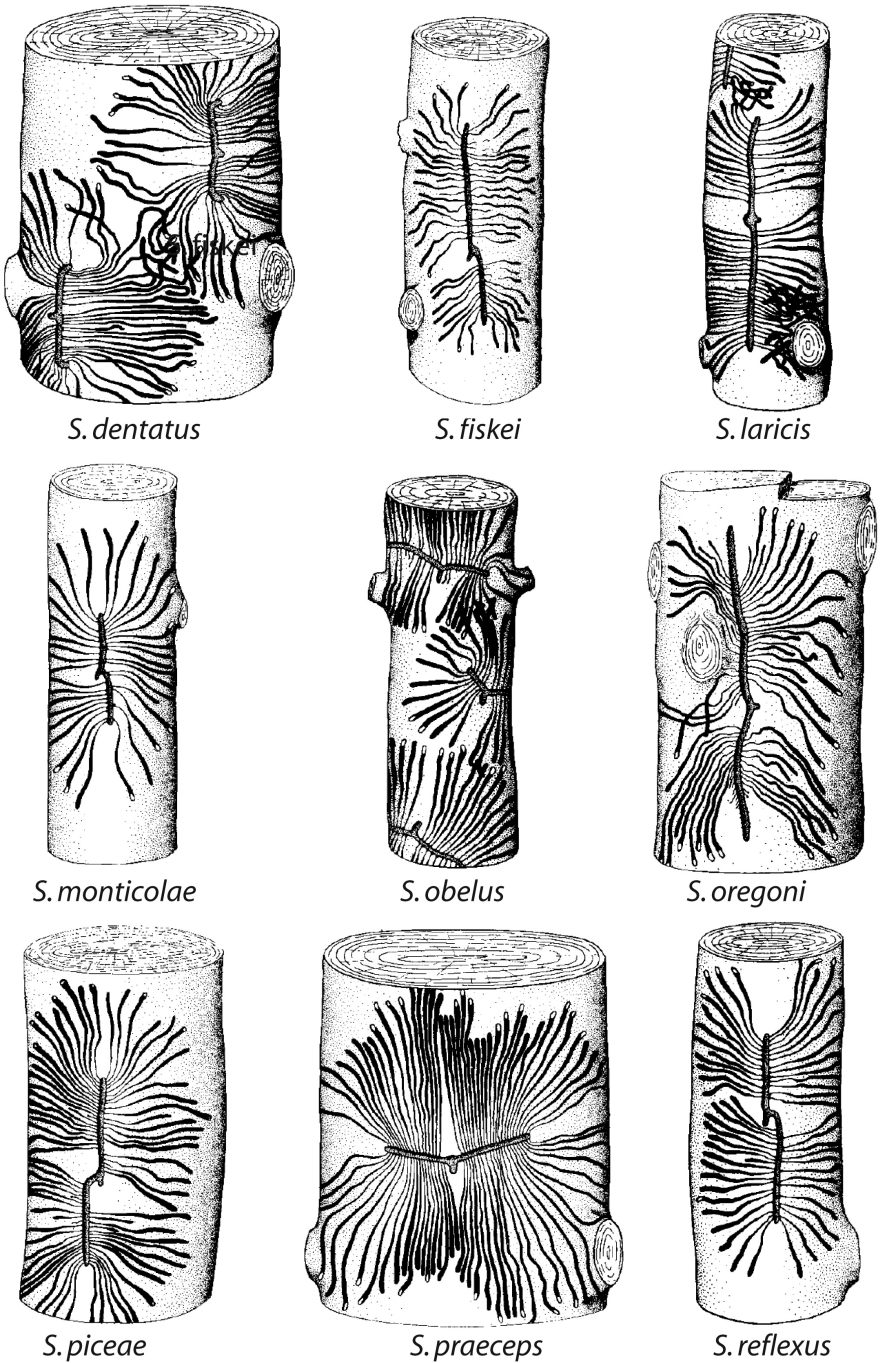
Galleries of conifer-feeding *Scolytus*, from Edson 1967.

**Figure 25.**
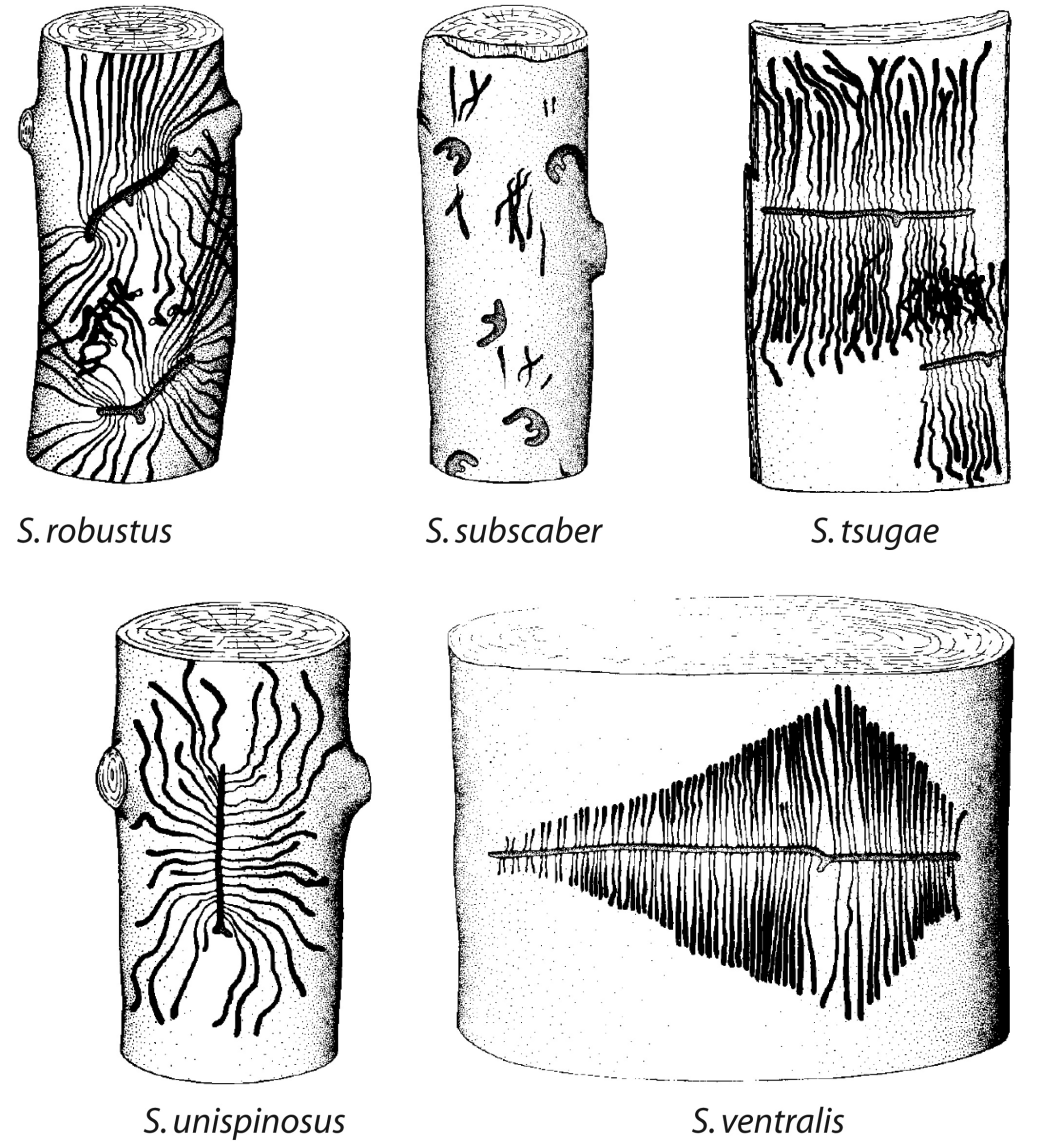
Galleries of conifer-feeding *Scolytus*, from Edson 1967.

The conifer clade (1.7–5.9 mm) (*Scolytus aztecus*, *Scolytus dentatus*, *Scolytus fiskei*, *Scolytus hermosus*, *Scolytus laricis*, *Scolytus monticolae*, *Scolytus mundus*, *Scolytus obelus*, *Scolytus oregoni*, *Scolytus piceae*, *Scolytus praeceps*, *Scolytus reflexus*, *Scolytus robustus*, *Scolytus silvaticus*, *Scolytus subscaber*, *Scolytus tsugae*, *Scolytus unispinosus*, and *Scolytus ventralis*) is monophyletic. The sexual dimorphism exhibited by species in the clade follows the general pattern outlined above except for the shape of the frons, which is quite variable. In some species, the frons is convex in both sexes with the female frons more strongly convex than that of the male (*Scolytus hermosus*, *Scolytus obelus*, *Scolytus praeceps*, and *Scolytus subscaber*). *Scolytus laricis* males have a flattened and impressed frons when viewed from a lateral profile and that of the female is convex. In the remaining species, the male frons appears flattened when viewed from a lateral profile. Galleries of the conifer clade are in Figures 24 and 25.

#### Scolytus aztecus

Taxon classificationAnimaliaColeopteraCurculionidae

Wood, 1967

Fig. 26

Scolytus aztecus Wood, 1967: 120.

##### Diagnosis.

*Scolytus aztecus* are distinguished by the characteristics of the elytral apex with the apical margin produced between interstriae 1 and 2, deeply emarginated at interstriae 3, produced on interstriae 4 and deeply emarginated at stria 4.

##### Description (male).

5.0 mm long (mean = 5.0 mm; n = 2); 2.5 times as long as wide. Body dark red-brown to black, antennae light brown, legs light brown to dark red-brown. Pronotum typically darker than elytra.

*Head.* Epistoma broadly, deeply emarginated; epistomal process weakly developed, low; median area above mandibles bearing dense patch of long yellow hair-like setae. Frons appearing flattened when viewed laterally, slightly transversely impressed just above epistoma; surface moderately, coarsely longitudinally aciculate-punctate; aciculations converging at epistoma; punctures large, coarse and more dense medially above epistoma; surface moderately and uniformly covered by long, fine, yellow erect hair-like setae, setae longer than width of midpoint of eye. Antennal scape short, elongate; club flattened, almost subquadrate, setose with partial septum, two arcuate sutures visible.

*Pronotum* as long as wide; apical margin broadly rounded, area between eyes lined with scales; sides distinctly arcuate, strongly constricted near apex, forming a weak transverse impression near apical margin; surface smooth, shining, punctures on disc fine, shallow and moderately abundant, larger, more abundant laterally and on apical constriction; apical, anterolateral and lateral margins bearing moderately abundant, erect, yellow hair-like setae; base weakly bisinuate.

*Elytra* with sides sub-parallel on apical half, narrowing to rounded, serrate apex; apical margin of elytra produced between interstriae 1 and 2, deeply emarginated at interstria 3, produced on interstria 4 and deeply emarginated at stria 4; apical margin of elytral apices bearing large, coarse punctures; apex moderately emarginated at suture. Disc smooth, shining; interstriae bearing short, sparse yellow hair-like setae spaced by length of a setae or less; interstriae weakly impressed, more than twice width of striae, interstrial punctures uniseriate, smaller than those of striae; striae moderately impressed. Declivity bearing sparse, short, erect yellow setae. Metepimeron half-length of metanepisternum.

*Venter.* Apical margin of ventrite 1 weakly elevated above base of ventrite 2. Ventrite 2 nearly perpendicular to ventrite 1; surface rugose, coarsely, deeply punctate; covered with abundant erect setae that are greater than length of segment 3; surface convex; armed medially with laterally compressed spine with base extending from apical margin to ¾ of segment, apex rounded, shining, impunctate; lateral margins of ventrites 2–3 and ventrite 4 unarmed. Ventrite 5 carinate ridge closer to basal margin of segment; length of ventrite 5 greater than combined lengths of ventrites 3 and 4; setal patch and median depression absent.

**Figure 26.**
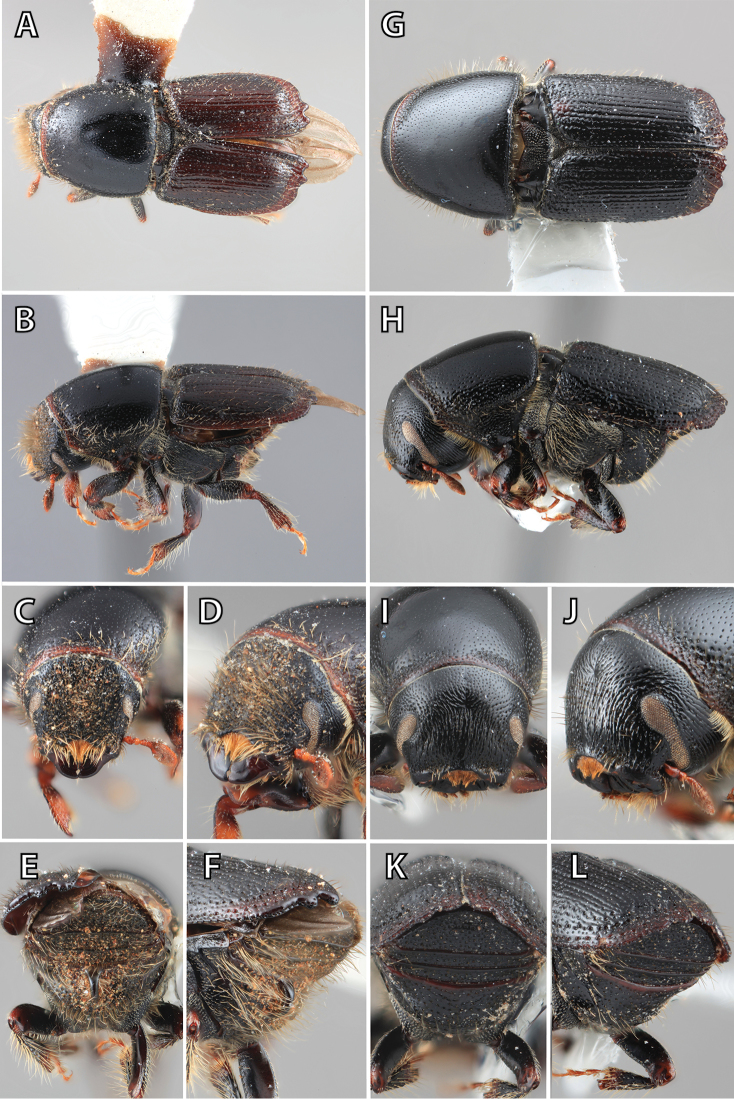
*Scolytus aztecus* **A** dorsal male habitus **B** lateral male habitus **C** male frons **D** male frons oblique **E** male venter **F** male venter oblique **G** dorsal female habitus **H** lateral female habitus **I** female frons **J** female frons oblique **K** female venter **L** female venter oblique.

##### Female.

4.6–5.9 mm long (mean = 5.23 mm; n = 4); 2.3–2.5 times as long as wide. Similar to male except epistoma feebly and broadly emarginated, epistomal process absent, frons convex when viewed laterally, weakly longitudinally aciculate, setae sparse, shorter, less than width of eye; frons weakly transversely impressed on lateral margins above epistoma and at vertex; median line on frons raised. Apical margin of elytra slightly produced between interstriae 1 and 2, emarginated at interstria 3, slightly produced on interstria 4 and emarginated at stria 4. Second ventrite unarmed.

##### Specimens examined.

11.

##### Type material.

Holotype *Scolytus aztecus* Wood: female, labeled “27 mi E. Morelia, Mich., VI-4-1965, Mexico, 8,000 ft, S.L. Wood, *Abies religiosa*” (USNM). Paratypes: **MEXICO:** ***Michoacán*:** 27 mi E. Morelia, 14.VI.1965, S.L. Wood, ex. *Abies religiosa* (USNM-3).

##### Non-type material.

**MEXICO:** ***DURANGO*:** Potreyo del Sauto y Cebadillas, 19.IV.[19]90, J. Tulio Mendez M., 935-A, ex. *Pseudotsuga menziesii* (THAC-4). ***Nuevo León*:** Santa Catarina, San Antonio de las Alazanas, 14.XII.2011, Gerardo Cuellar, ex. *Pseudotsuga menziesii* (MSUC-3).

##### Distribution.

**Figure 27.**
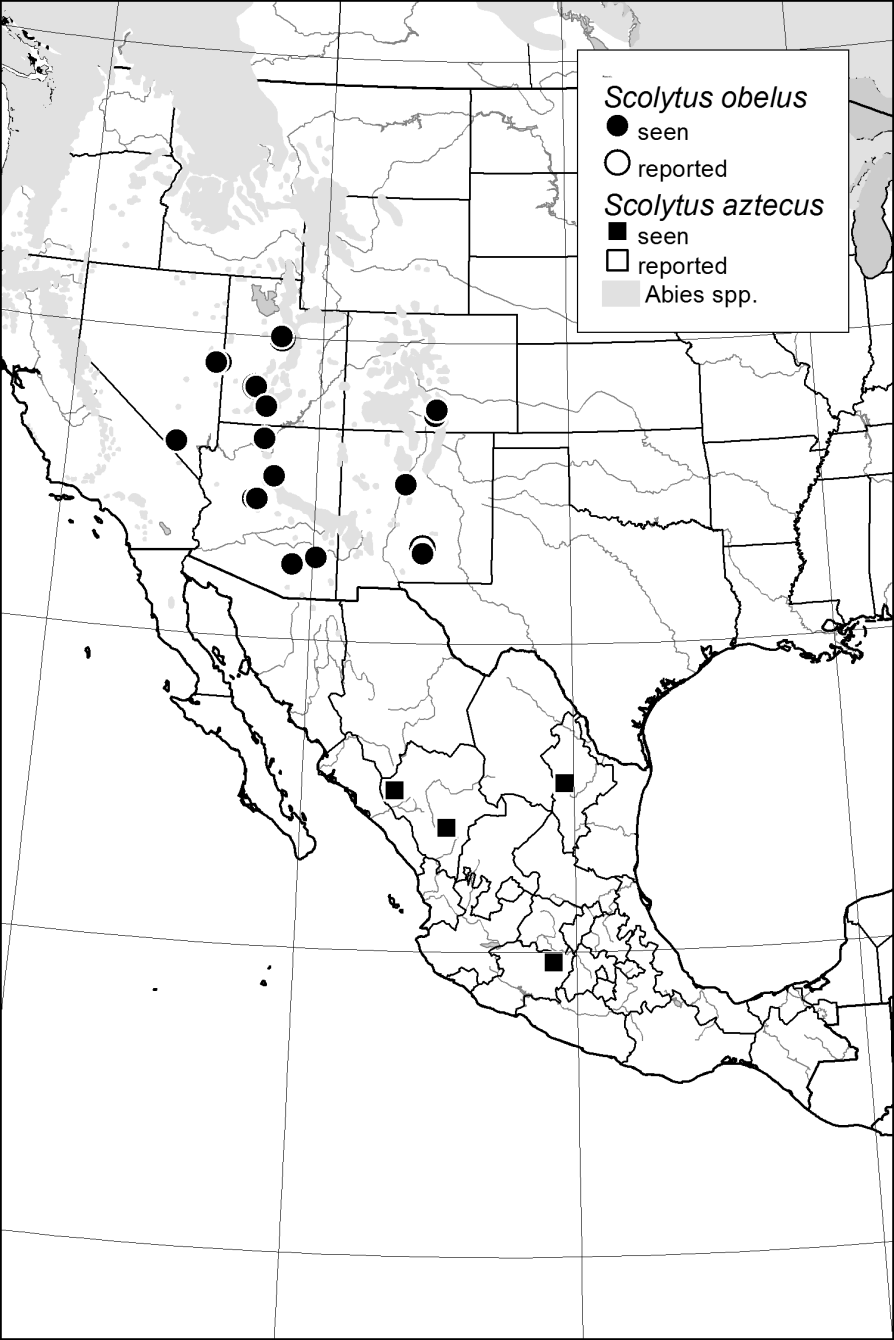
*Scolytus aztecus* and *Scolytus obelus* distribution map.

MEXICO: Durango, Michoacán, Nuevo León (Fig. 27).

##### Hosts.

*Abies religiosa* (Kunth) Schltdl. & Cham. (sacred fir), *Abies durangensis* Martínez (Durango fir) and *Pseudotsuga menziesii* (Mirb.) Franco (Douglas fir). *Abies* species are likely the preferred host.

##### Biology.

*Scolytus aztecus* is an uncommon species and little is known regarding its biology. *Scolytus aztecus* colonizes both standing trees and felled trees (Wood 1967; Cibrián Tovar et al. 1995). The treetops attacked by *Scolytus aztecus* exhibit red foliage. *Scolytus aztecus* has been collected feeding in the cambium of large *Abies* trees, although it has also been collected from *Pseudotsuga*. Because of limited host records, it is unknown whether the primary host is *Abies* or *Pseudotsuga*. Phylogenetic placement of the species suggests *Abies* (Fig. 4) because the species belongs to a clade of *Abies* feeding taxa. The adult gallery is transverse across the grain of the wood with a central nuptial chamber, similar in appearance to that of *Scolytus ventralis* (see Cibrián Tovar et al. 1995, fig 132). Larval mines are in the cambium and do not score the wood. Pupation occurs in the cambium (Wood 1967; Cibrián Tovar et al. 1995).

##### Remarks.

This is the first description of a male for this species.

#### Scolytus dentatus

Taxon classificationAnimaliaColeopteraCurculionidae

Bright, 1964

Figs 28 –29

Scolytus dentatus Bright, 1964: 167.

##### Diagnosis.

The *Scolytus dentatus* male is easily distinguished by the endemic distribution in the Santa Lucia range of California, and by the presence of a median denticle on the apical margin of ventrite 4, occasionally median denticles may also be present on the apical margins of ventrites 2 and 3. The female is distinguished by its distribution and differentiated from the *Scolytus praeceps* female by the presence of a strongly developed and distinct epistomal process and larger size.

##### Description (male).

3.0–4.0 mm long (mean = 3.45 mm; n = 15); 1.7–2.5 times as long as wide. Head, pronotum and abdominal venter dark red-brown, antennae light brown, legs dark red-brown to light brown, elytra light red-brown. Pronotum darker than elytra.

*Head.* Epistoma moderately, acutely emarginated; epistomal process strongly produced, elevated; median area above mandibles bearing dense patch of long, yellow, hair-like setae. Frons appearing flattened when viewed laterally, not impressed; moderately, finely, longitudinally aciculate, coarsely punctate; aciculations converging at epistoma; punctures small, coarse; moderately, uniformly covered by long, fine, yellow erect hair-like setae, these longer than width of midpoint of eye. Antennal scape short, elongate; club flattened, ovoid, setose with partial septum, two arcuate sutures visible.

*Pronotum* wider than long; apical margin broadly rounded, median area between eyes lined with scales; sides distinctly arcuate, strongly constricted near apex, forming a weak transverse impression near apical margin; surface smooth, shining, punctulate, punctures moderately abundant, larger and more abundant laterally and on apical constriction; apical and anterolateral margins bearing sparse, erect, yellow, hair-like setae; base weakly bisinuate.

*Elytra* with sides sub-parallel on basal half, narrowing to subquadrate, smooth apex; apex entire at suture. Margin of apical edge bearing large, coarse punctures. Disc glabrous, smooth, shining; interstriae not impressed, more than twice width of striae, punctures uniseriate, smaller than those of striae; striae weakly impressed. Declivity bearing sparse, short, erect yellow setae. Metepimeron half-length of metanepisternum.

*Venter.* Apical margin of ventrite 1 strongly produced, elevated above base of ventrite 2, ventrite base 2 appearing impressed. Ventrite 2 nearly perpendicular to ventrite 1; surface glabrous, shagreened, dull, finely, obscurely punctate; punctures small, fine and shallow; surface flattened. Apical margins of ventrites 2 and 3 may be armed with median denticle, median denticle always present on apical margin of ventrite 4; lateral margins of ventrites 2–4 unarmed. Ventrite 5 carinate ridge closer to apical margin of segment; length of ventrite 5 equal to combined lengths of ventrites 3 and 4; setal patch and median depression absent.

**Figure 28.**
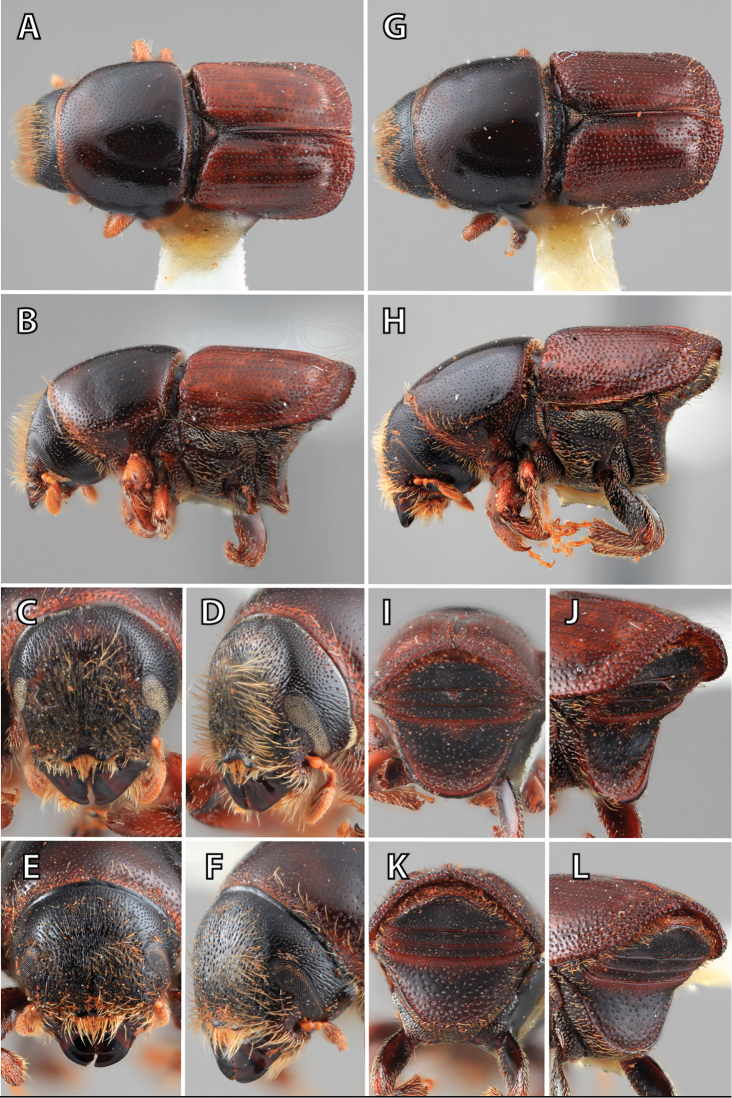
*Scolytus dentatus* **A** dorsal male habitus **B** lateral male habitus **C** male frons **D** male frons oblique **E** male venter **F** male venter oblique **G** dorsal female habitus **H** lateral female habitus **I** female frons **J** female frons oblique **K** female venter **L** female venter oblique.

**Figure 29.**
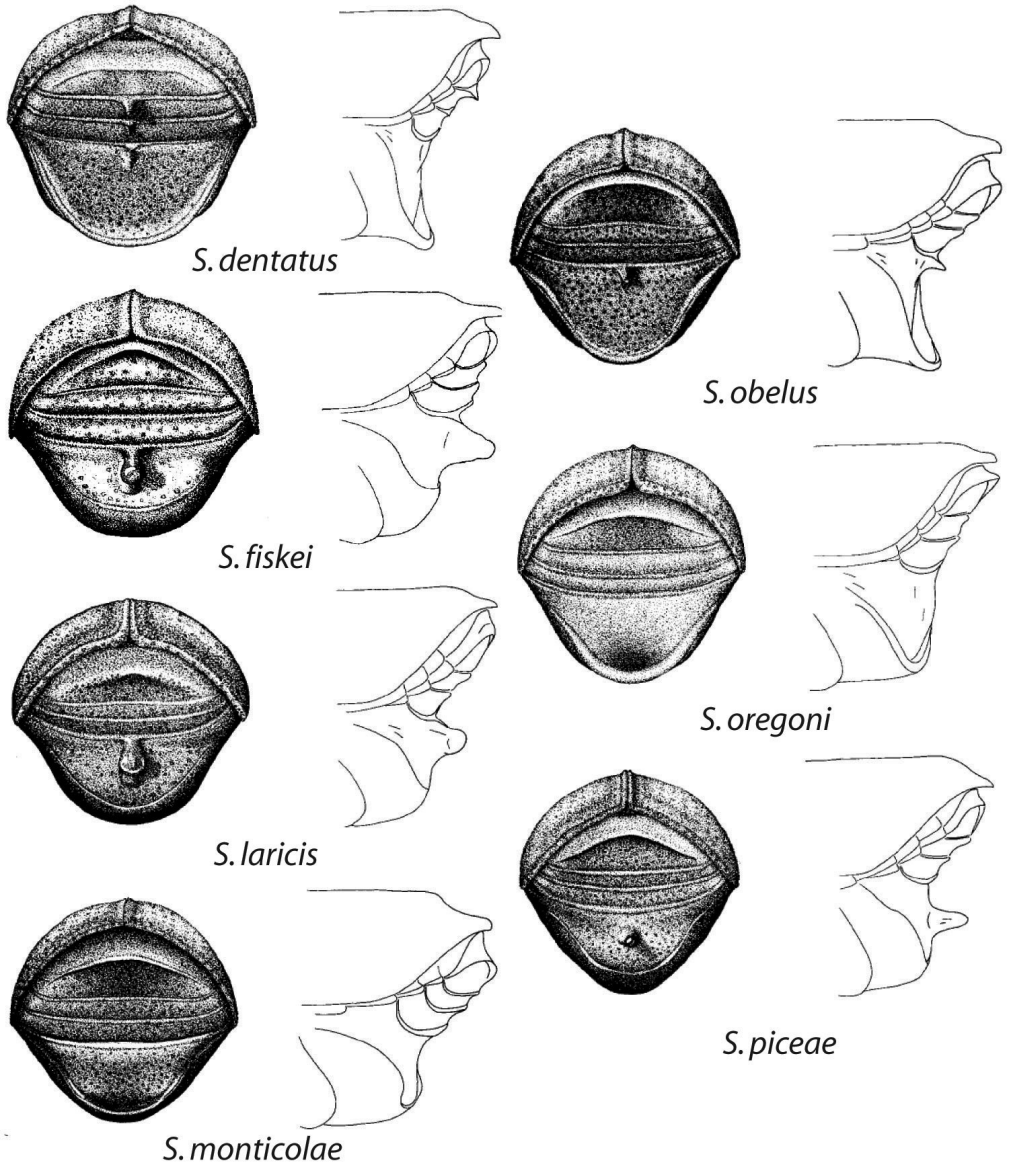
*Scolytus dentatus*, *Scolytus fiskei*, *Scolytus laricis*, *Scolytus monticolae*, *Scolytus obelus*, *Scolytus oregoni* and *Scolytus piceae* venters (from Edson 1967).

##### Female.

3.4–4.6 mm long (mean = 3.7 mm; n = 15); 2.2–2.5 times as long as wide. Similar to male except epistoma less strongly emarginated, epistomal process less strongly produced and elevated, frons convex when viewed laterally, weakly longitudinally aciculate, setae sparser, shorter, less than width of eye; weakly medially impressed between inner apices of eyes. Ventrites unarmed.

##### Specimens examined.

95.

##### Type material.

Holotype *Scolytus dentatus* Bright: male, labeled “Calif: Monterey Co, Cone Peak, 6-29-[19]63, *Abies bracteata*, C.J. Wray Collector” (CASC). Allotype, female, *Scolytus dentatus*, identical data as holotype. Paratypes: **UNITED STATES:** ***California*:** *Monterey Co.*: Cone Peak, 29.VI.[19]63, C.J. Wray, ex. *Abies bracteata* (CASC-6, CNCI-20, EMEC-39, USNM-8). Carmel Valley, 15 mi S., 22.VII.[19]63, C.J. Wray, ex. *Abies bracteata*, (CASC-4).

##### Non-type material.

**UNITED STATES:** ***California*:** *Monterey Co.*: Carmel Valley, 15 mi S., 23.VII.[19]63, D.E. Bright, ex. *Abies bracteata* (CASC-2); 30.VII.1964 (CASC-2). Cone Peak, 29.VI.[19]63, C.J. Wray, ex. *Abies bracteata* (CASC-1, USNM-12). Williams Canyon, Los Padres National Forest, 24.IV.1992, D.E. Bright, G. Ferrell, ex. *Abies bracteata* limbs (CNCI-1).

##### Distribution.

**Figure 30.**
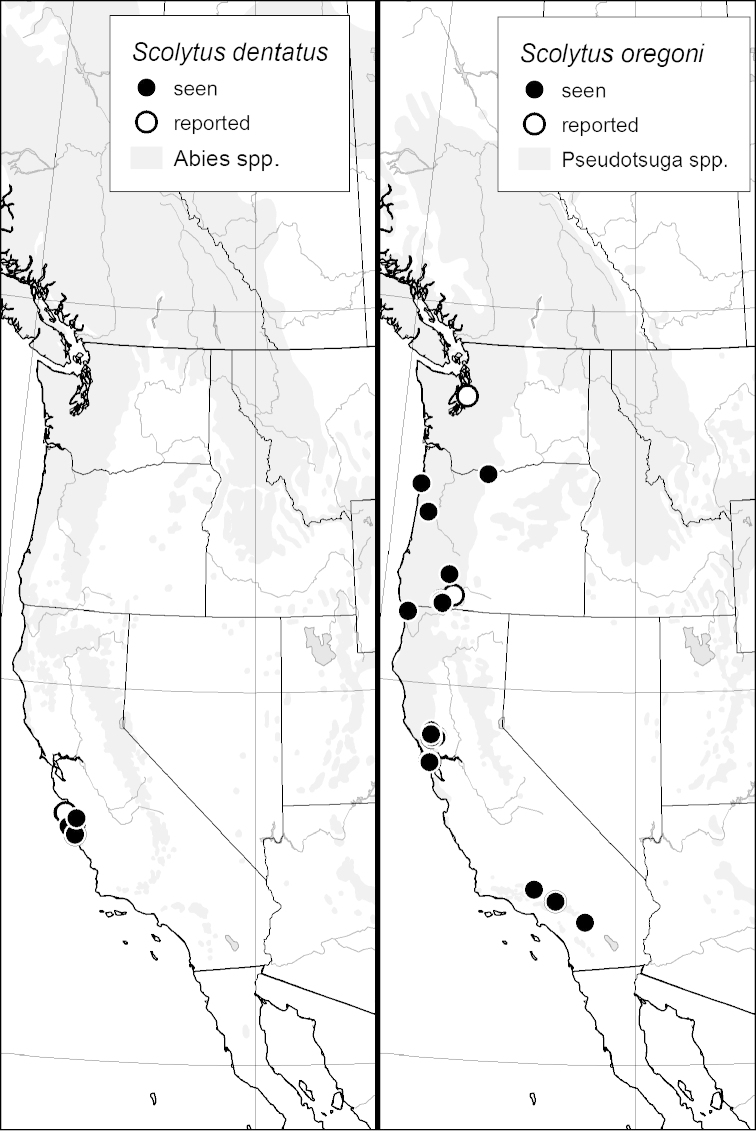
*Scolytus dentatus* and *Scolytus oregoni* distribution map.

UNITED STATES: California (Fig. 30).

##### Hosts.

*Abies bracteata* (D. Don) Poit. (bristlecone fir / Santa Lucia fir).

##### Biology.

Little is known regarding the biology of this uncommon and narrowly geographically restricted species. The host, *Abies bracteata*, is restricted to the Santa Lucia Mountains of California. The tree is distributed in small patches in deep, moist canyon bottoms as well as dry, rocky slopes and ledges within Los Padres National Forest in Monterey County, especially near Cone Peak and Church Creek (Griffin and Critchfield 1972; Sullivan 1993). *Scolytus dentatus* has been collected feeding in the bole of large standing trees (Bright 1964) but is also reported from larger limbs and fresh slash (Edson 1967).

The adult gallery somewhat resembles an expanded ‘E’ (Fig. 24). The gallery is parallel to the grain of the wood and consists of a central nuptial chamber and two egg galleries, one below and one above the nuptial chamber. Each egg gallery ends in a pronounced hook. Galleries strongly score the cambium and lightly score the sapwood. The adult gallery averages 2.5–12.0 cm in length (Bright 1964; Edson 1967). Egg niches are closely spaced on both sides of the maternal gallery (Bright and Stark 1973). Larval mines are first perpendicular to the egg gallery and later turn to form a fan shaped pattern before terminating at pupation chambers in the sapwood (Edson 1967).

##### Remarks.

Considerable variation is observed in the presence of median denticles on the apical margins of male ventrites 2 and 3. Frequently males only have a median denticle on the apical margin of ventrite 4 as those on 2 and 3 may or may not be present in the male. This species is related to *Scolytus robustus* and *Scolytus subscaber* based on morphological characters (Fig. 2). Both of these species also feed on true firs (*Abies* spp.) and have the apical margin of male ventrite 1 strongly apically produced.

#### Scolytus fiskei

Taxon classificationAnimaliaColeopteraCurculionidae

Blackman, 1934 valid sp.

Figs 29 , 31

Scolytus fiskei Blackman, 1934: 25.

##### Diagnosis.

*Scolytus fiskei* is very morphologically similar to *Scolytus laricis* and *Scolytus unispinosus*. Males of are distinguished from those of *Scolytus laricis* by the frons flattened when viewed laterally, never deeply impressed, by the moderately abundant frontal setae (compared to dense) and by the host genus, *Pseudotsuga*. Males are distinguished from those of *Scolytus unispinosus* by the following combination of characters: abdominal venter shiny in luster, the base of the ventrite 2 spine extends from the apical margin to three-quarters the length of the segment and geographical distribution east of the Rocky Mountains. The female is distinguished from that of both species by the shining luster of ventrite 2.

##### Description (male).

2.2–2.8 mm long (mean = 2.4 mm; n = 15); 2.1–2.8 times as long as wide. Head, antennae, pronotum, and abdominal venter dark red-brown, elytra and legs yellow-brown to light brown. Pronotum typically darker than elytra.

*Head.* Epistoma weakly emarginated; epistomal process present, moderately developed, low; median area above mandibles bearing dense patch of long, yellow, hair-like setae. Frons appearing flattened when viewed laterally from epistoma to vertex, slightly transversely impressed just above epistoma to inner apices of eyes; moderately, coarsely, longitudinally aciculate-punctate; aciculations converging at epistoma; punctures large, sparse and coarse; moderately, uniformly covered by long, fine, yellow, erect, hair-like setae, these longer than width of midpoint of eye. Antennal scape short, elongate; club flattened, irregularly ovoid, setose with partial septum, two broadly arcuate sutures visible.

*Pronotum* wider than long; apical margin broadly rounded, median area between eyes lined with scales; sides distinctly arcuate, strongly constricted near apex, forming a weak transverse impression near apical margin; surface smooth, shining, punctures on disc fine, shallow, moderately abundant, larger and more abundant laterally and on apical constriction; apical and anterolateral margins bearing sparse, erect, yellow, hair-like setae; base weakly bisinuate.

*Elytra* with sides sub-parallel on apical half, narrowing to subquadrate, smooth apex; apex moderately emarginated at suture. Margin of apical edge bearing small, fine punctures. Disc smooth, shining; interstriae not impressed, twice width of striae, punctures uniseriate, smaller than those of striae; punctures bearing short, sparse, recumbent, yellow setae slightly longer than size of a puncture (may be abraded); striae weakly impressed. Declivity bearing sparse, short, erect yellow setae. Metepimeron less than half-length of metanepisternum.

*Venter.* Apical margin of ventrite 1 rounded, 2 marked by weak carina. Ventrite 2 nearly perpendicular to ventrite 1; surface smooth, shining, finely punctate; punctures small, fine, shallow; covered with sparse setae less than length of segment 3; surface convex; apical margin armed with laterally compressed, median spine with base extending from apical margin to ¾ length of segment, apex rounded; lateral margins of ventrites 2–3 and ventrite 4 unarmed. Ventrite 5 carinate ridge equidistant between basal and apical margins of segment; length of ventrite 5 less than combined lengths of ventrites 3 and 4; setal patch and median depression absent.

**Figure 31.**
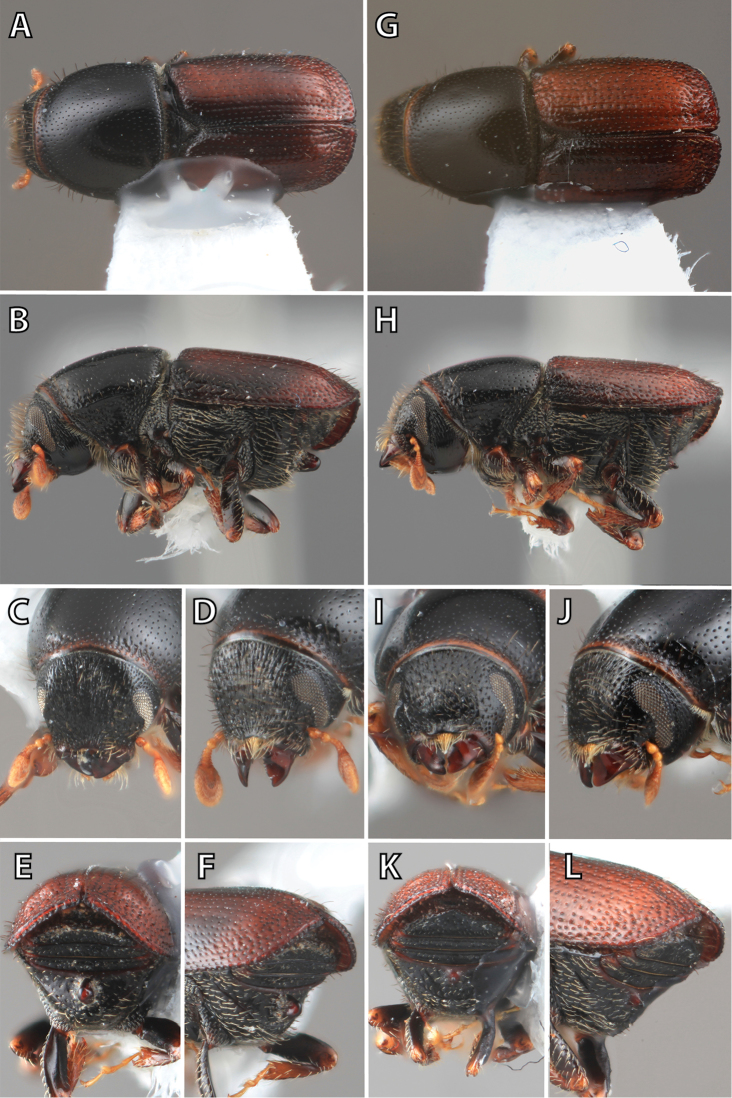
*Scolytus fiskei* **A** dorsal male habitus **B** lateral male habitus **C** male frons **D** male frons oblique **E** male venter **F** male venter oblique **G** dorsal female habitus **H** lateral female habitus **I** female frons **J** female frons oblique **K** female venter **L** female venter oblique.

##### Female.

2.2–3.5 mm long (mean = 4.65 mm; n = 15); 2.1–2.5 times as long as wide. Similar to male except epistoma feebly emarginated, epistomal process weakly developed, frons convex when viewed laterally, weakly longitudinally aciculate, setae sparser, shorter, less than width of eye; weakly transversely impressed between epistoma and inner apices of eyes. Second ventrite apical margin armed with acute median denticle, with base extending from apical margin to half-length of segment.

##### Specimens examined.

297.

##### Type specimens.

Holotype *Scolytus fiskei* Blackman: male, labeled “[Capitan Mountains, N. Mex. 25 April, 1907], Hopk. US 3959, W.F. Fiske Collector, Type No. 43840 USNM” (USNM).

##### Non-type specimens.

**CANADA:** ***BRITISH COLUMBIA*:** Aspen Grove, 12.VII.1931, H. Richmond (CNCI-11, FMNH-2). Clinton, 6 mi N., 7.VII.1972, D.E. Bright, ex. *Pseudotsuga menziesii* (CNCI-7). Creston, 8.VI.1958, H. & A. Howden (EMEC-1). Fort Steele, 14.VI.[19]26, R. Hopping (CASC-1). Indian Meadows, Midday Creek, 13.VII.1920, R. Hopping, ex. *Pseudotsuga taxifolia* [= *Pseudotsuga menziesii*] (CASC-2, CNCI-2). Lumby, Creighton Valley, 3.VI.[19]22, R. Hopping (CASC-1). Merritt, Midday Valley, 27.VI.1926, W. Mathers (CASC-2). Trinity Valley, 24.VI.1928, J.R. Howell, ex. *Pinus monticola* (CASC-1), 10.VII.1928 (CASC-1). **UNITED STATES:** ***ARIZONA*:** *Pima Co.*: Tucson, Mount Lemmon, 11.VI.1969, S.L. Wood, ex. *Pseudotsuga menziesii* (MSUC-2). ***COLORADO*:** *Boulder Co.*: Nederland, 5–7 km N., 5.VIII.2009, D.E. Bright, B.A. Barr, ex. branches of *Pseudotsuga menziesii* (CNCI-4). [*La Plata Co.*]: Durango, Junction Creek Rd, 10000 ft, 12-17.VII.1968, E.C. Becker (CNCI-1). *Larimer Co.*: Roosevelt National Forest, Big Thompson Canyon, N40°24.456', W105°24.565', 7080 ft, 5.V.2010, S.M. Smith, D.E. Bright, B.A. Barr, ex. *Pseudotsuga menziesii* (MSUC-36). ***IDAHO*:** [*Adams Co.*]: Tamarack, 10 mi S., 3.VII.1967 (WFBM-1). *Boise Co.*: Idaho City, 2.VI.1970 (WFBM-9), 1.I.1971 (WFBM-4). Lowman, Edna Creek, 6.VII.[19]72, A. Vaccares, G. Starr, ex. Douglas fir stump [= *Pseudotsuga menziesii*] (WFBM-1). *Bonner Co.*: 6.VI.1986, M.M. Furniss, J.B. Johnson, ex. *Pseudotsuga menziesii* (WFBM-7). Priest Lake, Indian Creek, 6.VI.1986, M.M. Furniss, J.B. Johnson, ex. *Pseudotsuga menziesii* (WFBM-25). Priest Lake, 6.VI.1986, M.M. Furniss, J.B. Johnson, ex. *Pseudotsuga menziesii* (WFBM-1). *Boundary Co.*: Parker Creek, 8.VI.1986, M.M. Furniss, J.B. Johnson, ex. *Pseudotsuga menziesii* (WFBM-5). *Clearwater Co.*: Elk River, 12 mi S.E., Hopk. U.S. 58536, 9.VII–16.VII.1973, R.D. Oakes (WFBM-1), 30.VII-6.VIII.1973 (WFBM-1); Hopk. U.S. 58771, 25.VI-1.VII.1974, J.M. Wells, ex. in flight (WFBM-1). *Custer Co.*: Herd Lake, Hopk. U.S. 60796-A, 3.IX.1978, M.M. Furniss, ex. *Pseudotsuga menziesii* (WFBM-9). MacKay, 9 mi N.E., 19.VII.1985, M.M. Furniss, J.B. Johnson, ex. *Pseudotsuga menziesii* (WFBM-3). Summit, W. of Pass Creek, 19.VII.1985, M.M. Furniss, J.B. Johnson, ex. *Pseudotsuga menziesii* (WFBM-12). *Latah Co.*: Big Sand Creek, Hopk. U.S. 53545, 11.VI.1969, M.M. Furniss (WFBM-1), 20.VI.1969 (WFBM-1). Moscow, 20.XI.1961, R.E. Stecker, ex. reared from Doug fir [= *Pseudotsuga menziesii*] (WFBM-33). Moscow Mountain, Hopk. U.S. 48869, 18.VII.1967, ex. *Pseudotsuga menziesii* (WFBM-3). Viola, 21.VI.1985, S.J. Gast, M.M. Furniss, ex. *Pseudotsuga menziesii* (WFBM-2). *Nez Perce Co.*: Forest, III.1985, M.M. Furniss, ex. *Pseudotsuga menziesii* (WFBM-6). *Shoshone Co.*: Red Ives Ranger Station, V.1983, M.M. Furniss, ex. *Pseudotsuga menziesii* (WFBM-1). *Valley Co.*: Cascade, 7 mi E., 14.VI.1966, R.L. Furniss (WFBM-1). ***MONTANA*:** *Lake Co.*: Swan Lake, 28.VI.1963, M.M. Furniss, ex. *Pseudotsuga menziesii* (WFBM-1). *Madison Co.*: Alder, 18 km S.W., 8.IX.1978, M.M. Furniss, ex. *Pseudotsuga menziesii* (WFBM-6), Alder, 12 mi S.W. (WFBM-16). *Park Co.*: Livingston, 10 mi S.E., 23.VII.1988, M.M. Furniss, J.B. Johnson, ex. *Pseudotsuga menziesii* (WFBM-2). ***NEW MEXICO*:** *Otero Co.*: Cloudcroft, 4.VI.1969, tree 53, S.L. Wood, ex. *Pseudotsuga menziesii* (MSUC-2). *Sierra Co.*: Emory Pass, 24.VII.1974, D.E. Bright, ex. *Pseudotsuga menziesii* (CNCI-1). ***WASHINGTON*:** [*Whitman Co.*]: Pullman, 23.VI.1951, N.M. Downie (FMNH-1). ***WYOMING*:** [*Teton Co.*]: Jackson, A.D. Hopkins, ex. *Pseudotsuga menziesii* (USNM-1).

##### Distribution.

**Figure 32.**
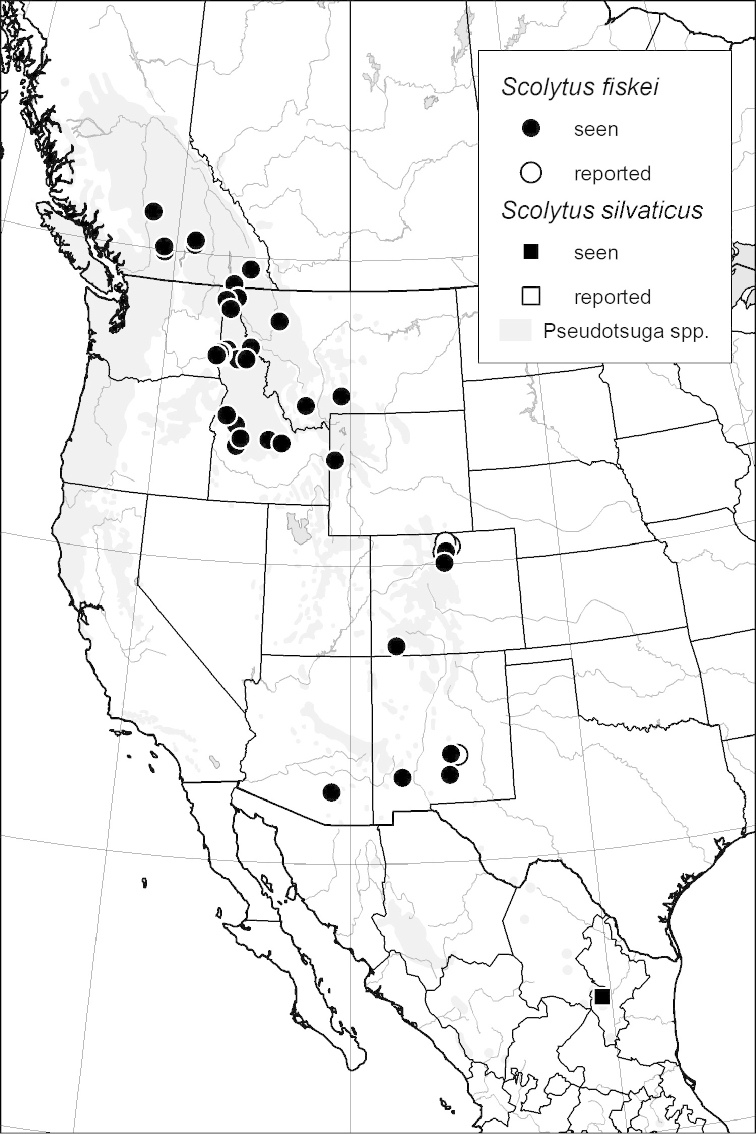
*Scolytus fiskei* and *Scolytus silvaticus* distribution map.

CANADA: British Columbia. UNITED STATES: Arizona, Colorado, Idaho, Montana, New Mexico, Washington, Wyoming (Fig. 32).

##### Hosts.

*Pseudotsuga menziesii* (Mirb.) Franco (Douglas fir).

##### Biology.

*Scolytus fiskei* colonizes suppressed limbs and branches of *Pseudotsuga menziesii* as well as fresh slash (Edson 1967; Smith, pers. obs.). The adult gallery is bayonet shaped, parallel to the grain of wood and consists of two egg galleries, one below and one above the nuptial chamber and a turning niche. The female constructs the adult gallery mostly in the cambium and lightly scores the sapwood. Larval mines are perpendicular to the egg gallery and turn to form a fan shaped pattern (Edson 1967; Furniss and Johnson 2002; Smith, pers. obs.) (Fig. 24). The adult gallery measures 3.8–9.0 cm in length (Edson 1967). The following year adults emerge and excavate tunnels within twigs of Douglas fir for maturation feeding causing twig flagging (reported as *Scolytus unispinosus* McMullen and Atkins 1962).

Like most *Scolytus* species, there is a very limited amount of information regarding *Scolytus fiskei*. For many years it was considered a synonym of *Scolytus unispinosus* and was thus referred to as *Scolytus unispinosus* in publications. Very little was written about this species in the US (see Wood and Bright 1992) other than host preference and gallery descriptions (Keen 1938; Edson 1967; Bright 1976). McMullen and Atkins (1962) reported some notes on the biology of *Scolytus unispinosus* in British Columbia and appear to have reported a combined account of *Scolytus unispinosus* and *Scolytus fiskei*. The authors noted the gallery of the *Scolytus unispinosus* studied in their investigations as “in about 15 per cent of the galleries over 20 days of age were the type described by Chamberlin and Keen [*Scolytus unispinosus*, which has only a single egg gallery]; the majority were of the forked type and were similar to those of *Scolytus tsugae* [referencing a forked or bayonet shaped gallery that is made by *Scolytus fiskei*]”. Their description matches the gallery description of *Scolytus fiskei*, which like has two egg galleries as part of a bayonet shaped adult gallery. Because most of the specimens are of *Scolytus fiskei*, this article does offer some information regarding the biology of the species be used with caution because not all of the findings may apply to *Scolytus fiskei*.

##### Remarks.

The holotype of *Scolytus fiskei* does not bear a locality label. Blackman’s (1934) description states the holotype was collected at the Capitan Mountains, New Mexico.

Furniss and Johnson (2002) report *Scolytus unispinosus* from Alberta, Canada. These specimens are likely *Scolytus fiskei* based on the geographic distribution of the species in the Rocky Mountains.

Wood (1977: 388) placed *Scolytus fiskei* in synonymy with *Scolytus unispinosus* after examining both holotypes and 164 specimens from Arizona to British Columbia and concluded that there was too much intraspecific variation to recognize them as separate species. We assessed the intraspecific and interspecific variation (Tables 6 and 7) for each of the four genes for each species. *Scolytus fiskei* intraspecific variation for COI was low, 0.0–0.0231, and averaged 0.0183 among all sampled populations. *Scolytus unispinosus* intraspecific variation was also low, 0.0016–0.0282 and averaged 0.0121. Interspecific variation between *Scolytus fiskei* and *Scolytus unispinosus* was much higher, 0.0331–0.0521 and averaged 0.043 among populations. Similar differences were also observed with CAD (Table 7). The species have separate geographical ranges with *Scolytus fiskei* occurring primarily in the Rocky Mountains and British Columbia while *Scolytus unispinosus* occurs in the Cascade and Sierra mountains from California to British Columbia. Both species are sympatric in the Interior Plateau of British Columbia near Merritt. The species are separated by the characters listed in the diagnosis and by the galleries. The galleries of *Scolytus fiskei* contain two egg galleries; one above and one below the nuptial chamber. The gallery of *Scolytus unispinosus* only contains a single egg gallery. In addition, the gallery of *Scolytus fiskei* lightly scores the sapwood while that of *Scolytus unispinosus* deeply scores the sapwood. After examining the types, 950 specimens of both species and testing the monophyly of each species using four genes, it is apparent that *Scolytus fiskei* is a distinct lineage and is here removed from synonymy with *Scolytus unispinosus*.

#### Scolytus hermosus

Taxon classificationAnimaliaColeopteraCurculionidae

Wood, 1968

Fig. 33

Scolytus hermosus Wood, 1968: 12.

##### Diagnosis.

Both sexes resemble the *Scolytus silvaticus* female. The female is distinguished by having a weakly developed epistomal process and the male is distinguished by having the apical margin of ventrite 1 produced, forming a carinate lip along the basal margin of ventrite 2 that is twice as produced as thick and by the host genus, *Abies*.

##### Description (male).

3.0 mm long (n = 1); 1.5 times as long as wide. Head, antennae, pronotum, and abdominal venter dark red-brown, elytra and legs yellow-brown to light brown. Pronotum typically darker than elytra.

*Head.* Epistoma weakly emarginated; epistomal process weakly developed, low; median area above mandibles bearing dense patch of long, yellow, hair-like setae. Frons appearing convex when viewed laterally, slightly transversely impressed just above epistoma and between inner apices of eyes; moderately, finely, longitudinally aciculate-punctate; aciculations converging at epistoma; punctures small, coarse; moderately, uniformly covered by long, fine, yellow, erect hair-like setae, these longer than width of midpoint of eye. Antennal scape short, elongate; club flattened, elongate, almost subquadrate, setose with partial septum, two arcuate sutures visible.

*Pronotum* wider than long; apical margin broadly rounded, median area between eyes lined with scales; sides distinctly arcuate, strongly constricted near apex, forming a weak transverse impression near apical margin; surface smooth, shining, punctures on disc fine, shallow, moderately abundant, larger and more abundant laterally and on apical constriction; apical and anterolateral margins bearing sparse, erect, yellow, hair-like setae; base weakly bisinuate.

*Elytra* with sides sub-parallel on apical half, narrowing to subquadrate, smooth apex; apex moderately emarginated at suture. Margin of apical edge bearing large, coarse punctures. Disc smooth, shining; interstriae not impressed, more than twice width of striae, punctures uniseriate, smaller than those of striae, punctures bearing sparse, long, erect, yellow hair-like setae (may be abraded); striae weakly impressed. Declivity bearing sparse, short, erect yellow setae. Metepimeron half-length of metanepisternum.

*Venter.* Apical margin of ventrite 1 produced, forming carinate lip along basal margin of ventrite 2 that is twice as produced as thick, basal margin of ventrite 2 appearing impressed. Ventrite 2 nearly perpendicular to ventrite 1; surface shagreened, dull, finely punctate; punctures small, fine, shallow; setae moderately abundant, recumbent, short, about four times length of a puncture; surface weakly concave; apical margin armed with broad median denticle, occasionally absent; lateral margins of ventrites 2–3 and ventrite 4 unarmed. Ventrite 5 carinate ridge closer to apical margin of segment; length of ventrite 5 greater than combined lengths of ventrites 3 and 4; setal patch and median depression absent.

**Figure 33.**
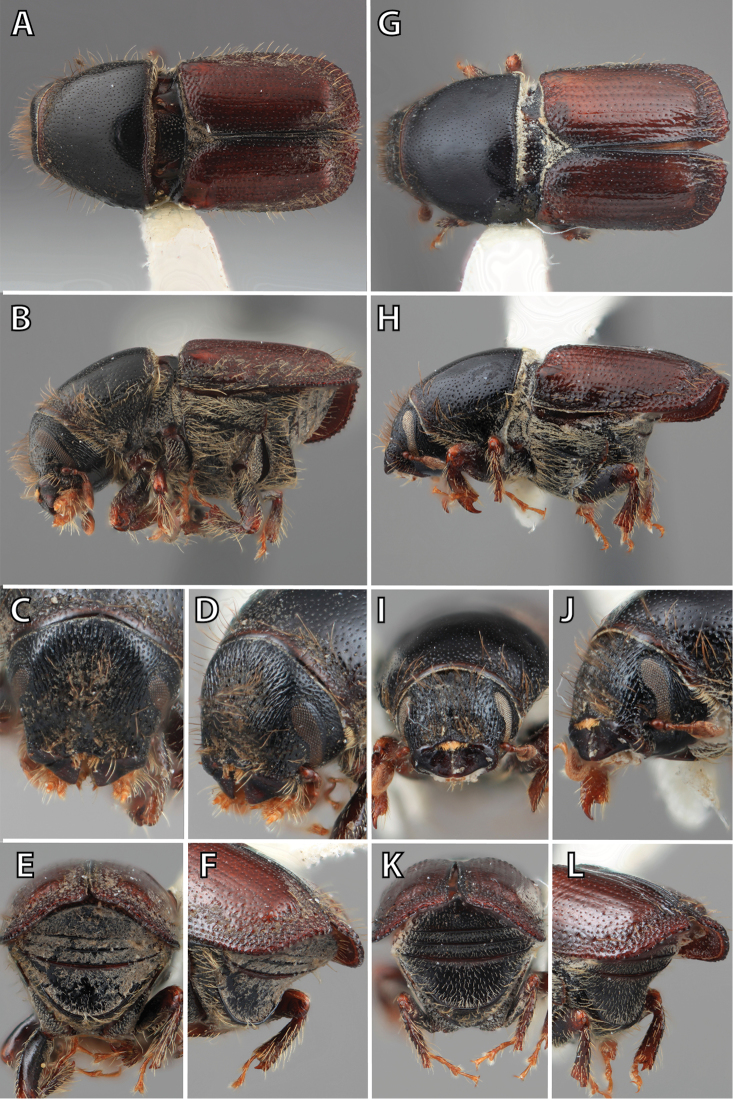
*Scolytus hermosus* **A** dorsal male habitus **B** lateral male habitus **C** male frons **D** male frons oblique **E** male venter **F** male venter oblique **G** dorsal female habitus **H** lateral female habitus **I** female frons **J** female frons oblique **K** female venter **L** female venter oblique.

##### Female.

2.5–3.5 mm long (mean = 3.27 mm; n = 6); 2.1–2.3 times as long as wide. Similar to male except epistoma feebly emarginated, epistomal process absent, frons more strongly convex when viewed laterally, weakly longitudinally aciculate, setae sparser, shorter, less than width of eye; weakly transversely impressed just above epistoma. Apical margin of ventrite 1 weakly elevated above base of ventrite 2. Second ventrite unarmed.

##### Specimens examined.

27.

##### Type material.

Holotype *Scolytus hermosus* Wood: male, labeled “Tlaxco 11 mi N., 8900 ft, 9.VII.1967, S.L. W[ood], ex. *Abies religiosa*” (USNM). Paratypes: **MEXICO:** [***Puebla***]: Tlaxco, 11 mi N., 8900 ft, 9.VII.1967, S.L. W[ood], ex. *Abies religiosa* (USNM-21).

##### Non-type material.

**MEXICO:** ***CHIHUAHUA*:** La Magdalena, Hopk. U.S. 62081-B, 27.IV.1981, M.M. Furniss, ex. *Abies durangensis* (USNM-1). ***Nuevo León*:** Cerro Potosi, Hopk. U.S. 58615-C, 21.III.[19]74, M.M. Furniss, ex. *Abies* sp. (USNM-2, WFBM-1).

##### Distribution.

**Figure 34.**
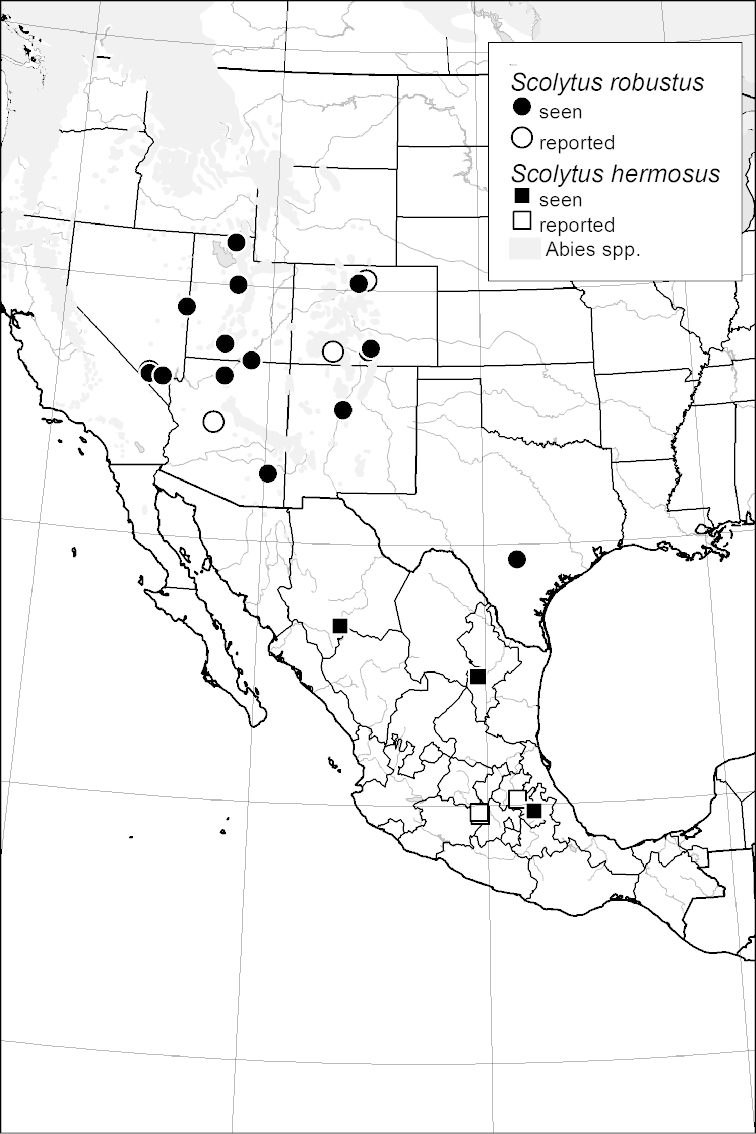
*Scolytus hermosus* and *Scolytus robustus* distribution map.

MEXICO: Chihuahua, Nuevo León, Puebla (Fig. 34).

##### Hosts.

*Abies religiosa* (Kunth) Schltdl. & Cham (sacred fir), *Abies durangensis* Martínez (Durango fir) and *Pseudotsuga menziesii* (Mirb.) Franco (Douglas fir). *Abies* species are likely the preferred hosts.

##### Biology.

There is a paucity of information regarding the biology of this uncommon species. *Scolytus hermosus* has been collected feeding in the cambium of large *Abies* slash greater than 10.0 cm in diameter (Wood 1967) and weakened and dying large *Pseudotsuga menziesii* (Cibrián Tovar et al. 1995). The adult galleries are transverse and perpendicular to the grain of the wood (Wood 1967). This species has been reported to colonize the same material as *Scolytus mundus* (Wood 1968).

#### Scolytus laricis

Taxon classificationAnimaliaColeopteraCurculionidae

Blackman, 1934

Figs 29 , 35

Scolytus laricis Blackman, 1934: 24.

##### Diagnosis.

*Scolytus laricis* is very morphologically similar to *Scolytus fiskei* and *Scolytus unispinosus*. Males are distinguished from those of *Scolytus fiskei* by the dull appearance of abdominal ventrite 2 and by the frons densely covered by long setae. Males can be distinguished from those of *Scolytus unispinosus* by the following combination of characters: the base of the ventrite 2 spine extends from the apical margin to three-quarters the length of the segment and host genus. The female is distinguished from that of *Scolytus fiskei* by the dull luster of ventrite 2 and is distinguished from that of *Scolytus unispinosus* by the frons moderately and coarsely aciculate-punctate; ventrite 1 rounded over onto surface of ventrite 2, not forming an obtuse angle, and the base of ventrite 2 finely impressed.

##### Description (male).

2.3–4.0 mm long (mean = 3.1 mm; n = 15); 1.8–2.5 times as long as wide. Head, pronotum, and abdominal venter dark red-brown to black, elytra and legs light brown, antennae yellow-brown. Pronotum typically darker than elytra.

*Head.* Epistoma weakly emarginated; epistomal process present, moderately developed and elevated; median area above mandibles bearing dense patch of long yellow hair-like setae. Frons appearing flattened when viewed laterally from epistoma to vertex, moderately transversely impressed just above epistoma to vertex; strongly, coarsely, longitudinally aciculate-punctate; aciculations converging at epistoma; punctures sparse, large, coarse; moderately to densely uniformly covered by long, fine, yellow, erect, hair-like setae, these longer than width of midpoint of eye. Antennal scape short, elongate; club flattened, irregularly ovoid, setose with partial septum, two arcuate sutures visible.

*Pronotum* wider than long; apical margin broadly rounded, median area between eyes lined with scales; sides distinctly arcuate, strongly constricted near apex, forming a weak transverse impression near apical margin; surface smooth, shining, punctures on disc fine, shallow, moderately abundant, larger and more abundant laterally and on apical constriction; apical and anterolateral margins bearing sparse, erect, yellow, hair-like setae; base weakly bisinuate.

*Elytra* with sides sub-parallel on apical half, narrowing to subquadrate, smooth apex; apex moderately emarginated at suture. Margin of apical edge bearing large, coarse punctures. Disc smooth, shining; interstriae not impressed, twice width of striae, punctures uniseriate, smaller than those of striae, bearing minute, recumbent setae length of interstrial punctures (may be abraded); striae weakly impressed. Declivity bearing sparse, short, erect, yellow setae. Metepimeron less than half-length of metanepisternum.

*Venter.* Apical margin of ventrite 1 rounded, marked by weak carina on ascendant part of venter. Ventrite 2 nearly perpendicular to ventrite 1; surface smooth, shining, finely punctate; punctures small, fine, shallow; covered with sparse setae less than length of segment 3; surface convex; apical margin armed with laterally compressed, median spine with base extending from apical margin to ¾ length of segment, apex rounded, rarely a slightly elevated vertical carina (Oregon: Dixie Pass and Frog Lake) instead of spine; lateral margins of ventrites 2–3 and ventrite 4 unarmed. Ventrite 5 carinate ridge equidistant between basal and apical margins of segment; length of ventrite 5 less than combined lengths of ventrites 3 and 4; setal patch and median depression absent.

**Figure 35.**
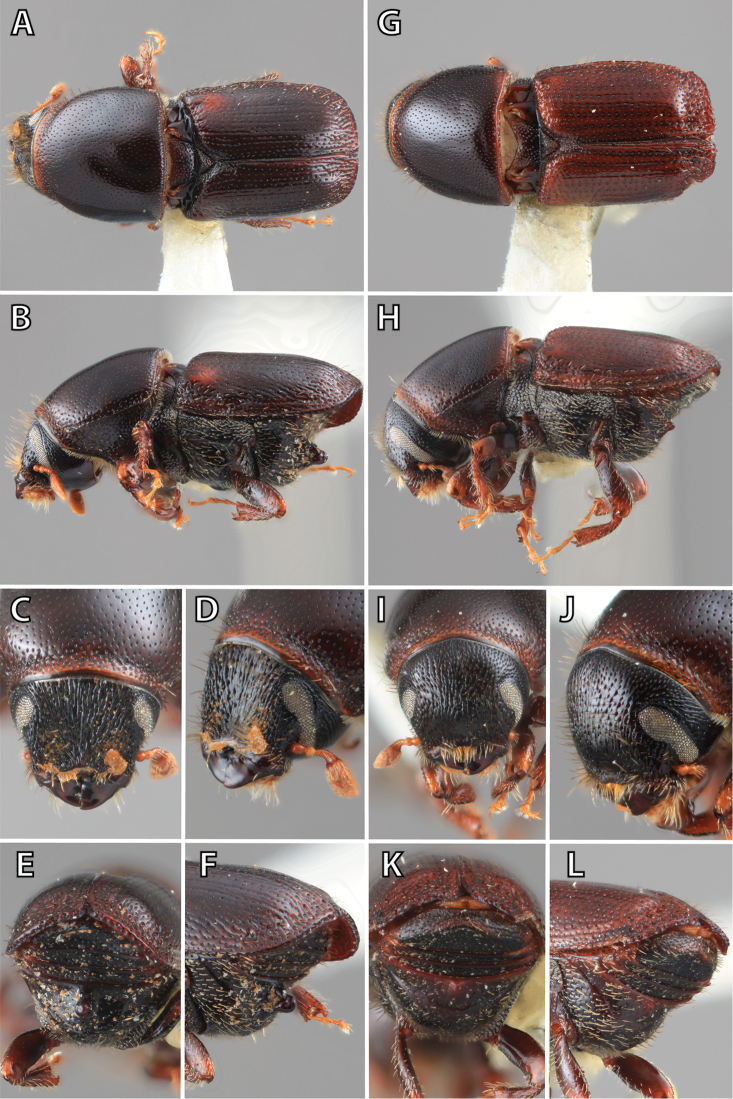
*Scolytus laricis* **A** dorsal male habitus **B** lateral male habitus **C** male frons **D** male frons oblique **E** male venter **F** male venter oblique **G** dorsal female habitus **H** lateral female habitus **I** female frons **J** female frons oblique **K** female venter **L** female venter oblique.

##### Female.

2.6–4.0 mm long (mean = 3.2 mm; n = 15); 2.1–2.9 times as long as wide. Similar to male except epistoma feebly emarginated, epistomal process weakly developed, frons convex when viewed laterally, weakly longitudinally aciculate, setae sparser, shorter, less than width of eye; weakly transversely impressed just above epistoma. Second ventrite apical margin armed with a blunted median denticle, with base extending from apical margin to a third length of segment. Ventrite 5 length greater than combined lengths of ventrites 3 and 4.

##### Specimens examined.

294.

##### Type material.

Holotype *Scolytus laricis* Blackman: male, labeled “[Cedar Mountain, Moscow, ID] Hopk. US 225d, 8636 m [sic!], 6-20-[18]99, Type No. 43839” (USNM).

##### Non-type material.

**CANADA:** ***BRITISH COLUMBIA*:** Canoe, 12.VII.1933, A. Thrupp, ex. *Larix occidentalis* (CASC-3). Rossland, 21.IX.[19]61, D.E. Bright, ex. *Larix laricina* (CNCI-2). **UNITED STATES:** ***IDAHO*:** *Boundary Co.*: [Idaho Panhandle National Forest], Robinson Creek campground, N48°58.197', W116°13.068', 2748 ft, 10.VIII.2010, S.M. Smith, [A.R. Gillogly], ex. *Larix occidentalis*, emerged II.2011, M.M. Furniss (MSUC-94). *Clearwater Co.*: Elk River, V.1981, M.M. Furniss, ex. *Larix occidentalis* (WFBM-17), VII.1981 (WFBM-11). Elk River, 13 mi S.W., Hopk. U.S. 60361-A, 23.IV.1975, M.M. Furniss, ex. *Larix occidentalis* (WFBM-4). *Idaho Co.*: Salmon Mountain, 18.VIII.1985, M.M. Furniss, J.B. Johnson, ex. *Larix lyalli* (WFBM-29). *Kootenai Co.*: Coeur d’Alene, Hopk. U.S. 16306-B, 2.VIII.1919, J.C. Evenden, ex. *Larix occidentalis* (WFBM-9), 8.VIII.1919 (MSUC-11). Deception Creek Experimental Forest, Hopk. U.S. 58889-A, 10.VII.1968, M.M. Furniss (OSAC-4), Hopk. U.S. 53376, 11.VII.1968 (WFBM-2), Hopk. U.S. 60320, 25.VII.1968, (OSAC-2), Hopk. U.S. 50421-B,C, 1.VII.1975 (OSAC-2), Hopk. U.S. 60356, 30.VII.[19]68, M.M. Furniss, ex. *Larix occidentalis* (WFBM-6). *Latah Co.*: Moscow, 2.VIII.1930, 2560 ft, P. Rice, ex. trap (WFBM-1). Moscow Mountain, Hopk. U.S. 53632-F, 23.VI.1964, M.M. Furniss, ex. in flight (WFBM-1), Hopk. U.S. 60421-A, 1.VII.1975 (OSAC-1). ***MONTANA*:** *Missoula Co*: Missoula, 35 mi N.W., 3.XI.[19]65, ex. *Larix occidentalis* (WFBM-2). Nine Mile Creek, Hopk. U.S. 48830, 3.XI.1965, M.M. Furniss, ex. *Larix occidentalis* (WFBM-2, OSAC-1). *Ravalli Co.*: [Bitterroot Mountains], Trapper Peak, 2.X.[19]88, M.M. Furniss, ex. *Larix lyalli* (WFBM-3). [*Unspecified County*]: Libby, 60 mi S.E., 30.VI.[19]72, D.E. Bright, ex. *Larix laricina* (CNCI-7). ***OREGON*:** *Crook Co.*: Summit Prairie, 12.VI.1940, Schuh, Scott, ex. *Larix occidentalis* (CNCI-3, EMEC-2). [*Grant Co.*]: Dixie Pass, Malheur National Forest, 23.VI.1961, S.L. Wood, J.B. Karren, D.E. Bright, ex. *Larix occidentalis* (CNCI-6, USNM-6). *Jefferson Co.*: Camp Sherman, T.O. Thatcher, ex. *Larix lyalli* limbs (CSUC-10), 9.VII.[19]66, [L. Edson], ex. *Larix lyalli* (CNCI-2). Suttle Lake, 4 mi N., 25.VII.1939, ex. *Larix lyalli* (CSUC-1); 28.VII.1939, F. Grey, J. Schuh, ex. *Larix occidentalis* (CASC-1, FMNH-6, MSUC-1, OSAC-14), W.J. Chamberlin, ex. *Larix lyalli* (EMEC-7, WFBM-3). Suttle Lake, 4 mi W., 15.VIII.1939, Schuh, Scott (MSUC-1). [*Marion Co.*]: Clear Lake, 17.VIII.[19]51, R. Kangur, ex. larch [= *Larix* sp.] (EMEC-4, WFBM-2). [*Umatilla Co.*]: Tollgate, 28.X.1948, C. Chastain, ex. *Larix occidentalis* (EMEC-1). [*Wasco Co.*]: [Mount Hood National Forest] Frog Lake [campground], 4S9 E17, 2.VIII.1951, R. Kangur (CNCI-3). ***WASHINGTON*:** *Okanogan Co.*: Disautel, 4.XI.1936, R.L. Furniss, ex. *Larix occidentalis* (OSAC-3). [*Pend Oreille Co.*]: Metaline Falls, Hopk. U.S. 19905, 20.VII.1930, D. DeLeon, ex. *Larix occidentalis* (OSAC-2, WFBM-1). [*Stevens Co.*]: Northport, 18.VII.1929, R. Hopping (OSAC-1). [*Unspecified County*]: Mount Adams, 7000 ft, Hopk. U.S. 53359-C, 2.VIII.1968, M.M. Furniss, ex. on snowfield (WFBM-1).

##### Distribution.

**Figure 36.**
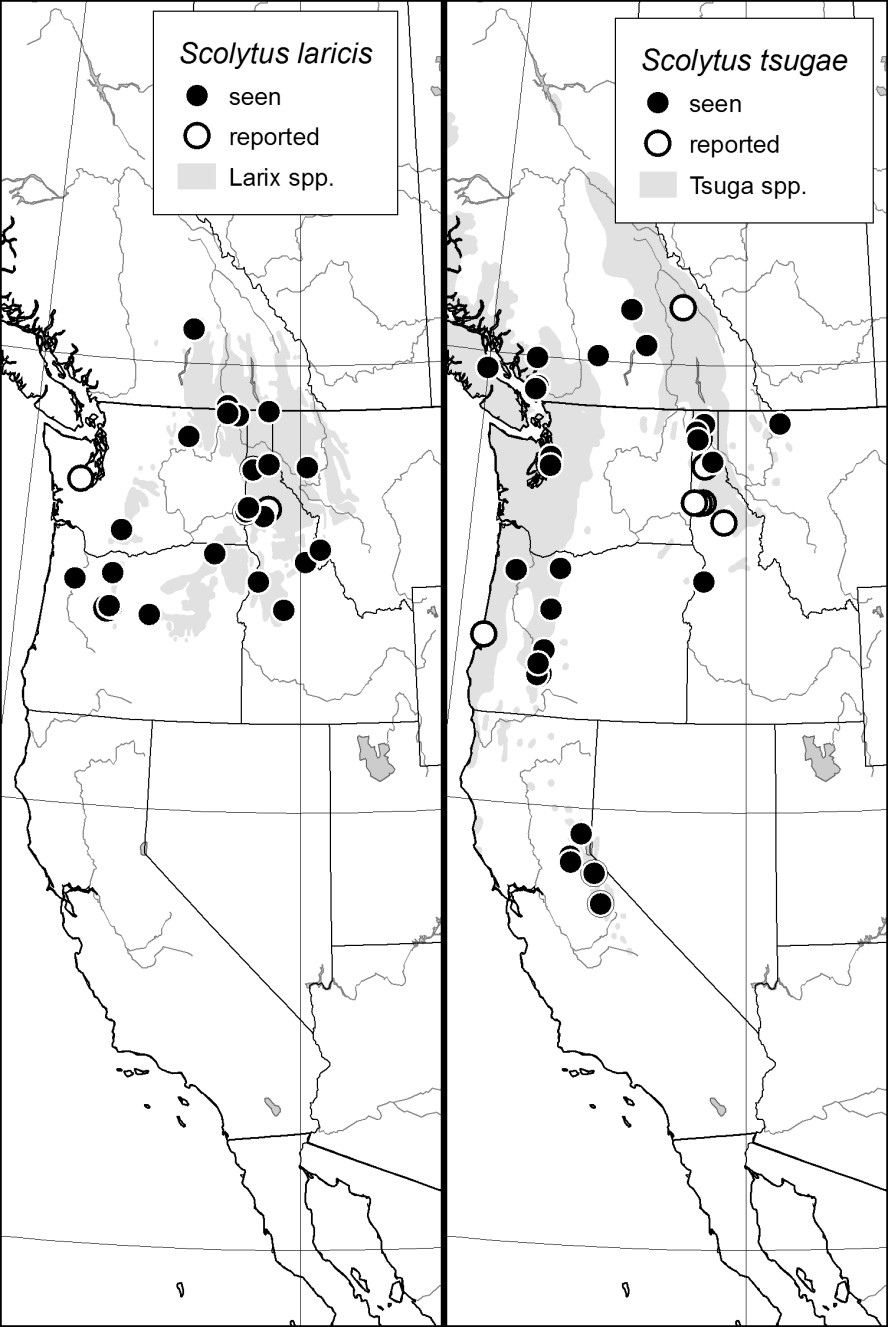
*Scolytus laricis* and *Scolytus tsugae* distribution map.

CANADA: British Columbia. UNITED STATES: Idaho, Montana, Oregon, Washington (Fig. 36).

##### Hosts.

*Larix occidentalis* Nutt. (western larch) and *Larix lyalli* Parl. (subalpine larch).

##### Biology.

*Scolytus laricis* prefers to colonize suppressed lower limbs of its host and fresh slash.

The adult gallery is parallel to the grain of the wood and consists of two egg galleries and a central nuptial chamber (Fig. 24). The central nuptial chamber extends at a right angle to the rest of the gallery and opposite the nuptial chamber is a rounded turning niche. This gives the central part of the gallery an ovoid appearance that is different from all other Nearctic *Scolytus*. Egg galleries are extended parallel to the grain of the wood. The adult gallery scores the sapwood more deeply than the cambium, and ranges in length from 6.4–11.5 cm. The female constructs widely spaced egg niches on both sides of each egg gallery. Larval mines extend against the grain of wood and gradually diverge before terminating at pupation chambers that deeply score the sapwood (Edson 1967; Furniss and Johnson 2002). There is one generation per year and broods overwinter as larvae (Furniss and Johnson 2002).

##### Collection notes.

*Scolytus laricis* was collected four times by the senior author and each time specimens were collected from fresh broken branches that were less than 5.0 cm in diameter with bright green needles. Specimens were also collected from a fallen *Larix occidentalis* of 15.0 cm DBH.

##### Remarks.

Furniss and Johnson (2002) reported that specimens collected from *Larix lyalli* create a different gallery structure than those collected from *Larix occidentalis* (M.M. Furniss pers. comm.). The galleries on *Larix lyalli* are “shorter, less uniform, and with the entrance chamber often extended a short distance to the sides of the gallery” (Furniss and Johnson 2002). Specimens from *Larix lyalli* could not be collected during the coarse of our investigation. We noticed slight morphological variation among some populations but were unable to determine if the differences represent species limits. Further study is needed to address this question.

The holotype of *Scolytus laricis* does not bear a locality label. Blackman’s (1934) description states A.D. Hopkins collected the holotype on Cedar Mountain near Moscow, Idaho. Numerous collectors listed in the material examined reported this species as collected from *Larix lyalli* in Oregon. This host species occurs east of the Cascade Range in the Wenatchee Mountains in Washington and is not known to occur in Oregon (Burns and Honkala 1990). It is probable that the host species for these specimens was *Larix occidentalis*.

#### Scolytus monticolae

Taxon classificationAnimaliaColeopteraCurculionidae

(Swaine, 1917)

Figs 29 , 37

Eccoptogaster monticolae Swaine, 1917: 32.
Scolytus monticolae (Swaine, 1917): Keen 1929: 13.

##### Diagnosis.

*Scolytus monticolae* males are easily confused with *Scolytus reflexus* males, especially those exhibiting the wickhami phenotype. They are easily distinguished by the size of male ventrite 5. In *Scolytus monticolae*, ventrite 5 is equal in length to width of ventrites 3 and 4 combined. In *Scolytus reflexus*, ventrite 5 is equal in length to ventrite 4. *Scolytus monticolae* lacks an epistomal process while *Scolytus reflexus* typically has a strongly developed epistomal process. Males are distinguished from those of *Scolytus tsugae* by the following combination of characters: surface of ventrite 2 shining but minutely reticulate; elytral striae not impressed; basal margin of ventrite 2 more pronounced and produced laterally; elytral strial punctures small, spaced 2–3 diameters of a puncture. Females of *Scolytus monticolae* are distinguished from *Scolytus reflexus* females by having the apical margin of ventrite 1 weakly produced, never rounded and the surface of ventrite 2 smooth and flat, and are separated from those of *Scolytus tsugae* by elytral discal striae not impressed and ventrite 2 shining in luster.

##### Description (male).

2.5–3.0 mm long (mean = 2.9 mm; n = 10); 2.0–2.5 times as long as wide. Head, pronotum, and abdominal venter dark red-brown, legs light brown, antennae yellow-brown, elytra usually dark red-brown but may be brown. Pronotum typically darker than elytra.

*Head.* Epistoma weakly emarginated; epistomal process weakly developed; median area above mandibles bearing dense patch of long yellow hair-like setae. Frons appearing flattened when viewed laterally, slightly transversely impressed just above epistoma; weakly aciculate-punctate, medial area appearing shagreened; aciculations converging at epistoma; punctures small, coarse; sparsely, uniformly covered by long, fine, yellow, erect, hair-like setae, these longer than width of midpoint of eye. Antennal scape short, elongate; club flattened, irregularly ovoid, setose with partial septum, two arcuate sutures visible.

*Pronotum* wider than long; apical margin broadly rounded, median area between eyes lined with scales; sides distinctly arcuate, strongly constricted near apex, forming a weak transverse impression near apical margin; surface smooth, shining, punctures on disc fine, shallow, moderately abundant, larger and more abundant laterally and on apical constriction; apical and anterolateral margins bearing sparse, erect, yellow, hair-like setae; base weakly bisinuate.

*Elytra* with sides sub-parallel on basal half, narrowing to subquadrate, smooth apex; apex moderately emarginated at suture. Margin of apical edge bearing large, coarse punctures. Disc glabrous, smooth, shining; interstriae not impressed, twice width of striae, punctures uniseriate, smaller than those of striae, bearing minute, recumbent setae length of interstrial punctures (may be abraded); striae not impressed. Declivity bearing sparse, short, erect yellow setae. Metepimeron less than half-length of metanepisternum.

*Venter.* Apical margin of ventrite 1 weakly elevated above base of ventrite 2, more pronounced and produced laterally forming two cups. Ventrite 2 nearly perpendicular to ventrite 1; surface smooth, shining, finely punctate; punctures small, fine, shallow; surface flattened, depressed above basal margin; apical margin unarmed; covered in recumbent setae twice width of a puncture; lateral margins of ventrites 2–3 and ventrite 4 unarmed. Ventrite 5 carinate ridge closer to apical margin of segment; length of ventrite 5 less than combined lengths of ventrites 3 and 4; setal patch and median depression absent.

**Figure 37.**
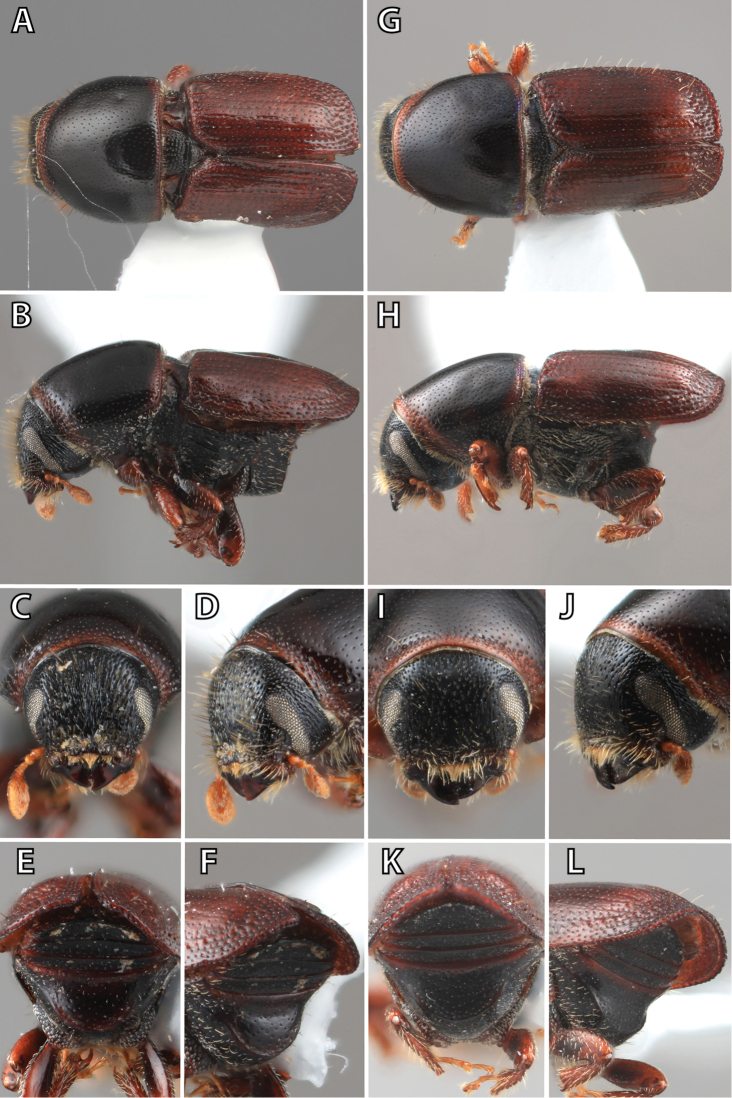
*Scolytus monticolae* **A** dorsal male habitus **B** lateral male habitus **C** male frons **D** male frons oblique **E** male venter **F** male venter oblique **G** dorsal female habitus **H** lateral female habitus **I** female frons **J** female frons oblique **K** female venter **L** female venter oblique.

##### Female.

2.3–3.5 mm long (mean = 3.0 mm; n = 10); 2.3–2.7 times as long as wide. Similar to male except epistoma feebly emarginated, frons convex when viewed laterally, strigate, setae sparser, shorter, less than width of eye; weakly transversely impressed between inner apices of eyes. Second ventrite unarmed. Length of ventrite 5 greater than combined lengths of ventrites 3 and 4.

##### Specimens examined.

123.

##### Type material.

Lectotype: female, labeled “[Arrowhead, British Columbia], *Pinus monticola*, 2423, J.M. Swaine Coll, female” (CNCI). Lectotype designated Bright 1967: 674.

##### Non-type material.

**CANADA:** ***BRITISH COLUMBIA*:** Merritt, Midday Valley, 27.VI.1926, W. Mathers (USNM-2), 4.IX.1926 (USNM-2). Trinity Valley, 4.VII.1954, ex. *Pseudotsuga taxifolia* (USNM-4). **UNITED STATES:** ***COLORADO*:** *Custer Co.*: Hillside, 6 mi W., Duckett Creek, FR331, Rita Alta Fuelwood area, 23.V.2000, D. Leatherman (CSUC-1). [*Garfield Co.*]: Glenwood Springs (CASC-1). *Jefferson Co.*: Buffalo Creek, 6.VIII.2004, D. Leatherman, ex. Douglas fir [= *Pseudotsuga menziesii*] (CASC-2). ***IDAHO*:** *Benewah Co.*: St. Maries, Hopk. U.S. 618074, 28.VIII.1978, M.M. Furniss, ex. *Pseudotsuga menziesii* (USNM-32). *Boise Co.*: Boise National Forest, Bogus Basin, Bogus Basin Rd, N43°44.347', W116°07.099', 6047 ft, 8.VIII.2010, S.M. Smith, A.R. Gillogly, ex. *Pseudotsuga menziesii* (MSUC-8). *Bonner Co.*: Priest River Experimental Forest, Hopk. U.S. 61809-A, 25.X.1978, M.M. Furniss, ex. *Pseudotsuga menziesii* (USNM-26). *Kootenai Co.*: Coeur d’Alene, 30.VIII.1919, J.C. Evenden (MSUC-12); 7.VIII.1919, J.C. Evenden, ex. *Pseudotsuga menziesii* (MSUC-4). *Shoshone Co.*: Coeur d’Alene National Forest, N47°25.708', W115°53.464', 3728 ft, 15.VIII.2010, S.M. Smith, A.R. Gillogly, ex. *Pseudotsuga menziesii* (MSUC-19). Prichard, 23.VII.1920, J.C. Evenden, ex. *Abies grandis* (MSUC-2). ***MONTANA*:** [*Sanders Co.*]: Trout Creek, 1.VIII.1981, J. Dunkel, ex. Douglas fir (USNM-2). ***OREGON*:** [*Unspecified County*]: Santiam National [State] Forest, 22.VIII.[19]14, W.J. Chamberlin, ex. *Abies amabalis* (EMEC-1). ***WASHINGTON*:** [*Kittitas Co.*]: Easton (USNM-1). [*Yakima Co.*]: Cliffdell, 7.VII.[19]35, R.H. Beaner (USNM-1). ***WYOMING*:** [*Park Co.*]: Cody, Hopk. U.S. 34220-F, 10.V.[19]56, H.E. Ostmark, ex. *Pseudotsuga taxifolia* [= *Pseudotsuga menziesii*] (CSUC-2).

##### Distribution.

**Figure 38.**
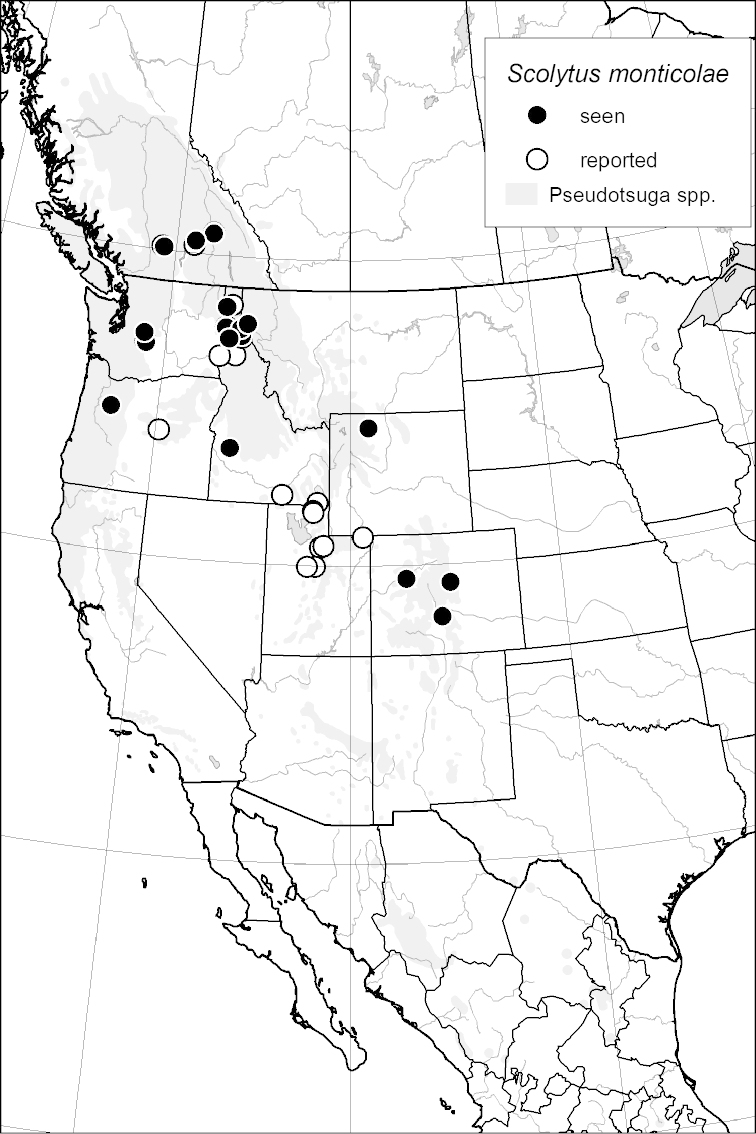
*Scolytus monticolae* distribution map.

CANADA: British Columbia. UNITED STATES: Colorado, Idaho, Montana, Oregon, Utah, Washington, Wyoming (Fig. 38).

##### Hosts.

*Pseudotsuga menziesii* (Mirb.) Franco (Douglas fir).

##### Biology.

*Scolytus monticolae* commonly infests the bole, suppressed branches and fresh slash of Douglas fir. *Scolytus monticolae* (as *Scolytus tsugae*, see remarks below) has been reported to kill drought stressed sapling and pole-sized Douglas fir trees in British Columbia (McMullen and Atkins 1959) and Wyoming (Furniss and Carolin 1977).

Adult galleries strongly resemble those of *Scolytus reflexus*, parallel to the grain of the wood, bayonet shaped and consist of two egg galleries and a central nuptial chamber (Fig. 24). From the central nuptial chamber, one egg gallery extends with the grain of the wood and the other egg gallery is slightly transversely extended and then is extended parallel to the grain. The nuptial chamber is oblique to the egg galleries. The adult gallery scores the sapwood more than the cambium and averages 5.0–9.0 cm in length. Egg niches are generally placed in pairs along the egg galleries and score the sapwood. Larvae extend their mines perpendicular to the egg gallery before diverging in a fan shaped pattern. Pupation may occur under the bark or in the sapwood. In Idaho, *Scolytus monticolae* has one generation per year with flight occurring in July (Furniss and Johnson 2002; Smith, pers. obs.). Broods overwinter as larvae and emerge the following summer (Edson 1967; Furniss and Johnson 2002).

##### Collection notes.

The senior author found this species to be common in suppressed limbs and slash in Idaho.

##### Remarks.

The lectotype does not bear a locality label. Swaine’s (1917) description states the lectotype was collected at Arrowhead, British Columbia.

In their paper describing the biology of *Scolytus tsugae*, McMullen and Atkins (1959) considered *Scolytus monticolae* a synonym of *Scolytus tsugae* based on correspondence with G.R. Hopping (page 417). Wood (1966: 30) formally synonymized *Scolytus monticolae* with *Scolytus tsugae*, and later removed the species from synonymy (Wood 1982; M.M. Furniss, pers. comm.). However, Wood (1982) did not explicitly state that he was the author that recognized the species. These two species are closely related and have subtle morphological differences. This has led to a confusing account of both species in the literature. The majority of which seems to be applicable to *Scolytus monticolae* rather than *Scolytus tsugae*. The above diagnostic characters and different biologies readily differentiate the species.

#### Scolytus mundus

Taxon classificationAnimaliaColeopteraCurculionidae

Wood, 1968

Fig. 39

Scolytus mundus Wood, 1968: 13.

##### Diagnosis.

This species most strongly resembles *Scolytus aztecus* and *Scolytus ventralis*. Both sexes are differentiated by elytral apices slightly emarginated only at interstria 3, by ventrite 3 covered with abundant erect setae that are greater than length of segment 3. The male can also be differentiated by the apical margin of ventrite 2 armed with a broad median denticle; larger size, the geographic distribution.

##### Description (male).

4.0–4.5 mm long (mean = 4.35 mm; n = 4); 2.1–2.5 times as long as wide. Color dark brown to black, antennae red brown. Pronotum same color as elytra.

*Head.* Epistoma weakly emarginated; epistomal process absent; median area above mandibles bearing dense patch of long, yellow, hair-like setae. Frons appearing convex when viewed laterally, moderately, transversely impressed just above epistoma; strongly, coarsely longitudinally, aciculate-punctate; aciculations converging at epistoma; punctures small, fine; densely, uniformly covered by long, fine, yellow, erect, hair-like setae, setae longer than width of midpoint of eye. Antennal scape short, elongate; club flattened, nearly subquadrate, setose with partial septum, two arcuate sutures visible.

*Pronotum* as long as wide; apical margin broadly rounded, median area between eyes lined with scales; sides distinctly arcuate, strongly constricted near apex, forming a weak transverse impression near apical margin; surface smooth, shining, punctures on disc fine, shallow, moderately abundant, larger and more abundant laterally and on apical constriction; apical, anterolateral and lateral margins bearing abundant, erect, long, dark yellow-brown, hair-like setae; base weakly bisinuate.

*Elytra* with sides sub-parallel on apical half, narrowing to subquadrate, smooth apex; elytral apices slightly emarginated at interstria 3; apex weakly emarginated at suture. Margin of apical edge bearing large, coarse punctures. Disc smooth, shining; interstriae weakly impressed, more than twice width of striae, punctures uniseriate, equal in size to those of striae, bearing moderately abundant long, erect, dark yellow-brown, hair-like setae; striae weakly impressed. Declivity bearing sparse, short, erect yellow setae. Metepimeron half-length of metanepisternum.

*Venter.* Apical margin of ventrite 1 weakly elevated above base of ventrite 2. Ventrite 2 nearly perpendicular to ventrite 1; surface smooth, shining, finely punctate; punctures small, fine, shallow; covered with abundant erect setae greater than length of segment 3; surface flattened; apical margin armed with broad median denticle; lateral margins of ventrites 2–3 and ventrite 4 unarmed. Ventrite 5 carinate ridge closer to apical margin of segment; length of ventrite 5 greater than combined lengths of ventrites 3 and 4; setal patch and median depression absent.

**Figure 39.**
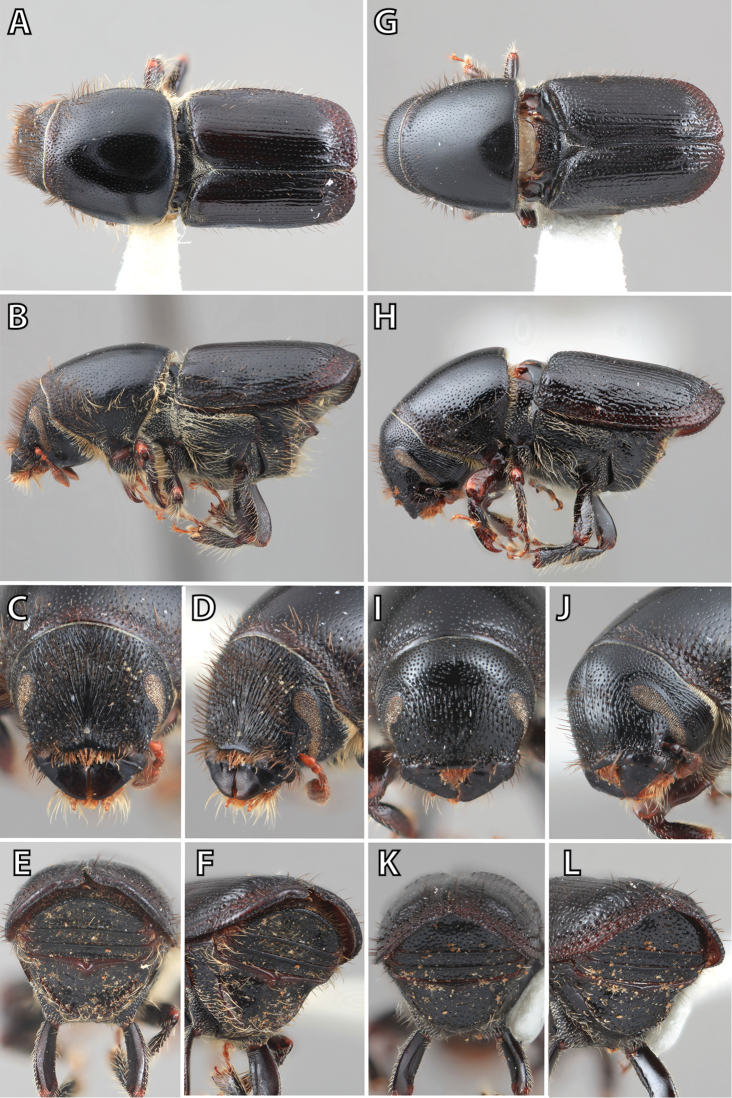
*Scolytus mundus* **A** dorsal male habitus **B** lateral male habitus **C** male frons **D** male frons oblique **E** male venter **F** male venter oblique **G** dorsal female habitus **H** lateral female habitus **I** female frons **J** female frons oblique **K** female venter **L** female venter oblique.

##### Female.

3.3–4.6 mm long (mean = 4.0 mm; n = 13); 1.8–2.6 times as long as wide. Similar to male except epistoma feebly emarginated, epistomal process absent, frons convex when viewed laterally, weakly longitudinally aciculate, setae sparser, shorter, less than width of eye; weakly transversely impressed between inner apices of eyes. Apical margin of second ventrite armed by small broad tumescence.

##### Specimens examined.

28.

##### Type material.

Holotype: male, labeled “11 mi, N Tlaxco, Puebla, Mexico, SL W[ood], *Abies religiosa*.” (USNM). Paratypes: **MEXICO:** [***Puebla***]: Tlaxco (Tlaxcala), 11 mi N., 8900 ft, 9.VII.1967, S.L. W[ood], ex. *Abies religiosa* (USNM-21).

##### Non-type material.

**MEXICO:** [***Distrito Federal***]: Desierto de los Leones National Park, III.1951, J.M. Miller, ex. *Abies religiosa* (OSAC-2). ***Hidalgo*:** El Chico, 31.IX.[19]77, E. Hernandez V., ex. *Abies religiosa* (CNCI-2). ***Oaxaca*:** Valle Nacional, 53 mi S., 10000 ft, 24.V.[19]71, D.E. Bright, ex. *Abies religiosa* (CNCI-2). ***Tlaxcala*:** Villarreal Terrenate, 23.II.[19]78, E. Hernandez, ex. *Abies religiosa* (USNM-4, WFBM-5).

##### Distribution.

**Figure 40.**
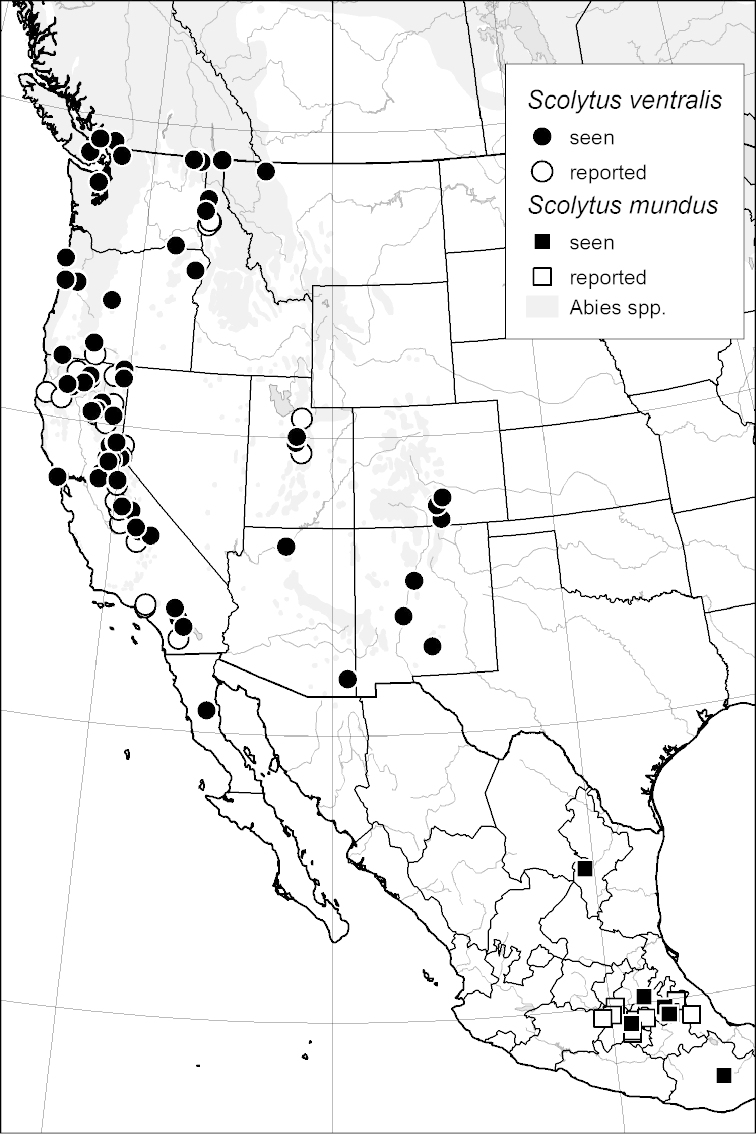
*Scolytus mundus* and *Scolytus ventralis* distribution map.

MEXICO: Distrito Federal, Estado de México, Hidalgo, Michoacán, Morelos, Oaxaca, Puebla, Tlaxcala (Fig. 40).

##### Hosts.

*Abies religiosa* (Kunth) Schltdl. & Cham. (sacred fir).

##### Biology.

*Scolytus mundus* is a serious pest of *Abies religiosa* in Mexico and its life cycle and habits have been well-studied (see Cibrián Tovar et al. 1995). *Scolytus mundus* attacks the tops of mature trees, trunks of recently cut trees and large logging slash greater than 10.0 cm in diameter (Wood 1968; Cibrián Tovar et al. 1995). Trees attacked by *Scolytus mundus* exhibit red foliage at the tree top. If the population density is high, subsequent generations will colonize the tree from the top down. However, attacks rarely occur at the base and so the host tree typically survives (Cibrián Tovar et al. 1995). *Scolytus mundus* has been reported to colonize the same material as *Scolytus hermosus* (Wood 1968).

The female initiates the attack by constructing an entrance in bark crevices at branch nodes. The adult galleries are transverse across the grain of the wood, similar in appearance to *Scolytus ventralis* and with a central nuptial chamber (see Cibrián Tovar et al. 1995, figs 130–131). Eggs are laid in niches excavated on each side of the central nuptial chamber. The larval mines extend parallel to the grain of wood and are first in the cambium and later penetrate into the sapwood. Pupation occurs in the sapwood and there are two generations per year with the first generation occurring from October to May and the second from June to October (Cibrián Tovar et al. 1995).

#### Scolytus obelus

Taxon classificationAnimaliaColeopteraCurculionidae

Wood, 1962

Figs 29 , 41

Scolytus obelus Wood, 1962: 81.

##### Diagnosis.

Both sexes of *Scolytus obelus* strongly resemble *Scolytus praeceps* and are distinguished by the presence of a small median denticle on the apical margin of ventrite 2.

##### Description (male).

1.8–2.9 mm long (mean = 2.2 mm; n = 20); 2.1–2.9 times as long as wide. Color red brown to dark red-brown, antennae brown. Pronotum typically darker than elytra.

*Head.* Epistoma weakly emarginate; epistomal process weakly developed, low; median area above mandibles bearing dense patch of long, yellow, hair-like setae. Frons appearing convex when viewed laterally, slightly transversely impressed just above epistoma; moderately, coarsely aciculate-punctate; aciculations converging at epistoma; punctures small, coarse; moderately, uniformly, covered by long, fine, yellow, erect hair-like setae, these longer than width of midpoint of eye. Antennal scape short, elongate; club flattened, ovoid, setose with partial septum, three arcuate sutures visible.

*Pronotum* wider than long; apical margin broadly rounded, median area between eyes lined with scales; sides distinctly arcuate, strongly constricted near apex, forming a weak transverse impression near apical margin; surface smooth, shining, punctures on disc fine, shallow, moderately abundant, larger and more abundant laterally and on apical constriction; apical and anterolateral margins bearing sparse, erect, yellow, hair-like setae; base weakly bisinuate.

*Elytra* with sides sub-parallel on apical half, narrowing to subquadrate, weakly serrate apex; apex weakly emarginated at suture. Margin of apical edge bearing large, coarse punctures. Disc glabrous, smooth, shining; interstriae not impressed, more than twice width of striae, punctures uniseriate, smaller than those of striae; striae not impressed. Declivity bearing sparse, short, erect yellow setae. Metepimeron half-length of metanepisternum.

*Venter.* Apical margin of ventrite 1 strongly, acutely produced, forming lip along base of ventrite 2, basal margin of ventrite 2 appearing impressed. Apical margin of ventrite 1 weakly elevated above base of ventrite 2. Ventrite 2 nearly perpendicular to ventrite 1; surface shagreened, dull, finely punctate; punctures small, fine, shallow, punctures varying in size, larger and more abundant near basal margin; surface weakly concave; apical margin armed with acute median denticle; setae short, recumbent, about three times that of a puncture in length; lateral margins of ventrites 2–3 and ventrite 4 unarmed. Ventrite 5 carinate ridge closer to apical margin of segment; length of ventrite 5 equal to combined lengths of ventrites 3 and 4; setal patch and median depression absent.

**Figure 41.**
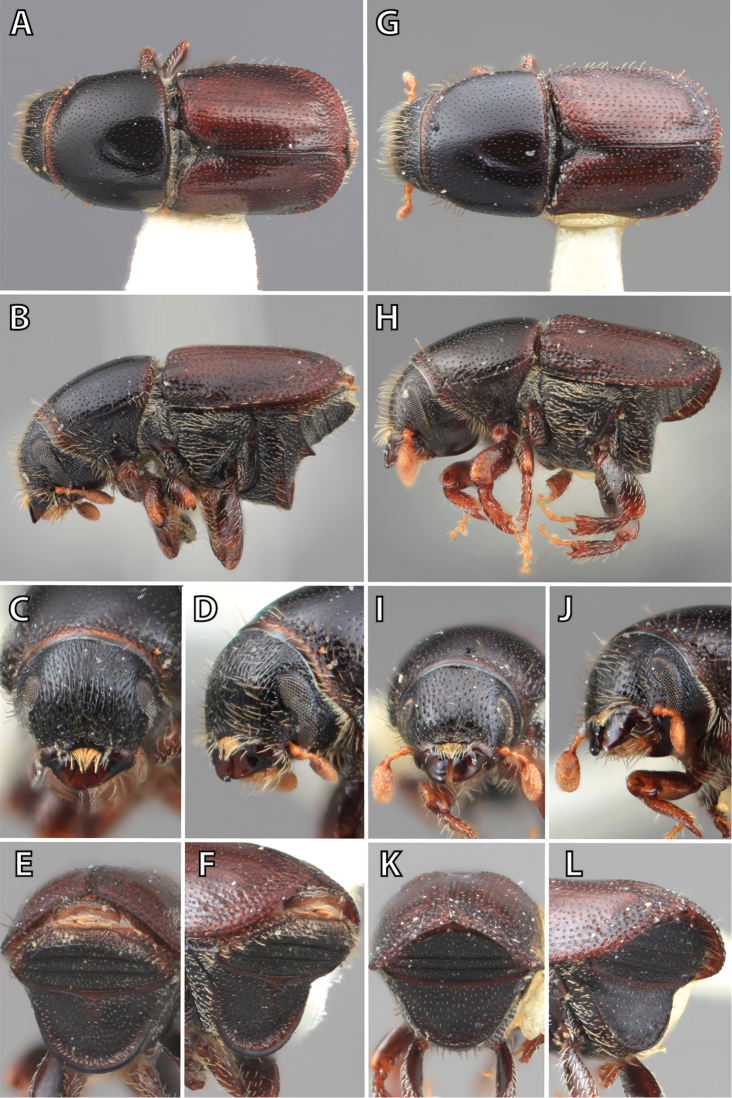
*Scolytus obelus* **A** dorsal male habitus **B** lateral male habitus **C** male frons **D** male frons oblique **E** male venter **F** male venter oblique **G** dorsal female habitus **H** lateral female habitus **I** female frons **J** female frons oblique **K** female venter **L** female venter oblique.

##### Female.

1.7–3.0 mm long (mean = 2.3 mm; n = 20); 2.0–2.7 times as long as wide. Similar to male except epistoma feebly emarginate, epistomal process absent, frons convex when viewed laterally, weakly aciculate, setae sparser, shorter, less than width of eye; weakly transversely impressed between inner apices of eyes. Apical margin of ventrite 1 moderately elevated above base of ventrite 2. Second ventrite armed with median tumescence or minute denticle.

##### Specimens examined.

138.

##### Type material.

Holotype: male, labeled “Payson Canyon, UT, S.L Wood, 14 May 1960, *Abies concolor*, BLNO 001271” (USNM). Paratypes *Scolytus obelus* **UNITED STATES:** ***UTAH*:** [*Beaver Co.*]: Beaver, 22.IV.1950, S.L. Wood, ex. *Abies concolor* (USNM-4). *Utah Co.*: Payson Canyon, 14.V.1960, S.L. Wood, ex. *Abies concolor* (USNM-6), 25.VI.1962 (USNM-2).

##### Non-type material.

**United States:** ***Arizona*:** *Coconino Co.*: Coconino National Forest, Arizona Snow Bowl, N35°19.593', W111°42.681', 9230 ft, 27.V.2010, S.M. Smith, ex. *Abies lasiocarpa* [var. *arizonica*] (MSUC-62). Jacob Lake, 12 mi S., 31.V.1969, W. Harwood, ex. *Abies concolor* branch (USNM-4). *Graham Co.*: Pinaleno Mountains, 15.VII.1968, D.E. Bright, ex. *Pseudotsuga menziesii* (DEBC-3). [Coronado National Forest], [Pinaleno Mountains], Mount Graham, Hospital Flat, 9050 ft, 19.VIII.1952, H.B. Leech, J.W. Green (CASC-1), 8950 ft, 3.VIII.1965, H.B. Leech (CASC-5). *Yavapai Co.*: Prescott, 1 mi S., 3.VIII.1962, S.L. W[ood] (USNM-1). Prescott, 9 mi S., 5 mi E., 7800 ft, 5.VI.1969, W. Harwood, ex. *Abies concolor* (USNM-6). [*Unspecified County*]: Santa Catalina Mountains, 8500 ft, 31.V.1969 (DEBC-1, FSCA-4). ***COLORADO*:** *Costilla Co.*: near Fort Garland, Forbes Trinchera Ranch, VII-VIII.1976, D. Leatherman, ex. white fir [= *Abies concolor*] (CSUC-4). *Huerfano Co.*: near Red Wing, 16.VII.1975, D. Leatherman, ex. white fir [= *Abies concolor*] (CSUC-2). ***NEVADA*:** *Clark Co.*: Mary Jane Falls, 7900 ft, 11.VI.1969, Harwood, ex. *Abies concolor* (USNM-7). [*White Pine Co.*]: Baker, S9 T13N R69E, Mount Diablo Meridian, 17.V.1917, T.O. Thatcher, ex. *Abies lasiocarpa*, LCNM 39-12 (CSUC-8, USNM-1, WFBM-1). Mount Wheeler, 19.VIII.1974, S.L. Wood, ex. *Abies concolor* (USNM-9). ***NEW MEXICO*:** [*Otero Co.*]: Cloudcroft, 14-26.VII.[19]49, W.B.R. Stromberg, ex. fir tree [= *Abies* sp.] (USNM-1). [*Unspecified County*]: Sandia Mountains, 8090 ft, 29.V.1969, S.L. W[ood], ex. *Abies concolor* (USNM-3), 30.V.1969 (USNM-9). ***UTAH*:** *Utah Co.*: Provo, Payson Canyon, VIII.1964, ex. *Abies concolor* (EMEC-4). [*Unspecified County*]: Bryce Canyon National Park, Hopk. U.S. 35-043, 11.VII.1952, R. Washburn, ex. *Abies concolor* (EMEC-2).

##### Distribution.

UNITED STATES: Arizona, Colorado, Nevada, New Mexico, Utah (Fig. 27).

##### Hosts.

*Abies concolor* (Gord. & Glend.) Lindl. ex Hildebr. (white fir) and Abies lasiocarpa var. arizonica (Merriam) Lemmon (corkbark fir). Incidental host: *Pseudotsuga menziesii*.

##### Biology.

*Scolytus obelus* is an uncommonly encountered species. The species prefers to colonize the limbs, tops and slash of its host. Infested material ranges from 3.0–10.0 cm in diameter (Wood 1982).

The adult gallery contains two egg galleries that branch from the central nuptial chamber. One egg gallery extends from the nuptial chamber perpendicular to the grain of wood and the second gallery extends at a 45° angle to the grain (Fig. 24). Galleries are frequently initiated near branch crotches and disguised under rough patches of bark. The adult gallery deeply scores the sapwood and lightly scores the cambium. The adult galleries range in size from 1.9–6.5 cm in length. Eggs are laid in niches on both sides of each egg gallery and larval mines radiate perpendicular to the egg gallery forming a fan shaped pattern. The larval mines gradually diverge and lightly etch the sapwood. Pupation occurs in the sapwood (Edson 1967).

##### Collection notes.

The senior author collected specimens from suppressed 2.0–4.0 cm diameter branches of Abies lasiocarpa var. arizonica that had been girdled at the base by porcupines. Needles of infested branches were pale green to yellow.

##### Remarks.

This species is sister to *Scolytus praeceps* and is very similar both in morphology and gallery architecture. Interspecific divergence in COI nucleotide difference between these two taxa is quite large and averages 10.36% (Table 7).

#### Scolytus oregoni

Taxon classificationAnimaliaColeopteraCurculionidae

Blackman, 1934

Figs 29 , 42

Scolytus oregoni Blackman, 1934: 18.

##### Diagnosis.

*Scolytus oregoni* is a rather distinctive species and both sexes are readily distinguished by having the apical margin of ventrite 1 thickened and on the surface of ventrite 2 and by the unarmed ventrite 2.

##### Description (male).

2.6–3.6 mm long (mean = 3.3 mm; n = 20); 2.0–2.4 times as long as wide. Color dark red-brown, antennae brown. Pronotum typically darker than elytra.

*Head.* Epistoma weakly emarginate; epistomal process weakly developed; median area above mandibles bearing dense patch of long, yellow, hair-like setae. Frons appearing flattened when viewed laterally, slightly transversely impressed just above epistoma; moderately, coarsely, longitudinally aciculate-punctate; aciculations converging at epistoma; punctures sparse, small, coarse; moderately, uniformly covered by long, fine, yellow, erect, hair-like setae, these longer than width of midpoint of eye. Antennal scape short, elongate; club flattened, irregularly ovoid, setose with partial septum, two strongly arcuate sutures visible.

*Pronotum* wider than long; apical margin broadly rounded, median area between eyes lined with scales; sides distinctly arcuate, strongly constricted near apex, forming a weak transverse impression near apical margin; surface smooth, shining, punctures on disc fine, shallow, moderately abundant, larger and more abundant laterally and on apical constriction; apical and anterolateral margins bearing sparse, erect, yellow, hair-like setae; base weakly bisinuate.

*Elytra* with sides sub-parallel on apical half, narrowing to subquadrate, smooth apex; apex weakly emarginated at suture. Margin of apical edge bearing large, coarse punctures. Disc glabrous, smooth, shining; interstriae not impressed, more than twice width of striae, punctures uniseriate, smaller than those of striae; striae weakly impressed. Declivity bearing sparse, short, erect, yellow setae. Metepimeron less than half-length of metanepisternum.

*Venter.* Apical margin of ventrite 1 not elevated above base of ventrite 2. Basal margin of ventrite 2 strongly thickened, lip-like; ventrite 2 nearly perpendicular to ventrite 1; surface shagreened, dull, finely punctate; punctures small, fine, shallow; surface weakly concave, weakly to strongly medially impressed just above base; apical margin unarmed; setae small, less than 1 diameter of a puncture; lateral margins of ventrites 2–3 and ventrite 4 unarmed. Ventrite 5 carinate ridge closer to apical margin of segment; length of ventrite 5 equal to combined lengths of ventrites 3 and 4; setal patch and median depression absent.

**Figure 42.**
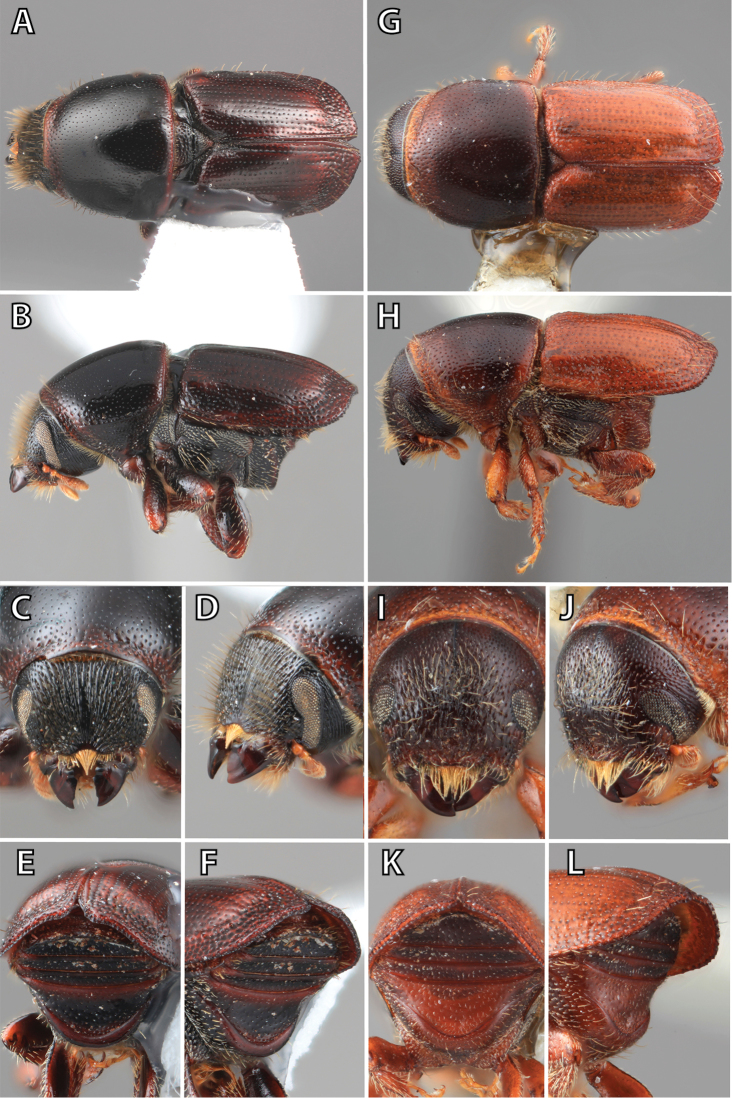
*Scolytus oregoni* **A** dorsal male habitus **B** lateral male habitus **C** male frons **D** male frons oblique **E** male venter **F** male venter oblique **G** dorsal female habitus **H** lateral female habitus **I** female frons **J** female frons oblique **K** female venter **L** female venter oblique.

##### Female.

2.8–4.0 mm long (mean = 3.23 mm; n = 20); 1.6–2.7 times as long as wide. Similar to male except epistoma entire, epistomal process absent, frons convex when viewed laterally, weakly longitudinally aciculate, setae sparser, shorter, less than width of eye; weakly transversely impressed between inner apices of eyes. Second ventrite unarmed, basal margin weakly thickened.

##### Specimens examined.

96.

##### Type material.

Holotype, male, labeled “Ashland Ore., May 20/19, Glendinning, WE Colr, *Pseudotsuga taxifolia*, Hopk. US. 13399a, Type No. 43834 USNM” (USNM). Paratypes: **UNITED STATES:** ***Oregon*:** [*Jackson Co.*]: Ashland, Hopk. U.S. 14246-C, 10.III.[19]16, P.D. Sergent, ex. *Pseudotsuga taxifolia* (CNCI-2); II.[19]19, W.E Glendinning, Hopk. U.S. 1399-A (USNM-14).

##### Non-type material.

**UNITED STATES:** ***CALIFORNIA*:** [*Del Norte Co.*]: [Six Rivers National Forest], Gasquet R.S. [= Ranger Station], Hopk. U.S. 31722-H, 10.VII. R.L. Furniss, ex. *Pseudotsuga taxifolia* (OSAC-23). *Lake Co.*: Middletown, 11.XI.1959, G.M. Thomas, ex. *Pseudotsuga menziesii* (OSAC-1). *Los Angeles Co.*: Angeles National Forest, Sawmill Mountain, 34.6926°N, 118.5499°W, V.28-VI.14.2007, Caterino, Chatzimanolis, ex. Lindgren trap (SBMN-4). *Marin Co.*: Alpine Lake, V.[19]57 (CASC-4). Mount Tamalpais 14.IX.[19]57, E.L. Smith, ex. *Pseudotsuga menziesii* (OSAC-1). Woodacre Creek, 9.VII.1951, P.S. Bartholomew (CASC-1). *Napa Co.*: Angwin, 2 mi N.N.E., N. side of Howell Mountain, 1300 ft, 16.VII.1974, H.B. Leech, ex. emerged from log of *Pseudotsuga menziesii* (CASC-1), 21.VII.1974 (CASC-4, USNM-5), 22.VII.1974 (CASC-1), 25.VII.1974 (CASC-4, USNM-1), 7.VIII.1983 (CASC-1), 12.IX.1983 (CASC-2). Callistoga, 5.X.1947, S.L. Wood, ex. *Pseudotsuga taxifolia* (USNM-2). Mount Saint Helena, Hopk. U.S. 15401-A, F.B. Herbert, ex. *Pseudotsuga taxifolia* (OSAC-1). *Riverside Co.*: Santa Barbara National Forest, Black Mountain Rd, 33.8395°N, 116.7306°W, 1.VII.2005, M. Caterino (SBMN-1). [*Valyermo Co.*]: Fenner Canyon, Hopk. U.S. 33853-A, 15.VIII.[19]51, A.D. Moore, ex. *Pseudotsuga macrocarpa* (EMEC-3, OSAC-8, USNM-4). ***OREGON*:** *Benton Co.*: Marys Peak, Corvallis Watershed, 9.VIII.1963, ex. rotary traps (EMEC-1). *Jackson Co.*: Ashland, Hopk. U.S. 13363-B, 26.VI.1918, W.E.G, ex. *Pseudotsuga taxifolia*, (OSAC-14). Mistletoe, Hopk. U.S. 15753-A, P.D. Sergent, ex. *Pseudotsuga taxifolia* [= *Pseudotsuga menziesii*] (OSAC-3). Rogue River National Forest, Rogue River Gorge viewpoint, N42°54.540', W122°26.733', 3489 ft, 21.VIII.2010, S.M. Smith, ex. *Pseudotsuga menziesii* (MSUC-3). *Linn/Lane Co.*: Blue River, 11 mi N.E., H.J. Andrews experimental forest, 5.VIII.1986, Log Decomp Study, Site 2, SE1/4 S15 T15S RSE, Trap 2WA (OSAC-1). [*Tillamook Co.*]: Woods, 18.XI.[19]38 (OSAC-1). *Wasco Co.*: The Dalles, ODA Port/Mill survey, Trap #65-01a, 14.VIII.1997, ex. Lindgren funnel with α-pinene & ethanol lure (MSUC-1).

##### Distribution.

UNITED STATES: California, Oregon, Washington (Fig. 30).

##### Hosts.

*Pseudotsuga menziesii* (Mirb.) Franco (= *Pseudotsuga taxifolia* Britton) (Douglas fir) and *Pseudotsuga macrocarpa* (Vasey) Mayr (bigcone Douglas fir).

##### Biology.

*Scolytus oregoni* colonizes large limbs and tops of its host and also fresh slash (Edson 1967; Smith, pers. obs.).

The adult gallery is typically constructed parallel to the grain of the wood and has a central nuptial chamber (Fig. 24). The gallery structure is typically bayonet shaped but may also be longitudinal. Each egg gallery extends in opposite directions to the grain of the wood from the central nuptial chamber. The nuptial chamber is transverse to the egg galleries. The adult gallery deeply scores the sapwood and lightly scores the cambium. The adult gallery averages 6.0–18.0 cm in length. Egg niches are closely spaced and deeply score the sapwood. Larvae extend their mines perpendicular to the egg gallery in a fan shaped pattern before terminating in pupation chambers, which are constructed in the sapwood (Edson 1967).

##### Remarks.

Specimens of *Scolytus oregoni* are very rarely collected and the species is perhaps the least common of the conifer-feeders in the United States. There are many gaps that occur in its distribution range, particularly between northern and southern California. Considerable variation is observed in the male ventrite 1 and 2 across the geographic range especially between northern California, Oregon and Washington and southern California. Southern California populations colonize *Pseudotsuga macrocarpa* while individuals from the rest of the range colonize *Pseudotsuga menziesii*. In addition, *Scolytus oregoni* males from California and particularly southern California have a greater impression of the second ventrite and thicker margin between ventrite 1 and 2 to specimens from Oregon and Washington.

#### Scolytus piceae

Taxon classificationAnimaliaColeopteraCurculionidae

(Swaine, 1910)

Figs 29 , 43

Eccoptogaster piceae Swaine, 1910: 34.
Scolytus piceae (Swaine, 1910): Blatchley and Leng 1916: 589.

##### Diagnosis.

Both sexes are easily diagnosed by the large conical median spine on the surface of the second ventrite, the spine base never touches any margin, by the lack of lateral denticles on the apical margins of ventrites 2–4 and by the unicolorous elytra.

##### Description (male).

2.5–3.0 mm long (mean = 2.4 mm; n = 12); 2.3–2.7 times as long as wide. Color red-brown to dark red brown, antennae yellow-brown, legs dark red-brown to yellow brown apically. Pronotum typically darker than elytra.

*Head.* Epistoma weakly emarginate; epistomal process present, moderately developed, low; median area above mandibles bearing dense patch of long, yellow, hair-like setae. Frons appearing flattened when viewed laterally from epistoma to vertex, slightly transversely impressed just above epistoma to inner apices of eyes; moderately, coarsely longitudinally aciculate-punctate; aciculations converging at epistoma; punctures large, dense, coarse; moderately, uniformly covered by long, fine, yellow, erect, hair-like setae, these longer than width of midpoint of eye. Antennal scape short, elongate; club flattened, irregularly ovoid, setose with partial septum, two arcuate sutures visible.

*Pronotum* wider than long; apical margin broadly rounded, median area between eyes lined with scales; sides distinctly arcuate, strongly constricted near apex, forming a weak transverse impression near apical margin; surface smooth, shining, punctures on disc fine, shallow, moderately abundant, larger and more abundant laterally and on apical constriction; apical and anterolateral margins bearing sparse, erect, yellow, hair-like setae; base weakly bisinuate.

*Elytra* with sides sub-parallel on apical half, narrowing to subquadrate, smooth apex; apex weakly emarginated at suture. Margin of apical edge bearing large, coarse punctures. Disc glabrous, smooth, shining; interstriae weakly impressed, more than twice width of striae, punctures uniseriate, smaller than those of striae; striae weakly impressed. Declivity bearing sparse, short, erect, yellow setae. Metepimeron half-length of metanepisternum.

*Venter.* Apical margin of ventrite 1 rounded, marked by weak carina on ascendant part of venter, more strongly marked laterally. Ventrite 2 nearly perpendicular to ventrite 1; surface reticulate, shagreened, dull, finely punctate; punctures small, fine, shallow punctures; covered with sparse setae that are about twice size of a puncture; surface convex; armed with large conical median spine, apex rounded; lateral margins of ventrites 2–3 and ventrite 4 unarmed. Ventrite 5 carinate ridge closer to apical margin of segment; length of ventrite 5 less than combined lengths of ventrites 3 and 4; setal patch and median depression absent.

**Figure 43.**
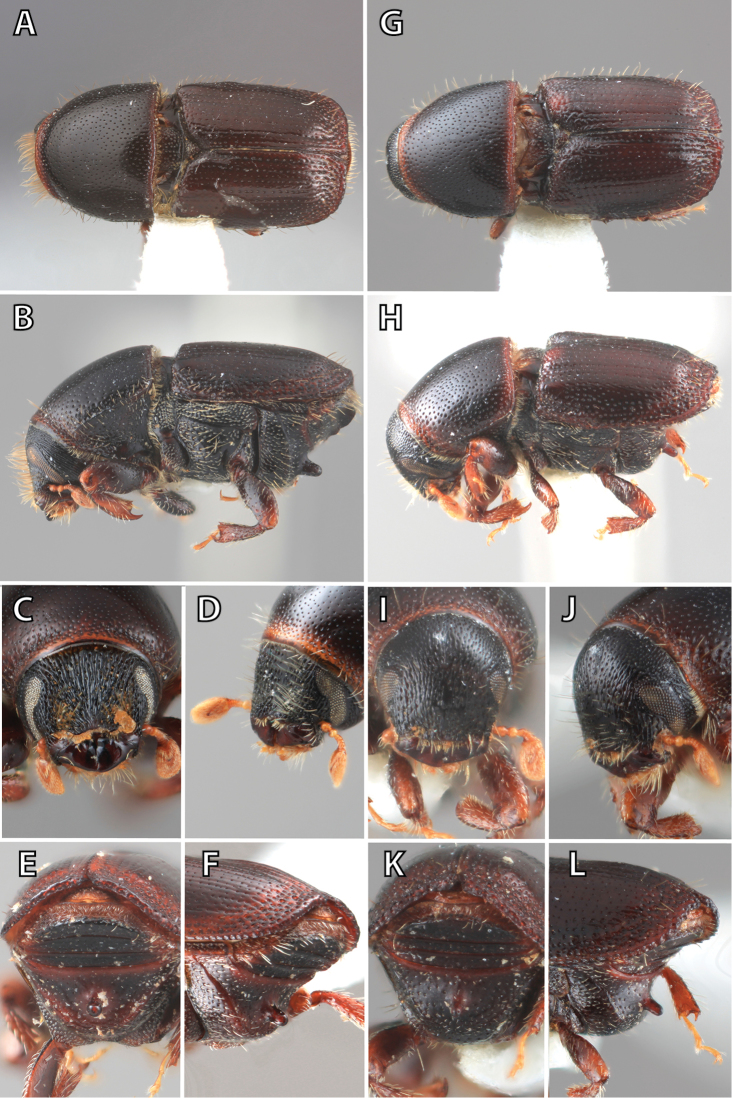
*Scolytus piceae* **A** dorsal male habitus **B** lateral male habitus **C** male frons **D** male frons oblique **E** male venter **F** male venter oblique **G** dorsal female habitus **H** lateral female habitus **I** female frons **J** female frons oblique **K** female venter **L** female venter oblique.

##### Female.

2.3–3.0 mm long (mean = 2.3 mm; n = 12); 2.3–2.7 times as long as wide. Similar to male except epistoma feebly emarginate, epistomal process weakly developed, frons convex when viewed laterally, weakly longitudinally aciculate, less coarsely punctate, setae sparser, shorter, less than width of eye; weakly transversely impressed just above epistoma. Second ventrite armed with smaller, rounded, median spine.

##### Specimens examined.

467.

##### Type material.

Lectotype: male, labeled “St. Anne’s, Que., July 21, 1907” (CUIC). Lectotype designated Bright 1967: 674. Paralectotype (**here designated**) *Eccoptogaster piceae*, female, **CANADA:** ***QUEBEC*:** St. Anne’s, 21.VII.1907 (CUIC-1). Paratypes: **CANADA:** ***QUEBEC*:** Hudson, 191[sic!] (CUIC-4).

##### Non-type material.

**CANADA:** ***ALBERTA*:** Banff (CNCI-2); IX.1916 (CNCI-1); 7.IX.1967, D.E. Bright, ex. *Picea glauca* (CNCI-1). Cypress Hills, 16.VII.[19]32 (CNCI-3). Edmonton, 16.I.1916, Carr (CNCI-1), 27.VIII.1916 (CNCI-1), 28.VIII.1916 (CNCI-1), 5.VII.1924, (CUIC-1); 6.XII.1916 (CASC-2); 15.VII.[19]24 (CASC-2); 29.VI.[19]16, J.W. Green (CASC-3), 2.IX.[19]16 (CASC-1). [Jasper National Park], Athabasca Falls, 9.VIII.1919, J.M. Swaine (CNCI-5). Medicine Hat, 2.IX.[19]26 (CNCI-2). Seebe, 7.VII.1966, D.F.J. Hilton, ex. *Picea glauca* (CNCI-8). ***BRITISH COLUMBIA*:** Hixon, 9.VII.1972, D.E. Bright, ex. *Picea* sp. (CNCI-2). [Big Creek Provincial Park], Lorna [Lake], 6.VII.1924, G. Hopping, ex. *Picea engelmannii* (CASC-1), 10.VIII.1924 (CASC-1), 22.VII.1925 (CASC-1), 14.VII.1926, ex. *Pinus contorta* (CASC-1), 9.VII.1926 (CASC-1); 10.VII.1926, H. Richmond ex. *Picea engelmannii* (CNCI-1), 14.VII.1926 (CNCI-1). Pine Pass, 11.VII.1972, D.E. Bright, ex. *Picea* sp. (CNCI-6). Trinity Valley, 21.VII.[19]30 (CNCI-1), 29.VII.[19]30 (CNCI-1), 13.VII.1928, J.R. Howell, ex. *Pinus monticolae* (CASC-1); 23.VII.[19]28, H. Richmond, ex. *Picea engelmannii* (CASC-1). ***MANITOBA*:** Aweme, 20.IX.[19]06, E. Criddle (CNCI-1); 1.VII.1916, N. Criddle, (CNCI-2) ex. bred from spruce [= *Picea* sp.], 2.VII.1916 (CNCI-4); 10.VII.[19]16 (CNCI-10) ex. spruce [= *Picea* sp.], 27.VII.[19]10 (CNCI-20), 14.VI.1918 (CNCI-2), 15.VI.1918 (CNCI-3); 5.VII.1916 (CNCI-3); 8.VII.1916 (CNCI-6), 9.VII.1916 (CNCI-1); 31.VII.1916 (CNCI-1); 9.VIII.1916 (CNCI-1); 10.IX.1917 (CNCI-10); 11.IX.1917 (CNCI-6); 9.VI.19[18] (CNCI-1). Brandon, 28.V.1940, L. Peterson, ex. *Picea canadensis* (CNCI-2). Gillam, 21.VII.950, W.J. Brown (CNCI-1). Glen Souris, 5.VI.[19]23, N. Criddle (CNCI-1). Grass River Provincial Park, 27.VII.1972, D.E. Bright, ex. *Pinus murrayana* [= *Pinus contorta murrayana*] (CNCI-7). Onah, 11.VII.1924, R.M. White (CNCI-3); N. Criddle, 25.VIII.[19]72 (CNCI-1). Telford, 24.VII.1963, ex. life table plot (CNCI-1), 2.VIII.1963 (CNCI-1). Winnipeg, Hanham (USNM-1). ***NEW BRUNSWICK*:** Kouchibouguac National Park, VIII.1977, D.E. Bright, Code-6224J (CNCI-1). McGraw Brook, 10 mi W., 7.VII.[19]70, D.E. Bright, ex. *Picea glauca* (CNCI-1). ***NEWFOUNDLAND*:** Deer Lake, 12 mi N.E., 23.VII.[19]70, D.E. Bright, ex. *Picea mariana* (CNCI-11). ***NOVA SCOTIA*:** Cape Breton Highlands National Park, MacIntosh Brook, PG703866, 21.VII.1983, D.E. & J.E. Bright, ex. *Picea glauca* (CNCI-7); 29.VII.1983, McKenzie, ex. *Picea* sp. (CNCI-10). Kejimkujik National Park, 16-17.VII.1967, D.E. Bright, ex. *Picea glauca* (CNCI-14). ***ONTARIO*:** Carp, 5.VIII.[19]66, D.E. Bright, ex. *Picea mariana* (CNCI-2). Lake of the Woods, 1.VIII.1972, D.E. Bright, ex. *Picea* sp. (CNCI-7). Ottawa, 24.VI.1913, J.M. Swaine, ex. crawling on dying *Larix* sp. (CNCI-1). Rainy River District, 3.VIII.[19]24, J.F. Brimley (CNCI-1). ***QUEBEC*:** Aylmer, 12.VII.1924, B.B. Watson, ex. *Picea glauca* (CNCI-1); 30.VII.1924, A.R. Graham (CNCI-1). Hudson, 191[sic!] (CNCI-52). Limbor/Touranine, 27.VI.1974, R. Sexton (CNCI-1). [Gaspésie National Park], Mount Albert, 28.VII.1954, W.J. Brown, ex. north base 650 ft (CNCI-1). St. Anne’s [Sainte-Anne-de-Beaupré], 19.VI.[19]19 (CASC-2, CNCI-42). South March, 19.VI.1958, S.D. Hicks (CNCI-1). ***SASKATCHEWAN*:** Big River, 23.VII.1972, D.E. Bright, ex. *Picea glauca* (CNCI-1). Big River, 40 mi N.W., 22.VII.[19]72, D.E. Bright, ex. *Picea glauca* (CNCI-1). Canoe Lake, 21.VII.1972, D.E. Bright, ex. *Picea glauca* (CNCI-2). Christopher Lake, 5.VIII.1959, A. & J. Brooks (CNCI-1). Cypress Hills, 3.IX.1967, D.E. Bright, ex. *Picea glauca* (CNCI-9); 24.IX.1964 (CNCI-2). ***YUKON*:** Old Crow, 28.VI.[19]81, D.E. Bright (CNCI-5). **UNITED STATES:** ***ALASKA*:** [*North Slope Borough*]: Prudhoe Bay Rd, 8 mi N., South Fork Koyukuk River, 67°13’N, 150°07’W, 1000 ft [sic!], 8.VII.1978, Smetana, Campbell (CNCI-1). *Fairbanks North Star Borough*: Fairbanks, 2.VIII.[19]55, W.F. McCambridge, ex. *Picea glauca* (DEBC-7). ***CALIFORNIA*:** *Siskiyou Co*: Callahan, 7 mi N.W., 16.VI.[19]63, D.E. Bright, ex. *Picea engelmannii* (DEBC-9, EMEC-6). Happy Camp, 18 mi N., 31.VII.[19]63, D.E. Bright, ex. *Picea engelmannii* (DEBC-4, EMEC-7). ***COLORADO*:** *Denver Co.*: Denver, Union Pacific, 14.IX.2000, USDA APHIS, ex. Lindgren funnel APEtOH Lure (CSUC-1). *Larimer Co.*: Livermore, 10.IX.2009, K. Smith, USDA APHIS, ex. Lindgren funnel APEtOH Lure, (CSUC-1). Roosevelt National Forest, Chambers Lake, 11.VIII.[19]68, 9200ft, L.A. Kelton (CNCI-1). ***MAINE*:** [*Oxford Co.*]: Wilsons Mills, 9.VIII.[19]70, D.E. Bright, ex. *Picea* sp. (CNCI-1). ***MASSACHUSETTS*:** [*Norfolk Co.*]: Dover, 8.VII.1933, C.W. Collins, ex. reared from blue spruce [= *Picea pungens*] (USNM-11). ***MINNESOTA*:** *Cook Co.*: Superior National Forest, Hwy 12 nr. Seagull Guard Station, 48°6'29"N, 90°50'12"W, 23.VI–9.VII.2003, K.J.K. Gandhi, ex. Lindgren funnel trap in *Pinus banksiana*, wind-disturbed-salvaged-logged, site D (CASC-1). *Mille Lacs Co.*: 2.VII.[19]36, H.R. Dodge, ex. under bark of tamarack tree [= *Larix* sp.] (CASC-2). ***MONTANA*:** Glacier National Park, 15.VII.[19]29 (CUIC-1). ***NEW MEXICO*:** [*Taos Co.*]: Red River, 3 mi W., C.C. Hoff (AMNH-1). ***NORTH DAKOTA*:** *Bottineau Co.*: S30 T162 R75, Hopk. U.S. 56561-A, 27.V.1973, A.D. Tagestad, ex. collected from *Picea glauca densata* (USNM-3). *Rolette Co.*: S15 T162 R69, Hopk. U.S. 56446, 19.V.[19]72, A.D. Tagestad, ex. collected from *Picea pungens* (USNM-2). ***OREGON*:** [*Jefferson Co.*]: Suttle Lake, 4 mi W., 3.IX.[19]39, Schuh, Gray, ex. *Picea engelmannii* (AMNH-12, FMNH-3). [*Unspecified County*]: Blue Mountains, 13.VII.[19]14, W.J. Chamberlain, ex. *Picea engelmannii* (CNCI-2). ***SOUTH DAKOTA*:** *Lawrence Co.*: near Leads, Brownsville Rd, N44.2922°, W103.7828°, 5650 ft, 27.VII.2004, K.P. Dole, ex. *Picea glauca* (MSUC-14). [*Unspecified County*]: Black Hills, 7.VII.[19]75, D.E. Bright, ex. *Picea glauca* (CNCI-5). ***UTAH*:** [*Daggett Co.*]: Mckee Draw, Ashley National Forest, 16.VI.1960, ex. *Picea pungens* (USNM-1). [*Unspecified County*]: Logan Canyon, S27 T13 NR4, 6.VII.1948, S.L. Wood, ex. *Picea engelmannii* (USNM-8). ***WYOMING*:** *Carbon Co.*: [Medicine Bow National Forest], Mirror Lake, 4 mi W., 6.IX.2010, D.E. Bright, B.A. Barr, ex. *Picea engelmannii* branches (DEBC-1); [Medicine Bow National Forest] Snowy Mountains, WY130, Lake Marie, N41°19.965', W106°19.516', 3208 m, 26.VII.2011, S.M. Smith, D.E. Bright, B.A. Barr, ex. emerged 1-5.IX.2011, ex. *Picea engelmannii* (MSUC-36). *Johnson Co.*: Buffalo, 16 km SW, 20.VI.1968, S.L. W[ood], ex. *Picea engelmannii* (USNM-1).

##### Distribution.

**Figure 44.**
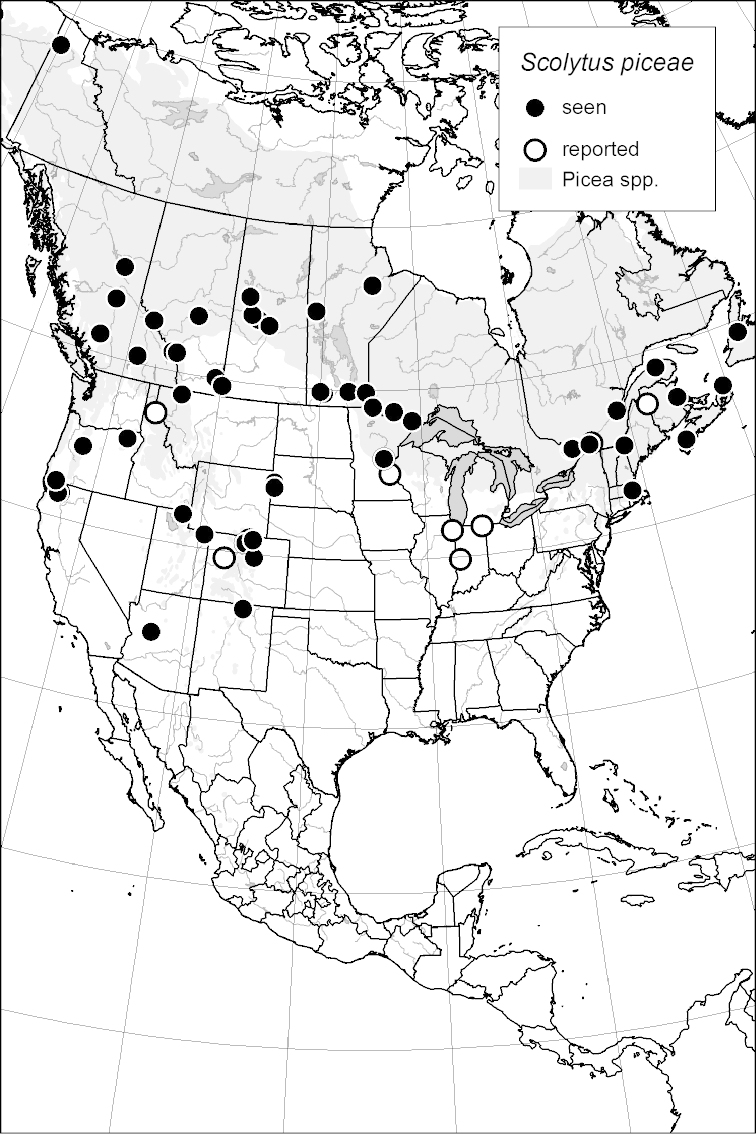
*Scolytus piceae* distribution map.

CANADA: Alberta, British Columbia, Manitoba, New Brunswick, Newfoundland, Northwest Territories, Nova Scotia, Ontario, Prince Edward island, Quebec, Saskatchewan, Yukon. UNITED STATES: Alaska, California, Colorado, Idaho, Maine, Massachusetts, Michigan, Minnesota, Montana, New Mexico, New York, North Dakota, Oregon, South Dakota, Utah, Washington, Wisconsin, Wyoming (Fig. 44).

##### Hosts.

Most spruce species including *Picea breweriana* S. Watson (Brewer spruce), *Picea engelmannii* Parry ex Engelm. (Engelmann spruce), *Picea glauca* (Moench) Voss (white spruce), *Picea mariana* (Mill.) B.S.P. (black spruce), *Picea pungens* Engelm. (blue spruce), and *Picea rubens* Sarg. (red spruce). This species rarely attacks *Larix* spp. (larch) and *Abies balsamea* (L.) Mill (balsam fir).

##### Biology.

*Scolytus piceae* infests dead and dying limbs (Chamberlin 1939; Bright and Stark 1973) and is commonly found in branches of fallen spruce and suppressed limbs of standing hosts (Smith, pers. obs.).

The adult gallery is parallel to the grain of the wood and bayonet shaped (Fig. 24). From the central nuptial chamber, one egg gallery extends to the grain of the wood and the other egg gallery is slightly transversely extended and then extends parallel to the grain. The nuptial chamber is oblique to the egg galleries. The adult gallery equally scores the sapwood and cambium but in some instances may only lightly score the sapwood. Adult galleries average 5.0–8.0 cm in length. Ten to 30 egg niches are widely spaced and deeply score the sapwood. Larvae extend their mines perpendicular to the egg gallery before diverging in a fan shaped pattern. Pupation may occur under the bark or in the sapwood (Edson 1967; Bright and Stark 1973). There is likely one generation per year (Bright and Stark 1973; Furniss and Johnson 2002).

##### Remarks.

The female paralectotype has been returned to CUIC from the CNCI following the repositories listed in Swaine (1910).

#### Scolytus praeceps

Taxon classificationAnimaliaColeopteraCurculionidae

LeConte, 1876

Figs 45 , 46 , 47

Scolytus praeceps LeConte, 1876: 373.
Scolytus abietis Blackman, 1934: 21. **syn. n.**
Scolytus opacus Blackman, 1934: 20. **syn. n.**

##### Diagnosis.

*Scolytus praeceps* is a morphologically variable species across its range. Both sexes most closely resemble *Scolytus obelus* and are differentiated by the absence of a median tubercle on the apical margin of ventrite 2 in both sexes. The female is differentiated from that of *Scolytus dentatus* by the presence of a weakly developed and almost indistinct epistomal process and smaller size.

##### Description (male).

1.8–3.2 mm long (mean = 2.5 mm; n = 20); 2.0–2.9 times as long as wide. Color dark red-brown to black, antennae yellow-brown. Pronotum typically darker than elytra.

*Head.* Epistoma weakly emarginate; epistomal process absent; median area above mandibles bearing dense patch of long, yellow, hair-like setae. Frons appearing convex when viewed laterally, slightly transversely impressed just above epistoma and along median line to upper level of eyes (may not be impressed in some specimens); moderately, coarsely, aciculate-punctate; aciculations converging at epistoma; punctures small, coarse; moderately, uniformly covered by long, fine, yellow, erect, hair-like setae, these longer than width of midpoint of eye. Antennal scape short, elongate; club flattened, ovoid, setose with partial septum, two arcuate sutures visible.

*Pronotum* wider than long; apical margin broadly rounded, median area between eyes lined with scales; sides distinctly arcuate, strongly constricted near apex, forming a weak transverse impression near apical margin; surface smooth, shining, punctures on disc fine, shallow, moderately abundant, larger and more abundant laterally and on apical constriction; apical and anterolateral margins bearing sparse, erect, yellow, hair-like setae; base weakly bisinuate.

*Elytra* with sides sub-parallel on apical half, narrowing to subquadrate, smooth apex; apex weakly emarginated at suture. Margin of apical edge bearing large, coarse punctures. Disc glabrous, smooth, shining; interstriae not impressed, more than twice width of striae, punctures uniseriate, smaller than those of striae; striae not impressed. Declivity bearing sparse, short, erect yellow setae. Metepimeron half-length of metanepisternum.

*Venter.* Apical margin of ventrite 1 strongly, acutely produced forming lip along base of ventrite 2, basal margin of ventrite 2 appearing impressed. Ventrite 2 nearly perpendicular to ventrite 1; surface glabrous, shagreened, finely punctate (obscurely punctate in some specimens); punctures small, fine, shallow; surface weakly concave; apical margin unarmed or armed with a longitudinal carina and blunt tubercle, appearing keel-shaped or a low median longitudinal carina; lateral margins of ventrites 2–3 and ventrite 4 unarmed. Ventrite 5 carinate ridge closer to apical margin of segment; length of ventrite 5 equal to combined lengths of ventrites 3 and 4; setal patch and median depression absent.

**Figure 45.**
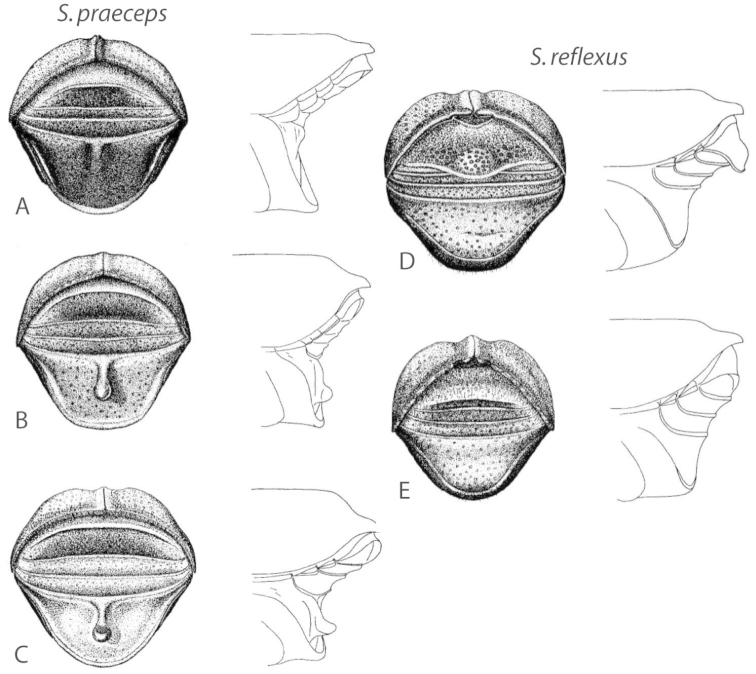
Male venters. **A** *Scolytus praeceps* **B** *Scolytus praeceps* abietis phenotype **C** *Scolytus praeceps* opacus phenotype **D** *Scolytus reflexus* **E** *Scolytus reflexus wickhami* phenotype (from Edson 1967).

**Figure 46.**
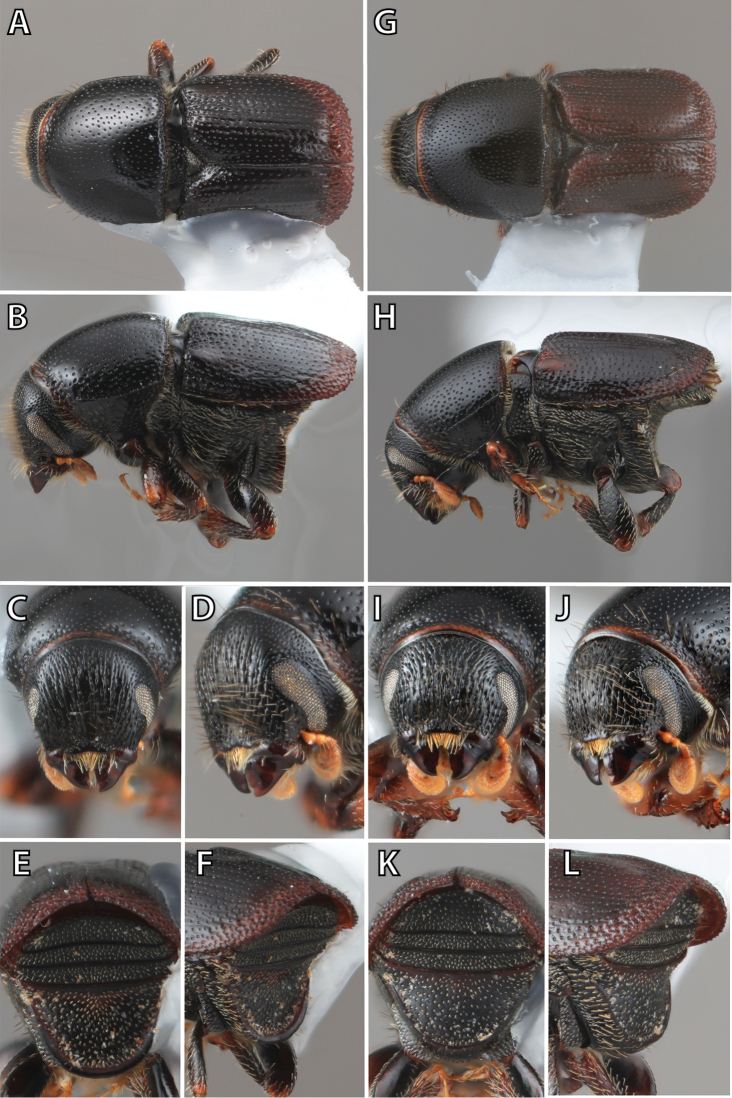
*Scolytus praeceps* **A** dorsal male habitus **B** lateral male habitus **C** male frons **D** male frons oblique **E** male venter **F** male venter oblique **G** dorsal female habitus **H** lateral female habitus **I** female frons **J** female frons oblique **K** female venter **L** female venter oblique.

**Figure 47.**
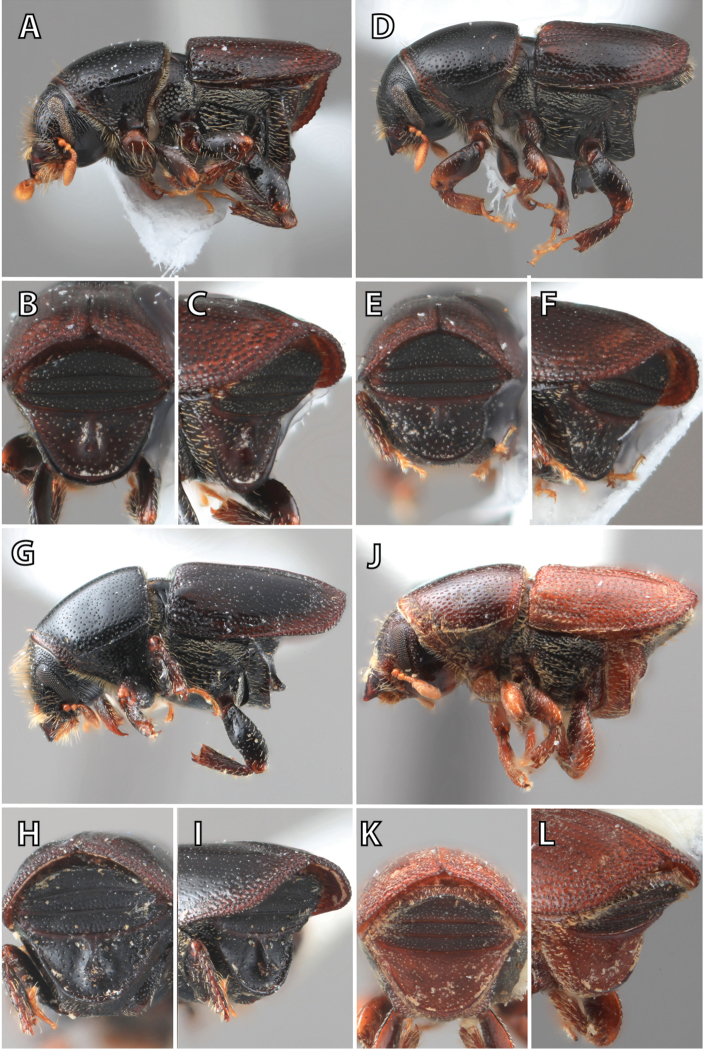
*Scolytus praeceps* abietis phenotype **A** lateral male habitus **B** male venter **C** male venter oblique **D** lateral female habitus **E** female venter **F** female venter oblique **G** lateral opacus phenotype male habitus **H** opacus phenotype male venter **I** opacus phenotype male venter oblique; opacus phenotype female **J** opacus phenotype lateral female habitus **K** opacus phenotype female venter **L** opacus phenotype female venter oblique.

##### Female.

1.7–3.5 mm long (mean = 2.69 mm; n = 20); 2.0–3.0 times as long as wide. Similar to male except epistoma feebly emarginate, epistomal process absent, frons more strongly convex when viewed laterally, weakly aciculate, setae sparser, shorter, less than width of eye; entirely convex to weakly transversely impressed just above epistoma. Apical margin of ventrite 1 weakly elevated above base of ventrite 2. Apical margin of ventrite 2 unarmed or weakly longitudinally tumescent.

##### Specimens examined.

627.

##### Type material.

Lectotype *Scolytus praeceps* LeConte: female, labeled “Cali. [California, Calaveras, Crotch Coll], Type 967” (MCZC). Lectotype designated Wood 1982: 439. Holotype *Scolytus abietis* Blackman: male, labeled “8637iss [Sandpoint, Idaho], 6-20-[18]99, [A.D. Hopkins], Hopk. US 224, Type No. 43837” (USNM). Holotype *Scolytus opacus* Blackman: male, labeled “Ouray, Colo[rado], 7,500-8,000 ft., July 1-15, [18]97, HF Wickham, Type No. 43836” (USNM).

##### Non-type material.

**CANADA:** ***BRITISH COLUMBIA*:** [Big Creek Provincial Park], Lorna [Lake], 5.VIII.1926, H. Richmond, ex. *Abies lasiocarpa* (CASC-2). Duncan, Genoa Bay, 16.VIII.1928, W.G. Mathers, ex. *Abies grandis* (CNCI-10), 17.VIII.1928 (CASC-3). **UNITED STATES:** ***CALIFORNIA*:** *Alpine Co.*: Humboldt-Toiyabe National Forest, Hwy 88, N38°45.824', W119°51.498', 6262 ft, 24.VII.2010, S.M. Smith, ex. *Abies concolor* (MSUC-3). *El Dorado Co.*: Georgetown, 10 mi E., University of California Blodgett [Experimental] Forest, VI.[19]62, R.W. Stark, ex. *Abies concolor* (DEBC-3, EMEC-8); 30.V.1986, K. Hobson (EMEC-2), 1-2.VI.1986 (EMEC-2), 21-24.VI.1986 (EMEC-2), 1-7.VII.1986 (EMEC-9), 9-16.VII.1986 (EMEC-3); 2.VI.2003, K. Apigian (EMEC-2), 5.VI.2003 (EMEC-1). Ice House Reservoir, 25.V.2007, A.I. Cognato (MSUC-5). [Lake Tahoe], Fallen Leaf Lake, 6300 ft, 5.VII.1940, H.T. Reynolds (EMEC-2). Pacific House, 0.7 mi E., 2.VII.1989, F.G. Andrews, ex. flume (CSCA-1). Pacific House, 1 mi E., 1.V.1994, C.B. Barr, ex. seining El Dorado ditch (EMEC-1). South Lake Tahoe, 16.VI.1988, D. Adams, ex. reared from *Abies concolor* (CSCA-1). [*Fresno Co.*]: Huntington Lake, 23.VII.[19]19, F.C. Clark (CASC-52). Shaver [Lake], 14.IX.[19]08, Miller, ex. *Abies concolor* (CNCI-3). [*Madera Co.*]: North Fork, 22.VI.1935, R.P. Allen (EMEC-1). *Marin Co.*: Inverness, 8.VIII.[19]62, C.A. Toschi (EMEC-1). *Mariposa Co.*: Yosemite Valley, 7.VII.1921 (CASC-21). *Mendocino Co.*: Noyo River, VI.1896 (CASC-2). *Modoc Co.*: Alturas, Knox Mountain, 8.VII.1964, D.L. Dahlsten (EMEC-1). Warner Mountains, 4.VII.1919, G. Hopping, ex. *Abies concolor* (CASC-4, USNM-2). *Monterey Co.*: 30.VII.1964, D.E. Bright, ex. *Abies bracteata* (CASC-8). Carmel Valley, 15 mi S., 22.VI.1963, C.J. Wray, ex. *Abies bracteata* (CNCI-7, DEBC-1, EMEC-5). Williams Canyon, Los Padres National Forest, 24.IV.1992, D.E. Bright, G. Ferrell, ex. *Abies bracteata* limbs (CNCI-22). *Nevada Co.*: Donner Memorial State Park, 10.IX.[19]87, S. Seybold, ex. collected on *Abies concolor* (CNCI-2, EMEC-1), 11.IX.[19]87 (CNCI-1, EMEC-1), 29.X.1987 (CNCI-2, EMEC-2). Nevada City, 28.V.1939, R.P. Allen (CNCI-5). *Riverside Co.*: Mount San Jacinto State Park, 33.807°N, 116.654°W, 15.VII.2003, M. Caterino (SBMN-1). Santa Rosa Mountain, 15.IX.[19]56, D.E. Bright, D.N. King, ex. *Abies concolor* (EMEC-4). *San Bernardino Co.*: San Bernardino National Forest, E. of Arrowbear Lake, 34.2076°N, 117.0584°W, 29.V.2004, M. Caterino (SBMN-2); S. Fork Trail, 34.1297°N, 117.8426°W, 28.V.2004, M. Caterino (SBMN-1). San Bernardino Mountains, Dollar Lake trail, 10.VII.1956, R.W. Bushing (EMEC-2), 11.VII.1956 (DEBC-1, EMEC-10). *Sierra Co.*: Calpine, 2.3 mi N.W., 5200ft, 27.VIII.1961, H.B. Leech, ex. under bark of *Abies* sp. (CASC-9). *Siskiyou Co.*: McCloud, 14.VI.1961, S.L. Wood, J.B. Karren, D.E. Bright, ex. *Abies concolor* (DEBC-5). Mount Shasta, 28.VII.1980, A.J. Gilbert (CASC-1). *Trinity Co.*: Klamath National Forest, FR 41N16, 0.2 mi E. FR 93, N41°14.822', W122°53.562', 5081ft, 28.VII.2010, S.M. Smith, ex. *Abies concolor* (MSUC-2). *Tuolumne Co.*: Cow Creek, 5 mi N. Strawberry, 18.VII.1964, C.W. O’Brien, ex. *Abies* sp. (CASC-102, DEBC-11). Pinecrest, Hopk. U.S. 19192-A, 29.VII.1930, G.R. Struble, ex. *Abies concolor* (DEBC-3); IX.1966, emerged 28.III.1967, G.T. Ferrell, ex. *Abies concolor* (EMEC-4). Sierra Village, 10.VII.1966, B.A. Tolden (EMEC-1). [*Unspecified County*]: Shasta [-Trinity] National Forest, Hopk. U.S. 21078, 10.V.1934, K.A. Salmon (DEBC-4). ***COLORADO*:** *Clear Creek Co.*: Idaho Springs, 12 mi S., 12.VII.2007, D.E. Bright, B.A. Barr, ex. bole *Abies lasiocarpa* (CSUC-2, DEBC-4). *Eagle Co.*: Basalt Mountain, 20.VI.1996, D. Leatherman (CSUC-1). ***IDAHO*:** [*Bonner Co.*]: Sandpoint, 2.VII.1964, N.M. Downie (FMNH-1). *Clearwater Co.*: Angel Butte thinning, T37N R3E, sec 3, ex. *Abies grandis* #86 (USNM-10). *Kootenai Co.*: Coeur d’Alene, 28.IV.1922, H.J. Rust, ex. *Abies grandis* (MSUC-3); 15.V.1951, 2157 ft, W.F. Barr, ex. *Abies grandis* (EMEC-3). *Latah Co.*: Moscow Mountain, 46.8042, -116.830183, 2713ft, 10.VIII.2010, S.M. Smith, [A.R. Gillogly], M.M. Furniss, ex. *Abies lasiocarpa*, emerged II.2011, M.M. Furniss (MSUC-72). *Lewis Co.*: Nezperce, 3 mi N., 3.VII.[19]69, R.C. Biggum (EMEC-1). ***OREGON*:** *Deschutes Co.*: Deschutes National Forest, Black Butte Rd, Black Butte, N44°24.924', W121°38.323', 4212 ft, 31.VII.2010, S.M. Smith, ex. *Abies grandis* (MSUC-91). Paulina Lake, 2 mi W., 12.VI.1940, Schuh, Scott, ex. *Abies concolor* (EMEC-2). *Douglas Co.*: Diamond Lake, 9.VII.[19]64, D.E. Bright, ex. *Abies* sp. (EMEC-7). *Jackson Co.*: Copper, 2 mi N., 3.IX.1970, W.G. Harwood, ex. *Abies lasiocarpa*, emerged 21.X.1970 (MSUC-7). [*Klamath Co.*]: Keno, 16.VII.[19]29, J.A. Beal, ex. *Abies concolor* (CSUC-2). *Linn/Lane Co.*: Blue River, 11 mi N.E., H.J. Andrews Experimental Forest, 5.VIII.1988, log decomp study, site 2, SE1/4 S15 T15S RSE, trap 2WA (OSAC-1). [*Umatilla Co.*]: Tollgate, 6.VI.[19]49, C. Chastain, ex. *Abies grandis* (CSUC-3, EMEC-2, FMNH-1). ***UTAH*:** *Utah Co.*: Hobble Creek Canyon, 14.VI.[19]60, D.E. Bright, ex. *Abies concolor* (DEBC-2). [*Unspecified County*]: (CNCI-1). 1952, T.O. Thatcher (CSUC-10). ***WYOMING*:** *Albany Co.*: Snowy Mountains, [Medicine Bow National Forest], Spruce campground, 6.IX.2010, D.E. Bright, B.A. Barr coll., ex. *Abies concolor* branches (DEBC-2). [*Carbon Co.*]: Saratoga, Hopk. U.S. 31518-E, 1.IX.[19]38, ex. *Abies lasiocarpa* (CSUC-1).

##### Distribution.

**Figure 48.**
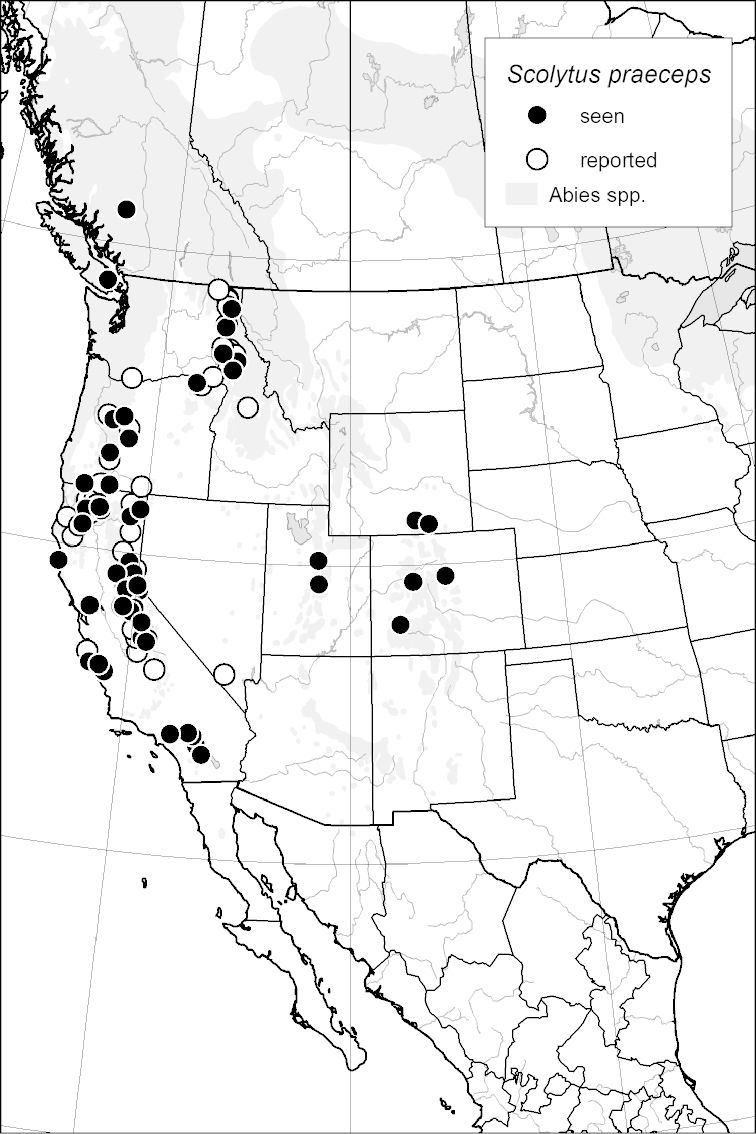
*Scolytus praeceps* distribution map.

CANADA: Alberta, British Columbia. UNITED STATES: California, Colorado, Idaho, Montana, Oregon, Utah, Washington, Wyoming (Fig. 48).

##### Hosts.

*Abies concolor* (Gord. & Glend.) Lindl. ex Hildebr. (white fir), *Abies grandis* (Douglas ex D. Don) Lindl. (grand fir), *Abies lasiocarpa* (Hook.) Nutt. (subalpine fir) and *Abies bracteata* (D. Don) Poit. (bristlecone fir).

##### Biology.

*Scolytus praeceps* prefers to colonize fresh slash but is also found in small limbs and tops of small standing trees (Edson 1967; Bright and Stark 1973).

The adult gallery contains two egg galleries that branch from the central nuptial chamber. Typically, one egg gallery extends from the nuptial chamber perpendicular to the grain of wood and the second egg gallery extends at a 45° angle to the grain (Fig. 24). Occasionally both egg galleries are extended obliquely to the grain, at a 45° angle to the grain or perpendicular to the grain (Edson 1967). Galleries are frequently initiated near branch crotches and disguised under rough patches of bark. The adult gallery equally scores the sapwood and cambium. The adult galleries range in size from 2.5–6.4 cm in length. Eggs are laid in niches on both sides of each egg gallery and larval mines radiate perpendicular to the egg gallery. The larval mines gradually diverge forming a fan shaped pattern and lightly etch the sapwood. Pupation occurs in the sapwood (Edson 1967). There is one generation per year and broods overwinter as larvae (Furniss and Johnson 2002).

##### Remarks.

There has been a great deal of historical uncertainty regarding the placement of *Scolytus abietis* and *Scolytus opacus* since they were described. The species are extremely morphologically similar and thus difficult to differentiate. This similarity led several authors to treat *Scolytus abietis* as either a synonym of *Scolytus opacus* (Wood 1982) or as a subspecies (Bright 1976). Equihua-Martínez and Furniss (2009) removed *Scolytus abietis* from synonymy with *Scolytus opacus* based on differences in host use, submentum shape, and striations on the underside of the head and characters of the second ventrite including setae, punctures and spine shape. The species *Scolytus abietis*, *Scolytus opacus* and *Scolytus praeceps* were not recovered as monophyletic in any of our analyses (Figs 2, 8, 9). We observed very low intraspecific COI and ArgK nucleotide differences among populations (COI: 0–0.0049; mean = 0.0032), (ArgK: 0–0.0044; mean = 0.0024) and no differences in 28S or CAD (Table 6). Considerable variation was also observed in the shape of the spine on the male second ventrite, especially within *Scolytus praeceps*. *Scolytus abietis* and *Scolytus opacus* are here placed in synonymy with *Scolytus praeceps* because they were recovered as polyphyletic (Figs 2, 8, 9), there are minute molecular difference among populations sampled from California, Oregon, Idaho and Wyoming (Table 6), the species have identical galleries (Edson 1967, Wood 1982) and variability of the spine on male ventrite 2.

The lectotype of *Scolytus praeceps* bears a partial locality label. LeConte’s (1868) description states that the lectotype was collected at Calaveras, California by Mr. Crotch.

#### Scolytus reflexus

Taxon classificationAnimaliaColeopteraCurculionidae

Blackman, 1934

Figs 45 , 49 –50

Scolytus reflexus Blackman, 1934: 13.
Scolytus virgatus Bright, 1972: 1490. **syn. n.**
Scolytus wickhami Blackman, 1934: 13. **syn. n.**

##### Diagnosis.

Males exhibiting the reflexus phenotype are readily distinguished by the presence of a strong recurved subapical carinate ridge on ventrite 5 that is medially produced to form a slightly recurved and subrostriform ridge that appears “reflexed”. Males of this species exhibiting the wickhami phenotype are easily confused with males of *Scolytus monticolae*. They are easily distinguished by the size of male ventrite 5. In *Scolytus reflexus*, ventrite 5 is equal in length to ventrite 4. In *Scolytus monticolae*, ventrite 5 is equal in length to ventrites 3 and 4 combined. *Scolytus monticolae* lacks an epistomal process while *Scolytus reflexus* typically has a strongly developed epistomal process. Females are distinguished from those of *Scolytus monticolae* by having the apical margin of ventrite 1 rounded and by the ventrite 2 surface rugose, shining, coarsely punctate and convex.

##### Description (male).

2.4–3.8 mm long (mean = 3.2 mm; n = 20); 1.8–2.5 times as long as wide. Color dark red-brown to black, antenna brown. Pronotum typically darker than elytra.

*Head.* Epistoma weakly to acutely, deeply emarginate; epistomal process weakly to strongly developed and elevated; median area above mandibles bearing dense patch of long, yellow, hair-like setae. Frons appearing flattened when viewed laterally, slightly transversely impressed just above epistoma; moderately, longitudinally aciculate, deeply, coarsely punctate; aciculations converging at epistoma; punctures small, coarse; moderately, uniformly covered by long, fine, yellow, erect, hair-like setae, these longer than width of midpoint of eye. Antennal scape short, elongate; club flattened, irregularly ovoid, setose with partial septum, three sharply arcuate sutures visible.

*Pronotum* wider than long; apical margin broadly rounded, median area between eyes lined with scales; sides distinctly arcuate, strongly constricted near apex, forming a weak transverse impression near apical margin; surface smooth, shining, punctures on disc fine, shallow, moderately abundant, larger and more abundant laterally and on apical constriction; apical, anterolateral and lateral margins bearing sparse, erect, yellow, hair-like setae; base weakly bisinuate.

*Elytra* with sides sub-parallel on apical half, narrowing to subquadrate, smooth apex; apex moderately emarginated at suture. Margin of apical edge bearing small, fine punctures. Disc smooth, shining; interstriae weakly impressed, more than twice width of striae, punctures uniseriate, smaller than those of striae, bearing minute, recumbent setae less than length of a puncture; striae weakly impressed. Declivity bearing sparse, short, erect yellow setae. Metepimeron half-length of metanepisternum.

*Venter.* Apical margin of ventrite 1 rounded, marked by weak carina on vertical surface of segment. Ventrite 2 nearly perpendicular to ventrite 1; surface rugose, shagreened, finely punctate; punctures small, coarse, shallow; surface convex; setae moderately abundant, long, erect and longer than length of ventrite 3; lateral margins of ventrites 2–3 and ventrite 4 unarmed. Ventrite 5 typically armed with strong, recurved, subapical, carinate ridge, occasionally modified and medially produced to form a slightly recurved and subrostriform ridge appearing “reflexed” (Chiricahua Mountains, Arizona and Chihuahua, Mexico populations). Ventrite 5 carinate ridge closer to apical margin of segment; length of ventrite 5 less than combined lengths of ventrites 3 and 4; setal patch and median depression absent.

**Figure 49.**
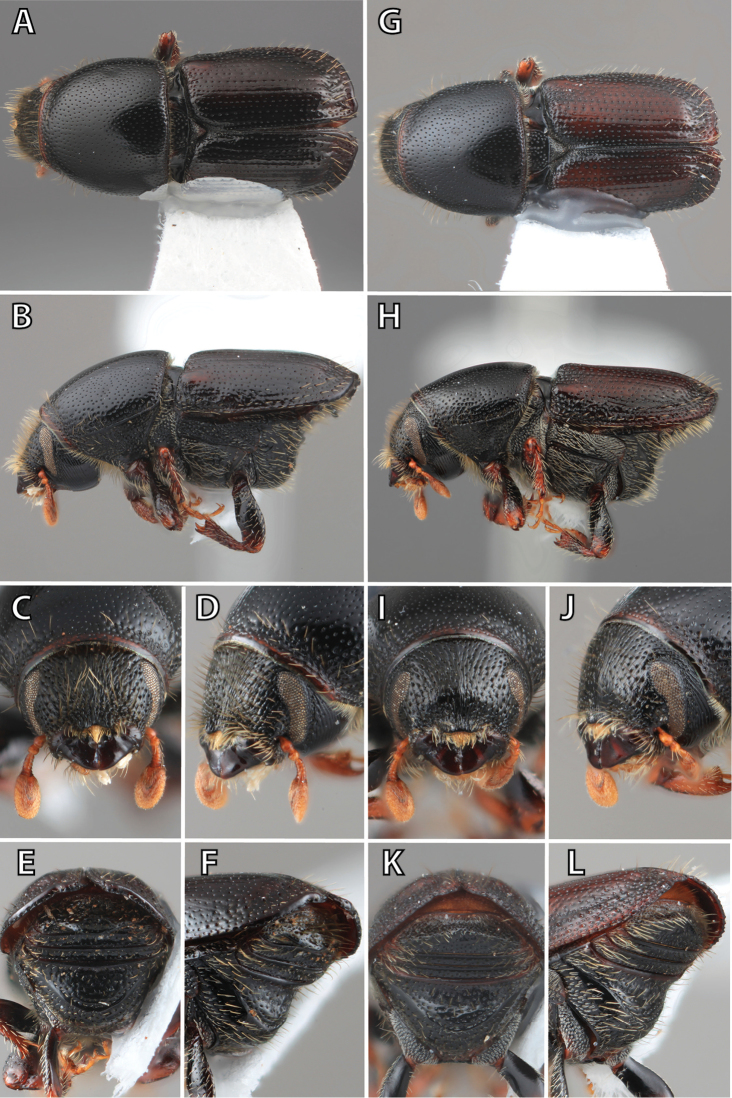
*Scolytus reflexus* **A** dorsal male habitus **B** lateral male habitus **C** male frons **D** male frons oblique **E** male venter **F** male venter oblique **G** dorsal female habitus **H** lateral female habitus **I** female frons **J** female frons oblique **K** female venter **L** female venter oblique.

**Figure 50.**
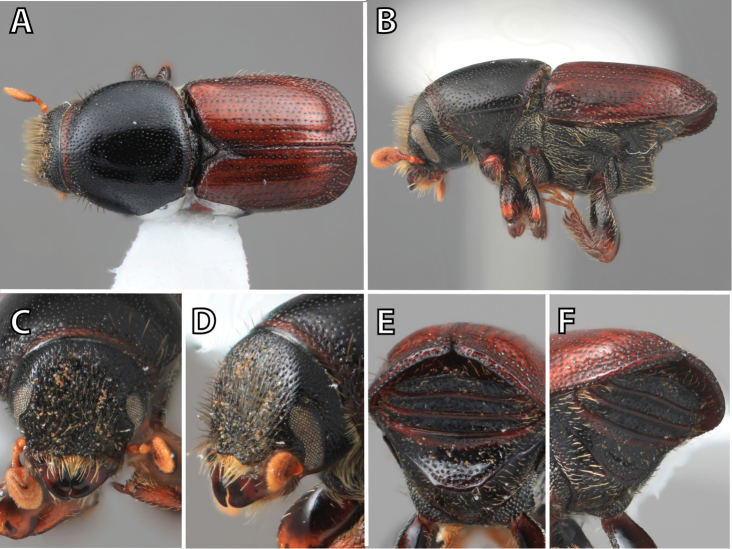
*Scolytus reflexus* wickhami phenotype **A** dorsal male habitus **B** lateral male habitus **C** male frons **D** male frons oblique **E** male venter **F** male venter oblique.

##### Female.

2.5–4.0 mm long (mean = 3.2 mm; n = 20); 1.87–2.5 times as long as wide. Similar to male except epistoma entire, epistomal process absent, frons convex when viewed laterally, weakly longitudinally aciculate, setae sparser, shorter, less than width of eye; weakly transversely impressed just above epistoma and between inner apices of eyes. Second ventrite unarmed, setae sparse, erect, measuring length of three diameters of a puncture. Length of ventrite 5 greater than combined lengths of ventrites 3 and 4, armed with apical strongly recurved subapical carinate ridge.

##### Specimens examined.

358.

##### Type material.

Holotype *Scolytus reflexus* Blackman: male, labeled “Sta Catalina Mts, Ariz., Chrisman, M. Coll, *Pseudotsuga taxifolia*, Hopk. U.S. 12210, Reared June 1-14 H.B. Kirk, Type No. 43831 USNM” (USNM). Holotype *Scolytus virgatus* Bright: male, labeled “MEX., N.L., Cerro Potosi, V.2.71, 8500', D.E. Bright, *Pseudotsuga menziesii*, CNC No. 12604” (CNCI). Holotype *Scolytus wickhami* Blackman: male, labeled “Buena Vista, Col. H.F. Wickham July 1-6 ’96, 7,900-8,000 ft, Type No. 43832 USNM” (USNM). Paratypes *Scolytus reflexus* **UNITED STATES:** ***ARIZONA*:** [*Cochise Co.*]: Chiricahua Mountains, 18.VI.[?], Hubbard, Schwarz (EMEC-1). *Pima/ Pinal Co.*: Santa Catalina Mountains, M. Chrisman, reared 25.VI.[19]14, H.B. Kirk, ex. *Pseudotsuga taxifolia* [= *Pseudotsuga menziesii*] (EMEC-1). Paratypes *Scolytus wickhami* **UNITED STATES:** ***ARIZONA*:** [*Cochise Co.*]: Chiricahua Mountains, Hopk. U.S. 5558-A, 5.VIII.[19]07, J.L. Webb, ex. *Pseudotsuga taxifolia* [= *Pseudotsuga menziesii*] (CNCI-1). ***NEW MEXICO*:** [*Lincoln Co.*]: Capitan Mountains, Hopk. U.S. 5674, J.L. Webb, ex. *Pseudotsuga taxifolia* [= *Pseudotsuga menziesii*] (CNCI-1). Paratypes *Scolytus virgatus* **MEXICO:** ***Nuevo León*:** Cerro Potosi, 8500 ft, 2.V.[19]71, D.E. Bright, ex. *Pseudotsuga menziesii* (CNCI-4).

##### Non-type material.

**MEXICO:** ***CHIHUAHUA*:** San Juanito, 16 km N.E., 50 m, 19.VII.1960, S.L. Wood, ex. *Pseudotsuga menziesii* (MSUC-3, USNM-7). San Juanito, Hopk. U.S. 58592, 16.III.1974, M.M. Furniss, ex. *Pinus* sp. (USNM-1, WFBM-5). ***DURANGO*:** Durango, Hopk. U.S. 58685, 24.III.1974, M.M. Furniss, ex. *Pseudotsuga menziesii* (USNM-14). **UNITED STATES:** ***ARIZONA*:** *Cochise Co.*: Chiricahua Mountains, 21.VII.1916, C.R. Bruck, (DEBC-2), 29.IX.[19]47, D.J. & J.N. Knull (DEBC-4). Coronado National Forest, Chiricahua Mountains, N31°54.915', W109°16.040', 8196 ft, 20.V.2010, S.M. Smith, ex. *Pseudotsuga menziesii* (MSUC-7). Huachuca Mountains, Upper Carr Canyon, 7500 ft, 6–10.VIII.[19]52, H.B. Leech, J.W. Green (CASC-1). [*Coconino Co.*]: Jacob Lake, Kaibab National Forest, 19.VI.[19]66, [L. Edson], ex. *Pseudotsuga menziesii* (EMEC-3). *Greenlee Co.*: Hannagan [Meadows] campground, 12.VII.1968, D.E. Bright, ex. *Pseudotsuga menziesii* (CNCI-5). *Pima Co.*: Tucson, Mount Lemmon, 11.VI.1969, S.L. Wood, ex. *Pseudotsuga menziesii* (MSUC-1). *Pima/Pinal Co.*: Santa Catalina Mountains, 9000 ft, 6.VI.1926, R.B. Streets (CASC-1); 5.VIII.1968, D.E. Bright, ex. *Pseudotsuga menziesii* (CNCI-1); Bear Wallow, 7800 ft, 11.VI.1969, S.L. Wood (USNM-5). *Santa Cruz Co.*: Carr Canyon, 8.VIII.1962, S.L. Wood, ex. *Pseudotsuga taxifolia* [= *Pseudotsuga menziesii*] (USNM-12). *Yavapai Co.*: Prescott National Forest, Mount Union, Lake. *Pseudotsuga menziesii* (EMEC-1). ***COLORADO*:** [*Boulder Co.*]: Boulder, Hopk. U.S. 17700-Y, 21.IV.[19]37, J.A. Beal, ex. *Pseudotsuga taxifolia* [= *Pseudotsuga menziesii*] (CSUC-3, USNM-5). *Chaffee Co.*: N. of Poncha Pass, 25.VII.1997, D. Leatherman, ex. ponderosa pine [= *Pinus ponderosa*] (CSUC-1). *Clear Creek Co.*: Idaho Springs, 7.5 mi S., 12.VII.2007, D. Bright, B.A. Barr, ex. bole *Pseudotsuga menziesii* (DEBC-3). Lawson, 26.VII.1994, D. Leatherman, ex. Douglas fir [= *Pseudotsuga menziesii*] (CSUC-4). Near Lawson, 26.VII.1994, S. Kelley, ex. *Pseudotsuga menziesii* (CNCI-2). [*Garfield Co.*]: Glenwood Springs, VII.[?] (CASC-1). *Jefferson Co.*: 1–15.VII.2010, Colorado Dept of Agriculture, ex. Lindgren funnel UHR EtOH & Ω-pinene conifer (CSUC-3). Buffalo Creek, 6.VIII.2004, D. Leatherman, ex. Douglas fir [= *Pseudotsuga menziesii*] (CSUC-1). *Larimer Co.*: Estes Park, 19.VI.[19]35, ex. *Pseudotsuga taxifolia* [= *Pseudotsuga menziesii*] (USNM-3); 28-VI-13.VII.2010, Colorado Dept of Agriculture, ex. Lindgren funnel UHR EtOH & Ω-pinene conifer (CSUC-3). Fort Collins, 29.VI.[19]35 (USNM-2). Mount Margaret trailhead, 4.VIII.1994, D. Leatherman, ex. Douglas fir [= *Pseudotsuga menziesii*] (CSUC-1). Pingree Park, 30.VIII.1995, D. Leatherman (CSUC-1). Poudre Canyon, 27.V.1975, D. Leatherman, ex. Douglas fir [= *Pseudotsuga menziesii*] (CSUC-1). Rist Canyon, 28.X.[19]56, D.E. Bright, ex. *Pseudotsuga taxifolia* [= *Pseudotsuga menziesii*] (DEBC-1); S28 T8 NR70W, 3.III.1957, T.O. Thatcher, ex. *Pseudotsuga taxifolia* [= *Pseudotsuga menziesii*] (DEBC-7). Rist Canyon Picnic Area, 26.VI.2008, D. Leatherman, ex. fallen Douglas fir [= *Pseudotsuga menziesii*] (CSUC-3). Roosevelt National Forest, Big Thompson Canyon, N40°24.456', W105°24.565', 7080 ft, 5.V.2010, S.M. Smith, D.E. Bright, B.A Barr, ex. *Pseudotsuga menziesii* (MSUC-2). Red Feather Lakes, 12.III.2003, D. Leatherman, ex. Douglas fir [= *Pseudotsuga menziesii*] (CSUC-1), VI.2000 (CSUC-5). CR 63E, 2 mi S. off Hwy 14, 4.VII.2007, D. Leatherman, ex. Douglas fir [= *Pseudotsuga menziesii*] (CSUC-11). *Mineral Co.*: Wolf Creek Pass off US 160, Sheep Mountain, 25.VII.2005, D. Leatherman, ex. Douglas fir [= *Pseudotsuga menziesii*] (CSUC-1). *Pueblo Co.*: SR 165, 5 mi S.E. San Isabel Millset trailhead, 23.VI.2000, D. Leatherman, ex. Douglas fir [= *Pseudotsuga menziesii*] (CSUC-3). *Teller Co.*: Ridgewood Subdivision, 5.VIII.2004, D. Leatherman, ex. Douglas fir [= *Pseudotsuga menziesii*] (CSUC-1). ***NEVADA*:** [*Lander Co.*]: Austin, 12.VIII.[19]40, D.E. Hardy (USNM-1). ***NEW MEXICO*:** *Otero Co.*: Cloudcroft, 4.VI.1969, tree 53, S.L. Wood, ex. *Pseudotsuga menziesii* (MSUC-14); 11-13.VII.[19]74, D.E. Bright, ex. *Pseudotsuga menziesii* (CNCI-4). Lincoln National Forest, Apache Point Observatory, N32°47.046', W105°48.841', 9116 ft, 15.V.2010, S.M. Smith, ex. *Pseudotsuga menziesii* (MSUC-145). [*Sandoval Co.*]: Jemez Springs, Hopk. U.S. 37218-F, ex. *Pseudotsuga menziesii* (USNM-4); Hopk. U.S. 37214-D, 1.IX.[19]57, F.M. Yasinski, ex. *Pseudotsuga taxifolia* [= *Pseudotsuga menziesii*] (USNM-15). ***TEXAS*:** *Culberson Co.*: Guadalupe Mountains National Park, The Bowl, 17.VII.1974, Bright, ex. *Pseudotsuga menziesii* (CNCI-6). ***UTAH*:** [*Utah Co.*]: Wasatch National Forest, Mount Timpanogos, 13.VII.1957, D.E. Bright, ex. *Abies concolor* (DEBC-1). [*Unspecified County*]: Logan Canyon, 5000 ft, 31.XII.1945, S.L. Wood, ex. Douglas fir [= *Pseudotsuga menziesii*] (USNM-3), 28.IV.1946 (USNM-2), 16.VI.1946 (USNM-4), 3.VII.1946 (USNM-4).

##### Distribution.

**Figure 51.**
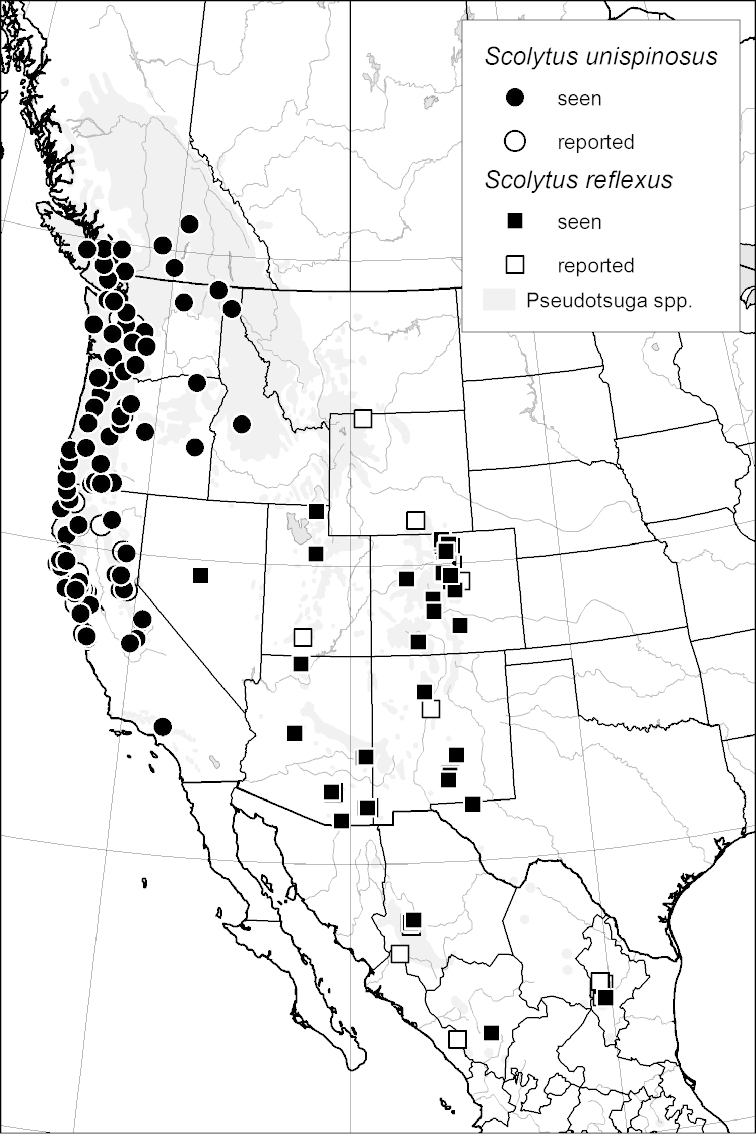
*Scolytus reflexus* and *Scolytus unispinosus* distribution map.

MEXICO: Chihuahua, Durango, Nuevo León. UNITED STATES: Arizona, Colorado, Nevada, New Mexico, Texas, Utah (Fig. 51).

##### Hosts.

*Pseudotsuga menziesii* (Mirb.) Franco (= *Pseudotsuga taxifolia* Britton) (Douglas fir).

##### Biology.

*Scolytus reflexus* is relatively common throughout its range and is found in fresh slash and branches of Douglas fir (Edson 1967).

The adult gallery consists of two egg galleries and a central nuptial chamber. The adult gallery is parallel to the grain of the wood and bayonet shaped (Fig. 24). From the central nuptial chamber, one egg gallery extends parallel to the grain of the wood and the other egg gallery is slightly transversely extended and then extends parallel to the grain. The nuptial chamber is oblique to the egg galleries. The adult gallery equally scores the sapwood and cambium and averages 3.9–7.6 cm in length. Egg niches are closely spaced and score the sapwood. Larvae extend their mines perpendicular to the egg gallery before diverging in a fan shaped pattern. Pupation may occur under the bark or in the sapwood (Edson 1967).

##### Collection notes.

The senior author has collected *Scolytus reflexus* in the Chiricahua Mountains, Arizona and Apache Point Observatory, New Mexico killing small (less than 10.0 cm DBH) Douglas fir trees. This species was also reported killing Douglas fir in the Sacramento Mountains and Santa Fe National Forest of New Mexico, and was tentatively and incorrectly identified as *Scolytus monticolae* (USFS 2004). *Scolytus monticolae* strongly resembles *Scolytus reflexus* (see diagnosis) but does not occur in New Mexico.

##### Remarks.

Wood (1977: 388) placed *Scolytus reflexus* and *Scolytus wickhami* in synonymy with *Scolytus tsugae*. Wood (1982) removed *Scolytus reflexus* from synonymy with *Scolytus tsugae*. In this publication he also listed *Scolytus wickhami* as a synonym of *Scolytus reflexus* and cited Wood (1977) for the synonymy. Wood and Bright (1992: 364) also cite Wood (1977) for the synonymy. However, *Scolytus wickhami* was never removed from synonymy with *Scolytus tsugae* and designated as a synonym of *Scolytus reflexus*.

In his description of *Scolytus virgatus*, Bright (1972) posited that the species was closely related to *Scolytus wickhami* or either a subspecies or variety. Wood (1982) considered *Scolytus wickhami* a synonym of *Scolytus reflexus*, but as discussed above, never formally placed it in synonymy. All three species are here treated as one slightly morphologically variable species. We assessed intraspecific variation within these three species for four genes (Table 6). It was small for each gene with the average divergence of 1.3% for COI and less than 0.12%, 0.03% and 0.29% for 28S, CAD and ArgK respectively among sampled populations (Table 6). These ranges are consistent with variation observed within other species. There are two main characteristics found that vary among the populations: the male fifth ventrite carina and the male epistomal process. There are two main phenotypes observed in populations: reflexus and wickhami. The reflexus phenotype includes individuals that were previously considered to be *Scolytus reflexus* with the male ventrite 5 armed with a strong recurved subapical carinate ridge that at its crest is closer to the basal than the apical margin of the segment. The length and height of the subapical carinate ridge is also variable within populations. This character is only found in the Chiricahua Mountains, Arizona and in Mexico. The wickhami phenotype includes individuals that were formerly considered *Scolytus wickhami* and *Scolytus virgatus*. In the wickhami phenotype the process on the male fifth ventrite is reduced in the wickhami phenotype and but still forms strong recurved subapical carinate ridge. However it is important to note that both the reflexus and wickhami phenotypes are sympatric in the Chiricahua Mountains. The male epistomal process also varies from a strongly developed and elevated ridge above the epistoma to weakly developed and elevated ridge. In general, the ridge is more strongly developed in the reflexus phenotype but considerable variation is observed especially in the wickhami phenotype. Additional variation is observed in the density of punctures on the male second ventrite and the coarseness of the male frons aciculations. In addition, the gallery structure of *Scolytus reflexus* and *Scolytus wickhami* are identical; the gallery of *Scolytus virgatus* is has not been noted. *Scolytus wickhami* and *Scolytus virgatus* are here designated as synonyms of *Scolytus reflexus*.

#### Scolytus robustus

Taxon classificationAnimaliaColeopteraCurculionidae

Blackman, 1934

Figs 52 –53

Scolytus robustus Blackman, 1934: 19.

##### Diagnosis.

The *Scolytus robustus* male is quite distinctive with its strongly flattened frons, apical margin of ventrite 1 strongly, acutely produced forming a lip along the base of ventrite 2, basal margin of ventrite 2 appearing impressed and by the unarmed apical margin of ventrite 2. The female is morphologically similar to that of *Scolytus ventralis*. The *Scolytus robustus* female is distinguished by having the apical margin of ventrite 1 forming a carinate lip along the basal margin of ventrite 2.

##### Description (male).

2.5–4.0 mm long (mean = 3.2 mm; n = 20); 1.8–2.7 times as long as wide. Head, pronotum and abdominal venter dark red-brown, antennae light brown, legs dark red-brown to light brown, elytra red-brown. Pronotum typically darker than elytra.

*Head.* Epistoma moderately emarginate; epistomal process strongly developed and elevated; median area above mandibles bearing dense patch of long, yellow, hair-like setae. Frons appearing flattened when viewed laterally, slightly transversely impressed just above epistoma; moderately, coarsely, longitudinally aciculate-punctate; aciculations converging at epistoma; punctures small, coarse; moderately and uniformly covered by long, fine, yellow erect hair-like setae, these longer than width of midpoint of eye. Antennal scape short, elongate; club flattened, irregularly ovoid, setose with partial septum, two sharply arcuate sutures visible.

*Pronotum* wider than long; apical margin broadly rounded, median area between eyes lined with scales; sides distinctly arcuate, strongly constricted near apex, forming a weak transverse impression near apical margin; surface smooth, shining, punctures on disc fine, shallow, moderately abundant, larger and more abundant laterally and on apical constriction; apical and anterolateral margins bearing sparse, erect, yellow, hair-like setae; base weakly bisinuate.

*Elytra* with sides sub-parallel on apical half, narrowing to subquadrate, weakly serrate apex; apex moderately emarginated at suture. Margin of apical edge bearing large, coarse punctures. Disc smooth, shining; interstriae not impressed, more than twice width of striae, interstrial punctures uniseriate, smaller than those of striae, bearing minute, recumbent setae less than length of a puncture; striae weakly impressed. Declivity bearing sparse, short, erect yellow setae. Metepimeron half-length of metanepisternum.

*Venter.* Apical margin of ventrite 1 strongly, acutely produced forming lip along base of ventrite 2, basal margin of ventrite 2 appearing impressed. Ventrite 2 nearly perpendicular to ventrite 1; surface glabrous, shining, rugose, finely punctate; punctures small, fine, shallow; surface flattened, unarmed or with weak median tumescence on apical margin; lateral margins of ventrites 2–3 and ventrite 4 unarmed. Ventrite 5 carinate ridge closer to apical margin of segment; length of ventrite 5 equal to combined lengths of ventrites 3 and 4; setal patch or median depression is absent.

**Figure 52.**
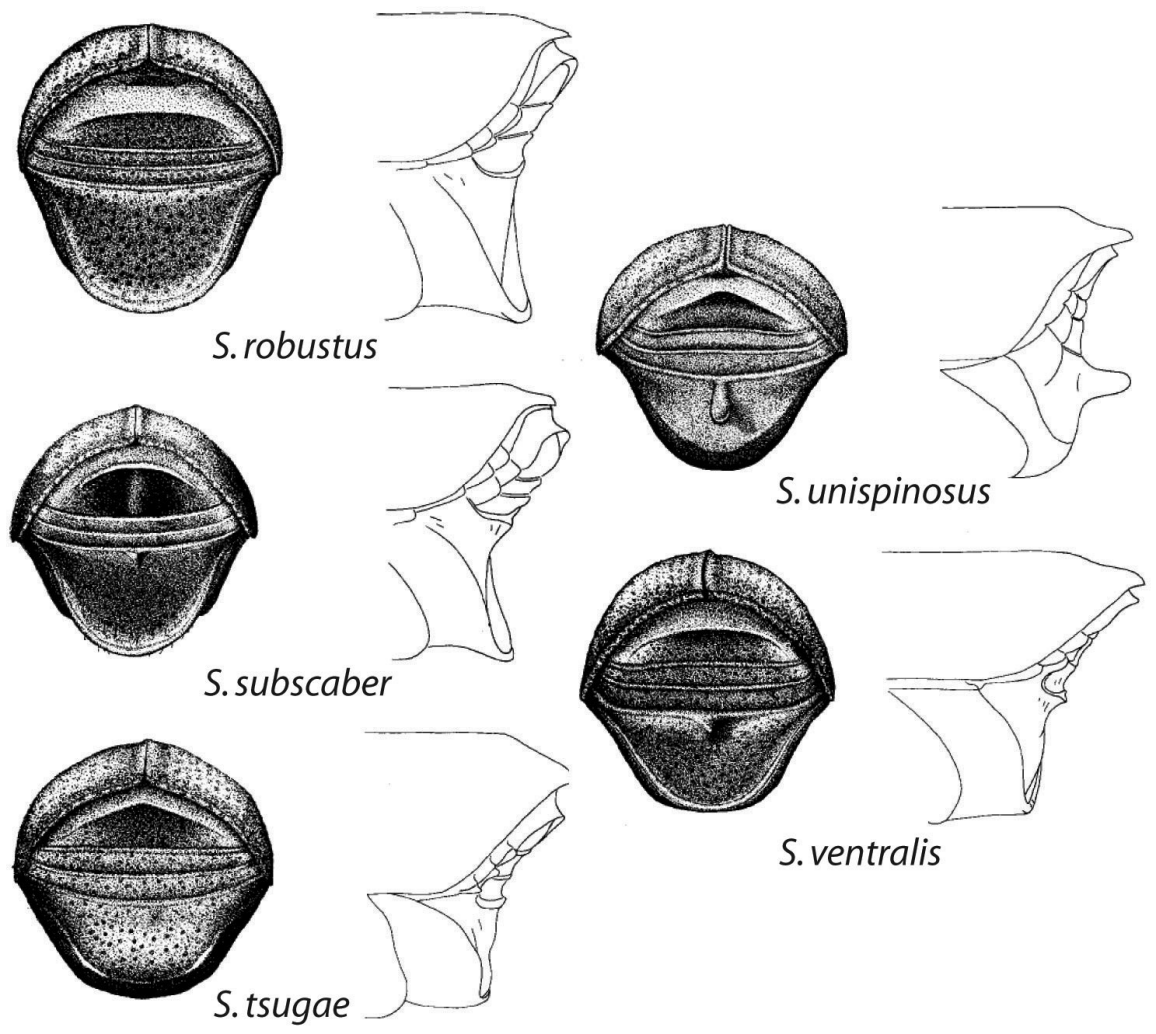
*Scolytus robustus*, *Scolytus subscaber*, *Scolytus tsugae*, *Scolytus unispinosus* and *Scolytus ventralis* male venters (from Edson 1967).

**Figure 53.**
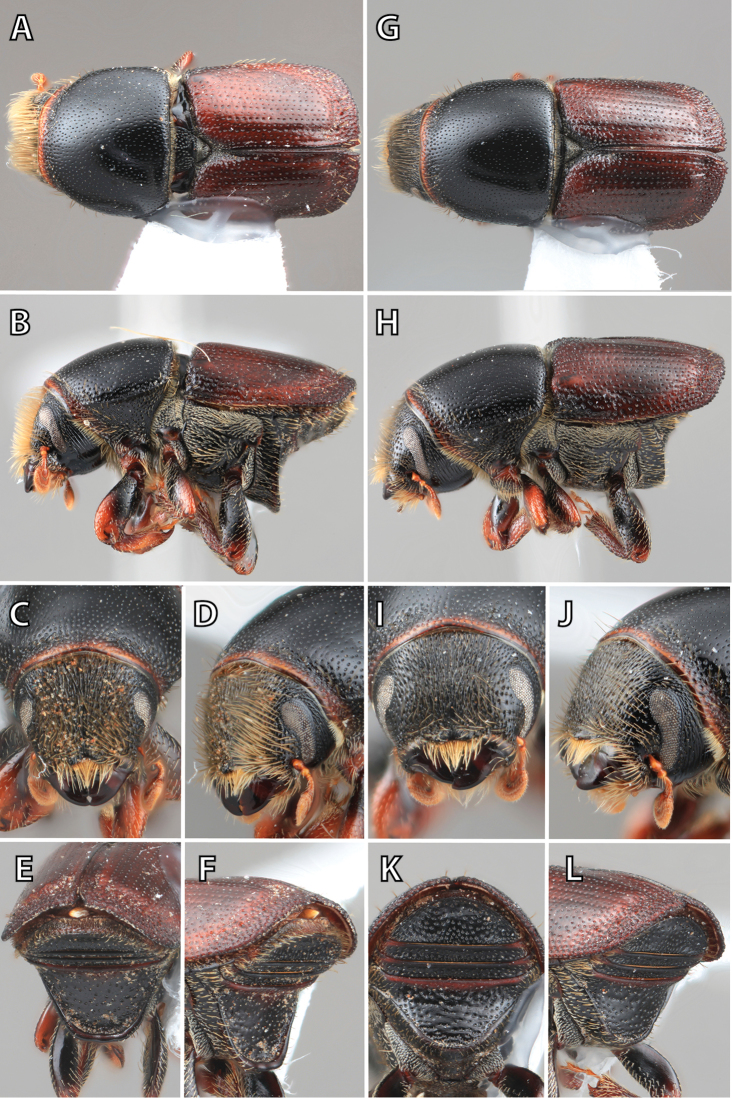
*Scolytus robustus* **A** dorsal male habitus **B** lateral male habitus **C** male frons **D** male frons oblique **E** male venter **F** male venter oblique **G** dorsal female habitus **H** lateral female habitus **I** female frons **J** female frons oblique **K** female venter **L** female venter oblique.

##### Female.

2.2–4.1 mm long (mean = 3.2 mm; n = 20); 1.9–2.5 times as long as wide. Similar to male except epistoma feebly emarginate, epistomal process less strongly developed and elevated, frons convex when viewed laterally, weakly longitudinally aciculate, setae sparser, shorter, less than width of eye; weakly transversely impressed between inner apices of eyes. Second ventrite unarmed.

##### Specimens examined.

143.

##### Type material.

Holotype *Scolytus robustus* Blackman: male, labeled “Prescott N.F. Ariz., VII-24-[19]30, M.W. Blackman Collector, *Abies concolor*, Hopk. US 20410E, Type No. 43835 USNM” (USNM).

##### Non-type material.

**UNITED STATES:** ***ARIZONA*:** [*Coconino Co.*]: Grand Canyon North Rim, Kaibab National Forest, VI.[19]66, [L. Edson] (EMEC-4). [*Graham Co.*]: Pinaleno Mountains, Swift Trail, 3 mi, Lady Bug Saddle, 11.IX.1964, C.W. O’Brien, ex. *Abies* sp. (CASC-1, CNCI-4, EMEC-2); Coronado National Forest, SR 366, N32°37.702', W109°49.472', 7896 ft, 24.V.2010, S.M. Smith, ex. *Abies concolor* (MSUC-2). ***COLORADO*:** *Costilla Co.*: near Fort Garland, Forbes Trinchera Ranch, VII-VIII.1976, D. Leatherman ex. white fir [= *Abies concolor*] (CSUC-1). *Huerfano Co.*: near Red Wing, 16.VII.1975, D. Leatherman, ex. white fir [= *Abies concolor*] (CSUC-4). Pass Creek, 20.VIII.1975, D. Leatherman, ex. white fir [= *Abies concolor*] (CSUC-2). *La Plata Co.*: Columbine Lake, 11.VIII.2004, D. Leatherman, ex. subalpine fir [= *Abies lasiocarpa*] (CSUC-1). ***NEVADA*:** *Clark Co.*: 19.VII.[19]29, C.C. Searl (DEBC-1, EMEC-4). Cathedral Rock, emerged 7-9.V.2002, R. Turnbow, ex. *Abies* sp. (WFBM-2). *White Pine Co.*: Baker, 17.V.1939, T.O. Thatcher (CNCI-2). ***NEW MEXICO*:** *Bernalillo Co.*: Cibola National Forest, Sandia Peak, 9.VII.[19]68, D.E. Bright (CNCI-1); NM536, N35°12.853', W106°24.743', 8753 ft, 10.V.2010, S.M. Smith, A.I. Cognato, ex. *Abies concolor* (MSUC-15), N35°11.655', W106°24.075', 8317 ft (MSUC-30). [*Santa Fe Co.*]: Santa Fe, Little Teseque Canyon, 14.VI.[19]35, Van Dyke (CASC-3). [*Unspecified County*]: Sandia Mountains, tree 9, 29.V.1969, S.L. Wood, ex. *Abies concolor* (MSUC-13), tree 18, 30.V.1969 (MSUC-19). ***TEXAS*:** [*Unspecified county*]: San Antonio, Hopk. U.S. 3938, W.F. Fiske, ex. bred 15.VI.[19]07 (USNM-3). ***UTAH*:** *Cache Co.*: Logan (CNCI-1). [*San Juan Co.*]: Mount Navajo, 8500 ft, 10.VI.[19]36, McAbee (CASC-4). *Utah Co.*: Payson Canyon, 14.V.1960, D.E. Bright, ex. *Abies concolor* (CNCI-3); 20.V.1961, S.L. Wood, ex. *Abies concolor* (USNM-9). [*Unspecified County*]: Bryce Canyon National Park, 10.V.1981, M.M. Furniss, ex. *Abies concolor* (WFBM-10). ***UNSPECIFIED STATE*:** (CASC-1).

##### Distribution.

UNITED STATES: Arizona, Colorado, Nevada, New Mexico, Texas, Utah (Fig. 34).

##### Hosts.

Principal host: *Abies concolor* (Gord. & Glend.) Lindl. ex Hildebr (white fir). Incidental host: *Abies lasiocarpa* (Hook.) Nutt (subalpine fir).

##### Biology.

*Scolytus robustus* is found in the limbs and tops of its host and fresh slash. Galleries are often initiated near limb bases (Edson 1967).

The adult gallery contains two egg galleries that branch from the central nuptial chamber. Gallery shape is quite variable and ranges from ‘S’ shaped at an oblique angle to the grain to nearly perpendicular against the grain (Fig. 25). Typically one egg gallery obliquely extends from the central nuptial chamber against the grain of wood for a short distance before becoming apically recurved. The second egg gallery is identical to the first but runs in the opposite direction. However galleries may extend perpendicular to the grain of the wood or may not be apically recurved. The adult gallery deeply scores the sapwood and ranges in size from 2.5–7.0 cm in length. Egg niches are distinct, score the sapwood and are closely spaced along the egg galleries. Larval mines rapidly diverge perpendicular to the adult gallery and are parallel to the grain of the wood. Larval mines terminate in pupation chambers that score the sapwood (Edson 1967).

##### Collection notes.

The senior author collected this species from 10.0 cm diameter slash with thick (5.0 mm) bark.

##### Remarks.

A relatively large amount of intraspecific variation (0.0537) was observed in mitochondrial COI sequences from specimens collected from Arizona and New Mexico and low variation observed between the two Arizona populations (Table 6). *Scolytus robustus* only occurs on *Abies*. In southeastern Arizona, *Abies* spp. and *Scolytus robustus* are confined to high elevation sites on the sky islands. These Arizona populations are isolated from the New Mexico populations by large areas that are unsuitable for *Abies*. No specific geographic morphological differences were observed among the examined specimens.

#### Scolytus silvaticus

Taxon classificationAnimaliaColeopteraCurculionidae

Bright, 1972 valid sp.

Fig. 54

Scolytus silvaticus Bright, 1972: 1489.

##### Diagnosis.

The male is distinguished from other species by having the apical margin of ventrite 4 thickened, forming a broad carina with a blunt median tubercle. The female is distinguished from the morphologically similar male of *Scolytus hermosus* by having the apical margin of ventrite 1 produced, forming a carinate lip along the basal margin of ventrite 2 that is about half as produced as thick and by the host and is distinguished from the *Scolytus hermosus* female by having a strongly developed epistomal process.

##### Description (male).

3.0 mm long (mean = 3.0 mm; n = 1); 2.1 times as long as wide. Color dark red-brown to black. Pronotum same color as elytra.

*Head.* Epistoma moderately, broadly emarginate; epistomal process weakly developed; median area above mandibles bearing dense patch of long, yellow, hair-like setae. Frons appearing flattened when viewed laterally, slightly transversely impressed just above epistoma; moderately, coarsely, aciculate-punctate; aciculations converging at epistoma; punctures small, coarse; moderately, uniformly covered by long, fine, erect, yellow-brown, hair-like setae, these longer than width of midpoint of eye. Antennal scape short, elongate; club flattened, almost subquadrate, setose with partial septum, two broadly arcuate sutures visible.

*Pronotum* wider than long; apical margin broadly rounded, median area between eyes lined with scales; sides distinctly arcuate, strongly constricted near apex, forming a weak transverse impression near apical margin; surface smooth, shining, punctures on disc fine, shallow, moderately abundant, larger and more abundant laterally and on apical constriction; apical and anterolateral margins bearing sparse, erect, dark yellow-brown setae; base weakly bisinuate.

*Elytra* with sides sub-parallel on apical half, narrowing to subquadrate, smooth apex; apex moderately emarginated at suture. Margin of apical edge bearing small, fine punctures. Disc smooth, shining; interstriae not impressed, more than twice width of striae, punctures uniseriate, smaller than those of striae; bearing sparse, recumbent, long, dark yellow-brown setae; striae weakly impressed. Declivity bearing abundant, long, erect dark yellow-brown hair-like setae. Metepimeron half-length of metanepisternum.

*Venter.* Apical margin of ventrite 1 moderately elevated above base of ventrite 2, ventrite 2 appearing impressed. Ventrite 2 nearly perpendicular to ventrite 1; surface smooth, shining, finely punctate; punctures small, fine, shallow; setae small, about two diameters of a puncture in length; surface flattened; lateral margins of ventrites 2–3 unarmed. Apical margin of ventrite 4 thickened forming a broad carina with blunt median tubercle. Ventrite 5 carinate ridge closer to apical margin of segment; length of ventrite 5 greater than combined lengths of ventrites 3 and 4; setal patch and median depression absent.

**Figure 54.**
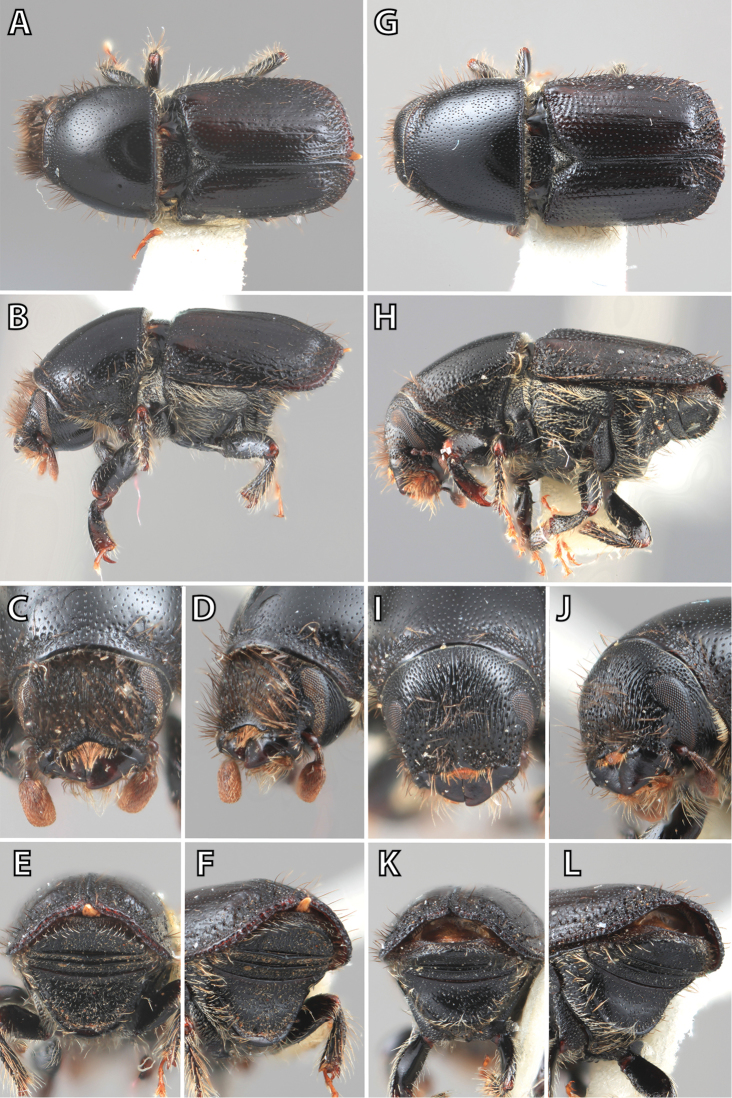
*Scolytus silvaticus* **A** dorsal male habitus **B** lateral male habitus **C** male frons **D** male frons oblique **E** male venter **F** male venter oblique **G** dorsal female habitus **H** lateral female habitus **I** female frons **J** female frons oblique **K** female venter **L** female venter oblique.

##### Female.

3.3–3.6 mm long (mean = 3.4 mm; n = 3); 2.2–2.8 times as long as wide. Similar to male except epistomal process more strongly developed, frons convex when viewed laterally, weakly aciculate, setae sparse, shorter, less than width of eye; weakly transversely impressed between inner apices of eye. Second ventrite unarmed.

##### Specimens examined.

4.

##### Type material.

Holotype *Scolytus silvaticus* Bright: male, labeled “MEX., N.L., Cerro Potosi, V.3.[19]71, 1100', D.E. Bright, *Pseudotsuga menziesii*, CNC No. 12603” (CNCI). Allotype, female, identical data as holotype. Paratypes, identical data as holotype (CNCI-2).

##### Non-type material.

None examined.

##### Distribution.

MEXICO: Nuevo León (Fig. 32).

##### Hosts.

*Pseudotsuga menziesii* (Mirb.) Franco (Douglas fir).

##### Biology.

*Scolytus silvaticus* is only known from a single collecting event from broken branches of *Pseudotsuga menziesii* (Bright 1972). The gallery and biology of this species are unknown but the gallery structure is presumably similar to that of other *Pseudotsuga* feeding *Scolytus*, parallel to the grain of the wood.

##### Remarks.

Wood (1975: 22) placed *Scolytus silvaticus* in synonymy with *Scolytus hermosus* because it occured on the same mountain as *Scolytus silvaticus*. Wood reasoned that only one *Scolytus* species could occur in the same location and that the observed morphological variation was due to intraspecific differences. In addition, he stated that Bright’s image of the *Scolytus silvaticus* male was a normal male of *Scolytus hermosus*. We here remove *Scolytus silvaticus* from synonymy with *Scolytus hermosus* because of the many distinct morphological and host differences originally noted by Bright (1972). In the male of *Scolytus silvaticus*, the apical margin of ventrite 1 is moderately thickened and weakly produced, the posterior margin of ventrite 3 is slightly medially thickened and the posterior margin of ventrite 4 is in strongly produced and thickened medially, forming a broad carina with a blunt median tubercle. In the male of *Scolytus hermosus* ventrite 1 apical margin is thickened and strongly produced and ventrites 3–4 are flat and *Scolytus hermosus* colonizes *Abies* species rather than *Pseudotsuga*. It is also not uncommon for multiple *Scolytus* species to have overlapping distributions.

#### Scolytus subscaber

Taxon classificationAnimaliaColeopteraCurculionidae

LeConte, 1876

Figs 52 , 55

Scolytus subscaber LeConte, 1876: 373.

##### Diagnosis.

The male most closely resembles those of *Scolytus obelus* and *Scolytus praeceps*. It is distinguished from that of *Scolytus obelus* by the sparse, obscure, fine and shallow punctures of ventrite 2, by the dull luster of ventrite 2 and by the geographic distribution. The male can be distinguished from that of *Scolytus praeceps* by the presence of a median denticle on the apical margin of ventrite 2. Females closely resemble those of *Scolytus ventralis* and are distinguished by the distinctly, moderately longitudinally aciculate and weakly punctate frons, and by the weakly produced apical margin of ventrite 1 that forms a weak carinate lip along the basal margin of ventrite 2.

##### Description (male).

2.0–4.3 mm long (mean = 3.5 mm; n = 20); 1.8–2.3 times as long as wide. Head, pronotum and abdominal venter dark red-brown, antennae light brown, legs dark red-brown to light brown, elytra red brown. Pronotum typically darker than elytra.

*Head.* Epistoma moderately, very broadly emarginate; epistomal process moderately developed and elevated; median area above mandibles bearing dense patch of long, yellow, hair-like setae. Frons appearing convex when viewed laterally, slightly transversely impressed just above epistoma; moderately, coarsely, longitudinally aciculate-punctate; aciculations converging at epistoma; punctures minute, fine; moderately and uniformly covered by long, fine, yellow, erect hair-like setae, these longer than width of midpoint of eye. Antennal scape short, elongate; club flattened, ovoid, setose with partial septum, three arcuate sutures visible.

*Pronotum* wider than long; apical margin broadly rounded, median area between eyes lined with scales; sides distinctly arcuate, strongly constricted near apex, forming a weak transverse impression near apical margin; surface smooth, shining, punctures on disc fine, shallow, moderately abundant, larger and more abundant laterally and on apical constriction; apical and anterolateral margins bearing sparse, erect, yellow setae; base weakly bisinuate.

*Elytra* with sides sub-parallel on apical half, narrowing to subquadrate, smooth apex; apex moderately emarginated at suture. Margin of apical edge bearing small, fine punctures. Disc smooth, shining; interstriae not impressed, more than twice width of striae, punctures uniseriate, smaller than those of striae; punctures bearing short, sparse, recumbent yellow setae slightly longer than size of a puncture (may be abraded); striae weakly impressed. Declivity bearing sparse, short, erect yellow setae. Metepimeron half-length of metanepisternum.

*Venter.* Apical margin of ventrite 1 strongly, acutely produced forming lip along base of ventrite 2, basal margin of ventrite 2 appearing impressed. Ventrite 2 nearly perpendicular to ventrite 1; surface glabrous, shagreened, dull, finely and obscurely punctate; punctures small, fine, shallow; surface weakly concave; apical margin armed with broad median denticle; lateral margins of ventrites 2–3 and ventrite 4 unarmed. Ventrite 5 carinate ridge closer to apical margin of segment; length of ventrite 5 greater than combined lengths of ventrites 3 and 4; setal patch and median depression absent.

**Figure 55.**
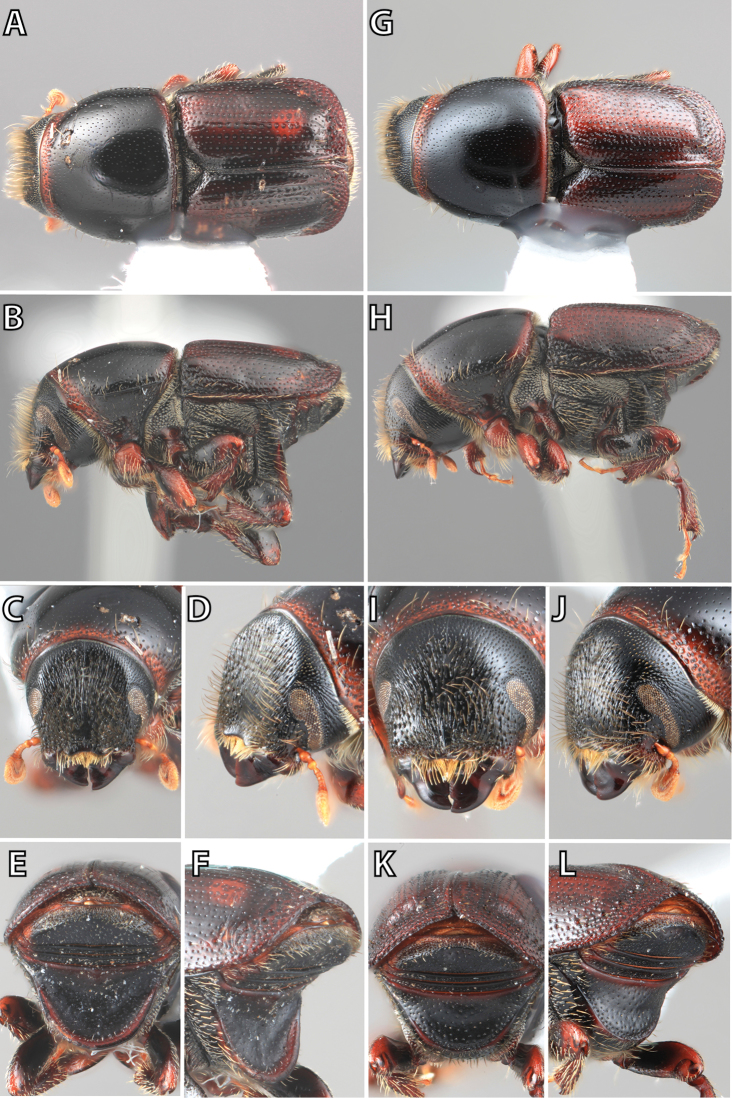
*Scolytus subscaber* **A** dorsal male habitus **B** lateral male habitus **C** male frons **D** male frons oblique **E** male venter **F** male venter oblique **G** dorsal female habitus **H** lateral female habitus **I** female frons **J** female frons oblique **K** female venter **L** female venter oblique.

##### Female.

3.1–5.0 mm long (mean = 3.85 mm; n = 20); 1.9–2.6 times as long as wide. Similar to male except epistoma feebly emarginate, epistomal process absent, frons more strongly convex when viewed laterally, weakly longitudinally aciculate, setae sparser, shorter, less than width of eye. Apical margin of ventrite 1 weakly elevated above base of ventrite 2. Second ventrite unarmed, surface weakly rugose, punctures larger, deeper.

##### Specimens examined.

111.

##### Type material.

Lectotype *Scolytus subscaber* LeConte: female, Vanc. [Vancouver, B.C.], Type 968” (MCZC). Lectotype designated Wood 1982: 443.

##### Non-type material.

**UNITED STATES:** ***CALIFORNIA*:** *Alpine Co.*: Stanislaus National Forest, Hermit Valley, Hopk. U.S. 19193-A, J.M. Miller, ex. *Abies concolor* (EMEC-1, OSAC-4). Humboldt-Toiyabe National Forest, Hwy 89, 6.6 mi E. NF 4188, N38°39.906', W119°38.540', 8011 ft, 24.VII.2010, S.M. Smith, ex. *Abies magnifica* (MSUC-3). *El Dorado Co.*: Echo Lake, Hopk. U.S. 18381-A, 27.V.[19]31, J.M. Miller, ex. *Abies magnifica* (CSUC-1, EMEC-17, OSAC-20, USNM-3). Lake Tahoe, Fallen Leaf Lake, 22.VII.1930, A.C. Browne (CASC-1). Uncle Tom’s, 0.1 road mile W., 28.VII-12.VIII.1978, J.A. Benedictis, ex. from pheromone trap baited with E-11 tetradecenyl acetate (EMEC-1). [*Madera Co.*]: Bass Lake, Hopk. U.S. 19376-A, 2.VII.[19]32, G.R. Struble, ex. *Abies concolor* (EMEC-2, OSAC-3). Sugar Pine [community], 12.VIII.1920, E. Schiffel (CASC-1). *Placer Co.*: Lake Tahoe, 1 mi N., 3.VIII.[19]67, G.T. Ferrell (EMEC-3). *Plumas Co.*: LaPort, Hopk. U.S. 17933-A, J.M. Miller, ex. *Abies magnifica* (EMEC-2, OSAC-2). [*Unspecified County*]: Stanislaus National Forest, Hopk. U.S. 19818, J.M. Miller, ex. *Abies concolor* (OSAC-1). ***IDAHO*:** *Clearwater Co.*: Pierce, 4 mi W.N.W., 18.VII.1973, H.L. Osborne, ex. flight trap (USNM-1). *Latah Co.*: 1992, M.M. Furniss, ex. *Abies grandis* (WFBM-1). Flat Creek, 11.II.1995, M.M. Furniss, ex. *Abies grandis* (WFBM-4). Harvard, 3.5 mi N.N.E., 6.VIII.1973, H.L. Osborne, ex. flight trap (USNM-1). Moscow, 20.VII.[19]73, LC-1 (USNM-1), 24.VII.[19]73, LC-1 (USNM-2). Potlatch, 4 mi N.E., 3.VIII.1973, H.L. Osbourne, ex. flight trap (USNM-1). ***OREGON*:** [*Benton Co.*]: [Corvallis], Kiger Island, IX.1922, W.J. Chamberlain (USNM-1). [*Klamath Co.*]: Crater Lake [National Park], 11.VI.[19]33, Hopk. U.S. 18966-A, W.J. Buckhorn, ex. *Abies concolor* (EMEC-4), 14.XII.[19]33 (EMEC-3, OSAC-20, WFBM-1). ***WASHINGTON*:** [*Chelan Co.*]: Lake Wenatchee [State Park], VII.[19]69 (EMEC-1). [*King Co.*]: Seattle, 9.VI.[19]12 (OSAC-2). [*Thurston Co.*]: Olympia, 25.IV.[18]94 (OSAC-2).

##### Distribution.

**Figure 56.**
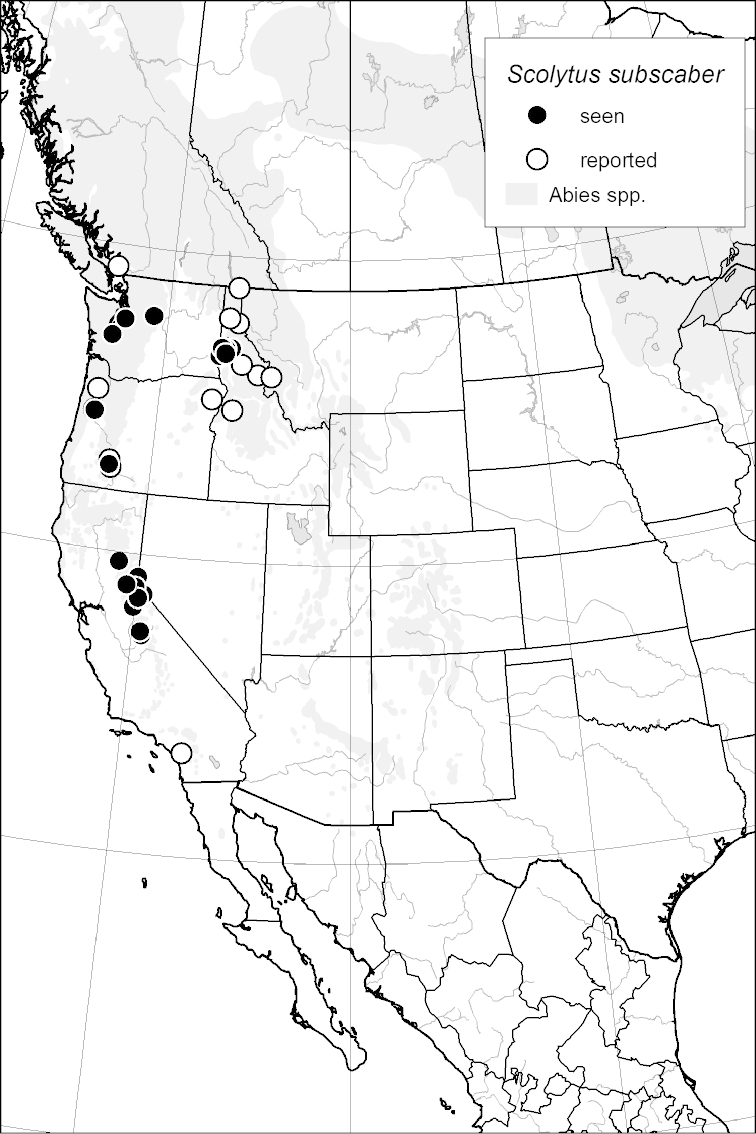
*Scolytus subscaber* distribution map.

CANADA: British Columbia. UNITED STATES: California, Idaho, Montana, Oregon, Washington (Fig. 56).

##### Hosts.

Principal host: *Abies grandis* (Dougl. Ex D. Don) Lindl. (grand fir). Incidental hosts: *Abies lasiocarpa* (Hook.) Nutt. (subalpine fir), *Abies magnifica* A. Murr. (red fir).

##### Biology.

Furniss and Kegley (2011) provide a detailed and thorough account of the biology of *Scolytus subscaber*. Adults infest suppressed branches in the crowns of mature trees and are rarely found in slash (Edson 1967; Furniss and Kegley 2011). *Scolytus subscaber* is also associated with a staining fungus, *Spicaria anomala* (Corda) Harz. that kills the host cambium (Wright 1938).

Adult galleries are distinct from those of other *Scolytus* species. They are epsilon (“**ε**”) shaped and deeply excavated in the sapwood (Fig. 25). The gallery consists of a central nuptial chamber and two egg galleries that are recurved around the nuptial chamber (Edson 1967). The adult gallery is 2.0–5.0 cm in length and varies by branch size, with larger branches having larger galleries (Furniss and Kegley 2011). Egg niches lightly score the sapwood. In Oregon 12–30 eggs are laid per gallery and in Idaho the upper limit appears to be 12 (Furniss and Kegley 2011). Larval mines radiate from the egg gallery in all directions, often crossing each other. The larval mines are located in the phloem and cambium for about the first centimeter of their length. After the first centimeter, larval mines lightly score the sapwood. Pupation chambers are formed in the cambium or outer bark. There is one generation per year and the brood overwinters as larvae. In Idaho, flight occurs in July and adults leave the gallery once eggs have been laid (Furniss and Kegley 2011).

##### Collection notes.

Old galleries of this species were observed by the senior author while hiking along the Tuolumne Grove Trail in Yosemite National Park on 23.VII.2010.

##### Remarks.

The lectotype bears a partial locality label. LeConte’s (1868) description states the lectotype was collected at Vancouver Island.

#### Scolytus tsugae

Taxon classificationAnimaliaColeopteraCurculionidae

(Swaine, 1917)

Figs 52 , 57

Eccoptogaster tsugae Swaine, 1917: 32.
Scolytus monticolae (Swaine, 1917): Keen 1929: 12.

##### Diagnosis.

*Scolytus tsugae* most strongly resembles *Scolytus monticolae* and the two species are easily and often confused. Both sexes are distinguished from those of *Scolytus monticolae* by the impressed elytral discal striae, giving the elytra a corrugated appearance, by the dull luster of ventrite 2 and the host genus *Tsuga*.

##### Description (male).

2.8–3.4 mm long (mean = 3.1 mm; n = 16); 2.1–2.5 times as long as wide. Body dark red-brown and antennae light brown. Pronotum typically darker than elytra.

*Head.* Epistoma weakly emarginate; epistomal process absent; median area above mandibles bearing dense patch of long, yellow, hair-like setae. Frons appearing flattened when viewed laterally, slightly transversely impressed just above epistoma; weakly longitudinally aciculate, moderately punctate; aciculations converging at epistoma; punctures small, coarse; moderately, uniformly covered by long, fine, erect, yellow, hair-like setae, these longer than width of midpoint of eye. Antennal scape short, elongate; club flattened, irregularly ovoid, setose with partial septum, two arcuate sutures visible.

*Pronotum* wider than long; apical margin broadly rounded, median area between eyes lined with scales; sides distinctly arcuate, strongly constricted near apex, forming a weak transverse impression near apical margin; surface smooth, shining, punctures on disc fine, shallow, moderately abundant, larger and more abundant laterally and on apical constriction; apical and anterolateral margins bearing sparse, erect, dark yellow-brown, hair-like setae; base weakly bisinuate.

*Elytra* with sides sub-parallel on basal half, narrowing to subquadrate, smooth apex; apex moderately emarginated at suture. Margin of apical edge bearing large, coarse punctures. Disc glabrous, smooth, shining; interstriae weakly impressed, more than twice width of striae, interstrial punctures uniseriate, smaller than those of striae; striae weakly impressed, elytra with a corrugated appearance. Declivity bearing sparse, short, erect yellow setae. Metepimeron less than half-length of metanepisternum.

*Venter.* Apical margin of ventrite 1 weakly, continuously elevated above base of ventrite 2. Ventrite 2 nearly perpendicular to ventrite 1; surface glabrous, shagreened, dull, finely punctate; punctures small, fine, shallow; surface flattened; apical margin unarmed; lateral margins of ventrites 2–3 and ventrite 4 unarmed. Ventrite 5 carinate ridge closer to apical margin of segment; length of ventrite 5 greater than combined lengths of ventrites 3 and 4; setal patch and median depression absent.

**Figure 57.**
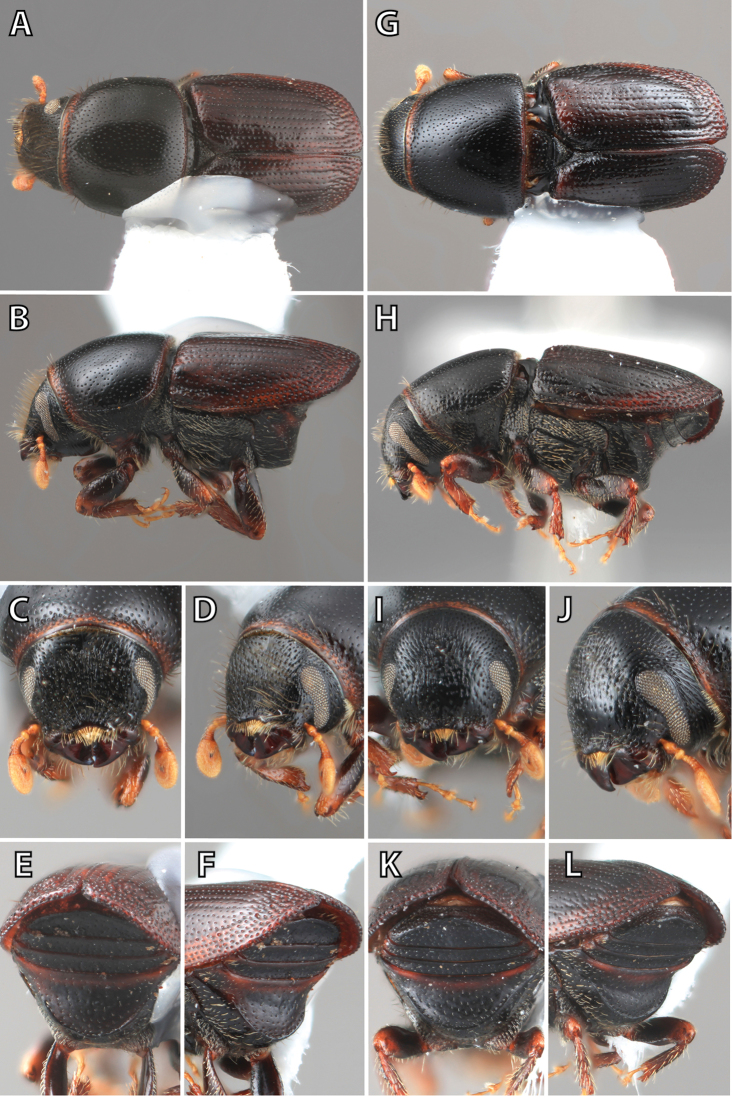
*Scolytus tsugae* **A** dorsal male habitus **B** lateral male habitus **C** male frons **D** male frons oblique **E** male venter **F** male venter oblique **G** dorsal female habitus **H** lateral female habitus **I** female frons **J** female frons oblique **K** female venter **L** female venter oblique.

##### Female.

2.3–3.5 mm long (mean = 3.0 mm; n = 16); 1.9–2.8 times as long as wide. Similar to male except epistoma feebly emarginate, epistomal process present, moderately developed, low, frons convex when viewed laterally, weakly strigate, setae sparser, shorter, less than width of eye; weakly transversely impressed between inner apices of eyes.

##### Specimens examined.

369.

##### Type material.

Lectotype *Eccoptogaster tsugae* Swaine: female, labeled “Entomological Branch, Ottawa, Canada No. 2251, female, J.M. Swaine Coll., Lectotype CNCNo. 9239” (CNCI). Lectotype designated Bright 1967: 674. Paralectotypes *Eccoptogaster tsugae* (CNCI), [*Unspecified locality*]: 2251 (CNCI-12, CUIC-2, EMEC-1), 2327 (CNCI-2).

##### Non-type material.

**CANADA:** ***BRITISH COLUMBIA*:** Adams Lake, 8.V.[19]22, R. Hopping, ex. *Tsuga mertensiana* (CASC-1). Bowman Creek, 10.VIII.1928, R. Hopping, ex. *Tsuga heterophylla* (CASC-2). Garibaldi, 7.VII.1988, R.J. Rabaglia (RJRC-1). Merritt, Midday Valley, 5.VI.1926, W. Mathers, 17134, lot 94, ex. *Pinus ponderosa* (CASC-1), 11.VI.1926, 17190, lot 23 (CNCI-1). North Vancouver, Lynn Canyon, 1.VI.[19]23, 17003, N.L. Cutler, ex. *Abies amabalis* (CASC-12, CNCI-2). Pender Harbour, 17189, lot 1, 11.V.[19]26, G.R. Hopping, ex. *Tsuga heterophylla* (CNCI-9); lot 2, 12.V.[19]26 (CASC-1, CNCI-8), lot 4, 29.V.[19]26 (CASC-1, CNCI-6), lot 5, 1926 (CNCI-1), 13.VI.1928 (CASC-4). Terrace, Mrs. W.W. Hippisley (CNCI-1). Trinity Valley, 16.VIII.1927, J.R. Howell (CASC-2), 1722, lot 46, 10.VII.1928, (CASC-1), lot 50, 24.VII.1928 (CASC-1), 17339, lot 12, 19.VI.[19]30, (CASC-2); 16.VI.1927, 17213, lot 27, E.A. Rendell (CASC-1). Vancouver, 11.VI.1935, A. Graham, ex. *Tsuga mertensiana* (CASC-2); 27.V.1939, W.G. Mathers, ex. *Tsuga heterophylla* (CASC-2), 31.V.1939 (CASC-5), 5.VI.1939 (CASC-3), 12.VI.1939 (CASC-3), 27.VI.1939 (CASC-5), 4.VII.1939 (CASC-1), 10.VII.1939 (CASC-2). **UNITED STATES:** ***CALIFORNIA*:** *Alpine Co.*: Ebbetts Pass, 8730 ft, 13.VIII.[19]63, D.E. Bright, ex. *Tsuga mertensiana* (CNCI-8, EMEC-9). *El Dorado Co.*: [Georgetown, 10 mi E.], Blodgett [Experimental] Forest, 2.VI.2003, K. Apigian (EMEC-1), 30.V.1986, K. Hobson (EMEC-1). Pollock Pines, 22.VI.[19]48, A. Bartel (EMEC-1). *Lassen Co.*: Grassy Lake, 27.IX.[19]14, lot 142, ex. *Tsuga mertensiana* (CASC-2). *Nevada Co.*: Tahoe National Forest, Sagehen Basin, Carpenter Ridge, 39.4149°N, 120.3109°W, 15.VII.2003, M. Caterino (SBMN-1). [*Unspecified County*]: Yosemite National Park, Hopk. U.S. 15727-A, 2.VII.1918, J.E. Patterson, ex. *Tsuga mertensiana* (OSAC-4). ***IDAHO*:** *Bonner Co.*: Priest River Experimental Forest, Hopk. U.S. 61810, 28.X.1978, M.M. Furniss, ex. *Tsuga heterophylla* (USNM-8). Priest Lake, Reader Bay, 6.VIII.1985, M.M. Furniss, J.B. Johnson, ex. *Tsuga heterophylla* (WFBM-54). *Boundary Co.*: Idaho Panhandle National Forest, Roman Nose, N48°40.911', W116°34.345', 4353 ft, 12.VIII.2010, S.M. Smith, A.R. Gillogly, ex. *Tsuga heterophylla* (MSUC-7). *Kootenai Co.*: Deception Creek Experimental Forest, Hopk. U.S. 58885-B, 10.VII.1968, M.M. Furniss, ex. *Tsuga heterophylla* (OSAC-1), 16.VII.1968 (OSAC-1), 24.VII.1968 (OSAC-4), 30.VII.1968 (OSAC-2), 8.VIII.1968 (OSAC-1). Magee, VII.[19]29, R.L. Furniss, ex. hemlock [= *Tsuga* sp.] (OSAC-4). ***MONTANA*:** [*Unspecified County*]: Glacier [National] Park, 8.VII.[19]49, D. Giuliani (CASC-1). ***OREGON*:** [*Douglas Co.*]: Diamond Lake, Hopk. U.S. 20959-A, VII.[19]31, R.L. Furniss, ex. *Tsuga mertensiana* (OSAC-18). *Hood River Co.*: Mount Hood National Forest, Hwy 35, Sherwood Forest campground, N45°19.278', W121°37.104', 4293 ft, 2.VIII.2010, S.M. Smith, ex. *Tsuga heterophylla* (MSUC-5). *Klamath Co.*: Crescent Lake, 6.VII.[19]60, ex. *Tsuga mertensiana* (CASC-4). Crater Lake [National Park], Hopk. U.S. 18.916-A, 22.V.[19]30, W.J. Buckhorn, ex. *Tsuga mertensiana* (OSAC-30), Hopk. U.S. 18,950-A, 16.VI.[19]31 (OSAC-44); Hopk. U.S. 18851-A, 14.VI.[19]31, J.A. Beal, ex. *Tsuga mertensiana* (OSAC-6, USNM-2); Hopk. U.S. 20537-A, 17.VI.[19]33, F.P. Keen, ex. *Tsuga mertensiana* (OSAC-19); 24.VIII.[19]62, D.E. Bright, ex. *Tsuga mertensiana* (CNCI-1); 12.VIII.1984, M.M. Furniss, ex. *Tsuga mertensiana* (WFBM-16); Hopk. U.S. 20807-A, R.L. Furniss, ex. *Tsuga heterophylla* (OSAC-2). *Linn Co.*: Santiam Pass 7.VII.[19]64 (EMEC-1). [*Yamhill Co.*]: McMinnville, 27.XI.1937, K.M. & D.M. Fender (OSAC-1). ***Washington*:** *King Co.*: Seattle, 27.V.[19]07 (OSAC-1), 10.IV.[19]12 (OSAC-2), 12.IV.[19]12 (OSAC-1). [*Snohomish Co.*]: [labeled *King Co.*] Mountlake Terrace, 20.VIII.[19]62, D.E. Bright, ex. *Tsuga heterophylla* (CNCI-4). ***UNSPECIFIED LOCALITY*:** Hopk. U.S. 13247-A, ex. *Tsuga mertensiana* (OSAC-2). Summit Viola Trail, 9.IX.1910, J.M. Miller, ex. *Tsuga mertensiana* (OSAC-1).

##### Distribution.

CANADA: Alberta, British Columbia. UNITED STATES: California, Idaho, Montana, Oregon, Washington (Fig. 36).

##### Hosts.

*Tsuga heterophylla* Sarg. (western hemlock) and *Tsuga mertensiana* (Bong.) Carrière (mountain hemlock).

##### Common name.

Hemlock engraver.

##### Biology.

*Scolytus tsugae* attacks fresh slash, the main bole and large branches of hemlock (Edson 1967).

*Scolytus tsugae* is an uncommon and poorly studied species. The adult galleries are typically perpendicular to the grain of the wood and 4.0–10.0 cm in length (Fig. 25) (Edson 1967; Furniss and Johnson 2002). Adult galleries score the cambium slightly more than the sapwood. However, Edson (1967) reported that specimens from a series in northern California produced an adult gallery that was oriented obliquely to the grain of the wood. Galleries consist of a central nuptial chamber and two egg galleries. Each egg gallery is extended perpendicular to the grain of the wood from the central nuptial chamber. Egg niches are irregularly spaced and faintly score the sapwood. Larval tunnels are extended parallel to the grain of the wood, etching the sapwood lightly at first and deeply near the pupation chamber. There is one generation per year and broods overwinter as larvae (Furniss and Johnson 2002).

##### Collection notes.

The senior author collected this species from fresh logging slash limbs that were 6.0–10.0 cm in diameter in Idaho and Oregon.

##### Remarks.

The lectotype does not bear a locality label. Swaine’s (1917) description states the type series was collected at “Cherry Creek valley, Vernon District, British Columbia, Glacier, B.C., Jasper Park, Alta.” from both *Tsuga mertensiana* and *Pseudotsuga mucronata* [= *Pseudotsuga menziesii*]. In Bright’s (1967) lectotype designation he lists the locality of the lectotype as “Glacier, BC, XI-26-15, *Tsuga mertensiana*”.

For many years *Scolytus monticolae* was considered a synonym of *Scolytus tsugae* (see *Scolytus monticolae* remarks). In their paper describing the biology of *Scolytus tsugae*, McMullen and Atkins (1959) actually described the biology of *Scolytus monticolae*. In their investigation the species studied was from *Pseudotsuga menziesii* rather than *Tsuga* spp. and created vertical instead of transverse galleries.

#### Scolytus unispinosus

Taxon classificationAnimaliaColeopteraCurculionidae

LeConte, 1876

Figs 52 , 58

Scolytus unispinosus LeConte, 1876: 372.
Scolytus sobrinus Blackman, 1934: 23.

##### Diagnosis.

*Scolytus unispinosus* is very morphologically similar to *Scolytus fiskei* and *Scolytus laricis*. Males are distinguished from those of *Scolytus laricis* by the frons flattened when viewed laterally, never deeply impressed, less moderately abundant frontal setae (compared to dense) and by the host genus, *Pseudotsuga*. Males are distinguished from those of *Scolytus fiskei* by the following combination of characters: abdominal venter dull in luster, the base of the ventrite 2 spine extending from the apical margin to half the length of the segment and by the geographical distribution. The female is distinguished from that of *Scolytus fiskei* by the dull luster of ventrite 2 and is distinguished from that of *Scolytus laricis* by the finely aciculate-punctate frons; ventrite 1 joining base of ventrite 2 more obtusely, base of ventrite 2 not finely impressed, flush with ventrite 1; epistomal process weakly developed, almost indistinct.

##### Description (male).

2.2–3.2 mm long (mean = 2.7 mm; n = 15); 2.3–2.7 times as long as wide. Head, antennae, pronotum, and abdominal venter dark red-brown. Elytra and legs yellow-brown to light brown. Pronotum typically darker than elytra.

*Head.* Epistoma weakly emarginate; epistomal process present, weakly developed, low; median area above mandibles bearing dense patch of long, yellow, hair-like setae. Frons appearing flattened when viewed laterally from epistoma to vertex, slightly transversely impressed just above epistoma to inner apices of eyes; moderately, coarsely, longitudinally aciculate-punctate; aciculations converging at epistoma; punctures sparse, small, fine; sparsely, uniformly covered by long, fine, erect, yellow, hair-like setae, thesee longer than width of midpoint of eye. Antennal scape short, elongate; club flattened, irregularly ovoid, setose with partial septum, two arcuate sutures visible.

*Pronotum* wider than long; apical margin broadly rounded, median area between eyes lined with scales; sides distinctly arcuate, strongly constricted near apex, forming a weak transverse impression near apical margin; surface smooth, shining, punctures on disc fine, shallow, moderately abundant, larger and more abundant laterally and on apical constriction; apical and anterolateral margins bearing sparse, erect, yellow, hair-like setae; base weakly bisinuate.

*Elytra* with sides sub-parallel on apical half, narrowing to subquadrate, smooth apex; apex moderately emarginated at suture. Margin of apical edge bearing small, fine punctures. Disc smooth, shining; interstriae not impressed, twice width of striae, punctures uniseriate, smaller than those of striae; bearing short, sparse, recumbent yellow setae slightly longer than size of a puncture (may be abraded); striae weakly impressed. Declivity bearing sparse, short, erect yellow setae. Metepimeron less than half-length of metanepisternum.

*Venter.* Apical margin of ventrite 1 rounded, marked by weak carina. Ventrite 2 nearly perpendicular to ventrite 1; surface shagreened, dull, finely punctate; punctures small, fine, shallow, nearly obscure; covered with sparse setae that are less than length of segment 3; surface convex; apical margin armed with laterally compressed, median spine with base extending from apical margin to half length of segment, apex rounded; lateral margins of ventrites 2–3 and ventrite 4 unarmed. Ventrite 5 carinate ridge closer to apical margin of segment; length of ventrite 5 less than combined lengths of ventrites 3 and 4; setal patch and median depression absent.

**Figure 58.**
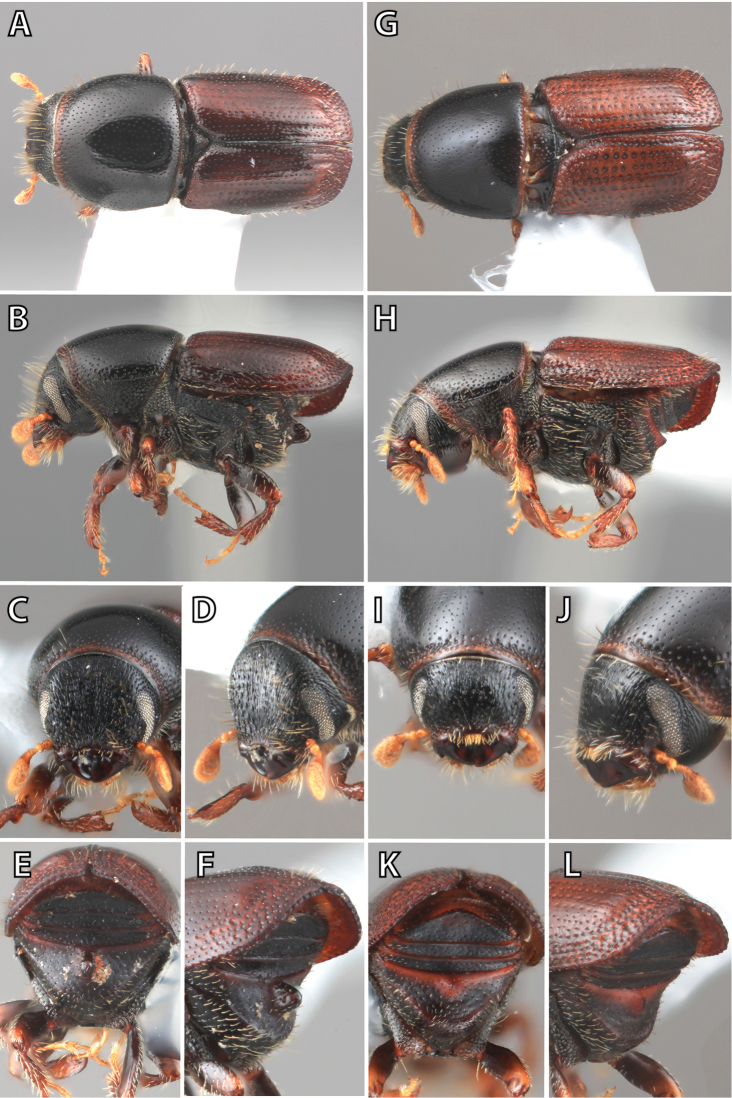
*Scolytus unispinosus* **A** dorsal male habitus **B** lateral male habitus **C** male frons **D** male frons oblique **E** male venter **F** male venter oblique **G** dorsal female habitus **H** lateral female habitus **I** female frons **J** female frons oblique **K** female venter **L** female venter oblique.

##### Female.

2.2–3.0 mm long (mean = 2.7 mm; n = 15); 2.3–2.7 times as long as wide. Similar to male except epistoma feebly emarginate, epistomal process feebly developed, frons convex when viewed laterally, weakly longitudinally aciculate, setae sparser, shorter, less than width of eye; weakly transversely impressed between epistoma and inner apices of eyes. Second ventrite apical margin armed with acute median denticle, with base extending from apical margin to half-length of segment.

##### Specimens examined.

654.

##### Type material.

Lectotype *Scolytus unispinosus* LeConte: male, labeled “Or. [Oregon], Type 965” (MCZC). Lectotype designated Wood 1982: 432. Holotype *Scolytus sobrinus* Blackman: male, labeled “Burke Colr, Kent, WA, *Pseudotsuga taxifolia* [= *Pseudotsuga menziesii*], Hopk. US 41900, Type No. 43838” (USNM). Synonymy: Wood 1966: 30. Paratypes *Scolytus sobrinus* Blackman **UNITED STATES:** ***OREGON*:** [*Jackson Co.*]: Little Applegate River, Hopk. U.S. 14238-B, J.E. Patterson (EMEC-6). Ashland, Hopk. U.S. 14264-T, F.P. Keen (EMEC-3). ***WASHINGTON*:** [*King Co.*]: Kent, Hopk. U.S. 4190-A, [H.E.] Burke, ex. *Pseudotsuga taxifolia* [= *Pseudotsuga menziesii*] (EMEC-5).

##### Non-type material.

**CANADA:** ***BRITISH COLUMBIA*:** Cloverdale, 28.XII.[19]22, N.L. Cutler, ex. *Tsuga heterophylla* (CASC-32, CNCI-11). Duncan, Genoa Bay, 10.VII.1928, W.G. Mathers, ex. *Pseudotsuga taxifolia* [= *Pseudotsuga menziesii*] (CNCI-1). Indian Meadows, Midday Creek, 13.VII.1920, R. Hopping, ex. *Pinus ponderosa* (CNCI-1). Merritt, Midday Valley, 31.V.1926, W. Mathers (CNCI-1), 2.VII.1926 (CNCI-2); R. Hopping 3.VIII.1923 (CNCI-1), 15.VIII.19[23] (CNCI-1), 7.VII.1926 (CNCI-1). Nanaimo, [Pacific] Biological Station, 23.VI.1920 (CASC-1). Nelson Island, West Lake, 1701, 23.VI.1922, R. Hopping, ex. *Pseudotsuga taxifolia* [= *Pseudotsuga menziesii*] (CNCI-12). Oliver, 25 mi N.W., 15.VI.1958, H. & A. Howden (EMEC-3). Pender Harbour, 30.VI.1928, G. Hopping, ex. *Pseudotsuga taxifolia* [= *Pseudotsuga menziesii*] (CASC-2). Squamish, Diamond Head trail, 3200 ft, 9.VIII.1953, G.J. Spencer (CNCI-1). Shuswap, 24.IV.[19]13, T. Wilson, ex. Douglas fir [= *Pseudotsuga menziesii*] (CNCI-1). Vancouver, 7.VI.1934, W. Mathers ex. *Pseudotsuga taxifolia* [= *Pseudotsuga menziesii*] (CASC-1), 11.VI.1934 (CASC-2). Vanguard, 23.VI.[19]32, R. Hopping, ex. *Pseudotsuga mucronata* [= *Pseudotsuga menziesii*] (CASC-2, CNCI-2). Vernon, 27.V.1932, R. Hopping (CASC-1). **UNITED STATES:** ***CALIFORNIA*:** *Del Norte Co.*: 3.VII.[19]24, E.B. Leach (EMEC-1). *El Dorado Co.*: [Georgetown, 10mi E.], Blodgett Experimental Forest, 4000 ft, 26.V.1986, Hobson, Atkinson, ex. Lindgren trap, ponderosa pine resin, H&L B.P. fractions, burned over area (EMEC-3); 27.V.1986, Hobson, Irving, ex. Lindgren trap, ponderosa pine resin, untreated Oleo, burned over area (EMEC-2); 30.V.1986, Hobson, Atkinson, ex. Lindgren trap, unbaited, logged area (EMEC-1); 30.V.1986, Hobson, Atkinson, ex. Lindgren trap, unbaited, burned over area (EMEC-2). Placerville, Hopk. U.S. 33961-A, 26.III.[19]54, M.M. Furniss, ex. *Pseudotsuga taxifolia* [= *Pseudotsuga menziesii*] (EMEC-16). Pollack Pines, 22.VI.[19]48, R.C. Bynum (EMEC-6). *Humboldt Co.*: Blocksburg, 13.V.[19]34, B.P. Biven (CASC-2). Orleans, 15 mi N.W., 13.VI.[19]64, D.E. Bright, ex. *Pseudotsuga taxifolia* [= *Pseudotsuga menziesii*] (EMEC-7). Orick, 11 mi S., 11.VI.[19]62, D.E. Bright, B.A. Barr, ex. *Pseudotsuga taxifolia* [= *Pseudotsuga menziesii*] (DEBC-4, EMEC-3). *Lake Co.*: Whispering Pines, 14.IV.1964, ex. Douglas fir [= *Pseudotsuga menziesii*] (CASC-1). *Los Angeles Co.*: Coquillett (EMEC-2). *Madera Co.*: 1.VII.[19]38 (EMEC-1). *Marin Co.*: Inverness, 11.X.[19]61, D.E. Bright, ex. *Pseudotsuga taxifolia* [= *Pseudotsuga menziesii*] (DEBC-3, EMEC-2). [*Mariposa Co.*]: [Yosemite National Park], Yosemite Valley, Hopk. U.S. 2810, Hopkins (EMEC-4). *Mendocino Co.*: (CASC-7). 14.VII.[19]22, E.R. Leach (CASC-4). Bransomb, CDFA#870697, 11.VII.[20]01, M. Garvin, ex. Lindgren funnel trap with ISP (CSCA-1), 25.VII.[20]01 (CSCA-3). Flynn Creek between Comptche and Navarro, 17.VIII.1953, P.S. Bartholomew (CASC-1). Mill Creek, 20.III.[19]59, R.E. Stevens, ex. Douglas fir [= *Pseudotsuga menziesii*] (EMEC-13). Noyo River, VI.1896 (CASC-14). *Napa Co.*: Angwin, 2 mi N.N.E., North side of Howell Mountain, 1300 ft, 24.V.1974, H.B. Leech, ex. emerged from log of *Pseudotsuga menziesii* (CASC-3, USNM-6), 7.VII.1974 (CASC-3), 20.VII.1974 (CASC-16), 21.VII.1974 (CASC-11), 22.VII.1974 (CASC-4), 23.VII.1974 (CASC-8), 25.VII.1974 (CASC-15), 26.VII.1974 (CASC-2, USNM-3), 27.VII.1974 (CASC-1), 28.VII.1974 (CASC-6, USNM-3), 1.VIII.1974 (CASC-3), 3.VIII.1974 (CASC-3). Callistoga, 26.V.[19]57 (CASC-4); 4.X.1947, T.O. Thatcher, ex. *Pseudotsuga taxifolia* [= *Pseudotsuga menziesii*] (EMEC-3); 1.V.[19]63, D.E. Bright, ex. *Pseudotsuga menziesii* (EMEC-3). *Nevada Co.*: Grass Valley, 17.I.1961, ex. *Pseudotsuga taxifolia* [= *Pseudotsuga menziesii*] (CASC-2). Middleton [labeled as *Lake Co.*], Hopk. U.S. 37588-A, R.W. Bushing, ex. *Pseudotsuga menziesii* (EMEC-3), G.M. Thomas (EMEC-3). [*Placer Co.*]: Colfax, 1 mi E., Hopk. U.S. 34068-A, 31.I.[19]37, ex. *Pseudotsuga menziesii* (EMEC-4). Towle, 11.XI.1932 (EMEC-1). *Plumas Co.*: Walker Mine, 15.VII.[19]30 (EMEC-3). [*San Francisco Co.*]: San Francisco, Hopk. U.S. 8557, 11.V.[18]99, ex. on pine [= *Pinus* sp.] (EMEC-1), 15.V.[18]99 (EMEC-1). *Santa Cruz Co.*: 16.IV.[19]59, J.E. Henry (WFBM-16). Aptos, New Brighton Beach State Park, 13.IX.1986, D. Adams, ex. *Pinus radiata* (EMEC-54). *Shasta Co.*: Hat Creek, 17.VI.[19]62, D.E. Bright, B.A. Barr, ex. *Abies concolor* (EMEC-2). *Siskiyou Co.*: Grass Valley, 16.X.[19]60, R.W. Bushing, emerged XI.1960, ex. *Pseudotsuga menziesii* (EMEC-6). *Sonoma Co.*: (CNCI-1). Fort Ross, 2 mi E., 2.XI.1947, T.O. Thatcher, ex. *Pseudotsuga taxifolia* [= *Pseudotsuga menziesii*] (EMEC-3). Mount Saint Helena, 3.VI.[19]31, E.C. Zimmermann, ex. digger pine [= *Pinus sabiniana*] (EMEC-1); Hopk. U.S. 21,125-F, 3.IV.1934, R.L. Furniss, ex. *Pseudotsuga taxifolia* [= *Pseudotsuga menziesii*] (EMEC-4). Sebastopol, Hopk. U.S. 32638-A, 20.I.1940, ex. *Pseudotsuga taxifolia* [= *Pseudotsuga menziesii*] (DEBC-4, EMEC-5). Stillwater Cove, 12.V.1951, H.R. Moffitt (EMEC-1). *Trinity Co.*: 14.V.[19]23, ex. *Pseudotsuga taxifolia* [= *Pseudotsuga menziesii*] (CASC-4). [*Unspecified County*]: (CUIC-7). Yosemite National Park, Hopk. U.S. 20953-B, 19.VI.1932, J.M. Miller, ex. *Pseudotsuga taxifolia* [= *Pseudotsuga menziesii*] (EMEC-8). ***IDAHO*:** *Bonner Co.*: Sandpoint, 13.VI.931, N.M. Downie (FMNH-1), 9.VII.1977 (FMNH-1); 9.VII.1977 (FMNH-1). ***OREGON*:** [*Benton Co.*]: Corvallis, 17.IV.[19]16, W.J. Chamberlin, ex. *Pseudotsuga taxifolia* [= *Pseudotsuga menziesii*] (EMEC-3); 8.VII.1946, K.R. Hobbs (EMEC-1). *Coos Co.*: Myrtle Point, 18.VI.[19]64, D.E. Bright, ex. *Pseudotsuga menziesii* (EMEC-10). *Curry Co.*: Agness, 6 mi S.W., 10.VII.1990, M.M. Furniss, J.B. Johnson, ex. *Pseudotsuga menziesii* (WFBM-6). Brookings, 12 mi E.N.E., 12.VIII.1990, M.M. Furniss, J.B. Johnson, ex. *Pseudotsuga menziesii* (WFBM-4). Illahe, 4 mi N., 9.VIII.[19]90, M.M. Furniss, J.B. Johnson, ex. *Pseudotsuga menziesii* (WFBM-2). *Deschutes Co.*: Deschutes National Forest, Black Butte Rd, Black Butte, N44°24.924', W121°38.323', 4212 ft, 31.VIII.2010, S.M. Smith, ex. *Pseudotsuga menziesii* (MSUC-44). *Douglas Co.*: Roseburg, W.J. Chamberlin, ex. *Pseudotsuga taxifolia* [= *Pseudotsuga menziesii*] (DEBC-2, EMEC-6, WFBM-2). [*Grant Co.*]: Dixie Pass, Malheur National Forest, 23.VI.1961, S.L. Wood, J.B. Karren, D.E. Bright, ex. *Pseudotsuga taxifolia* [= *Pseudotsuga menziesii*] (DEBC-5). *Jackson Co.*: Dead Indian Spring, 17.V.1962, J. Schuh (CNCI-1). Mistletoe, Hopk. U.S. 15753-A, P.D. Sergent, ex. *Pseudotsuga taxifolia* [= *Pseudotsuga menziesii*] (EMEC-3), Hopk. U.S. 15753-B, 21.VIII.1918, P.D. Sargent (DEBC-5, EMEC-10). Pinehurst, 2 mi E., 18.VII.[19]64, D.E. Bright, ex. *Pseudotsuga menziesii* (CNCI-5, EMEC-6). Prospect, 10.VII.[19]64, D.E. Bright, ex. *Pseudotsuga menziesii* (CNCI-11, EMEC-14). [*Klamath Co.*]: Klamath Falls, 17.VI.[19]64, D.E. Bright, ex. *Pseudotsuga menziesii* (EMEC-2). [*Lane Co.*]: McCredie Springs, 19.VI.1961, D.E. Bright, ex. *Tsuga heterophylla* (CNCI-2). Vaughn, J. Pierce, ex. Douglas fir limbs [= *Pseudotsuga menziesii*] (WFBM-4); 21.VI.1955, J.A. Rudinsky, J.R. Pierce, ex. Doug fir branches [= *Pseudotsuga menziesii*] (WFBM-3). *Linn Co.*: Santiam Pass, 19.VI.1951, S.L. Wood, J.B. Karren, D.E. Bright, ex. *Pseudotsuga taxifolia* [= *Pseudotsuga menziesii*] (DEBC-1). [*Malheur Co.*]: Monument Peak, 25.IX.[19]53, ex. *Pseudotsuga taxifolia* [= *Pseudotsuga menziesii*] (EMEC-3). [*Marion Co.*]: Clear Lake, 17.VIII.[19]51, R. Kangur, ex. Larch [= *Larix* sp.] ((EMEC-4, WFBM-2). [*Multnomah Co.*]: Portland, Hubbard, Schwarz (EMEC-2). [*Umatilla Co.*]: Tollgate, 30.VI.1950, E.S. McClurskey, ex. on aluminum roof (CNCI-1), 1.IX.1950 (CNCI-1). [*Unspecified County*]: Detroit, 25 mi E., 17.VII.1939, Schuh, Scott, ex. *Tsuga mertensiana* (FMNH-6). Middle Sister Mountain, 8000 ft, Hopk. U.S. 53349-G, 4.VIII.1968, M.M. Furniss, ex. on snowfield (WFBM-2). Portland, Wickham (CNCI-1). Tillamook burn, 18.VII.1941, R. Kangur (EMEC-1). Warm Springs Indian Reservation, 15.VIII.[19]51, R. Kangur, ex. Douglas fir [= *Pseudotsuga menziesii*] (EMEC-4). ***WASHINGTON*:** [*Clallam Co.*]: Port Angeles, Hopk. U.S. 130, A.D. Hopkins, ex. *Pseudotsuga taxifolia* [= *Pseudotsuga menziesii*] (EMEC-1). Port Williams [Marlyn Nelson County Park at Port Williams], Hopk. U.S. 168-F, A.D. Hopkins, ex. *Pseudotsuga taxifolia* [= *Pseudotsuga menziesii*] (EMEC-1). *Cowlitz Co.*: Castle Rock, 10 mi E., 27.VI.[19]64, D.E. Bright, ex. *Pseudotsuga menziesii* (DEBC-18, EMEC-9). [*Grays Harbor Co.*]: Humptulips, 28.V.1914, E.C. VanDyke (CASC-3). [*Kittitas Co.*]: Easton (CASC-6, EMEC-2). *Okanogan Co.*: Disautel, 4.XI.1936, R.L. Furniss, ex. *Pseudotsuga menziesii* (OSAC-17, WFBM-7). [*Pend Oreille Co.*]: Metaline Falls, Hopk. U.S. 21340, 17.VII.1931, W.D. Bedard, ex. *Pseudotsuga taxifolia* [= *Pseudotsuga menziesii*] (WFBM-15). *Skamania Co.*: Mineral Springs, 27.VI.[19]64, D.E. Bright, ex. *Pseudotsuga menziesii* (EMEC-9). [*Snohomish Co.*]: [labeled *King Co.*] Mountlake Terrace, 20.VIII.[19]62, ex. *Pseudotsuga menziesii* (CNCI-3). *Thurston Co.*: Olympia, 7-30.V.1996, E. LaGasa, ex. Washington Department of Agriculture port trapping survey (WFBM-1). *Yakima Co.*: Naches, 14 mi W., Dry Creek Ridge, Snoqualamie National Forest, 3.VII.1965, R.B. Hutt (DEBC-2). [*Unspecified County*]: Mount Adams, Bird Creek, 6000-7000 ft, 2.VII.1925 (CASC-1). Mount Rainier National Park, 21.VIII.[19]62, ex. *Pseudotsuga taxifolia* [= *Pseudotsuga menziesii*] (CNCI-6).

##### Distribution.

CANADA: British Columbia. UNITED STATES: California, Idaho, Oregon, Washington (Fig. 51).

##### Hosts.

*Pseudotsuga menziesii* (Mirb.) Franco (Douglas fir) but also likely occurs in *Pseudotsuga macrocarpa* (Vasey) Mayr (bigcone Douglas fir) in Southern California. Incidental in *Abies*, *Pinus* and *Tsuga*.

##### Common name.

Douglas-fir engraver.

##### Biology.

*Scolytus unispinosus* is very common (Chamberlin 1939; Smith pers. obs.) and attacks the boles and branches of weakened, injured, dying and recently killed Douglas fir. Populations of this species can build up in windfalls, slash and during drought when the species becomes capable of killing young trees (Keen 1938; Chamberlin 1939; Wood et al. 2003). Outbreaks are sporadic and are of short duration (Keen 1938; Wood et al. 2003).

The adult gallery of this species consists of a single egg gallery that extends with the grain of the wood (Fig. 25). The nuptial chamber includes a short extension at a 45° angle from the egg gallery and is located at one end on the egg gallery. The gallery deeply scores the sapwood and lightly scores the cambium (Chamberlin 1958). The female deposits 40–100 eggs in egg niches on both sides of the egg gallery 0.5–1.0 mm apart (Doane et al. 1936). Larvae extend their mines at a right angle to the egg gallery forming a fan shaped pattern (Chamberlin 1939). There are two generations per year in California and the broods overwinter as either eggs or larvae (Keen 1938; Wood et al. 2003). Adults emerge from late April through July (Keen 1958). In Oregon one generation per year has been observed at high elevations in and two at lower elevations (Chamberlin 1939).

McMullen and Atkins (1962) reported some notes on the biology of *Scolytus unispinosus* in British Columbia and appear to have reported a combined account of *Scolytus unispinosus* and *Scolytus fiskei* (see *Scolytus fiskei* biology for more information). This paper was the main source of information regarding the biology of *Scolytus unispinosus* and served as the basis for describing the biology of the species in numerous publications (including Bright 1976; Furniss and Carolin 1977; Furniss and Johnson 2002; Wood et al. 2003). Chamberlin (1939, 1958) provides the most reliable source of information regarding the biology of the species.

##### Remarks.

There are 25 *Scolytus sobrinus* specimens bearing paratype labels found in the EMEC. These specimens are not designated as such by Blackman (1934) and have been labeled “not Paratype” by the authors. These specimens bear the following Hopkins numbers: 4205-A (EMEC-2), 4272-A (EMEC-4), 1968-D1 (EMEC-1), 1968-D3 (EMEC-1), 4273-B (EMEC-1), 4204 (EMEC-2), 4220-A (EMEC-3), 4226-D (EMEC-1), 4201-A (EMEC-2), 13232-C (EMEC-1), 17 (EMEC-1), 18 (EMEC-1), 19 (EMEC-2) and Sonoma, California (EMEC-3).

This species occurs along the western coastal states of the United States and British Columbia, Canada. The range of *Scolytus unispinosus* does not overlap with that of *Scolytus fiskei* except in south central British Columbia where species are sympatric in the Interior Plateau near Merritt.

#### Scolytus ventralis

Taxon classificationAnimaliaColeopteraCurculionidae

LeConte, 1868

Figs 52 , 59

Scolytus ventralis LeConte, 1868: 167.

##### Diagnosis.

Males are distinguished from those of other species by having the base of ventrite 2 elevated, the surface of ventrite 2 flat, the apical margin of ventrite 2 often bearing a median denticle, and by the glabrous ventrite 2. Females most closely resemble those of *Scolytus robustus* and *Scolytus subscaber*. Females are distinguished from those of both species by the indistinctly and weakly aciculate and strongly punctate frons and by the apical margin of ventrite 1 flush with basal margin of ventrite 2, appearing rounded.

##### Description (male).

3.0–4.0 mm long (mean = 3.65 mm; n = 20); 2.1–2.7 times as long as wide. Head, pronotum and abdominal venter dark red-brown, antennae light brown, elytra and legs yellow-brown to light brown. Pronotum typically darker than elytra.

*Head.* Epistoma weakly emarginate; epistomal process weakly elevated; median area above mandibles bearing dense patch of long, yellow, hair-like setae. Frons appearing convex when viewed laterally, slightly transversely impressed just above epistoma; moderately, coarsely, longitudinally aciculate-punctate; aciculations converging at epistoma; punctures small, coarse; moderately, uniformly covered by long, fine, yellow, erect, hair-like setae, these longer than width of midpoint of eye. Antennal scape short, elongate; club flattened, irregularly ovoid, setose with partial septum, three arcuate sutures visible.

*Pronotum* wider than long; apical margin broadly rounded, median area between eyes lined with scales; sides distinctly arcuate, strongly constricted near apex, forming a weak transverse impression near apical margin; surface smooth, shining, punctures on disc fine, shallow, moderately abundant, larger and more abundant laterally and on apical constriction; apical and anterolateral margins bearing sparse, erect, yellow, hair-like setae; base weakly bisinuate.

*Elytra* with sides sub-parallel on apical half, narrowing to subquadrate, smooth apex; apex moderately emarginated at suture. Margin of apical edge bearing large, coarse punctures. Disc glabrous, smooth, shining; interstriae not impressed, more than twice width of striae, interstrial punctures uniseriate, smaller than those of striae; striae weakly impressed. Declivity bearing sparse, short, erect yellow setae. Metepimeron half-length of metanepisternum.

*Venter.* Apical margin of ventrite 1 weakly elevated above base of ventrite 2. Ventrite 2 nearly perpendicular to ventrite 1; surface glabrous, shagreened, dull, finely punctate; punctures small, fine, shallow; surface flattened; apical margin armed with broad median denticle, occasionally absent; lateral margins of ventrites 2–3 and ventrite 4 unarmed. Ventrite 5 carinate ridge closer to apical margin of segment; length of ventrite 5 less than combined lengths of ventrites 3 and 4; setal patch and median depression absent.

**Figure 59.**
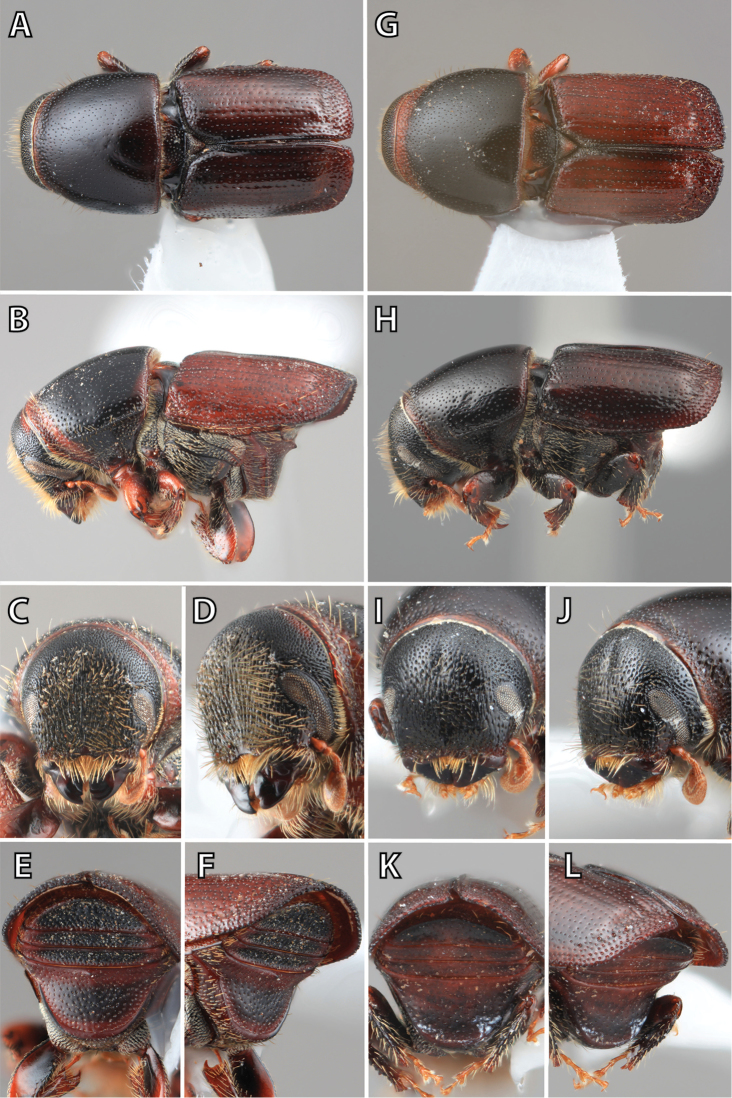
*Scolytus ventralis* **A** dorsal male habitus **B** lateral male habitus **C** male frons **D** male frons oblique **E** male venter **F** male venter oblique **G** dorsal female habitus **H** lateral female habitus **I** female frons **J** female frons oblique **K** female venter **L** female venter oblique.

##### Female.

2.2–4.6 mm long (mean = 3.56 mm; n = 20); 2.1–2.7 times as long as wide. Similar to male except epistoma feebly emarginate, epistomal process absent, frons convex when viewed laterally, weakly longitudinally aciculate, setae sparser, shorter, less than width of eye; weakly transversely impressed between epistoma and inner apices of eyes. Second ventrite unarmed.

##### Specimens examined.

324.

##### Type material.

Lectotype: male, labeled “[Washington Territory] Type 970” (MCZC). Lectotype designated Wood 1982: 441.

##### Non-type material.

**CANADA:** ***BRITISH COLUMBIA*:** Duncan, Genoa Bay, 30.VI.1928, W.G. Mathers, ex. *Abies grandis* (CNCI-4), 21.VI.1928 (CNCI-1), 23.VI.1928 (CASC-1), 24.VI.1928 (CASC-1), 16.VII.1928 (CASC-1), 13.VIII.1936 (CASC-1). Steelhead, 6.VII.1933, H.B. Leech, ex. *Abies amabalis* (CNCI-1), 27.VII.1933 (CASC-1), 24.VII.1933 (CNCI-1), 26.VII.1933 (CNCI-1). Trail, 22 km S.E., 24.V.1991, J.E. Macias, ex. *Abies grandis* (CNCI-1). Vancouver, 10.VI.1935, K. Graham, ex. *Abies grandis* (CASC-12). **MEXICO:** ***Baja California*:** San Pedro Mártir, Hopk. U.S. 32716-A, 5.VI.1944, F.P. Keen, ex. *Abies concolor* (EMEC-1). **UNITED STATES:** ***ARIZONA*:** *Cochise Co.*: Coronado National Forest, Chiricahua Mountains, 14.VII.2009, J. Hulcr, ex. *Pseudotsuga menziesii* (MSUC-3). ***CALIFORNIA*:** [*Amador Co.*]: Jackson, 31.III.[19]55, ex. *Abies concolor* (EMEC-1). *Calaveras Co.*: Big Trees [State Park], VII.1930, F.E. Blaisdell (CASC-1). *El Dorado Co.*: Bijou at south end of Lake Tahoe, H.B. Leech, ex. Douglas fir log [= *Pseudotsuga menziesii*], emerged San Francisco, 18-20.V.1965, W.E. Kuhl (CASC-8). [Georgetown, 10 mi E.], Blodgett [Experimental] Forest, 30.V.1986, K. Hobson (EMEC-1), 11.VI.1986 (EMEC-1), 21–24.VI.1986 (EMEC-6), 25-27.VI.1986 (EMEC-1), 1–7.VII.1986 (EMEC-2), 9–16.VII.1986 (EMEC-4); 30.V.1986, Hobson, Atkinson, ex. Lindgren trap, unbaited over burned area (EMEC-3), ex. Lindgren trap, turpentine bait, logged area (EMEC-1); 27.V.1986, Hobson, Irving, ex. Lindgren trap, ponderosa pine resin H&L B.P. fractions, logged area (EMEC-1), ex. Lindgren trap, ponderosa pine resin, untreated oleo, burned over area (EMEC-1). Ice House Reservoir, 23.VI.[19]90, J.B. Johnson (EMEC-1). Pacific, 8.VII.[19]62, R.L. McDonald (CASC-1). *Fresno Co.*: Huntington Lake, 16.VII.[19]19, F.E. Blaisdell (CASC-5). *Lassen Co.*: Lassen National Forest, 20–25.VII.1994, S1/2 S35 T34N R7E, A.I. Cognato (MSUC-20, SBMN-4); Black Mountain Experimental Forest, VII.1995, A.I. Cognato (MSUC-1). Near Loon Lake campground, 6300 ft, 28.VII–12.VIII.1978, J.A. De Benedictis (EMEC-1). Norval Flats, 5500 ft, 18.VII.[19]20, J.O. Martin, ex. *Abies concolor* (CASC-47). Uncle Tom’s, 0.1 road mile W., 28.VII-12.VIII.1978, J.A. De Benedictis (EMEC-1). *Marin Co.*: VI-VIII.[19]56, D. Giuliani (CASC-1). *Mariposa Co.*: Miami Ranger Station, 2mi S., 19.VII.1946, H.P. Chandler (DEBC-3). Miami Lodge, 17.VII.1946, G.R. Struble, ex. *Abies concolor* (CSUC-1). *Mendocino Co.*: Noyo River, VI.1896 (CASC-22). *Modoc Co.*: Modoc National Forest, Hwy 299, 5.3 mi W. Cedarville at creek near exit from ski area, 41°32.9’N 120°14’W, 9.IX.1995, J. Schweikert, ex. swept creek side plants (CASC-1). Davis Creek, 4 mi N.E., Sugar Hill, 6300 ft, 9.VI.1970, W. Middlekauff (EMEC-1). *Nevada Co.*: Sagehen Creek Field Station, 39.4298°N, 120.2429°W, 12-18.VIII.2003, M. Caterino, ex. FIT (SBNM-1). Near Sagehen campground, 39.4344°N, 120.2808°W, 15.VIII.2003, M. Caterino (SBNM-1). Sagehen Creek, 20.VII.1966, W.J. Turner (EMEC-1). [*Placer Co.*]: Cisco (USNM-1). *Plumas Co.*: Bartle, 9 mi N., 12-15.VI.1974, L. Green (EMEC-1). Chester, 8mi N.W., Warner Creek, 5000 ft, 21.VI.1989, E.E. Lundquist (CNCI-2). Janesville, 15-21.VIII.[19]50, M. Wasbauer, ex. *Abies concolor* (EMEC-1). *Riverside Co.*: Mount San Jacinto State Park, 33.800°N, 116.673°W, 15.VII.2003, M. Caterino (SBMN-1). Santa Rosa Mountains, 10.IV.[19]63, D.E. Bright, ex. *Abies concolor* (EMEC-1). [*San Bernardino Co.*]: San Bernardino Mountains, Dollar Lake trail, 10.VII.1956, H.W. Michalk (CSCA-1). *Siskiyou Co.*: Happy Camp, 18 mi N., 31.VII.[19]63, C.J. Wray, ex. *Picea breweriana* (DEBC-2, EMEC-1). Klamath National Forest, goosenest adaptive management area, 41.5°N, 121.9°W, 26.VII.2000, ex. pitfall 3-840 (SBNM-1). McCloud, 22.VI.1914 (CASC-23), 14.VI.1962, D.E. Bright, B.A. Barr, C. Hector (CASC-1). *Tehama Co.*: Mineral, 11.VIII.[19]35 (CASC-1). *Trinity Co.*: Carville, 30.VI.1913, ex. dug out of *Abies concolor* (CASC-1), 2400-2500 ft, 23.V.1934 (CASC-1). Nash Mine, 12.VI.1913 (CASC-1). *Tulare Co.*: Giant Forest, 6500 ft, VII.1908, Hopping (CASC-3). Kaweah, 100 ft, Hopping (CASC-1). Sequoia National Park, Redwood Canyon, 20.IX.1980, S.F. Muzzio (CASC-2). [*Unspecified county*]: ***COLORADO*:** *Costilla Co.*: Pass Creek, 17.VI.1976, D. Leatherman, ex. white fir [= *Abies concolor*] (CSUC-4). *Huerfano Co.*: near Red Wing, 16.VI.1975, D. Leatherman, ex. white fir [= *Abies concolor*] (CSUC-1). *Las Animas Co.*: Monument Lake, 6.VII.1994, S. Kelley, ex. white fir [= *Abies concolor*] (DEBC-2). *Pueblo Co.*: SR 165, 5 mi S.E. San Isabel Millset trailhead, D. Leatherman, ex. burned white fir [= *Abies concolor*] (CSUC-6). ***IDAHO*:** *Benewah Co.*: Plummer, 4 mi S., 14.VIII.1956, W.F. Barr, E.C. Clark, ex. *Abies grandis* (FMNH-1). *Boundary Co.*: Idaho Panhandle National Forest, Hwy 95, Robinson Lake campground, N48°58.200', W116°13.067', 2696 ft, 13.VIII.2010, S.M. Smith, A.R. Gillogly, ex. *Abies grandis* (MSUC-6). *Kootenai Co.*: Coeur d’Alene, 5.IX.1919, J.C. Evenden, ex. *Abies grandis* (MSUC-3). ***MONTANA*:** [*Unspecified county*]: Glacier [National] Park, 15.VII.[19]31 (CUIC-1). ***NEW MEXICO*:** *Bernalillo Co.*: Cibola National Forest, Sandia Peak, NM536, N35°11.655', W106°24.075', 8317 ft, 10.V.2010, S.M. Smith, A.I. Cognato, ex. *Abies concolor* (MSUC-1). [*Otero Co.*]: Cloudcroft, 900 ft, W. Knaus (CNCI-1). *Santa Fe Co.*: Little Tesuque Canyon, 14.VI.[19]35 (CASC-1). *Socorro Co.*: VII.[?], Wickham (CASC-1). ***OREGON*:** *Benton Co.*: Corvallis, VIII.1919, W.J. Chamberlin, ex. *Pseudotsuga taxifolia* [= *Pseudotsuga menziesii*] (EMEC-1), Kiger Island, VII.[19]22, W.J. Chamberlin (EMEC-2). Mary’s Peak, 14 mi W. Corvallis, XII.1958 (EMEC-2). [*Deschutes Co.*]: Bend, Hopk. U.S. 33,531-B, 8.VII.[19]58, W.J. Buckhorn, P.W. Orr, ex. *Abies grandis* (OSAC-8). [*Klamath Co.*]: Klamath Falls, Geary Ranch, 4.X.1962, J. Schuh, ex. *Abies concolor* (CNCI-2, FMNH-4, MSUC-7). [*Linn Co.*]: McMinnville, 7.VIII.1951, R. Kangur (WFBM-2). [*Wallowa Co.*]: Wallowa Lake, 19.VII.[19]51, Quintus, ex. *Abies concolor* (WFBM-1). ***UTAH*:** *Juab Co.*: Mount Nebo, 20.VII.1958, D.E. Bright, ex. *Abies concolor* (CNCI-2). *Utah Co.*: Payson Canyon, 20.V.1961, S.L. Wood, ex. *Abies concolor* (USNM-18). ***WASHINGTON*:** [*Jefferson Co.*]: Quilcene, Hopk. U.S. 65564, 30.IX.1946 (OSAC-6). [*Pend Oreille Co.*]: Metaline Falls, Hopk. U.S. 21405, 17.VII.[19]31, W.D. Bedard, ex. flying (USNM-4). [*Stevens Co.*]: Northport, 1.IX.1930, R. Hopping (CASC-3). [*Walla Walla Co.*]: Walla Walla, VIII.1933, M.C. Lume (USNM-11). [*Yakima Co.*]: [Mount Baker], Snoqualmie National Forest, Dry Creek Ridge, 14 mi W. of Naches, 3.VII.1965, R.B. Hutt (DEBC-1).

##### Distribution.

CANADA: British Columbia. MEXICO: Baja California. UNITED STATES: Arizona, California, Colorado, Idaho, Montana, Nevada, New Mexico, Oregon, Utah, Washington, Wyoming (Fig. 40).

##### Hosts.

Principle hosts: *Abies concolor* (Gord. & Glend.) Lindl. ex Hildebr. (white fir), *Abies grandis* (Douglas ex D. Don) Lindl. (grand fir), and *Abies magnifica* A. Murray (red fir). Incidental hosts: *Abies lasiocarpa* (Hook.) Nutt. (subalpine fir).

##### Common name.

Fir engraver.

##### Biology.

*Scolytus ventralis* can cause significant fir mortality and is the most destructive conifer-feeding *Scolytus* species in the Nearctic (Keen 1938; Bright and Stark 1973). During a period from 1924–1936, *Scolytus ventralis* killed 15% and damaged an additional 25% of the merchantable fir in California. It has also been reported to be quite destructive in Oregon (Keen 1938). *Scolytus ventralis* is associated with a symbiotic stain fungus, *Trichasporium symbioticum* Wright, which the adult beetle introduces when it excavates the adult gallery. The fungus spreads in all directions around the gallery system (Bright and Stark 1973). Due to the potential of *Scolytus ventralis* to kill fir trees, this species is the most well studied native *Scolytus* in North America. Attacks usually occur on the boles of weakened and stressed standing trees from a few feet above the base to the top of the tree, but can also occur in large slash and fresh and fallen trees (Chamberlin 1958; Edson 1967; Furniss and Johnson 2002). Attacks at the top of the tree are more common on overmature standing trees during drought and healthy, vigorous trees are not preferred (Chamberlin 1958; Raffa and Berryman 1987). Trees can also become successively attacked over a period of years and slowly die. Healthy trees may survive the attacks but can develop rots and defects that reduce timber value (Struble 1937). Unlike most tree-killing or primary bark beetles, *Scolytus ventralis* does not have pheromone to aggregate conspecifics to host trees. The beetles locate suitable hosts via primary attraction to host volatiles (Macías-Sámano et al. 1998a,b).

Adult galleries are perpendicular to the grain of the wood (Fig. 25), deeply score the sapwood, lightly score the cambium and consist of two egg galleries with a central nuptial chamber (Edson 1967). The nuptial chamber is typically short and at a right angle to the egg galleries but may also extend at an oblique angle. When this occurs, one of the egg galleries is briefly extended at an oblique angle against the grain before becoming perpendicular to the grain (Edson 1967). Eggs are deposited singly in triangular niches spaced 1.0–1.5 mm apart on each side of the egg gallery with 80–300 niches per gallery. Galleries range in size from 8.0–30.0 cm in length (Chamberlin 1958; Edson 1967; Bright and Stark 1973; Furniss and Johnson 2002). Larval mines are perpendicular to the egg gallery and parallel to the grain of the wood. Larval mines are also parallel to each other both above and below the egg gallery, giving the gallery a diamond shaped appearance (Keen 1938; Edson 1967). Larval mines lightly score the sapwood and deeply score the cambium. Larvae construct pupation chambers in the phloem or outer bark (Edson 1967). Broods overwinter as larvae or adults (Bright and Stark 1973). The number of generations per year varies both geographically and with elevation. Development time can range from as little as 41 days at low latitudes and elevations to as many as 380 days at high latitudes and elevations (Bright and Stark 1973). There is typically one generation per year (Bright and Stark 1973). In Idaho pupation occurs from June to July and peak flight occurs in July (Furniss and Johnson 2002).

##### Remarks.

The lectotype does not bear a locality label. LeConte’s (1868) description states that George Gibbs collected the lectotype in the Washington Territory.

### Key to the Nearctic adults of *Scolytus*

This is the first key to both sexes of Nearctic *Scolytus* adults. Unlike previous keys (e.g. Edson 1967; Wood 1982) apriori knowledge of the gender is not required. Only one sex is necessary to identify the specimen and host species or gallery types are not needed. Because the sexual dimorphic characters are not consistent for the Nearctic species (see sexual dimorphism discussion above), the sexes could not be evenly split. In general, males and females of native hardwood-feeding species will key out beginning at couplet 3 and both sexes of some *Abies* feeding species beginning at couplet 23. The key lists the gender of the identified specimen. If no gender is specified, the identification applies to both sexes. The length of frons setae is measured relative to the width of the midpoint of the eye.

**Table:** 

1	Elytral apices narrowly rounded (Fig. 60A)	***Scolytus rugulosus***
–	Elytral apices subquadrate (Fig. 60B) or broadly rounded	**2**
2	Lateral profile of frons clearly flattened and/or impressed (Fig. 1A)	**3**
–	Lateral profile of frons convex (Fig. 1C)	**23**
3	Apical margin of ventrite 3 and/or 4 armed with teeth or spines laterally and/ or medially	**4**
–	Apical margin of ventrite 3 and 4 unarmed	**7**
4	Ventrite 3 and 4 armed with lateral teeth or spines	**5**
–	Ventrites 3 and 4 not armed with lateral teeth or spines	**6**
5	Apical margin of ventrite 3 armed by three acute spines (two lateral and one medial); apical margin of ventrite 4 armed by one median tooth; ventrite 1 apically descending; ventrite 2 deeply concave, basal margin produced and bearing a median tubercle	***Scolytus quadrispinosus* (male)**
–	Apical margin of ventrite 3 and 4 each armed by two lateral teeth; ventrite 1 horizontal; ventrite 2 convex and bearing a blunt median tubercle on the basal margin	***Scolytus multistriatus* (male)**
6	Apical margin of ventrite 4 armed by an acute median denticle; apical margins of ventrites 2 and 3 may also each bear a smaller median denticle (variable)	***Scolytus dentatus* (male)**
–	Apical margin of ventrite 4 thickened forming a broad carina with a blunt median tubercle; apical margins of ventrites 2 and 3 always unarmed	***Scolytus silvaticus* (male)**
7	Ventrites 2–4 covered with abundant, erect, long, hair-like setae	**8**
–	Ventrites 2–4 glabrous or covered with minute ground vestiture or short, sparse, recumbent, hair-like or scale-like setae	**10**
8	Frons with hair-like setae on lateral and dorsal margins thicker, longer, incurved, remaining areas of frons largely devoid of setae; frons strongly longitudinally aciculate	***Scolytus muticus***
–	Frons with equally distributed hair-like setae of uniform length; frons weakly to moderately longitudinally aciculate or weakly aciculate-punctate	**9**
9	Apical margin of elytra produced between interstriae 1 and 2, deeply emarginate at interstria 3, produced on interstria 4 and deeply emarginate at stria 4; ventrite 2 armed with a laterally compressed tubercle that extends from the apical margin of ventrite 2 to approximately ¾ the length of ventrite; frons weakly longitudinally aciculate, strongly punctate; epistoma strongly emarginate	***Scolytus aztecus* (male)**
–	Apical margin of elytra slightly emarginate at interstriae 3; apical margin of ventrite 2 armed with a median denticle; frons moderately longitudinally aciculate, almost impunctate; epistoma entire to faintly emarginate	***Scolytus mundus* (male)**
10	Apical margin of ventrite 1 rounded; basal margin of ventrite 2 flat or marked by a weak carina; surface of ventrite 2 convex, often armed with a spine or tubercle (Fig. 61A)	**11**
–	Apical margin of ventrite 1 thickened or produced on the ventral and/or lateral margins, forming a carinate lip along the basal margin of ventrite 2, often weakly produced in *Scolytus monticolae* and *Scolytus tsugae*; surface of ventrite 2 flat to impressed (Fig. 61B)	**19**
11	Frons impressed just above epistoma or medially impressed, strongly longitudinally aciculate	**12**
–	Frons flat, weakly to moderately longitudinally aciculate or coarsely punctate	**15**
12	Ventrite 2 unarmed; elytral surface shining and glabrous	***Scolytus mali* (male)**
–	Ventrite 2 armed with a spine; elytral surface dull, sparse setae present (rarely the spine on ventrite 2 is absent in *Scolytus schevyrewi*)	**13**
13	Base of spine touching apical margin of ventrite 2; frons strongly longitudinally aciculate	***Scolytus laricis* (male)**
–	Base of spine not touching apical margin of ventrite 2; frons strigose-punctate	**14**
14	Spine conical, narrow; elytra always unicolorous; elytral disc shining and glabrous; subapical carina on ventrite 5 located 1/2 length of segment from apex	***Scolytus piceae* (male)**
–	Spine broadly conical with blunted apex; elytra often with a banded appearance; subapical carina on ventrite 5 located just before end of segment	***Scolytus schevyrewi* (male)**
15	Ventrite 2 armed with a rounded spine	**16**
–	Ventrite 2 unarmed	**17**
16	Spine on ventrite 2 extending from apical margin to three-fourths length of ventrite; ventrite 2 surface shining (Fig. 62A)	***Scolytus fiskei* (male)**
–	Spine on ventrite 2 extending from apical margin to one half length of ventrite; ventrite 2 surface dull (Fig. 62B)	***Scolytus unispinosus* (male)**
17	Ventrite 5 length at middle longer than or equal to that of ventrites 3 and 4 combined (Fig. 63A); ventrite 5 without a transverse carina near base	**18**
–	Ventrite 5 length at middle shorter than that of ventrites 3 and 4 combined (Fig. 63B); ventrite 5 with a transverse carinate ridge near base that may or may not be reflexed (variable)	***Scolytus reflexus* (male)**
18	Frons either granulate or faintly longitudinally aciculate; frons with setae uniformly distributed, fewer setae on lateral and dorsal margins, shorter, finer; elytra glabrous (except on declivity); ventrite 5 unarmed	***Scolytus fagi* (male)**
–	Frons moderately longitudinally aciculate, with long, fine, incurved setae predominately on lateral and dorsal margins, fewer, shorter and finer setae medially; elytra with minute ground vestiture	***Scolytus quadrispinosus* (female)**
19	Apical margin of ventrite 1 moderately produced and not forming a distinct lip along base of ventrite 2	2**0**
–	Apical margin of ventrite 1 distinctly thickened or strongly produced, forming a lip along base of ventrite 2	**21**
20	Surface of ventrite 2 shining but minutely reticulate; elytral striae not impressed; basal margin of ventrite 2 more pronounced and produced laterally; elytral strial punctures small, spaced 2–3 diameters of a puncture (Fig. 64A)	***Scolytus monticolae* (male)**
–	Surface of ventrite 2 opaque; elytral discal striae impressed; basal margin of ventrite 2 continuously and evenly elevated; elytral strial punctures large, spaced 1–2 diameters of a puncture (Fig. 64B)	***Scolytus tsugae* (male)**
21	Apical margin of ventrite 1 distinctly thickened, at most slightly produced over base of ventrite 2	**22**
–	Apical margin of ventrite 1 strongly acutely produced forming a lip along base of ventrite 2, surface of ventrite 2 appearing concave	***Scolytus robustus* (male)**
22	Base of ventrite 2 strongly thickened; surface of ventrite 2 often weakly medially impressed just above the base; apical margin of ventrite 2 unarmed (Fig. 65A)	***Scolytus oregoni* (male)**
–	Base of ventrite 2 faintly elevated; surface of ventrite 2 flat; apical margin of ventrite 2 slightly elevated often with a median denticle (Fig. 65B )	***Scolytus ventralis* (male)**
23	Apical margin of elytra produced between interstriae 1 and 2, deeply emarginate at interstria 3, produced on interstria 4 and deeply emarginate at stria 4	***Scolytus aztecus* (female)**
–	Apical margin of elytra entire or slightly emarginate at interstriae 3	**24**
24	Ventrite 2 armed either on the surface or on apical margin by either a spine or a low median tumescence	**25**
–	Ventrite 2 unarmed	**35**
25	Surface of ventrite 2 armed with a rounded, keel shaped or broadly acute spine that is at least as long as its basal width	**26**
–	Surface of ventrite 2 apical margin armed by a low median tumescence or small denticle	**30**
26	Base of spine on ventrite 2 touches the basal margin of segment; apical margins of ventrites 3 and 4 each armed by two lateral teeth	***Scolytus multistriatus* (female)**
–	Base of spine on ventrite 2 never touches basal margin of segment; apical margins of ventrites 3 and 4 unarmed	**27**
27	Base of spine not touching apical margin of ventrite 2	**28**
–	Base of spine touching apical margin of ventrite 2	**29**
28	Spine conical, narrow; elytra always unicolorous; elytral disc shining and glabrous; subapical carina on ventrite 5 located 1/5 length of segment from apex	***Scolytus piceae* (female)**
–	Spine broadly conical with blunted apex; elytra often with a banded appearance; subapical carina on ventrite 5 located just before end of segment	***Scolytus schevyrewi* (female)**
29	Ventrite 2 armed with a stout and broadly acute spine extending from the apical margin to one half-length of the ventrite, never with a longitudinal carina	***Scolytus fiskei* (female)**
–	Ventrite 2 armed with a longitudinal carina and a blunt tubercle, appearing keel-shaped, or a low median longitudinal carina	***Scolytus praeceps*, in part**
30	Apical margin of ventrite 1 produced, forming a carinate lip along the basal margin of ventrite 2; ventrite 2 appearing impressed (Fig. 61B)	**31**
–	Apical margin of ventrite 1 rounded; basal margin of ventrite 2 flat or marked by a weak carina; surface of ventrite 2 convex (Fig. 61A)	**34**
31	Apical margin of ventrite 2 weakly longitudinally tumescent, never pointed	***Scolytus praeceps* (male)**
–	Apical margin of ventrite 2 with a small median denticle	**32**
32	Apical margin of ventrite 1 slightly elevated; ventrite 2 appearing convex; median denticle on apical margin of ventrite 2 broad, broadly pointed	***Scolytus mundus* (female)**
–	Apical margin of ventrite 1 strongly, acutely produced, forming a lip along the base of ventrite 2, surface of ventrite 2 appearing impressed, median denticle on apical margin of ventrite 2 narrow, acutely pointed	**33**
33	Second ventrite punctures abundant, fine and moderately impressed, appearing distinct; second ventrite subopaque	***Scolytus obelus***
–	Second ventrite punctures sparse, fine and shallow, almost appearing indistinct; second ventrite appearing strongly opaque	***Scolytus subscaber* (male)**
34	Frons finely longitudinally aciculate-punctate (Fig. 66A); ventrite 1 joining base of ventrite 2 obtusely, base of ventrite 2 not finely impressed, flush with ventrite 1	***Scolytus unispinosus* (female)**
–	Frons moderately and coarsely longitudinally aciculate-punctate (Fig. 66B); ventrite 1 rounded over onto surface of ventrite 2, not forming an obtuse angle, base of ventrite 2 finely impressed	***Scolytus laricis* (female)**
35	Epistomal process absent (Fig. 67A)	**36**
–	Epistomal process present, may be indistinct (Fig. 67B)	**37**
36	Frons weakly longitudinally aciculate, strongly punctate, with uniformly distributed long setae; elytral striae weakly impressed	***Scolytus fagi* (female)**
–	Frons finely longitudinally aciculate and glabrous or minutely setose; elytral striae not impressed	***Scolytus mali* (female)**
37	Setae on frons at least 1.5 times the width of the eye at the middle	**38**
–	Setae on frons equal in length to the width of the eye at the middle	**42**
38	Ventrites 2–5 shining; thorax and apical and lateral margins of elytra covered with long hair-like setae as long as the length of ventrites 3 and 4 combined, with pointed apices	**39**
–	Ventrites 2–5 opaque; thorax covered with setae as long as the length of ventrite 3, with quadrate apices	**41**
39	Epistomal process weakly developed, and faintly emarginate medially	***Scolytus hermosus* (female)**
–	Epistomal process strongly developed, and strongly emarginate medially	**40**
40	Apical margin of ventrite 1 produced, forming a carinate lip along the basal margin of ventrite 2 that is about half as produced as thick; *Pseudotsuga menziesii*	***Scolytus silvaticus* (female)**
–	Apical margin of ventrite 1 produced, forming a carinate lip along the basal margin of ventrite 2 that is twice as produced as thick; *Abies religiosa*	***Scolytus hermosus* (male)**
41	Frons distinctly, moderately longitudinally aciculate, weakly punctate (Fig. 68A); apical margin of ventrite 1 weakly produced, forming a weak carinate lip along the basal margin of ventrite 2	***Scolytus subscaber* (female)**
–	Frons indistinctly, weakly longitudinally aciculate, strongly punctate (Fig. 68B); apical margin of ventrite 1 flush with basal margin of ventrite 2, appearing rounded	***Scolytus ventralis* (female)**
42	Apical margin of ventrite 1 rounded (Fig. 61A); surface of ventrite 2 rugose, shining, coarsely punctate and convex	***Scolytus reflexus* (female)**
–	Apical margin of ventrite 1 with thickened lip or produced (Fig. 61B), never rounded; surface of ventrite 2 smooth and flat	**43**
43	Apical margin of ventrite 1 thickened, on surface of ventrite 2 and nearly flush with the surface of ventrite 2	***Scolytus oregoni* (female)**
–	Apical margin of ventrite 1 not thickened or on the surface of ventrite 2, often slightly apically produced (unequally carinate in *Scolytus monticolae*)	**44**
44	Ventrite 2 punctulate with small or minute, shallow punctures	**45**
–	Ventrite 2 distinctly punctate with large and deep punctures	**47**
45	Apical margin of ventrite 1 equally and continuously carinate (Fig. 69A)	***Scolytus praeceps*, in part (female)**
–	Apical margin of ventrite 1 unequally carinate, more laterally produced forming cusps on each side of ventrite 2 (Fig. 69B)	**46**
46	Elytral discal striae not impressed; ventrite 2 shining in luster (Fig. 70A)	***Scolytus monticolae* (female)**
–	Elytral discal striae impressed, giving the elytra a corrugated appearance; ventrite 2 opaque, dull in luster (Fig. 70B)	***Scolytus tsugae* (female)**
47	Ventrite 2 shining	***Scolytus robustus* (female)**
–	Ventrite 2 dull, opaque	**48**
48	Epistomal process strongly developed, distinct; frons evenly convex	***Scolytus dentatus* (female)**
–	Epistomal process weakly developed, almost indistinct; frons flattened or convex with slight medial depression	***Scolytus praeceps*, in part**

**Figure 60.**
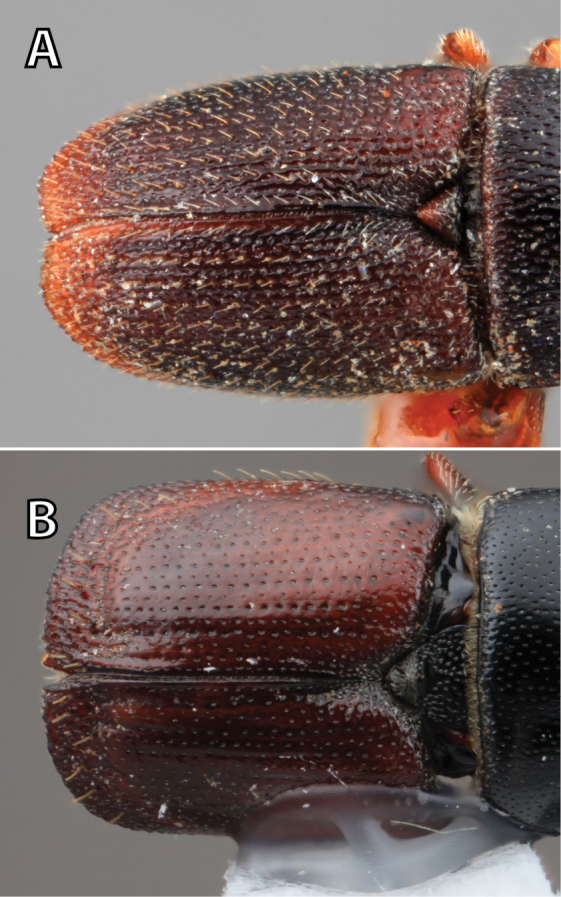
Elytral apices consist of two general shapes: **A** narrowly rounded (*Scolytus rugulosus*) **B** subquadrate (*Scolytus robustus*).

**Figure 61.**
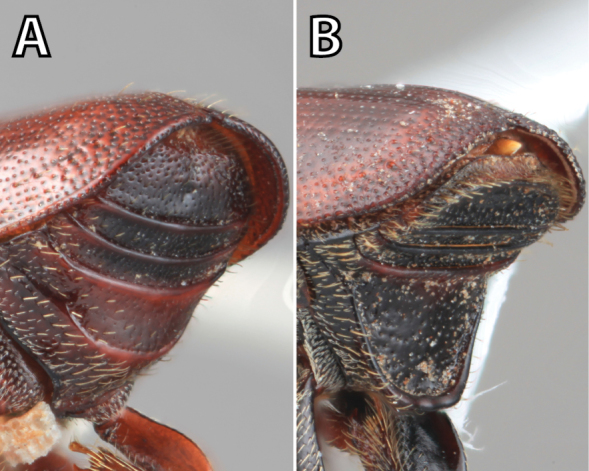
Apical margin of ventrite 1: **A** rounded (*Scolytus mali*) **B** thickened and produced forming a carinate lip (*Scolytus robustus*).

**Figure 62.**
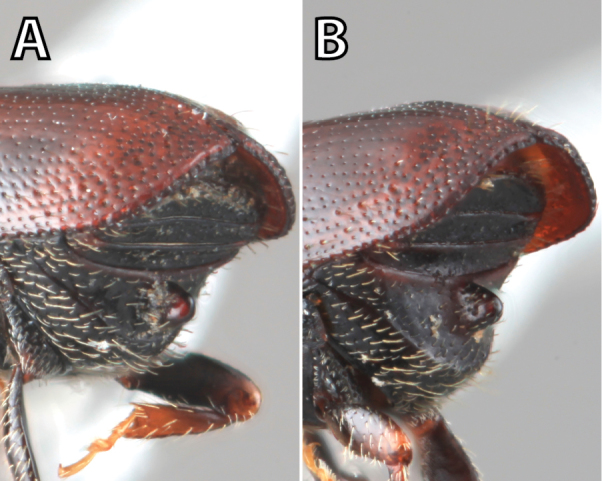
Spine on ventrite 2: **A** extending from apical margin to three-fourths length of ventrite **B** extending from apical margin to one half length of ventrite (*Scolytus fiskei*)

**Figure 63.**
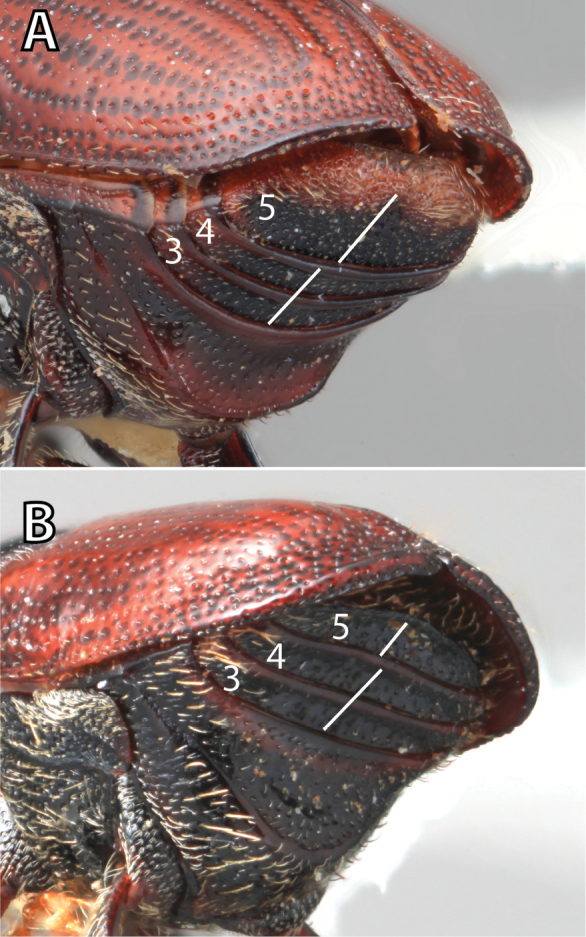
Length of ventrite 5 at middle: **A** longer than or equal to that of ventrites 3 and 4 combined (*Scolytus mali*) **B** shorter than that of ventrites 3 and 4 combined (*Scolytus reflexus*).

**Figure 64.**
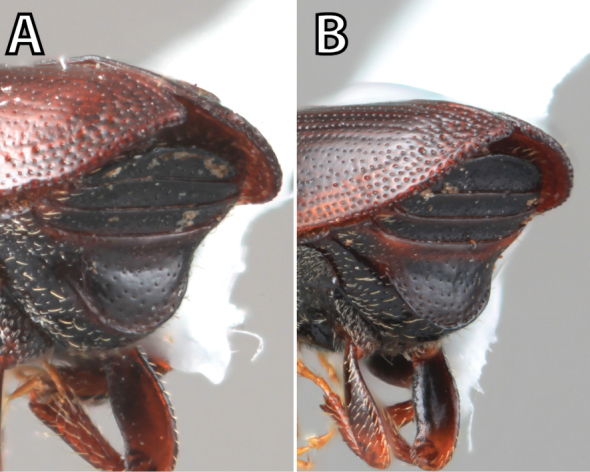
Surface of ventrite 2: **A** shining but minutely reticulate (*Scolytus monticolae*) **B** opaque (*Scolytus tsugae*).

**Figure 65.**
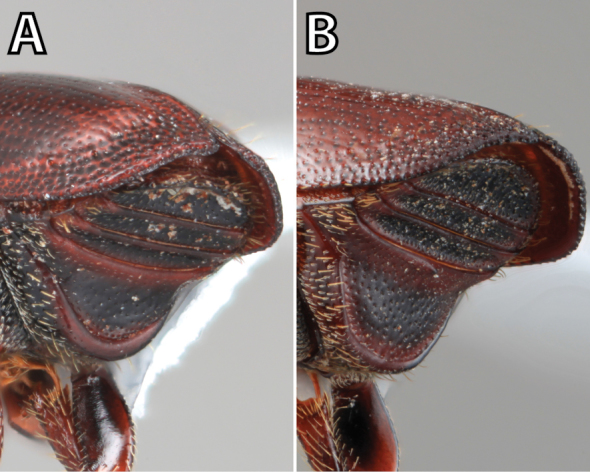
Base of ventrite 2: **A** distinctly thickened (*Scolytus oregoni*) **B** faintly elevated (*Scolytus ventralis*).

**Figure 66.**
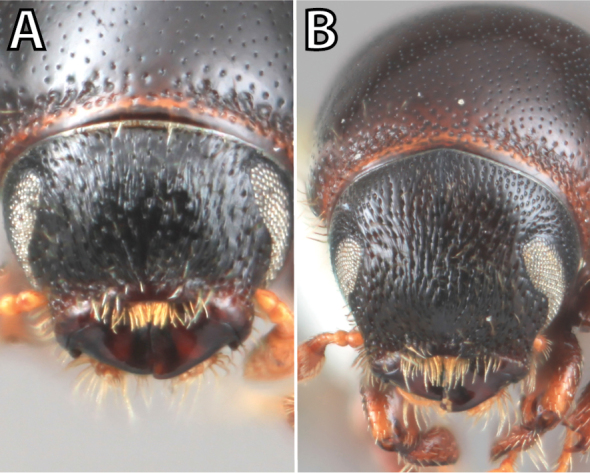
Frons: **A** finely longitudinally aciculate-punctate (*Scolytus unispinosus*) **B** moderately and coarsely longitudinally aciculate-punctate (*Scolytus fiskei*).

**Figure 67.**
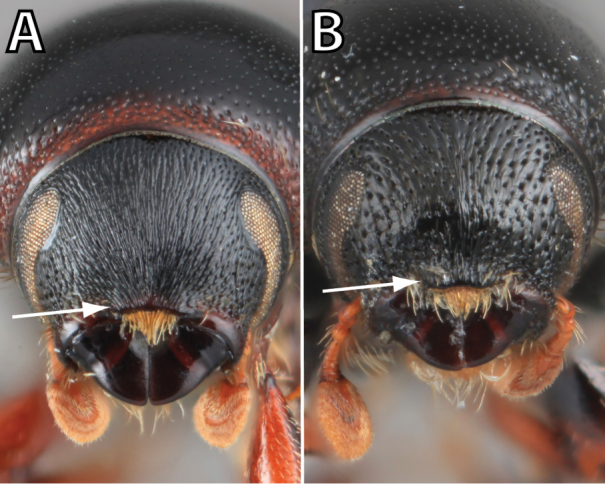
Epistomal process: **A** absent (*Scolytus mali*) **B** present (*Scolytus reflexus*).

**Figure 68.**
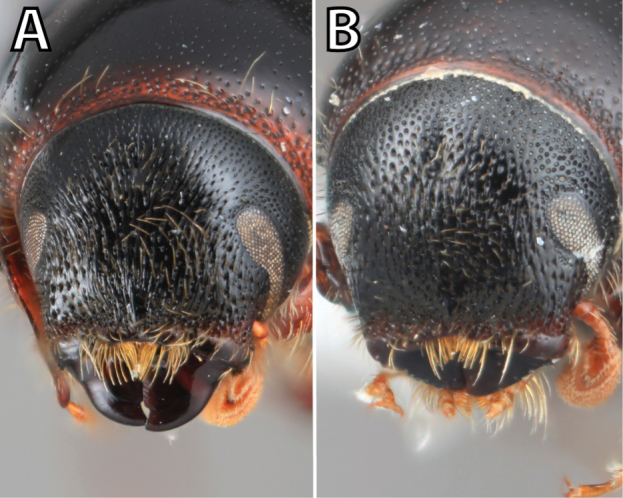
Frons: **A** distinctly, moderately longitudinally aciculate, weakly punctate (*Scolytus subscaber*) **B** indistinctly, weakly longitudinally aciculate, strongly punctate (*Scolytus ventralis*).

**Figure 69.**
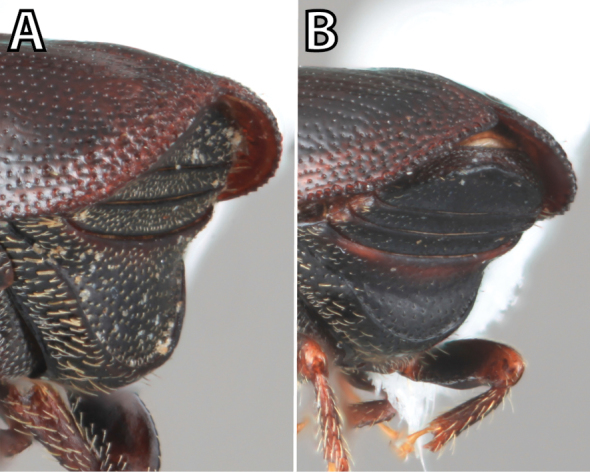
Apical margin of ventrite 1: **A** equally and continuously carinate (*Scolytus praeceps*) **B** unequally carinate, more laterally produced forming cusps on each side of ventrite 2 (*Scolytus tsugae*).

**Figure 70.**
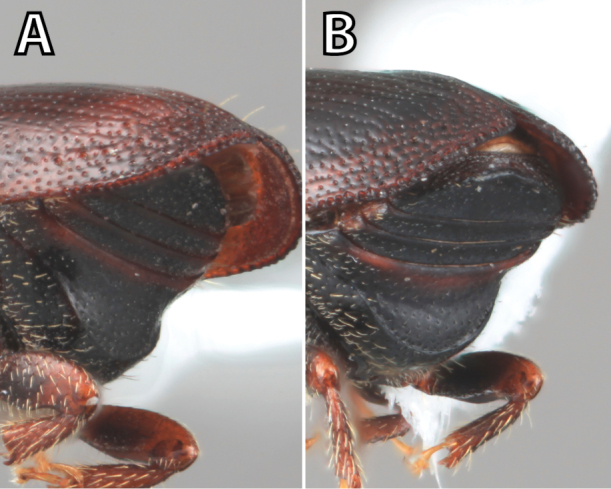
Ventrite 2: **A** shining in luster (*Scolytus monticolae*) **B** opaque, dull in luster (*Scolytus tsugae*).

## Discussion

Morphology failed to resolve the *Scolytus* phylogeny and very few synapomorphic characters were found (Fig. 2). Unique combinations of homoplastic characters define species limits. Characters that are among the most informative are sexually dimorphic and male including: male fifth ventrite carina (character 36), size and coarseness of frons punctures (character 7), first ventrite apical margin (character 27), second ventrite surface (character 29) and presence of an epistomal process (character 2). Other non-dimorphic informative characters included elytral apex emargination (character 20), host (character 43), and strial impression (character 18). The lack of phylogenetically informative characters necessitated the need for further investigation using molecular characters to illuminate species boundaries.

As in other phylogenetic studies, COI data failed to recover many species as monophyletic (Fig. 3) (Cognato et al. 2005; Jordal and Hewitt 2004). Several relationships found in this phylogeny did not agree with the topologies obtained from different analyses. For example a hardwood-feeder, *Scolytus rugulosus*, was found to be sister to the conifer clade in the Bayesian analysis and *Scolytus ventralis* was found to be sister to the Nearctic conifer clade. In addition, the hardwood clade was paraphyletic with *Scolytus fagi* and *Scolytus muticus* forming one lineage and *Scolytus quadrispinosus* as sister to *Scolytus multistriatus*. Saturation of nucleotide substitutions and a lack of lineage sorting are potential reasons for the discordance among these mitochondrial and nuclear phylogenies (Sota and Vogler 2001; Funk and Ohmland 2003; Lin and Danforth 2004). The mitochondrial phylogeny may suffer from both phenomena because of the observed poor resolution and support for deeper nodes and short-branch lengths among the conifer-feeding species (Fig. 3). As with other studies, concatenating the COI dataset with multiple genes and morphology remedied its deficiency and elucidated the relationships among the *Scolytus* species (Jordal and Hewitt 2004).

When analyzed together, the genes used in this study (COI, 28S, CAD, ArgK) were useful in resolving the *Scolytus* phylogeny and species limits. These genes have demonstrated similar phylogenetic utility in other scolytine studies (Cognato and Sperling 2000; Farrell et al. 2001; Jordal et al. 2002; Jordal and Hewitt 2004; Cognato et al. 2005; Cognato and Sun 2007; Jordal et al. 2008; Dole et al. 2010; Cognato et al. 2011; Jordal et al. 2011; Jordal and Cognato 2012). Overall, the model-based rates of nucleotide evolution utilized in Bayesian analysis combined with all four genes and morphology allowed us to recover a well-supported and sufficiently resolved phylogeny which enabled the revision of *Scolytus* (Fig. 4).

Nearctic *Scolytus* were recovered as paraphyletic in two clades, native hardwood and conifer (Fig. 4). A group of introduced Palearctic species was also found. Members of the native hardwood clade are sister to the *Scolytus scolytus* subgenus *sensu* Butovitsch (1929) rather than the native conifer clade. This is supported by morphological similarities between the two groups. The clade containing the *Scolytus scolytus* subgenus *sensu* Butovitsch and the native hardwood-feeders share a median impression on male ventrite 5 (except *Scolytus laevis* and *Scolytus quadrispinosus*) and a setal patch on ventrite 5 (except *Scolytus laevis* and *Scolytus quadrispinosus*). Several species in these groups also share the absence of a carina on male ventrite 5 (*Scolytus laevis*, *Scolytus mali*, *Scolytus ratzeburgii*, *Scolytus fagi* and *Scolytus muticus*).

## Supplementary Material

XML Treatment for Scolytus

XML Treatment for Scolytus mali

XML Treatment for Scolytus multistriatus

XML Treatment for Scolytus rugulosus

XML Treatment for Scolytus schevyrewi

XML Treatment for Scolytus fagi

XML Treatment for Scolytus muticus

XML Treatment for Scolytus quadrispinosus

XML Treatment for Scolytus aztecus

XML Treatment for Scolytus dentatus

XML Treatment for Scolytus fiskei

XML Treatment for Scolytus hermosus

XML Treatment for Scolytus laricis

XML Treatment for Scolytus monticolae

XML Treatment for Scolytus mundus

XML Treatment for Scolytus obelus

XML Treatment for Scolytus oregoni

XML Treatment for Scolytus piceae

XML Treatment for Scolytus praeceps

XML Treatment for Scolytus reflexus

XML Treatment for Scolytus robustus

XML Treatment for Scolytus silvaticus

XML Treatment for Scolytus subscaber

XML Treatment for Scolytus tsugae

XML Treatment for Scolytus unispinosus

XML Treatment for Scolytus ventralis
